# Combined Genotypic, Phylogenetic, and Epidemiologic Analyses of *Mycobacterium tuberculosis* Genetic Diversity in the Rhône Alpes Region, France

**DOI:** 10.1371/journal.pone.0153580

**Published:** 2016-04-29

**Authors:** Catherine Pichat, David Couvin, Gérard Carret, Isabelle Frédénucci, Véronique Jacomo, Anne Carricajo, Sandrine Boisset, Oana Dumitrescu, Jean-Pierre Flandrois, Gérard Lina, Nalin Rastogi

**Affiliations:** 1 Laboratoire de bactériologie, Centre hospitalier Lyon-Sud, Pierre-Bénite, France; 2 WHO Supranational TB Reference Laboratory, Institut Pasteur de la Guadeloupe, Abymes, France; 3 Laboratoire de Biométrie et Biologie Évolutive, CNRS-UMR 5558, Université Lyon 1, Villeurbanne, France; 4 Service des mycobactéries, Laboratoire Biomnis, Lyon, France; 5 Laboratoire de bactériologie, CHU de Saint-Étienne Nord, Saint-Étienne, France; 6 Laboratoire de bactériologie, CHU de Grenoble, Grenoble, France; 7 Centre Internationl de Recherche en Infectiologie, INSERM U1111, Université Lyon 1, Lyon, France; University of Padova, Medical School, ITALY

## Abstract

**Background:**

The present work relates to identification and a deep molecular characterization of circulating *Mycobacterium tuberculosis* complex (MTBC) strains in the Rhône-Alpes region, France from 2000 to 2010. It aimed to provide with a first snapshot of MTBC genetic diversity in conjunction with bacterial drug resistance, type of disease and available demographic and epidemiologic characteristics over an eleven-year period, in the south-east of France.

**Methods:**

*Mycobacterium tuberculosis* complex (MTBC) strains isolated in the Rhône-Alpes region, France (n = 2257, 1 isolate per patient) between 2000 and 2010 were analyzed by spoligotyping. MIRU-VNTR typing was applied on n = 1698 strains (with full results available for 974 strains). The data obtained were compared with the SITVIT2 database, followed by detailed genotyping, phylogenetic, and epidemiologic analyses in correlation with anonymized data on available demographic, and epidemiologic characteristics, and location of disease (pulmonary or extrapulmonary TB).

**Results:**

The most predominant spoligotyping clusters were SIT53/T1 (n = 346, 15.3%) > SIT50/H3 (n = 166, 7.35%) > SIT42/LAM9 (n = 125, 5.5%) > SIT1/Beijing (n = 72, 3.2%) > SIT47/H1 (n = 71, 3.1%). Evolutionary-recent strains belonging to the Principal Genetic Group (PGG) 2/3, or Euro-American lineages (T, LAM, Haarlem, X, S) were predominant and represented 1768 or 78.33% of all isolates. For strains having drug resistance information (n = 1119), any drug resistance accounted for 14.83% cases vs. 1.52% for multidrug resistance (MDR); and was significantly more associated with age group 21–40 years (p-value<0.001). Extra-pulmonary TB was more common among female patients while pulmonary TB predominated among men (p-value<0.001; OR = 2.16 95%CI [1.69; 2.77]). Also, BOV and CAS lineages were significantly well represented in patients affected by extra-pulmonary TB (p-value<0.001). The origin was known for 927/2257 patients: 376 (40.6%) being French-born vs. 551 (59.4%) Foreign-born. French patients were significantly older (mean age: 58.42 yrs 95%CI [56.04; 60.80]) than Foreign-born patients (mean age: 42.38 yrs. 95%CI [40.75; 44.0]).

**Conclusion:**

The study underlined the importance of imported TB cases on the genetic diversity and epidemiologic characteristics of circulating MTBC strains in Rhône-Alpes region, France over a large time-period. It helps better understand intricate relationships between certain lineages and geographic origin of the patients, and pinpoints genotypic and phylogenetic specificities of prevailing MTBC strains. Lastly, it also demonstrated a slow decline in isolation of *M*. *africanum* lineage in this region between 2000 and 2010.

## Introduction

Tuberculosis (TB) still represents a challenging and serious public health problem despite the fact that many efforts are ongoing to eradicate the disease. According to the Global report 2015 [1], as high as 9.6 million people were estimated to have fallen ill with TB in 2014 worldwide (12% co-infected with HIV) and 1.5 million people died from it; with an alarmingly increasing problem of multidrug-resistant TB (MDR-TB). Despite a great effort to fight TB dissemination in France and despite the fact that incidence of new TB cases fell significantly in France between 2000 and 2010, the disease has remained a visible threat to public health—especially in precarious populations and migrants—with an increase in MDR-TB cases (http://sante.lefigaro.fr/actualite/2014/03/18/22121-tuberculose-recule-france-sauf-seine-saint-denis). With a mean incidence of 8.1/100,000 inhabitants (or 5187 cases) in 2010 according to the “Regional Health Agency (Agence Régionale de la Santé, ARS), its prevalence was highly variable in different French regions with significantly higher incidences in 3 regions, namely—Ile de France, Provence Alpes Cotes d’Azur, and Rhône-Alpes, which collectively totalized more than the half of the declared TB cases in France [2]. In the Rhône-Alpes region, 439 TB cases were declared in 2010, corresponding to an incidence of 7.1/100,000 inhabitants.

Innovations in the molecular epidemiology of tuberculosis in conjunction with phylogenetical analyses, databases and web tools allow today not only to better understand and monitor the evolution of TB, but also help to comprehend intricate relationships between certain lineages and geographic origin of the patients, emergence of drug-resistance, and to draw important conclusions regarding risk factors for developing the disease [3–5]. For example, recent reports have highlighted the impact of immigration on imported cases of disease in low incidence Western European countries [reviewed in 3]. However, with the exception of a single 5-year retrospective study on *Mycobacterium bovis* infections in Lyon [6], no such studies have yet been performed in the Rhône-Alpes region, France. We therefore decided to retrospectively analyze all *Mycobacterium tuberculosis* complex (MTBC) strains isolated in the Rhône Alpes (France) over an eleven year period (2000–2010), for which the genotyping results were available under a pooled systematic genotyping program among various participating centers (n = 2257 isolates). This analysis was meant to characterize existing transmission patterns, assess the impact of imported vs. local TB cases, and study combined genotypic, phylogenetic, epidemiologic and demographic correlations over a large timeframe. We believe that the results obtained under this large population-based study represent an important initiative for the supervision, control, and eradication of TB in low-incidence western countries.

## Materials and Methods

### Ethical considerations

Systematic genotyping of MTBC strains was introduced in 2000 at the laboratory of Centre Hospitalier Lyon-Sud. The “Observatoire Rhône Alpes des Mycobactéries” (ORAM) was set up in 2005 in order to facilitate a study between several laboratories and regional anti-TB centers (“Centre de Lute Anti-Tuberculeuse” or CLAT), as well as the regional health agency (Agence Régionale de la Santé, ARS). The genotyping of MTBC strains isolated in the Rhône-Alpes region targeting “an improved TB control” was approved by the “Comités de Protection des Personnes—Sud Est IV” (approval number: DC-2011-1306). It should be noted that: (i) all sampling and testing were made as part of diagnosing the disease and drug resistance for patient benefit, and (ii) all genotyping data were anonymized prior to reporting to the SITVIT2 database curator for an anonymous retrospective comparison, focusing on genotypic and phylogenetic analysis of MTBC genetic diversity.

### Bacterial isolates, genotyping and database comparison

Data obtained on a total of 2257 MTBC isolates (1 strain per patient) isolated during 2000–2010 in the Rhône Alpes region, France were used in this study. Available epidemiologic and demographic characteristics such as the origin of patients, sex and age, results of chest radiographs and localization of the disease (pulmonary vs. extrapulmonary disease), drug resistance, and available microbiological data were recorded. Drug susceptibility testing (DST) was performed using the Bactec MGIT 960 system (BD Diagnostics, Sparks MD, USA) according to the manufacturer’s recommendations with following drug concentrations: streptomycin (STR), 1.0 μg/ml; isoniazid (INH), 0.2 and 0.1 μg/ml; rifampicin (RIF), 1.0 μg/ml; ethambutol (EMB), 5.0 μg/ml, and pyrazinamide (PYR), 100 μg/ml.

Spoligotyping was performed on all strains (n = 2257) as reported [7], while the MIRU-VNTR typing which was a method available later, was applied as described [8] to strains isolated from 2005 onwards (n = 1698 strains with full copy numbers available for 974 strains). Two formats of MIRU-VNTRs were performed: (i) the 12-loci format corresponded to MIRU loci in the order: MIRU 2, 4, 10, 16, 20, 23, 24, 26, 27, 31, 39 and 40; (ii) the 15-loci format corresponded to MIRU loci in the order: MIRU 4, 10, 16, 26, 31, and 40, ETRA, ETRC, QUB-11b, QUB-26, QUB-4156, Mtub04, Mtub21, Mtub30, and Mtub39.

For computer assisted genotyping analysis and comparison with an international database, spoligotype patterns as octal codes were entered in the SITVIT2 proprietary database of the Institut Pasteur de la Guadeloupe [9,10] which is an updated version of the previously released SpolDB4 [11] and SITVITWEB [12] databases. At the time of this study, SITVIT2 contained a total of 111,635 MTBC clinical isolates from 169 countries of patient origin. In this database, Spoligotype International Type (SIT) and a 12 or 15-MIRU International Type (12MIT or 15MIT) designate patterns shared by two or more isolates, whereas “orphan” designates patterns reported for a single isolate.

Genotypic lineages were assigned according to revised rules [10] as various MTBC members (AFRI, *Mycobacterium africanum*; BOV, *Mycobacterium bovis*; CANETTII, *Mycobacterium canettii*; MICROTI, *Mycobacterium microti*; PINI, *Mycobacterium pinnipedii*), as well as for *M*. *tuberculosis* sensu stricto, i.e., the Beijing lineage, the Central-Asian (CAS) lineage, the East-African-Indian (EAI) lineage, the Haarlem/Ural lineages, the Latin-American-Mediterranean (LAM) lineage, the Cameroon and Turkey lineages, the “Manu” lineage, the IS*6110*-low banding X lineage, and the ill-defined T lineage. Note that two LAM sublineages were recently raised to independent lineage level: LAM10-CAM as the Cameroon lineage [13], and LAM7-TUR as the Turkey lineage [14, 15]. Lastly, some spoligotypes previously classified as H3/H4 or H4 sublineages were relabeled “Ural” [16]; these include patterns belonging to H4 sublineage that were relabeled “Ural-2”, and some patterns previously classified as H3 sublineage but with an additional specific signature (presence of spacer 2, absence of spacers 29 to 31, and 33–36), that are now relabeled “Ural-1”. These revised signatures are referred to as Haarlem/Ural in global analysis (visible in S1, S2 and S3 Tables).

Lastly, distinction of strains as belonging to ancient Principal Genetic Group (PGG) 1 group (AFRI, Beijing, BOV, CAS, and EAI) versus evolutionary-recent PGG2/3 group of Euro-American lineages (T, LAM, Haarlem, X, S) was done centered on *katG-gyrA* polymorphism [17], as inferred from published data on linking of spoligotyping based phylogenetical lineages with PGG groupings and the presence of a specific deletion region TbD1 [18, 19].

### Phylogenetical analysis and geographical distribution

BioNumerics software version 6.6 (Applied Maths, Sint-Martens-Latem, Belgium) was used to compare spoligotypes and MIRU-VNTRs patterns, by drawing Minimum spanning trees (MSTs) in order to visualize evolutionary relationships between the clinical isolates in our study. MSTs are undirected graphs in which all samples are connected together with the fewest possible connections between nearest neighbors [20].

SpolTools software (available through http://www.emi.unsw.edu.au/spolTools) was used to draw spoligoforest tree based on the Fruchterman-Reingold algorithm [21, 22]. Contrary to the MSTs, spoligoforests are directed and not necessarily connected graphs. They permitted to highlight the evolutionary relationships between ascendant and descendant spoligotyping patterns.

The MLVA Compare software developed by Ridom and Genoscreen (http://www.ridom.de/) was also used to draw supplemental trees (an UPGMA tree and a MST) using the labels provided by MIRU-VNTR plus web server (http://www.miru-vntrplus.org/) in order to visualize deeper associations between spoligotypes and MIRU-VNTRs involved in this study. MIRU-VNTRplus is a collection of 186 strains representing the major *M*. *tuberculosis* complex lineages with data on strain species, lineage, and epidemiologic and genotyping information such as copy numbers of 24 MIRU loci, spoligotyping patterns, regions of difference (RD) profiles, single nucleotide polymorphisms (SNPs), susceptibility data, and IS*6110* RFLP fingerprint images [23, 24].

**Fig 1:**
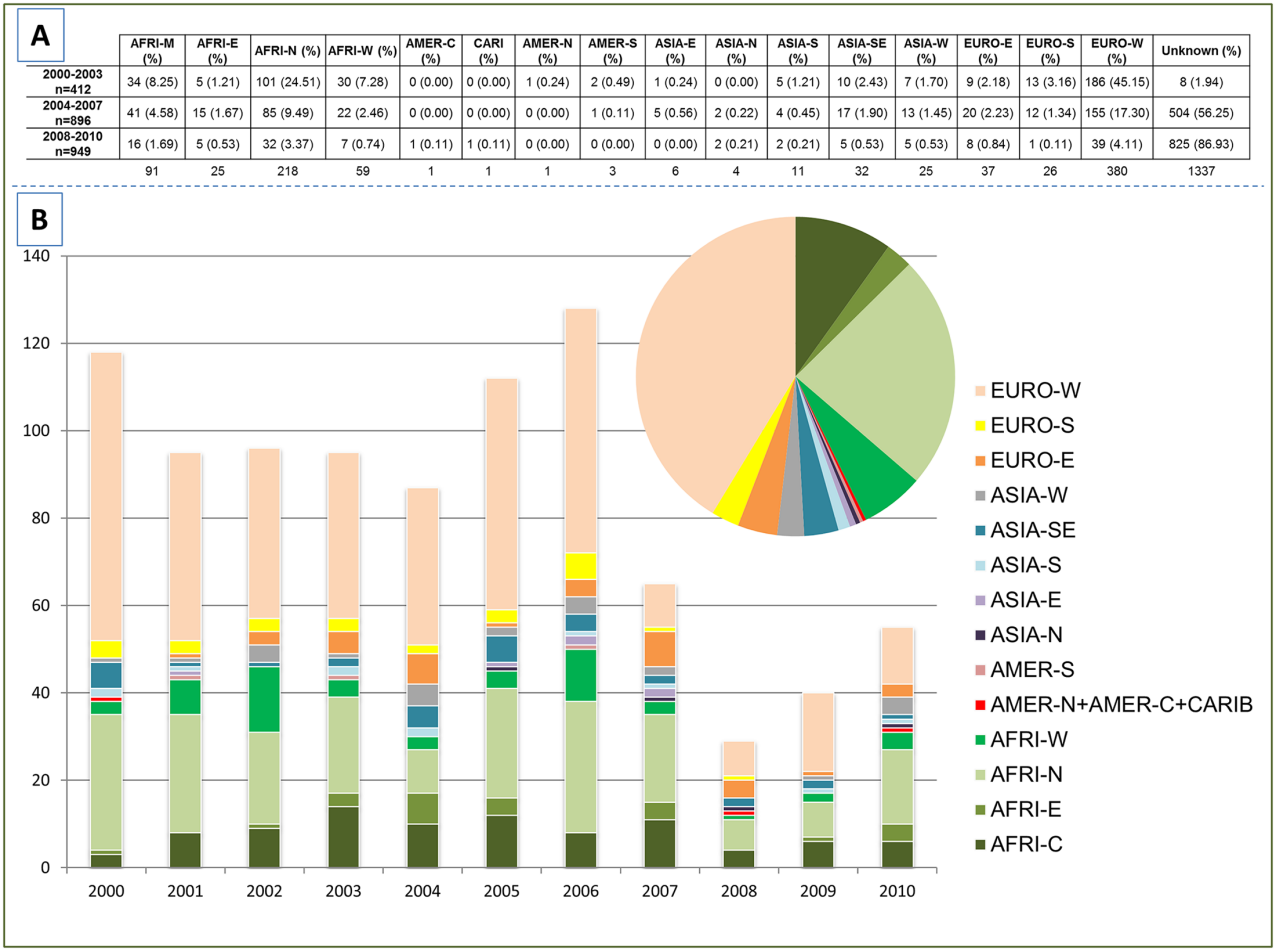
Number of patients considering their subregion of origin per groups of year (A); and per year of study (from 2000–2010) (B). Note that although 927 patients were known to be foreign-born (as shown in Table 2), the exact geographical origin allowing to classify them in a specific UN subregion lacked for 7 of 927 foreign-born patients.

Geographical specificities of MTBC strains have already been demonstrated previously [25]. In our study, the worldwide distribution of *M*. *tuberculosis* strains was studied both at the country level (3 letter country codes according to http://en.wikipedia.org/wiki/ISO_3166-1_alpha-3), as well as macro-geographical (United Nations subregions at http://unstats.un.org/unsd/methods/m49/m49regin.htm); Note that Russia was attributed a new subregion by itself (Northern Asia) instead of including it among the rest of Eastern Europe. Fig 1 summarizes the different regions of origin of patients involved in our study.

### Statistical analysis

STATA software version 12 and MEGASTAT 12 (a statistical tool developed by The McGraw-Hill Companies which could be used as an add-in in Microsoft Excel) were used for descriptive, univariate and multivariate (logistic regression) analyses. Comparisons between different genotyping, demographic and epidemiological parameters were performed using Pearson's Chi-square test and Fisher's Exact Test. Student's t-test was used to compare age distribution between foreign and French patients. P values of <0.05 were considered as statistically significant. Odds Ratios (OR) and 95% Confidence Intervals (CI) were calculated to better visualize the significance of the statistical tests.

## Results

### MTBC population Structure and Phylogenetic Analysis

The present study comprised 2257 MTBC isolates from the Rhône-Alpes Region, 182 strains corresponded to orphan patterns, while a SIT number was attributed for 433 patterns totaling 2075 strains (S1 and S2 Tables respectively). The proportion of SITed vs. orphan strains between French and foreign-born patients (90.16% vs. 92.56% respectively), may be suggestive of a higher TB transmission inside the foreign-born group. Readers interested in detailed results including demographic, epidemiologic, drug-resistance, and genotyping information on all the 2257 strains are referred to S3 Table. As compiled from this table, the distribution of the spoligotyping based lineages in this study was as follows: T (n = 762, 33.76%); Haarlem (n = 496, 21.98%); LAM (n = 360, 15.95%); BOV (n = 95, 4.21%); Beijing (n = 84, 3.72%); EAI (n = 66, 2.92%); S (n = 63, 2.79%); the Cameroon lineage (previously labeled LAM10-CAM; n = 53, 2.35%); AFRI (n = 36, 1.60%); CAS (n = 30, 1.33%); Ural (n = 18, 0.80%); X (n = 9, 0.40%); the Turkey lineage (previously labeled LAM7-TUR; n = 7, 0.31%). The lineage designation was unknown for n = 178 or 7.89% of all strains.

**Fig 2:**
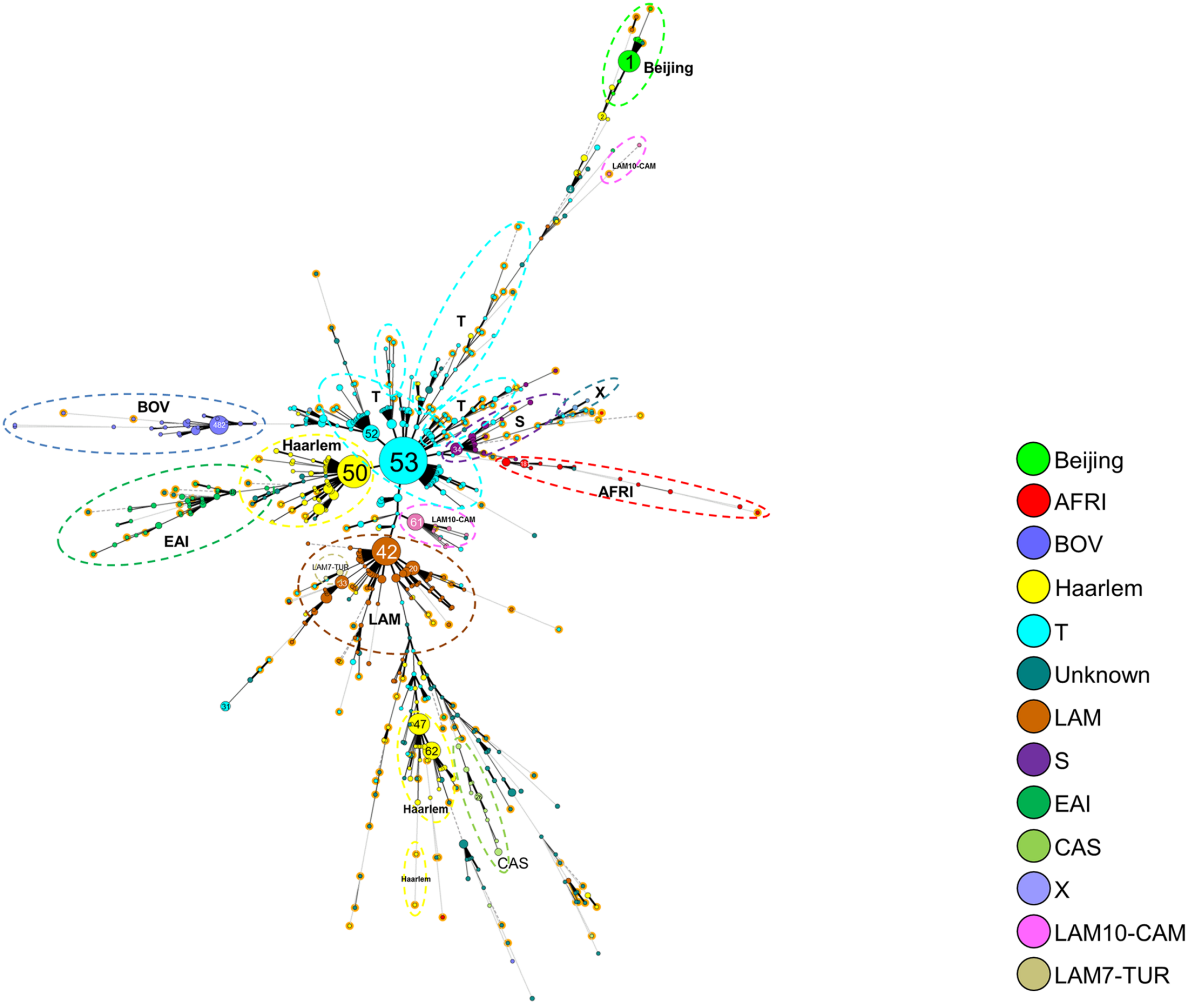
A minimum spanning tree (MST) illustrating evolutionary relationships between *M*. *tuberculosis* spoligotypes. The phylogenetic tree was constructed on both SITed isolates (n = 2075) as well as orphan patterns (n = 182), i.e. all the 2257 MTBC isolates. It connects each genotype based on degree of changes required to go from one allele to another. The structure of the tree is represented by branches (continuous vs. dashed and dotted lines) and circles representing each individual pattern. Note that the length of the branches represent the distance between patterns while the complexity of the lines (continuous, gray dashed and gray dotted) denotes the number of allele/spacer changes between two patterns: solid lines, 1 or 2 or 3 changes (thicker ones indicate a single change, while the thinner ones indicate 2 or 3 changes); gray dashed lines represent 4 changes; and gray dotted lines represent 5 or more changes. The size of the circle is proportional to the total number of isolates in our study, illustrating unique isolates (smaller nodes) versus clustered isolates (bigger nodes). The color of the circles indicates the phylogenetic lineage to which the specific pattern belongs. Note that orphan patterns are circled in orange (182 patterns belonged to orphans in our study). Note that the numbers in the nodes represent the SIT numbers.

A minimum spanning tree (MST) was drawn using all clustered spoligotypes (SITs) representing n = 2075 isolates (Fig 2). The resulting MST clearly shows the relationships between each spoligotyping pattern, one may notice that ancient Principal Genetic Group (PGG) 1 strains (AFRI, Beijing, BOV, CAS, and EAI) mostly appeared at the final branches of the tree whereas evolutionary recent PGG2/3 strains were more centralized in the figure. The SITs featuring on the MST can be referred to in detail in S2 Table. The most predominant spoligotyping clusters were organized as follows: SIT53/T1 (n = 346, 15.3%) > SIT50/H3 (n = 166, 7.35%) > SIT42/LAM9 (n = 125, 5.5%) > SIT1/Beijing (n = 72, 3.2%) > SIT47/H1 (n = 71, 3.1%).

**Fig 3:**
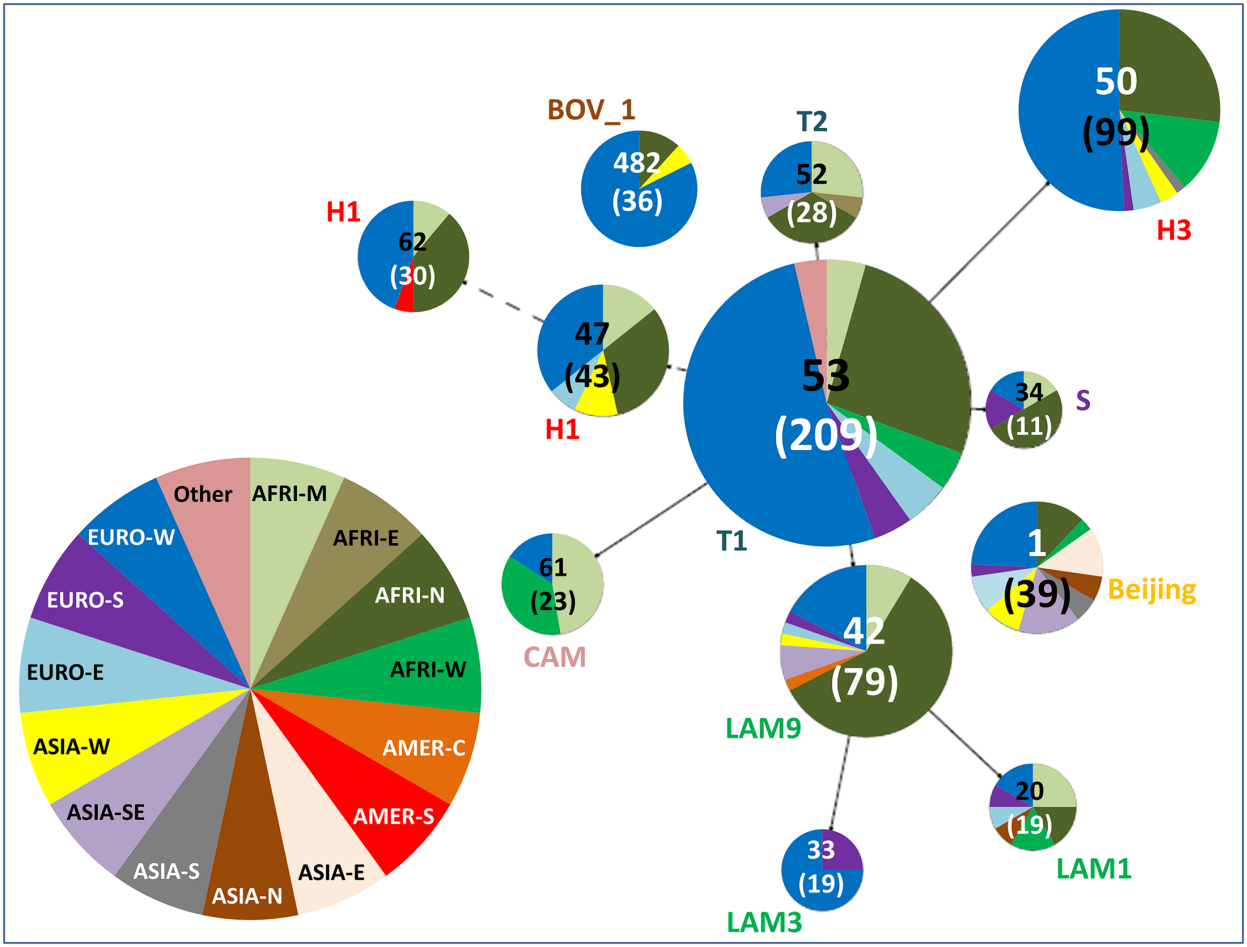
The Spoligoforest tree based on the Fruchterman Reingold algorithm. The tree represents only the predominant SITs, giving the proportion and distribution of these SITs among the different origin of patients involved in the study. In this tree, each spoligotype pattern from the study is represented by a node with area size being proportional to the total number of isolates with that specific pattern (shown in brackets). Note that the numbers outside of brackets represent the SIT number for each specific pattern. Changes (loss of spacers) are represented by directed edges between nodes, with the arrowheads pointing to descendant spoligotypes. In this representation, the heuristic used selects a single inbound edge with a maximum weight using a Zipf model. Solid black lines link patterns that are very similar, i.e., loss of one spacer only (maximum weigh being 1.0), while dashed lines represent links of weight comprised between 0.5 and 1, and dotted lines a weight less than 0.5.

Major SITs representing at least 1% of all isolates (i.e. SITs having 23 isolates or more) and their worldwide distribution in the SITVIT2 database is summarized in Table 1, while a Spoligoforest tree drawn using the Fruchterman-Reingold algorithm from the SpolTools software highlights the proportion and distribution of these SITs among the different origin of patients involved in the study (Fig 3). In this tree, each spoligotype pattern from the study is represented by a node with area size being proportional to the total number of isolates with that specific pattern (shown in brackets). Note that the numbers outside of brackets represent the SIT number for each specific pattern.

**Table 1:** Description of clusters containing 23 or more isolates in this study, and their worldwide distribution in the SITVIT2 database.

SIT (Lineage) Octal Number Spoligotype Description	Number (%) in study	% in study vs. database	Distribution in Regions with > = 3% of a given SIT *	Distribution in countries with > = 3% of a given SIT **
1 (Beijing) 000000000003771 ▫▫▫▫▫▫▫▫▫▫▫▫▫▫▫▫▫▫▫▫▫▫▫▫▫▫▫▫▫▫▫▫▫▫▪▪▪▪▪▪▪▪▪	72 (3.19)	0.71	ASIA-E 31.94, AMER-N 19.53, ASIA-SE 9.83, AFRI-S 8.06, ASIA-N 6.77, ASIA-S 5.1, EURO-N 3.56, AMER-S 3.48	USA 19.28, CHN 18.46, JPN 11.2, ZAF 8.06, RUS 6.77, VNM 3.79, PER 3.07
20 (LAM1) 677777607760771 ▪▪▫▪▪▪▪▪▪▪▪▪▪▪▪▪▪▪▪▪▫▫▫▫▪▪▪▪▪▪▪▪▫▫▫▫▪▪▪▪▪▪▪	31 (1.37)	3.56	AMER-S 26.72, AMER-N 18.23, EURO-W 10.55, AFRI-S 10.21, CARI 9.75, EURO-S 9.06, AFRI-E 4.24	USA 18.23, BRA 17.09, HTI 7.8, NAM 7.11, PRT 5.62, FXX 5.62, VEN 4.82, ZAF 3.1
33 (LAM3) 776177607760771 ▪▪▪▪▪▪▪▪▫▫▫▪▪▪▪▪▪▪▪▪▫▫▫▫▪▪▪▪▪▪▪▪▫▫▫▫▪▪▪▪▪▪▪	27 (1.2)	2.38	AFRI-S 28.61, AMER-S 25.18, AMER-N 14.0, EURO-S 12.5, EURO-W 7.22, AMER-C 4.75	ZAF 28.61, USA 14.0, BRA 11.97, ESP 7.84, PER 5.99, ARG 5.02, FXX 4.67, ITA 4.05, HND 3.79
34 (S) 776377777760771 ▪▪▪▪▪▪▪▪▫▫▪▪▪▪▪▪▪▪▪▪▪▪▪▪▪▪▪▪▪▪▪▪▫▫▫▫▪▪▪▪▪▪▪	23 (1.02)	2.67	AMER-N 24.1, AFRI-S 17.85, EURO-S 14.72, AMER-S 10.89, EURO-W 9.27, AFRI-N 4.17, CARI 3.71, EURO-E 3.59, ASIA-W 3.48, EURO-N 3.24	ZAF 17.85, USA 15.41, ITA 12.28, CAN 8.57, BRA 6.37, FXX 4.17, BGR 3.59
42 (LAM9) 777777607760771 ▪▪▪▪▪▪▪▪▪▪▪▪▪▪▪▪▪▪▪▪▫▫▫▫▪▪▪▪▪▪▪▪▫▫▫▫▪▪▪▪▪▪▪	125 (5.54)	3.67	AMER-S 29.21, AMER-N 11.65, EURO-S 11.12, EURO-W 9.3, AFRI-N 8.42, EURO-N 4.81, CARI 4.14, AMER-C 3.49, AFRI-E 3.49, AFRI-S 3.11	BRA 12.3, USA 11.65, COL 7.49, MAR 6.93, ITA 6.43, FXX 4.96, ESP 3.29, VEN 3.26, ZAF 3.11
47 (H1) 777777774020771 ▪▪▪▪▪▪▪▪▪▪▪▪▪▪▪▪▪▪▪▪▪▪▪▫▫▫▫▫▫▪▫▫▫▫▪▪▪▪▪▪▪▪▪	71 (3.15)	4.49	EURO-W 20.1, AMER-N 15.36, EURO-S 13.34, AMER-S 12.52, EURO-N 10.37, EURO-E 7.14, ASIA-W 3.98, AFRI-N 3.6	USA 15.04, ITA 8.15, AUT 7.97, BRA 7.27, FXX 6.57, FIN 5.94, CZE 3.73, ESP 3.54, SWE 3.35, PER 3.03
50 (H3) 777777777720771 ▪▪▪▪▪▪▪▪▪▪▪▪▪▪▪▪▪▪▪▪▪▪▪▪▪▪▪▪▪▪▫▪▫▫▫▫▪▪▪▪▪▪▪	166 (7.35)	4.68	AMER-S 17.36, EURO-W 16.77, AMER-N 16.77, EURO-S 11.02, CARI 5.55, EURO-E 5.27, EURO-N 5.21, AFRI-N 4.06, AFRI-S 3.86, AFRI-M 3.61, ASIA-W 3.07	USA 16.74, BRA 7.19, FXX 6.57, AUT 5.8, ITA 5.19, ESP 5.19, PER 4.51, ZAF 3.86, CMR 3.55, CZE 3.49, HTI 3.27
52 (T2) 777777777760731 ▪▪▪▪▪▪▪▪▪▪▪▪▪▪▪▪▪▪▪▪▪▪▪▪▪▪▪▪▪▪▪▪▫▫▫▫▪▪▪▫▪▪▪	43 (1.91)	4.54	EURO-W 19.11, ASIA-E 14.89, EURO-N 13.52, AMER-N 11.72, EURO-S 5.17, ASIA-W 5.17, AFRI-M 5.17, AFRI-E 4.86, EURO-E 4.22, AMER-C 3.38	CHN 11.51, USA 11.3, SWE 9.5, FXX 8.77, BEL 4.75, CMR 4.22, ITA 3.38, JPN 3.17, ETH 3.17
53 (T1) 777777777760771 ▪▪▪▪▪▪▪▪▪▪▪▪▪▪▪▪▪▪▪▪▪▪▪▪▪▪▪▪▪▪▪▪▫▫▫▫▪▪▪▪▪▪▪	346 (15.33)	5.42	EURO-W 15.4, AMER-N 13.27, AMER-S 12.25, EURO-S 9.26, EURO-N 7.36, ASIA-W 7.19, AFRI-S 4.89, AFRI-E 4.57, ASIA-E 4.17, AFRI-N 3.46, EURO-E 3.21, AMER-C 3.18, CARI 3.16	USA 12.99, FXX 7.75, BRA 5.45, ITA 5.25, ZAF 4.78, TUR 3.42, AUT 3.37, CHN 3.04
61 (Cameroon) 777777743760771 ▪▪▪▪▪▪▪▪▪▪▪▪▪▪▪▪▪▪▪▪▪▪▫▫▫▪▪▪▪▪▪▪▫▫▫▫▪▪▪▪▪▪▪	42 (1.86)	4.11	AFRI-M 35.23, AFRI-W 28.57, EURO-W 10.96, AMER-N 7.83, ASIA-W 4.4, EURO-S 3.91	CMR 34.54, NGA 25.54, FXX 8.02, USA 7.83, SAU 4.31
62 (H1) 777777774020731 ▪▪▪▪▪▪▪▪▪▪▪▪▪▪▪▪▪▪▪▪▪▪▪▪▪▫▫▫▫▫▫▪▫▫▫▫▪▪▪▫▪▪▪	48 (2.13)	8.41	AMER-S 35.03, EURO-W 15.41, EURO-S 11.91, AMER-N 11.91, EURO-N 5.25, AFRI-W 4.38, AFRI-E 3.5	COL 32.75, USA 11.91, FXX 11.56, ITA 7.18, GMB 4.2, ESP 3.68, FIN 3.5
482 (BOV_1) 676773777777600 ▪▪▫▪▪▪▪▪▫▪▪▪▪▪▪▫▪▪▪▪▪▪▪▪▪▪▪▪▪▪▪▪▪▪▪▪▪▪▫▫▫▫▫	53 (2.35)	3.19	EURO-S 39.49, EURO-W 35.88, ASIA-N 8.07, EURO-N 5.54, AMER-S 3.61	ESP 34.26, FXX 23.72, RUS 8.07, ITA 5.06, FRA 4.64, SWE 3.31, NLD 3.19, DEU 3.13

* Worldwide distribution is reported for regions with more than 3% of a given SITs as compared to their total number in the SITVIT2 database. The definition of macro-geographical regions and sub-regions (http://unstats.un.org/unsd/methods/m49/m49regin.htm) is according to the United Nations; Regions: AFRI (Africa), AMER (Americas), ASIA (Asia), EURO (Europe), and OCE (Oceania), subdivided in: E (Eastern), M (Middle), C (Central), N (Northern), S (Southern), SE (South-Eastern), and W (Western). Furthermore, CARIB (Caribbean) belongs to Americas, while Oceania is subdivided in 4 sub-regions, AUST (Australasia), MEL (Melanesia), MIC (Micronesia), and POLY (Polynesia). Note that in our classification scheme, Russia has been attributed a new sub-region by itself (Northern Asia) instead of including it among rest of the Eastern Europe. It reflects its geographical localization as well as due to the similarity of specific TB genotypes circulating in Russia (a majority of Beijing genotypes) with those prevalent in Central, Eastern and South-Eastern Asia.

** The 3 letter country codes are according to http://en.wikipedia.org/wiki/ISO_3166-1_alpha-3; countrywide distribution is only shown for SITs with ≥3% of a given SITs as compared to their total number in the SITVIT2 database.

As compared to Table 1 which provides data on all strains, the Spoligoforest in Fig 3 only takes in account number of strains from known patient origin so as to detect phylogeographical specificities. Changes (loss of spacers) are represented by directed edges between nodes, with the arrowheads pointing to descendant spoligotypes. In this representation, the heuristic used selects a single inbound edge with a maximum weight using a Zipf model. Solid black lines link patterns that are very similar, i.e., loss of one spacer only (maximum weigh being 1.0), while dashed lines represent links of weight comprised between 0.5 and 1, and dotted lines a weight less than 0.5. A careful scrutiny of this Spoligoforest tree illustrating the distribution of predominant SITs vs. origin of patients (both at the level of macro-regions as well as countries), corroborates the reported phylogeographical specificity of certain spoligotypes/lineages [11, 12]. For example, taking the example of SIT1/Beijing lineage which is reportedly well spread in patients originating in Asia, Eastern-Europe and former Soviet Republics [9, 10], was also traced back to foreign-born patients originating in the very same countries in our study. Indeed, for SIT1/Beijing cases with known patient origin in our study, exact country of origin was traced back to 25 foreign-born cases, split as: 10 cases originating in Asia (Vietnam, China, Korea, Cambodia, Thailand, and India), 9 cases in Eastern-Europe and former Soviet Republics (Armenia, Azerbaijan, Albania, Chechnya, Moldova, Poland, and Yugoslavia), and 6 from other countries (Afghanistan, Senegal, Maghreb). Similarly, one may also note that SIT61/Cameroon strains [13] were noticeably present in our study among patients from Middle and Western Africa.

**Fig 4:**
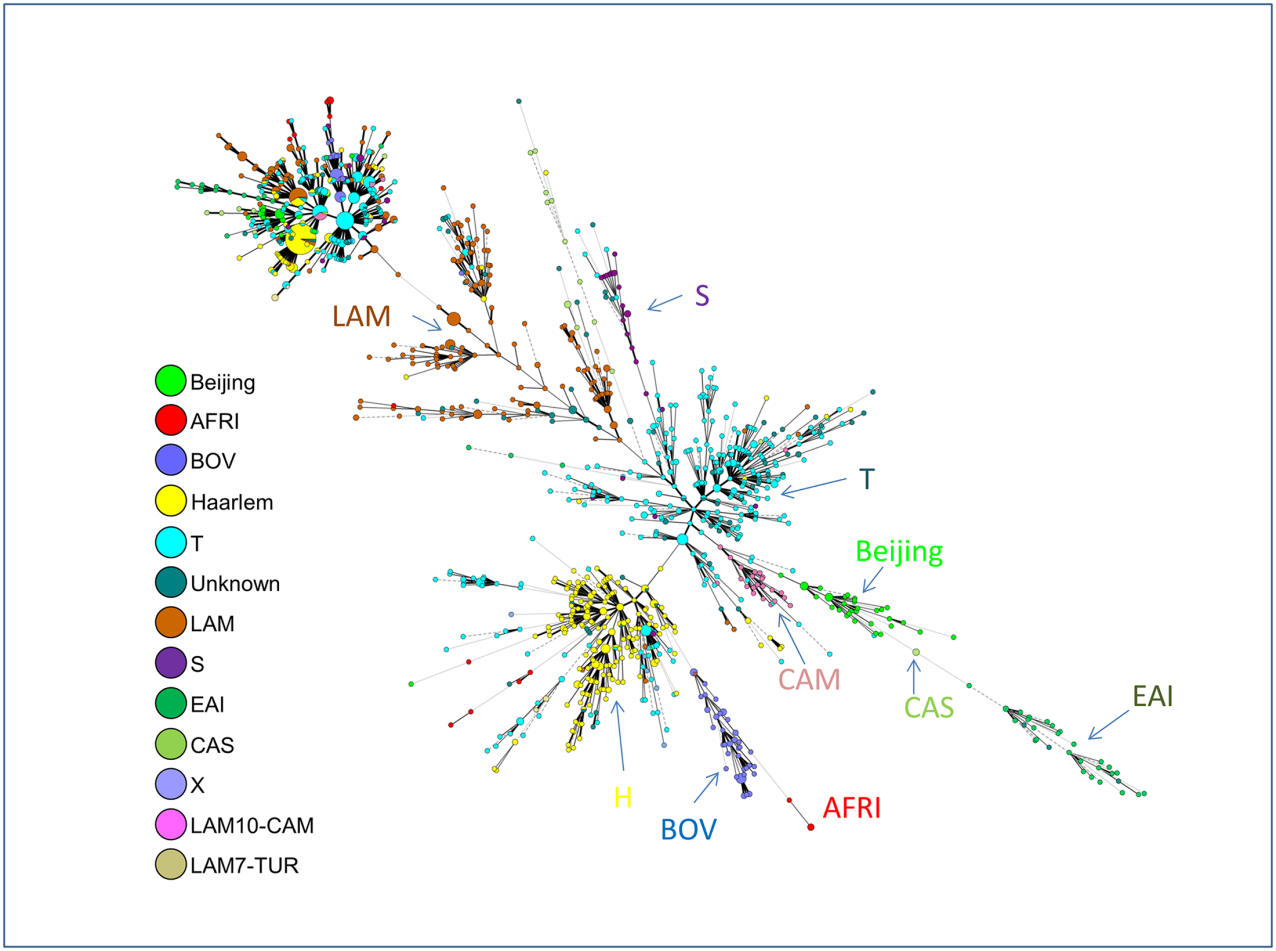
A minimum spanning tree (MST) illustrating evolutionary relationships between all MIRU15-types. Note that the designations for each circle and line in the tree (n = 1698 isolates) are the same as in Fig 2.

When another MST was drawn using available 15-loci MIRU-VNTR data (n = 1698); one could witness a remarkably higher diversity observed as compared to spoligotypes since 15-loci MIRU typing split most of the patterns, notably the patterns belonging to the Euro-American lineage group (Fig 4). Remarkably, despite this higher diversity observed due to a faster molecular clock of 15-loci MIRUs that led to patterns genetically more diversified than spoligotyping—all major lineage groups remained very well organized within their corresponding spoligotyping based lineages, highlighting the phylogeographic specificity of lineages that evolved in close association with their respective human hosts [11, 12, 25]. Interestingly, two LAM sublineages that were recently raised to independent lineage level (LAM10-CAM as the Cameroon lineage [13], and LAM7-TUR as the Turkey lineage [14, 15]), were both well differentiated from LAM lineage in Fig 4.

Phylogenetic trees were also constructed using the MLVA Compare software on combined spoligotype and MIRU data in order to visualize deeper associations between spoligotypes and MIRU-VNTRs when 12-MIT/15-MIT labels were available (a total of 531 strains with 12-MIT labels, and 353 strains with 15-MIT labels were available). The resulting “spoligotype+12-MIT” MST and a circular “spoligotype+15-MIT” UPGMA (Unweighted Pair Group Method with Arithmetic mean) tree are illustrated in S1 and S2 Figs respectively. The color designations of lineages are the same in both Figures, and indicate the phylogenetic lineage to which a specific pattern belongs, and facilitate visualization of most predominant SIT/12-MIT or SIT/15-MIT couples. One may notice the predominance of certain SIT/12-MIT couples: (i) SIT53/12-MIT15 (MIRU pattern: 223325153322), (ii) SIT50/12-MIT42 (MIRU pattern: 225313153323), (iii) SIT50/12-MIT45 (MIRU pattern: 225325153323), (iv) SIT211/12-MIT226 (MIRU pattern: 224326152323), (v) SIT53/12-MIT169 (MIRU pattern: 223225153324), (vi) SIT1/12-MIT86 (MIRU pattern: 223325173433); as well as the following SIT/15-MIT couples: (i) SIT20/15-MIT224 (MIRU pattern: 232531242421312), (ii) SIT53/15-MIT263 (MIRU pattern: 243533333712343), (iii) SIT482/15-MIT219 (MIRU pattern: 223532553500122), (iv) SIT20/15-MIT330 (MIRU pattern: 242531243724312), (v) SIT53/15-MIT322 (MIRU pattern: 233533343522223), and (vi) SIT53/15-MIT380 (MIRU pattern: 283643332632648). It might be worthy of mentioning that most of the isolates were relatively well organized inside the MST with star-like branching in S1 Fig, and distributed homogeneously in the circular UPGMA tree in S2 Fig showing the close genetic relatedness of the strains (despite the fact that some outliers belonging to T- lineage were observed on the tree based on spoligotyping + 15-loci MIRUs).

Based on the global genotyping data generated during the course of this investigation, one obtains a primary rate of clustering by spoligotyping of 77% as compared to 35% by MIRU-VNTRs. However, using the n-1 formula, we obtained a rate of recent transmission of 23.8% in a sub-sample of 974 isolates typed by spoligotyping and 15-loci MIRU-VNTRs.

### Phylogeography, origin of patients, drug resistance and type of disease

**Fig 5:**
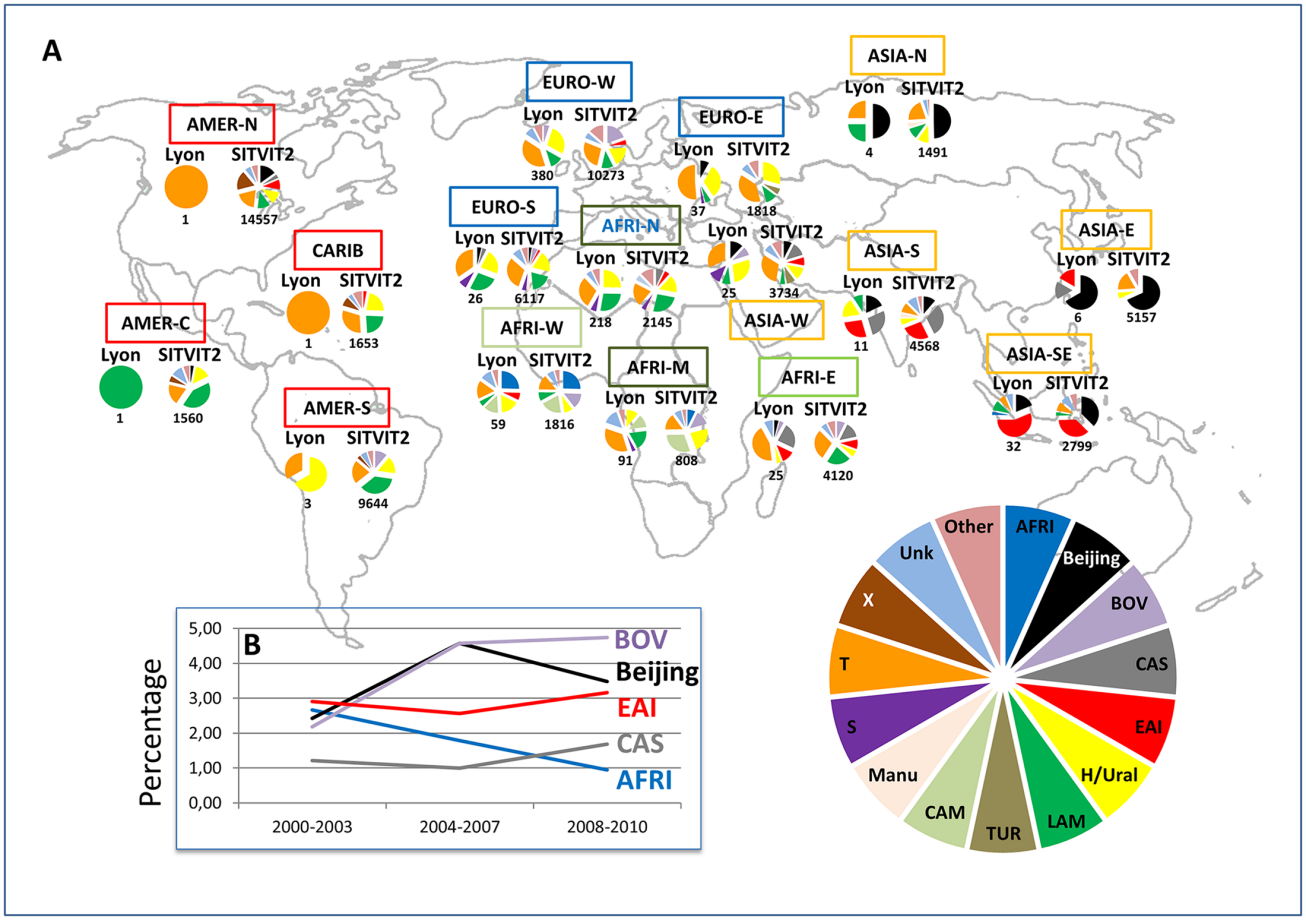
Phylogeographical distribution of major *M*. *tuberculosis* lineages according to patient region of origin. Comparison between all strains isolated in our study (Lyon, n = 2257) from 2000 to 2010, and all strains contained in our genotyping database (SITVIT2, n = 108845 at the time of the study) (**A**); and trends of evolution over time of Principal Genetic Group 1 (PGG1) lineages involved in the study (B). Note that the map shown in Fig 5A was downloaded using the link: https://en.wikipedia.org/wiki/United_Nations_geoscheme#/media/File:United_Nations_geographical_subregions.png under Creative Commons License, and was manually reshaped using GIMP and Paint.NET softwares.

The origin was known for 927/2257 patients: 376 (40.6%) patients were French and 551 (59.4%) were Foreign-born patients. The phylogeographical distribution of major *M*. *tuberculosis* lineages according to patient origin is summarized in Fig 5A, and corroborates our previous findings [26] that a majority of foreign-born patients in low TB incidence countries are infected by the lineages prevailing in their country of origin, particularly if they happen to come from countries where TB is highly endemic.

To have an idea of changes over time in the phylogenetic distribution of the genotypes, we not only considered the distribution of MTBC lineages between French and foreign-born patients (Table 2), but also depicted the evolution trends over time of PGG1 and PGG2/3 lineages involved in our study (Fig 5B and Table 3). A careful analysis of trends of evolution over time of PGG1 vs/ PGG2/3 lineages (n = 2079/2257 or 92.1% of classified strains and 178 isolates with unknown signatures) showed that the proportion of PGG1 lineages increased from 47/412 (11.41%) in 2000–2003 to 130/896 (14.51%) in 2004–2007, and 134/949 (14.12%) in 2008–2010. The corresponding decline in the isolation frequency of evolutionary recent PGG2/3 lineages for the same time period corresponded to: 335/412 (81.31%) in 2000–2003 to 701/896 (78.24%) in 2004–2007, and 732/949 (77.13%) in 2008–2010. A finer analysis of trends of evolution over time of PGG1 sublineages (split in BOV, Beijing, EAI, CAS and AFRI) is illustrated in Fig 5B. One may note that as opposed to the other lineages, the AFRI lineage was constantly decreased over time in our study (representing 2.67% in 2000–2003, 1.79% in 2004–2007, and 0.95% in 2008–2010), and this trend is really different from the evolution trend of the other lineages (with a statistically significant p-value < 0.05, when comparing PGG1 strains; OR = 1.33 95%CI [1.02; 1.75]). On the contrary, one may notice that the curve corresponding to the BOV lineage relatively increased over time (representing 2.18% in 2000–2003, 4.58% in 2004–2007, and 4.75% in 2008–2010). Despite a different of proportion in both EAI and CAS lineages, their evolution trend was very similar (their curves being almost parallel), slightly decreasing from 2000–2003 year group (2.91% vs. 1.21%) to 2004–2007 year group (2.57% vs. 1.00%), and growing in 2008–2010 year group (3.16% vs. 1.69%). Lastly, the number of female patients was particularly high for strains belonging to AFRI, BOV, Cameroon, S, and Turkey lineages (with male/female sex ratios oscillating from 0.4 to 1.0; p-value = 0.009).

**Table 2:** Description of *M*. *tuberculosis* complex biodiversity for patients with known geographical origin (n = 927).

Phylogenetic lineages	Total	Number (%) of French patients	Number (%) of Foreign-born patients
T	311	146 (46.9)	165 (53.1)
LAM	142	45 (31.7)	97 (68.3)
Cameroon / LAM10-CAM	25	5 (20.0)	20 (80.0)
Turkey / LAM7-TUR	1	0 (0.0)	1 (100.0)
Haarlem and Ural	215	106 (49.3)	109 (50.7)
Unknown	65	31 (47.7)	34 (52.3)
EAI	35	3 (8.6)	32 (91.4)
Beijing	36	9 (25.0)	27 (75.0)
S	32	10 (31.3)	22 (68.8)
Africanum	22	2 (9.1)	20 (90.9)
CAS	13	0 (0.0)	13 (100.0)
Bovis	29	19 (65.5)	10 (34.5)
X	1	0 (0.0)	1 (100.0)
**Total**	927	376	551

**Table 3:** Trends of evolution of lineages over time, and distribution of lineages vs. studied parameters.

Lineage	2000–2003 (%)	2004–2007 (%)	2008–2010 (%)	Mean Age (standard deviation) [Min;Max]	Male/Female Sex ratio	Pulmonary (%)	Extra-pulmonary (%)	Pan-susceptible	Drug resistant	MDR
**AFRI**	11 (2.67)	16 (1.79)	9 (0.95)	32.61 yrs. (12.63) [5;61]	19/17 = 1.12	19 (2.19)	8 (2.17)	20 (2.14)	5 (3.01)	0 (0.00)
**Beijing**	10 (2.43)	41 (4.58)	33 (3.48)	38.40 yrs. (17.64) [8;88]	58/25 = 2.32	48 (5.53)	6 (1.63)	37 (3.95)	6 (3.61)	8 (47.06)
**BOV**	9 (2.18)	41 (4.58)	45 (4.74)	44.91 yrs. (28.06) [1;94]	46/49 = 0.94	13 (1.50)	46 (12.47)	4(0.43)	28 (16.87)	0 (0.00)
Cameroon*	12 (2.91)	19 (2.12)	22 (2.32)	32.87 yrs. (14.61) [6;74]	26/26 = 1.00	23 (2.65)	8 (2.17)	25 (2.67)	3 (1.81)	0 (0.00)
**CAS**	5 (1.21)	9 (1.00)	16 (1.69)	35.43 yrs. (18.08) [10;95]	18/12 = 1.50	9 (1.04)	7 (1.90)	12 (1.28)	2 (1.20)	1 (5.88)
**EAI**	12 (2.91)	23 (2.57)	31 (3.27)	47.24 yrs. (18.66) [19;85]	40/26 = 1.54	33 (3.80)	11 (2.98)	41 (4.38)	1 (0.60)	0 (0.00)
**H/Ural**	96 (23.30)	202 (22.54)	216 (22.76)	54.30 yrs. (22.07) [2;109]	284/230 = 1.23	195 (22.47)	83 (22.49)	214 (22.86)	39 (23.49)	1 (5.88)
**LAM**	61 (14.81)	150 (16.74)	149 (15.70)	49.65 yrs. (21.84) [1;94]	222/138 = 1.61	135 (15.55)	65 (17.62)	160 (17.09)	27 (16.27)	3 (17.65)
**S**	18 (4.37)	22 (2.46)	23 (2.42)	52.89 yrs. (21.31) [1;91]	26/37 = 0.70	29 (3.34)	9 (2.44)	28 (2.99)	3 (1.81)	2 (11.76)
**T**	148 (35.92)	300 (33.48)	314 (33.09)	56.90 yrs. (23.72) [1;101]	456/304 = 1.50	299 (34.45)	92 (24.93)	309 (33.01)	47 (28.31)	2 (11.76)
Turkey**	0 (0.00)	4 (0.45)	3 (0.32)	34.29 yrs. (21.13) [19;66]	2/5 = 0.40	5 (0.58)	0 (0.00)	4 (0.43)	0 (0.00)	0 (0.00)
**Unknown**	30 (7.28)	65 (7.25)	83 (8.75)	54.68 yrs. (23.85) [1;95]	102/76 = 1.34	58 (6.68)	32 (8.67)	78 (8.33)	5 (3.01)	0 (0.00)
**X**	0 (0.00)	4 (0.45)	5 (0.53)	51.0 yrs. (23.40) [19;79]	6/3 = 2.00	2 (0.23)	2 (0.54)	4 (0.43)	0 (0.00)	0 (0.00)

* The Cameroon lineage was previously labeled LAM10-CAM in the database.

** The Turkey lineage was previously labeled LAM7-TUR in the database.

In conjunction with the trends of evolution of these specific lineages, we can observe the trends of strains from patients of different origin (subregions) collected over time in Fig 1A and 1B. Interestingly, unlike the difference observed by comparing the PGG1 strains, no statistically significant differences were noted when we compared the different proportions found in AFRI-W, ASIA-E, ASIA-N, ASIA-S, ASIA-SE, EURO-E, ASIA-W, AFRI-E (subregions in which PGG1 strains were more common), regarding the same groups of years (2000–2003, 2004–2007, and 2008–2010; with a p-value>0.13; Fig 1A). Nevertheless, a closer look on the distribution of MTBC lineages between French and foreign-born patients, and trends of evolution over time (between 2000 and 2010) of ancient PGG1 group strains in the Rhône-Alpes Region (Tables 2 and 3, Fig 5B) suggest that transmission might be occurring between French and foreign-born patients. Indeed, ancient PGG1 strains are highly endemic in Africa and Asia while most of the TB in Europe and Americas is caused by evolutionary recent PGG2/3 *M*. *tuberculosis* strains [9–12, 25–28], a fact that strongly argues in favor of probable ongoing transmission between French and foreign-born patients in our study setting. A similar observation can also be made for the PGG2/3 group of Cameroon lineage strains (formerly LAM10-CAM [13]) that are apparently spread between both French and foreign-born patients (Table 2).

Finally, in order to validate the observed trend of PGG1 strains over time, both the conventional univariate analysis and ordinal logistic regression were used to assess the likelihood of differences in evolution of PGG1 vs. PGG2/3 lineages over time as illustrated in Tables 3 and 4. Numbers for PGG1 vs. PGG2/3 group for origin (foreign-born vs. French), sex (female vs. male), disease localization (extrapulmonary vs. pulmonary), drug-susceptibility (susceptible vs. resistant), and interquartile range for age are detailed in S1 File. Considering P values of <0.05 as statistically significant in the analysis shown in Table 4, one can conclude that with the exception of gender, all other parameters studied significantly varied between PGG1 vs PGG2/3 groupings. Lastly, regarding age group distributions vs. lineages (histograms for age-group ranges 1–20, 21–40, 41–60, 61–80, and >80 years can be consulted in S1 File), one observes that age-group 21–40 yrs. was predominant among AFRI, Beijing, Cameroon (LAM10-CAM), CAS, EAI, LAM, and Turkey (LAM7-TUR) lineages, age-group 61–80 yrs. predominated in S and X lineages, whereas all age-groups (including the age group 1–20 yrs.) were almost equally present in BOV lineage. Lastly, Haarlem and T lineage strains grouped patients of all age groups with the exception of group 1–20 yrs.

**Table 4:** Multivariate analysis using PGG1 vs. PGG2/3 as the dependent variables.

PGG1 vs. PGG2/3 *	OR	Standard error	Z score	P-value	[95% CI]
**French**	0.60	0.14	-2.21	0.027	[0.39; 0.95]
**Year** **	1.07	0.04	2.06	0.039	[1.003; 1.14]
**Male Gender**	1.21	0.26	1.11	0.359	[0.81; 1.81]
**Age** **	0.98	0.005	-3.88	<0.001	[0.97; 0.99]
**Pulmonary-TB**	0.54	0.11	-2.95	0.003	[0.36; 0.81]

* Cases belonging to PGG1 group were coded 1, and cases belonging to PGG2/3 group were coded 0; Reference categories considered were foreign-born, female gender, and extrapulmonary cases. Note that data without information on origin, sex, and localization of disease was excluded from this analysis (further details are provided in S1 File).

** Note that variables "Year" and "Age" were taken as continuous variables. This means that they have not to be taken into account in the reference categories list (foreign born, female gender, and extrapulmonary cases). Reference categories are used only for categorical variables.

Demographic data (S3 Table) showed that French patients were significantly older than Foreign-born patients (mean age 58.42 yrs. 95%CI [56.04; 60.80] vs. 42.38 yrs. 95%CI [40.75; 44.0]; p-value<0.001). Most of the foreign-born patients originated from Africa, representing nearly 70% of foreign-born cases: with 39% from Maghreb and 30.1% from Sub Saharan Africa (Fig 1A and 1B). Regarding all patients (including autochthonous patients), the predominant subregion of origin was Western Europe (EURO-W), however, one may notice 2 exceptions for years 2007 and 2010 when the number of patients from North-Africa (AFRI-N or Maghreb) was more important than number of patients from EURO-W. One may also notice that the number of patients from unknown origin was very important, especially from 2005 to 2010. Among all foreign-born patients, the regions of origin were distributed as follows: AFRI-N (n = 218), AFRI-M (n = 91), AFRI-W (n = 59), EURO-E (n = 37), ASIA-SE (n = 32), EURO-S (n = 26), ASIA-W (n = 25), AFRI-E (n = 25), ASIA-S (n = 11), ASIA-E (n = 6), ASIA-N (n = 4), EURO-W without France (n = 4), AMER-S (n = 3), AMER-N (n = 1), CARIB (n = 1), AMER-C (n = 1); moreover, no precise area was available for 7 patients (4 from Africa, 2 from Asia, and 1 from Europe).

In our study, drug resistance status was known for 1119 (49.58%) of all isolates. Among these isolates, 936 (83.65%) were pan-susceptible, 166 (14.83%) were resistant to any drug (but not involving isoniazid and rifampicin simultaneously), and only 17 (1.52%) were multidrug resistant (MDR). As recently shown for Beijing strains [10], drug-resistant and MDR-TB strains were more frequently associated with age group 21–40 years (p-value<0.001), and mostly concerned male patients (representing respectively 66.87% and 76.47% vs. 60.26% for pansusceptible isolates; p-value = 0.0052). Strains belonging to Beijing genotype were more frequently associated with MDR-TB (47.06% of all isolates), followed by LAM lineage (17.65% of LAM strains) as compared to other lineages (p-value<0.001). Last but not least, MDR strains were more common among foreign-born patients (p-value = 0.0068), and the proportion of MDR strains was particularly high among patients from Russia and Eastern European countries (with 50.0% and 8.11% of MDR strains respectively).

Regarding the type of disease, extra-pulmonary TB was more frequent among female patients whereas pulmonary TB predominated among men (p-value<0.001). Our data also corroborated a recent finding showing association of BOVIS and CAS genotypic lineages with extrapulmonary disease [27], since these were significantly well represented in patients affected by extra-pulmonary TB (p-value<0.001). On the other hand, Beijing lineage was found to be linked with pulmonary TB. Besides, pulmonary TB was more frequent in patients of age-group 21–40 years while extra-pulmonary disease was more common in older patients >60 years (p<0.001). Last but not least, the proportion of extra-pulmonary disease was significantly higher among patients originating from ASIA-S, AFRI-E, and AFRI-W, representing respectively 54.55%, 44.0%, and 38.98% of extra-pulmonary TB cases (S3 Table). However, contrary to the study by Lari et al [27], the present study did not show a significant association between the Haarlem genotype and pulmonary disease. Indeed, the Haarlem lineage strains were almost equally distributed among pulmonary (22.47%) and extra-pulmonary TB cases (22.49%) in our study.

If one focuses specifically on spoligotypes circulating among French patients (n = 370), certain strains are apparently specific to countries neighboring the Alps. One may notice presence of SIT134/H3 (n = 7 among French isolates) and SIT62/H1 (n = 8 among French isolates); according to SITVIT2, these patterns are notably present in Italy (n = 5 for SIT134/H3, and n = 41 for SIT62/H1) as well as in Austria and Czech Republic (n = 11 and n = 6, respectively, for SIT62/H1). Remarkably, the spoligoforest tree based on French isolates (S3 Fig) shows several descendant spoligotypes from SIT62 within our dataset, suggesting an ongoing evolution of these strains in Rhône-Alpes region. Likewise, SIT31/T (n = 3 among French isolates) is reportedly present in Czech Republic (n = 20 in SITVIT2), Italy (n = 4) and Austria (n = 1); SIT49/H3 in Finland and countries surrounding the Alps, as well as SIT99/H3, SIT3/H3, and SIT2/H2 in the Alps region. One may speculate that SIT3/H3, and SIT2/H2 that originated in the Alps later spread to Americas/Caribbean through the conquest of the New World since SIT2/H2 is today predominant in Haiti; while SIT3/H3 is well noticeable in countries like Mexico and Brazil [9–12]. On the same lines, SIT760/Unknown (n = 1 in our study), is reportedly present in Italy and Austria (and also in Algeria, a former French colony); and SIT174/Unknown (present in France) led to 2 descendant spoligotypes in the spoligoforest tree among French patients. Similarly, SIT335 (n = 3 in our study) was predominantly found in Czech Republic, Austria, Italy, and France (forming a kind of path of transmission). It might be meaningful to mention that although SIT335 has been classified as LAM9 in SITVITWEB [12], it appeared nearer to H3 sublineage according to the spoligoforest analysis (S3 Fig). Lastly, our study allowed us to highlight the presence of patterns predominantly found/isolated from French patients: e.g. SIT488/H1 (n = 3 isolates from French patients), and SIT713/T1 (n = 3).

## Discussion

Currently, the whole world is on the move. People are moving to work, for leisure, or to flee political crisis. According to United Nations Population Fund (UNFPA), in 2013, 232 million people– 3.2 per cent of the world's population—lived outside their country of origin; the majority of migrants crossed borders in search of better economic and social opportunities while others were forced to flee prevailing crises [29]. One of the most significant changes in migration patterns in the last half century is the increased number of migrating women (who are particularly vulnerable to human trafficking), indeed women now constitute almost half the international migrant population, and in some countries, as much as 70 or 80 per cent [29]. On a more optimistic side, migration is increasingly being perceived as a force that can contribute to development; nevertheless on average the individuals are more pro-trade than pro-immigration across several countries [30].

In such a context, TB genotyping information has become a prominent tool not only to assess risk factors involved in prevailing TB transmission but also to pinpoints genotypic and phylogenetic specificities of prevailing *M*. *tuberculosis* isolates in order to better comprehend intricate relationships between certain lineages and geographic origin of the patients. Unfortunately, such studies from France have remained scarce. We therefore decided to undertake one of the biggest studies of its kind in France by carrying out a profound survey of MTBC strains circulating in Rhône Alpes, an Eastern region of France, during an 11 year period (from 2000 to 2010). The choice of this setting was justified by the fact that not only the Rhône-Alpes region represents one of the higher TB risk French regions [2], it also has a high proportion of immigrants: 5 to 8% of residents of this region were immigrants in 2006 vs. 13% in 2008 [31, 32].

Conjointly led by a team of microbiologists, molecular biologists, and medical researchers, this long-term study aimed to focus on main characteristics of imported and local TB cases in Rhône-Alpes region in France. The results obtained on an impressive number of *M*. *tuberculosis* clinical isolates (n = 2257 strains from as many patients) collected over a period of eleven years (from 2000 to 2010), helped obtain relevant conclusions, briefly; (i) the prominence of imported TB cases leading to a changed genetic background and epidemiologic characteristics of circulating strains, (ii) relatively younger age of foreign-born vs. French patients, (iii) statistically significant association of drug resistance (information available for n = 1119 strains) with age group 21–40 years, (iv) higher proportions of extra-pulmonary TB among female patients vs. pulmonary TB among men, (v) a significantly higher proportion of evolutionary-recent strains (78.33%) belonging to the Euro-American lineages (T, LAM, Haarlem, X, S) as compared to ancient PGG1 (AFRI, Beijing, BOV, CAS, and EAI) strains, and lastly (vi) statistically significant overrepresentation of BOV and CAS lineage strains in patients affected by extra-pulmonary TB.

Our results altogether underlined a subtle change in the genetic diversity landscape of prevailing *M*. *tuberculosis* strains in the Rhône-Alpes region as compared to the expected genotyping profiling based on earlier 2006 version of the SpolDB4 database [11]. Indeed, a majority of the foreign-born patients originated from Africa, followed by Asia that explains for the trends of evolution over time of associated lineages (Table 3). Indeed, higher proportions of foreign-born vs. autochthonous TB cases are more common in low TB incidence countries, implying a probable change in the genetic diversity landscape of TB strains circulating in such countries [3]. In our dataset, AFRI, EAI, LAM, Beijing, S, and CAS lineages were predominantly found in patients from foreign origin while Haarlem, T and BOV lineages were predominantly found in patients of French origin (Tables 3 and 4). The possible reasons for this relative increase in BOV lineage strains over time (2.18% in 2000–2003, 4.58% in 2004–2007, and 4.75% in 2008–2010), such as consumption of raw milk or possible cases of BCGitis (a lymphadenitis which may occur as a complication associated with BCG vaccine, which was mandatory in France until 2007) should be carefully monitored in future investigations.

A careful scrutiny of detailed data provided in S3 Table allows to trace phylogeographical specificity of particular genotypes to countries of origin of patients; one can take for illustration the examples of SIT1332/T2 and SIT152/EAI5, genotypes that were predominantly found among patients originating from Central African Republic and Vietnam, respectively. Other meaningful correlations could also be done amid origin and genotypes of patients, e.g., close similarity between SIT152/EAI5 and SIT139/EAI4-VNM, the lone difference being the presence of spacer 26 in SIT152, an observation which suggests the necessity to fine-tune phylogenetical investigations for reassessment certain EAI sublineages.

Regarding the Beijing lineage, it is worthwhile to underline that a particular MIRU pattern (12-MIT86 pattern corresponding to 223325173433) was predominantly found (n = 8) in our study, that was reported earlier in Singapore (n = 5, when interrogating SITVITWEB database [12]). As regards to couple SIT20/15-MIT224 (LAM1), it is worthy of mention that strains belonging to 15-MIT224 (MIRU pattern: 232531242421312) have been isolated in France and Belgium from patients originating in Equatorial Guinea, Republic of the Congo, Senegal, Angola, Portugal, and Zambia (one individual per country), according to SITVIT2 database [9, 10]. These observations altogether suggest Asian and African origin of SIT1/12-MIT86 (Beijing) and SIT20/15-MIT224 (LAM1) genotypes respectively. Thus conjointly with the observations made in other European or Western countries [3, 26–28], our study suggests that TB transmission is ongoing between foreign-born and autochthonous patients.

Interestingly, regarding *M*. *tuberculosis* isolated in France and recorded in the SITVIT2 database [9, 10], one may notice that with the exception of AFRI (representing 1.6% both in this study and in database), Turkey (0.31% in study vs. 0.53% in SITVIT2), and X lineages (0.4% in study vs. 0.69% in SITVIT2), the proportion of all other lineages mostly involved in foreign-born patients reached a proportion as never recorded before. Indeed, when we compare the proportions found in our study vs. the proportions in SITVIT2 database: Beijing (3.72% in study vs. 1.64% in SITVIT2), LAM10-CAM or the Cameroon lineage (2.35% in study vs. 2.1% in SITVIT2), CAS (1.33% in study vs. 1.26% in SITVIT2), EAI (2.92% in study vs. 1.95%), and S lineage (2.79% in study vs. 1.3% in SITVIT2), the rising significance of imported cases of *M*. *tuberculosis* cannot be overlooked any further. Whether certain lineages may evolve and spread at different levels of swiftness among different host populations remains a matter of debate, nonetheless human populations naïve to a particular strain may be highly receptive to a newly arrived strain as shown recently for *M*. *africanum* in West-Africa; apparently, the proportion of evolutionary ancestral *M*. *africanum* is gradually decreasing in West-Africa, and these strains are being replaced by more successful, evolutionary recent *M*. *tuberculosis*, particularly the LAM10-CAM or Cameroon lineage [13, 33, 34]. In this context, our study also corroborated a slight slope in the proportion of the AFRI lineage as compared to other lineages observing the trends of evolution of PGG1 lineages over time (Fig 5B).

To finish, it might be sensible to nuance the conclusions on foreign vs. French-born patients by considering a limitation of this study regarding the record of the origin of patients. Undeniably, the number of patients from unknown origin became particularly important, especially from 2005 to 2010. The reason for the lack of information concerning the origin (as well as other demographical data) of the patients is linked to a stronger participation of the Biomnis platform to our cohort over the years. Indeed, Biomnis merely subcontracted MTBC cultures from other primary laboratories without systematically collecting the demographical details and origin of patients. Unfortunately, it was almost unmanageable to retrieve it “a posteriori” at the time of the present analysis for majority of samples. Another probable cofounding factor relates to the fact that an important proportion of the French population fosters very close relationship with Maghreb countries. This population, even though French-born, constantly shifts between France and Maghreb rendering it difficult to assess the level of its participation to our cohort, particularly as ethnical data are not allowed to be revealed in France.

A limitation of our study could be the fact that MIRU-VNTRs patterns were not available for all strains. Furthermore, traditional genotyping methods might not be sufficient to exhaustively distinguish all patterns involved in transmission of genetically monomorphic organisms like *M*. *tuberculosis* [35]. Coupled to conventional epidemiological investigations, new genetic markers such as single nucleotide polymorphisms, large sequence polymorphisms, hypervariable MIRU-VNTR loci for subtyping of Beijing isolates, and whole-genome sequencing might soon prove their efficacy in future investigations on molecular epidemiology of tuberculosis [36, 37]. Nevertheless, at the time this study was initiated, spoligotyping coupled to 12 or 15-loci MIRU-VNTRs were the only available and reliable methods for the planned investigation [8, 35, 38–40].

## Conclusions

In recent decades, the decline in the incidence of TB in France took place mainly in patients born in France. With the decrease in the incidence of TB in the Western European countries, patients born abroad (especially from countries where TB is highly endemic), now account for the vast majority of TB cases in France. By studying a significant number of cases during a long time interval, the present study allowed for the first time an improved genotypic tracking of specific *M*. *tuberculosis* isolates circulating in the Rhône-Alpes region in order to allocate the main trends of evolution of TB.

As reported recently, drug resistant-TB including MDR-TB seems to gain ground in France and other low tuberculosis incidence European countries [41], and it seems to be somehow related to immigration [42], like in the present study. These countries have in common a preponderance of the so-called European-American Lineage 4 strains [25]. Undeniably, cross-border and cross-continental movements of people for leisure or work in the last decades to these low incidence countries are increasingly resulting in changing socio-epidemiological scenarios generated by massive immigration from high TB endemic countries [3, 42, 43, 44]. It is therefore imperative to comprehend how tubercle bacilli are spread, identify clones involved in drug-resistant cases, detect newly emerging clones in a setting, and detect risk factors and subpopulations with the highest threat of developing the disease—a task which can be accomplished today within the evolutionary framework of the MTBC—using a variety of molecular typing methods developed over the last two decades [45].

Our work also aims to alert public health organisms and authorities about the impact of immigration on TB epidemiology, since drug resistance is a major issue facing the worldwide struggle against TB. It will be equally important to decipher the proportion of cases due to newly arrived foreign-born patients (i.e. imported cases of active disease) compared to the reactivation of latent TB infection (LTBI) which would require knowing precisely for these patients their time to stay in France before TB is effectively diagnosed. In our opinion, the importance of effectively preventing the reactivation of LTBI in the group of patients from foreign origin should not be neglected. New preventive measures such as screening and treatment of LTBI as well as facilitating access to health services should be urgently taken for this group of patients. To sum up, the occurrence of recent transmission (observed at a rate of 23.8% in a sub-sample of 974 isolates typed by spoligotyping and 15-loci MIRU-VNTRs), as well as cases of shared genotypes between French and foreign-born patients, do emphasize the need for improved public health interventions for all.

## Supporting Information

S1 Fig MST constructed on both spoligotypes and 12-loci MIRUs. Note that only MIRUs with 12-MITs number were considered (n = 531 isolates). The phylogenetic tree connects each genotype based on degree of changes required to go from one allele to another (the distance numbers are visible on each edge). Solid black line denotes one unique change between two patterns, while solid gray line denotes 2 changes, bold dashed line denotes 3 changes, and thin dotted line represents 4 or more changes. The size of the circle is proportional to the total number of isolates. The numbers in each node represent respectively the SIT and the 12-MIT. The color of the circles indicates the phylogenetic lineage to which the specific pattern belongs.
(PNG)

S2 Fig A circular UPGMA (Unweighted Pair Group Method with Arithmetic mean) tree drawn using MLVA Compare based on both spoligotypes and 15-loci MIRUs. Note that only MIRUs with 15-MITs number were considered (n = 353 isolates). The numbers in each node represent respectively the SIT and the 15-MIT. The color designations of lineages are the same as S1 Fig.
(PNG)

S3 Fig Spoligoforest on MTBC isolates from French patients. The tree using the SpolTools software was drawn as a Hierarchical Layout on a total of 376 isolates. Designation of nodes and links are the same as Fig 3.
(PDF)

S1 File Further details in relation to information summarized in Tables 3 and 4. (A). Number of isolates per category in univariate analysis, (B) Histograms of age-group distributions vs. lineages (age ranges 1–20, 21–40, 41–60, 61–80, and >80 years).
(PDF)

S1 Table Listing of orphan strains. Orphan strains (n = 182) and corresponding spoligotyping defined lineages/sublineages recorded among a total of 2257 M. tuberculosis strains from patients residing in the Rhône Alpes region of France.
(PDF)

S2 Table Listing of SITed strains. Description of 433 shared-types (SITs; n = 2075) and corresponding spoligotyping defined lineages starting from a total of 2257 strains isolated in Rhône-Alpes region of France.
(PDF)

S3 Table Full report. Detailed results obtained including demographic, epidemiologic, drug-resistance, and genotyping information on a total of 2257 M. tuberculosis strains isolated in the Rhône-Alpes Region, France.
(PDF)
