# Supplementary figures and images for: Combined Genotypic, Phylogenetic, and Epidemiologic Analyses of Mycobacterium tuberculosis Genetic Diversity in the Rhône Alpes Region, France
Source: PLoS One. 2016 Apr 29;11(4):e0153580. doi: 10.1371/journal.pone.0153580 (PMC4851328; doi:10.1371/journal.pone.0153580)

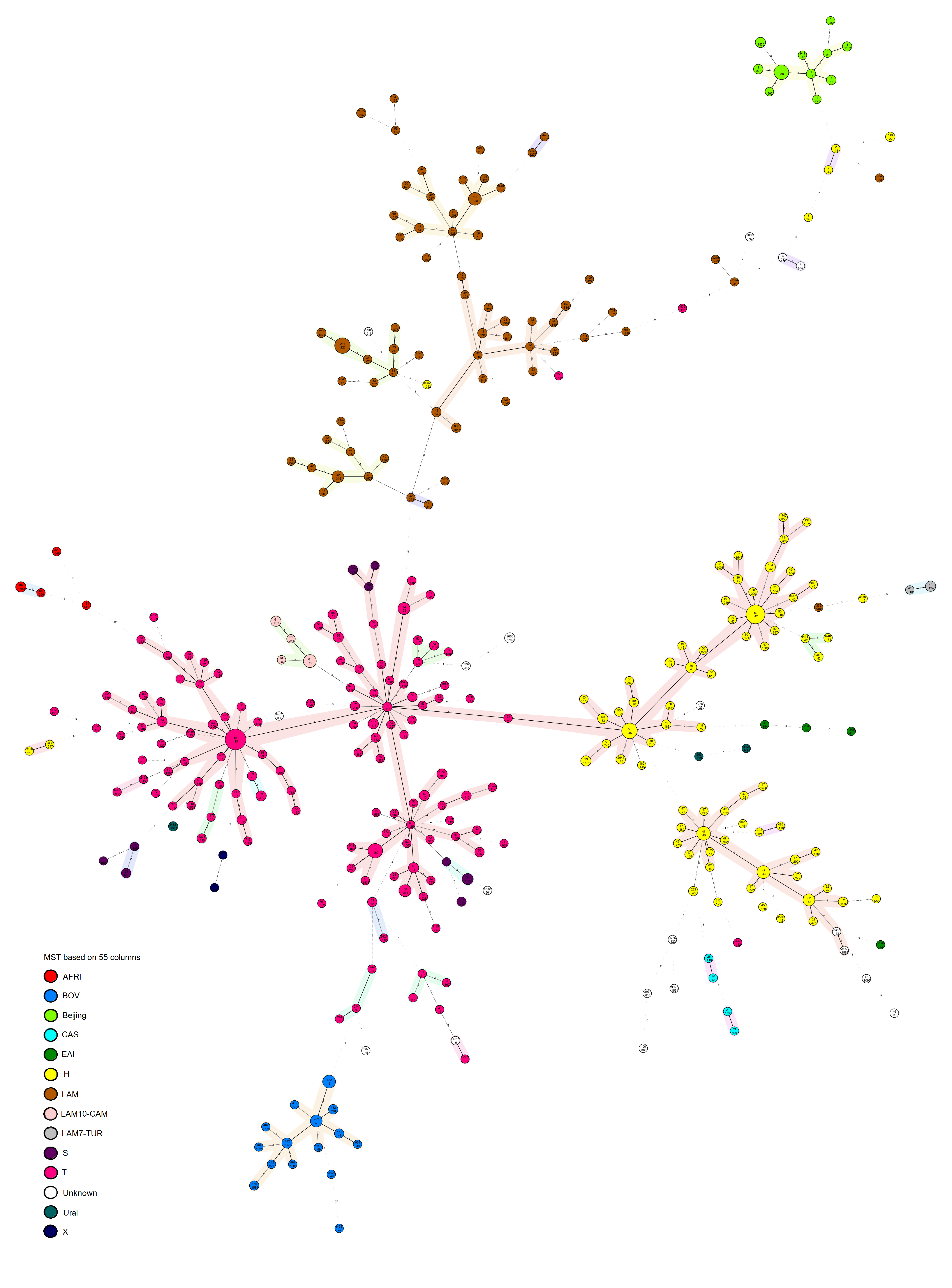

Supplement: S1 Fig — Note that only MIRUs with 12-MITs number were considered (n = 531 isolates). The phylogenetic tree connects each genotype based on degree of changes required to go from one allele to another (the distance numbers are visible on each edge). Solid black line denotes one unique change between two patterns, while solid gray line denotes 2 changes, bold dashed line denotes 3 changes, and thin dotted line represents 4 or more changes. The size of the circle is proportional to the total number of isolates. The numbers in each node represent respectively the SIT and the 12-MIT. The color of the circles indicates the phylogenetic lineage to which the specific pattern belongs. (PNG) [file pone.0153580.s001.png]

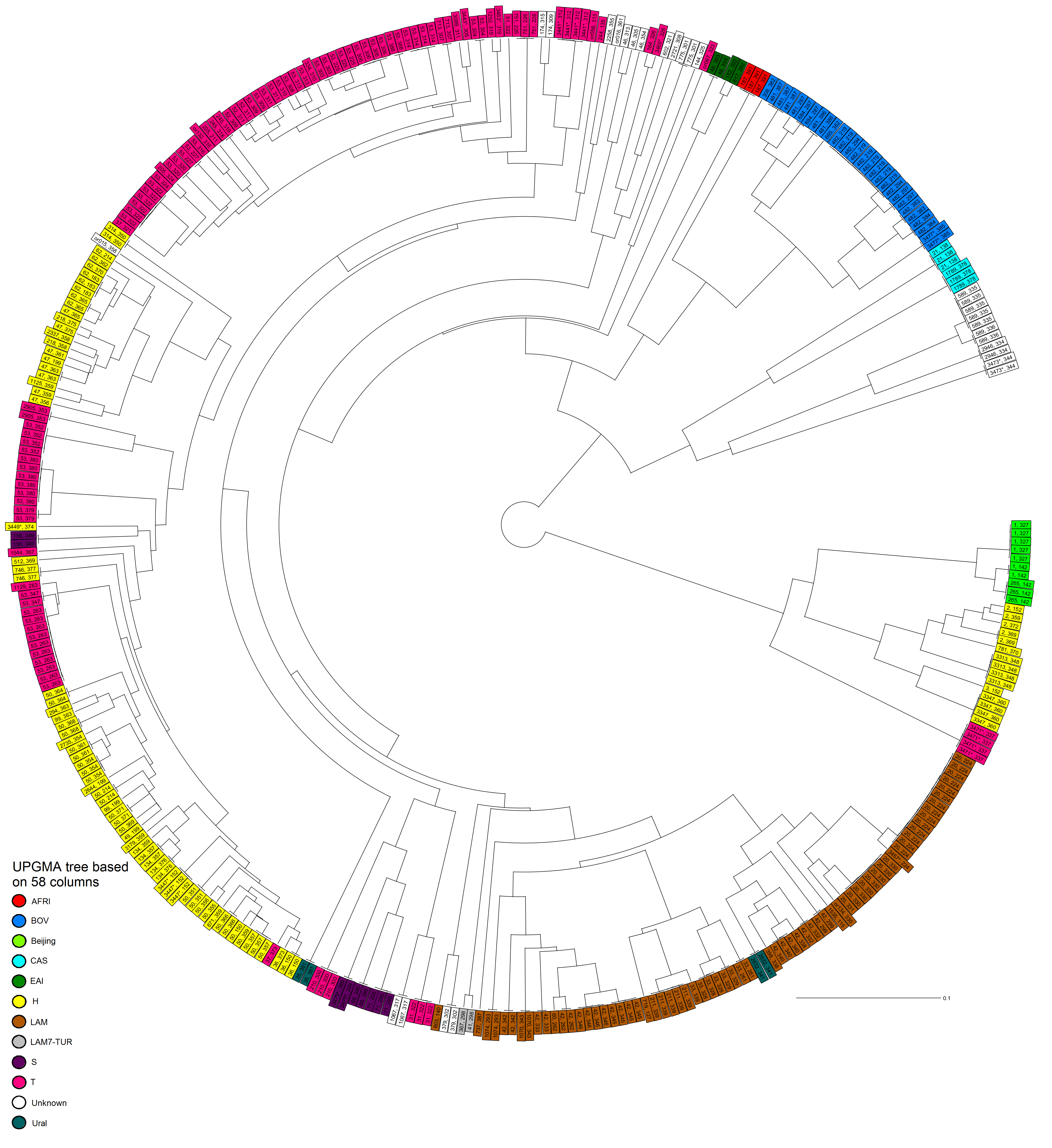

Supplement: S2 Fig — Note that only MIRUs with 15-MITs number were considered (n = 353 isolates). The numbers in each node represent respectively the SIT and the 15-MIT. The color designations of lineages are the same as S1 Fig. (PNG) [file pone.0153580.s002.png]

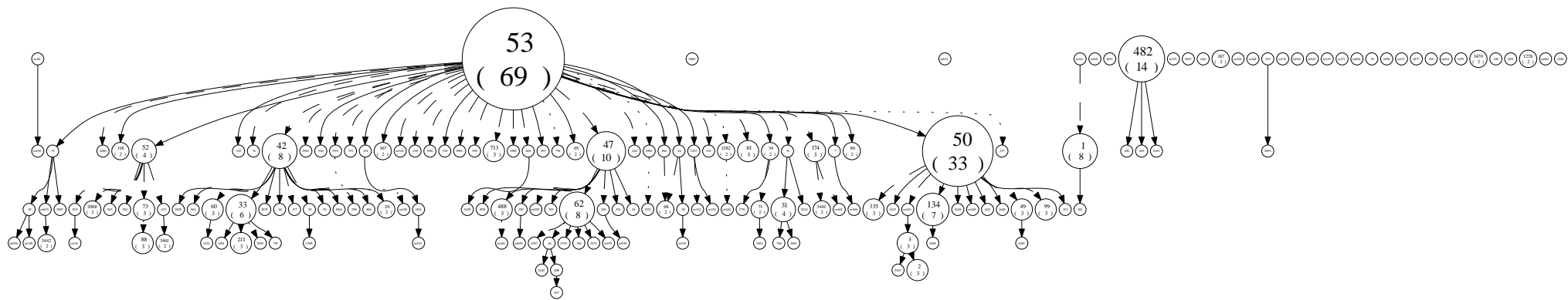

Supplement: S3 Fig — The tree using the SpolTools software was drawn as a Hierarchical Layout on a total of 376 isolates. Designation of nodes and links are the same as Fig 3. (PDF) [file pone.0153580.s003.pdf]
