# Supplementary material for: Combined Genotypic, Phylogenetic, and Epidemiologic Analyses of Mycobacterium tuberculosis Genetic Diversity in the Rhône Alpes Region, France
Source: PLoS One. 2016 Apr 29;11(4):e0153580. doi: 10.1371/journal.pone.0153580 (PMC4851328; doi:10.1371/journal.pone.0153580)
Supplement: S1 File — (A). Number of isolates per category in univariate analysis, (B) Histograms of age-group distributions vs. lineages (age ranges 1–20, 21–40, 41–60, 61–80, and >80 years). (PDF) [file pone.0153580.s004.pdf]

<u>Supplemental File S1</u>: Further details in relation to information summarized in Tables 3 and 4.
(A). Number of isolates per category in univariate analysis, (B) Histograms of age-group distributions vs. lineages (age ranges 1-20, 21-40, 41-60, 61-80, and >80 years).

(A). Details on number of isolates per category:

Parameters	PGG1 (n=311)	PGG2/3 (n=1768)
**Origin**		
Foreign-born	102	415
French	33	312
**Gender**		
Female	129	732
Male	181	1033
**Disease localization**		
Extra-pulmonary	78	259
Pulmonary	122	688
**Drug-susceptibility**		
Pansusceptible	114	744
Any drug-resistant	51	127
**Age**		
Mean	41.30	53.69
Standard deviation	21.63	23.00
Median	37	54
First quartile (25%)	26	33
Third quartile (75%)	55	75

(B). Histograms of age group distributions vs. lineages (age ranges 1-20, 21-40, 41-60, 61-80, and >80 years).

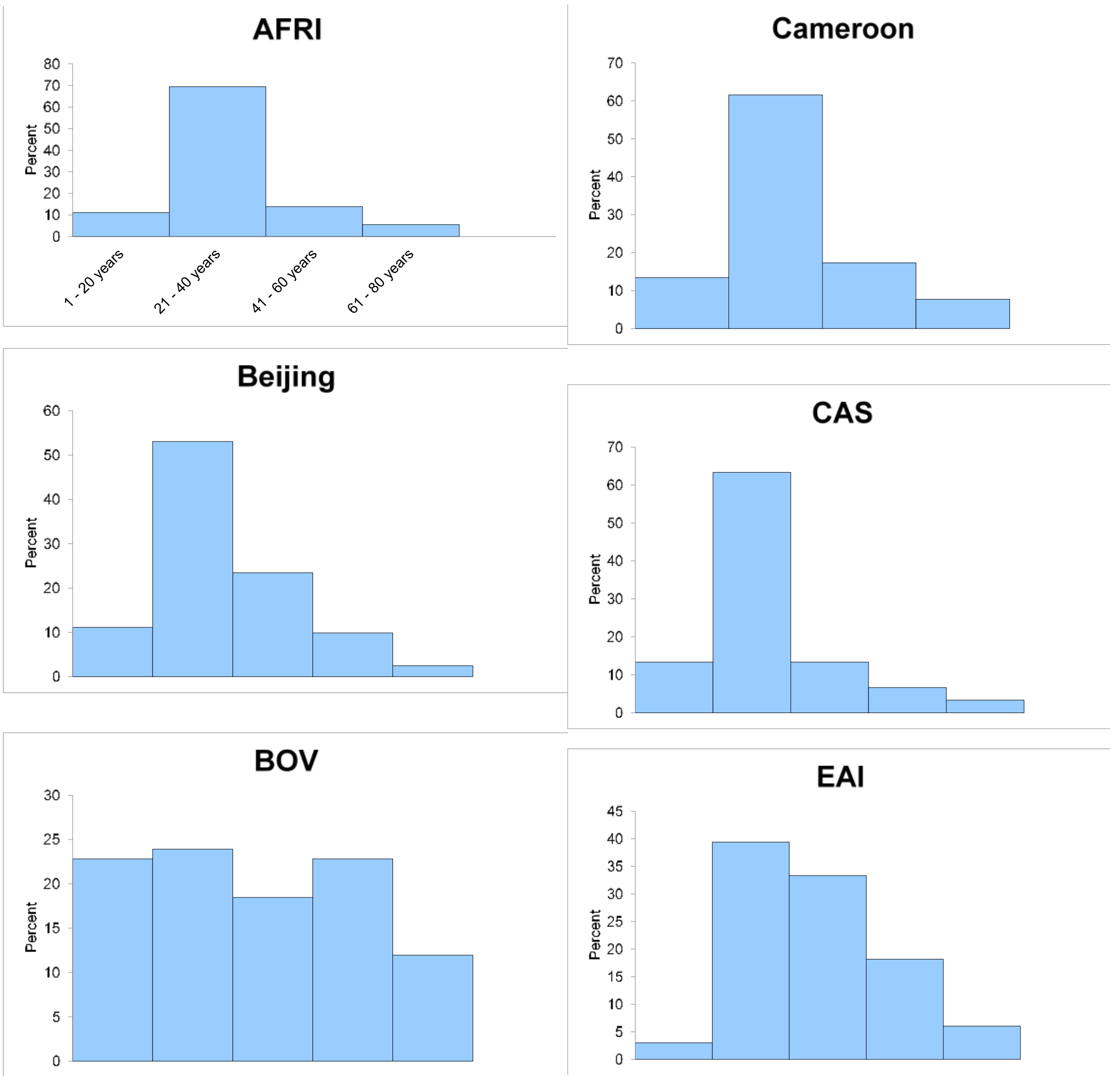

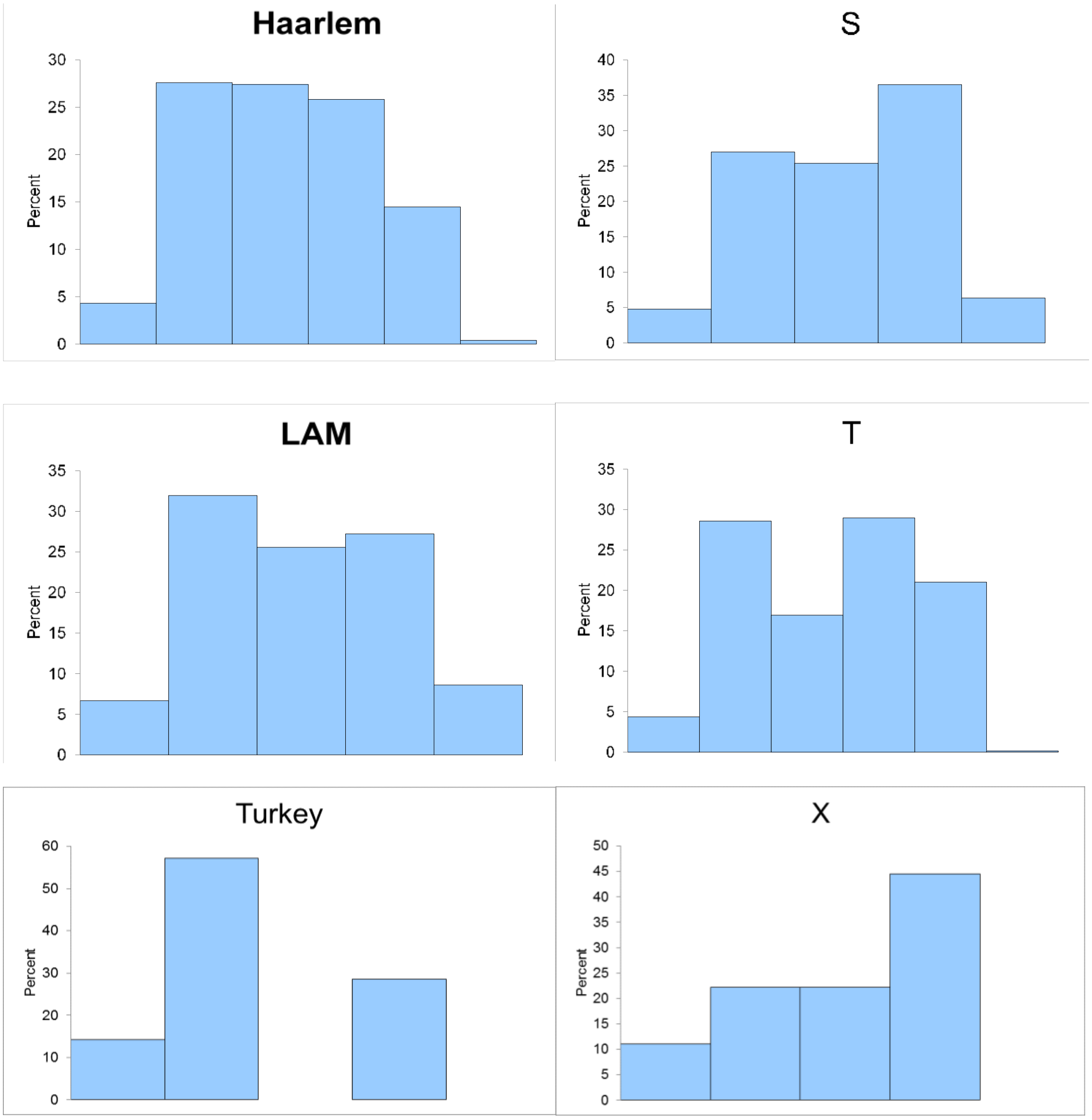
Haarlem
S
LAM
T
Turkey
X
Percent
30
25
20
15
10
5
0
40
35
50
45
60
