# Supplementary material for: Combined Genotypic, Phylogenetic, and Epidemiologic Analyses of Mycobacterium tuberculosis Genetic Diversity in the Rhône Alpes Region, France
Source: PLoS One. 2016 Apr 29;11(4):e0153580. doi: 10.1371/journal.pone.0153580 (PMC4851328; doi:10.1371/journal.pone.0153580)
Supplement: S2 Table — Description of 433 shared-types (SITs; n = 2075) and corresponding spoligotyping defined lineages starting from a total of 2257 strains isolated in Rhône-Alpes region of France. (PDF) [file pone.0153580.s006.pdf]

Supplemental Table S2. Description of 433 shared-types (SITs; n=2075) and corresponding spoligotyping defined lineages starting from a total of 2257 strains isolated in Rhône-Alpes region of France.

SIT*	Spoligotype Description	Octal Number	Nb in study (%)	% in study vs. database	Lineage**	Unique vs. Clustered SIT***
1	□□□□□□□□□□□□□□□□□□□□□□□□□□□□□□□□□□■■■■■■■■■	000000000003771	72 (3.19)	0.73	Beijing	Clustered
2	□□□□□□□□□□□□□□□□□□□□□□□□■□□□□□□□■□□□□■■■■■■■	000000004020771	11 (0.49)	2.8	H2	Clustered
3	□□□□□□□□□□□□□□□□□□□□□□□□■■■■■■□■□□□□■■■■■■■	000000007720771	7 (0.31)	6.67	H3	Clustered
4	□□□□□□□□□□□□□□□□□□□□□□□□■■■■■■■■□□□□■■■■■■■	000000007760771	9 (0.4)	2.55	Unknown	Clustered
5	□□□□□□□□□■■□■■■■■■■■■■■■■■■■■■■■□□□□■■■■■■■	000677777760771	1 (0.04)	3.33	T1	Unique
7	□■■■■■■■■■■■■■■■■■■■■■■■■■■■■■■■□□□□■■■■■■■	377777777760771	5 (0.22)	6.49	T1	Clustered
11	■□□■■■■■■■■■■■■■■■■■■■■■■■■■■□□□□■□■■□□□■■■■	477777777413071	3 (0.13)	0.53	EAI3-IND	Clustered
17	■■□■■■■■■■■■□■■■■■■■■□□□□■■■■■■■■□□□□■■■■■■■	677737607760771	3 (0.13)	0.46	LAM2	Clustered
19	■■□■■■■■■■■■■■■■■■■□□■■■■■■■■□□□□■□■■■■■■■■■	677777477413771	4 (0.18)	0.46	EAI2-Manila	Clustered
20	■■□■■■■■■■■■■■■■■■■■■□□□□■■■■■■■■□□□□■■■■■■■	677777607760771	31 (1.37)	3.83	LAM1	Clustered
21	■■■□□□□■■□■■■■■■■■■■■□□□□□□□□□□□□□□■■■■■■■■	703377400001771	7 (0.31)	1.85	CAS1-Kili	Clustered
25	■■■□□□□■■■■■■■■■■■■■■■□□□□□□□□□□□■■□□■■■■■	703777740003171	4 (0.18)	0.76	CAS1-Delhi	Clustered
26	■■■□□□□■■■■■■■■■■■■■■■□□□□□□□□□□□■■■■■■■■■	703777740003771	8 (0.35)	0.6	CAS1-Delhi	Clustered
29	■■■■■□□□□□□□□□■■□□□□□□□□□□□□□□□□□□□■■■■■	760001400000171	3 (0.13)	7.32	LAM	Clustered
31	■■■■■■■□□□□□□■■■■■■■■■■■■■■■■■■■□□□□■■■□□□□	774037777760700	14 (0.62)	18.18	T	Clustered
32	■■■■■■■■□□□□□□□□□□□□□□□□□□□□□□□□□□□□■■■■■	776000000000171	1 (0.04)	0.96	Unknown	Unique
33	■■■■■■■■□□□■■■■■■■■■■□□□□■■■■■■■■□□□□■■■■■■■	776177607760771	27 (1.2)	2.41	LAM3	Clustered
34	■■■■■■■■□□■■■■■■■■■■■■■■■■■■■■■■□□□□■■■■■■■	776377777760771	23 (1.02)	2.77	S	Clustered
35	■■■■■■■■■■■■□■■■■■■■■■■■■■■■■■■□□□■□□□□■■■■■■■	777737777420771	4 (0.18)	3.01	Ural-1	Clustered
36	■■■■■■■■■■■■□■■■■■■■■■■■■■■■■■□■□□□□■■■■■■■	777737777720771	14 (0.62)	10.94	H3	Clustered
37	■■■■■■■■■■■■□■■■■■■■■■■■■■■■■■■■□□□□■■■■■■■	777737777760771	14 (0.62)	3.07	T3	Clustered
39	■■■■■■■■■■■■■■■■■■□■■■■□□■■■■■■■■□□□□■□□■■■■	777777347760471	5 (0.22)	3.57	T4-CEU1	Clustered
40	■■■■■■■■■■■■■■■■■■□■■■■■■■■■■■■■□□□□■■■■■■■	777777377760771	3 (0.13)	1.95	T4	Clustered
41	■■■■■■■■■■■■■■■■■■■■■□□□□□■□□■■■■■■■□□□□■■■■■■■	777777404760771	6 (0.27)	1.28	LAM7-TUR	Clustered
42	■■■■■■■■■■■■■■■■■■■■□□□□■■■■■■■■□□□□■■■■■■■	777777607760771	125 (5.54)	3.89	LAM9	Clustered
44	■■■■■■■■■■■■■■■■■■■■■■□■■■■■■■■■■□□□□■■■■■■■	777777757760771	10 (0.44)	4.83	T5	Clustered
45	■■■■■■■■■■■■■■■■■■■■■■■□■□□□□□□■□□□□■■■■■■■	777777764020771	1 (0.04)	0.94	H1	Unique
46	■■■■■■■■■■■■■■■■■■■■■■■■□□□□□□□□□□□□□□□□□□□	777777770000000	11 (0.49)	5.5	Unknown	Clustered
47	■■■■■■■■■■■■■■■■■■■■■■■■■□□□□□□■□□□□■■■■■■■	777777774020771	71 (3.15)	4.83	H1	Clustered
48	■■■■■■■■■■■■■■■■■■■■■■■■■■■■□□□□■□■■■■■■□■■■	777777777413731	6 (0.27)	1.4	EAI1-SOM	Clustered
49	■■■■■■■■■■■■■■■■■■■■■■■■■■■■■■□■□□□□■■■□■■■	777777777720731	14 (0.62)	8.14	H3	Clustered
50	■■■■■■■■■■■■■■■■■■■■■■■■■■■■■■□■□□□□■■■■■■■	777777777720771	166 (7.35)	5.04	H3	Clustered
51	■■■■■■■■■■■■■■■■■■■■■■■■■■■■■■■■□□□□■■■□□□□	777777777760700	9 (0.4)	3.24	T1	Clustered
52	■■■■■■■■■■■■■■■■■■■■■■■■■■■■■■■■□□□□■■■□■■■	777777777760731	43 (1.91)	4.8	T2	Clustered
53	■■■■■■■■■■■■■■■■■■■■■■■■■■■■■■■■□□□□■■■■■■■	777777777760771	346 (15.33)	5.72	T1	Clustered
57	■■■■■■■■■■■■■■■■■■□□■■■□□□■■■■■■■■□□□□■■■■■■■	777777143760771	1 (0.04)	20	LAM10-CAM	Unique
58	■■■■■■■■■■■■■■■■■■■□■■□■■■■■■■■■■□□□□■■■■■■■	777777557760771	9 (0.4)	5.2	T5-Madrid2	Clustered
59	■■■■■■■■■■■■■■■■■■■■□□□□■■□□□□■■□□□□■■■■■■■	777777606060771	1 (0.04)	0.22	LAM11-ZWE	Unique
60	■■■■■■■■■■■■■■■■■■■■□□□□■■■■■■■■□□□□■■■□■■■	777777607760731	14 (0.62)	3.41	LAM4	Clustered
61	■■■■■■■■■■■■■■■■■■■■■■□□□■■■■■■■■■□□□□■■■■■■■	777777743760771	42 (1.86)	5.08	LAM10-CAM	Clustered
62	■■■■■■■■■■■■■■■■■■■■■■■■■□□□□□□■□□□□■■■□■■■	777777774020731	48 (2.13)	8.78	H1	Clustered
64	■■■■■■■■■■■■■■■■■■■■□□□□■■■■□■■■■□□□□■■■■■■■	777777607560771	11 (0.49)	3.07	LAM6	Clustered
65	■■■■■■■■■■■■■■■■■■■■■■■■■■■■■■■■□□□□■□□■■■■	777777777760471	4 (0.18)	3.81	T1	Clustered
68	■■■■■■■□■■■■■■■■■■■■■■□■■■■■■■■■■□□□□■■■■■■■	775777757760771	5 (0.22)	6.58	T5	Clustered
71	■■■■■■■■□□■■□■■■■■■■■■■■■■■■■■■■■□□□□■■■■■■■	776337777760771	8 (0.35)	9.41	S	Clustered
73	■■■■■■■■■■■■□■■■■■■■■■■■■■■■■■■■□□□□■■■□■■■	777737777760731	11 (0.49)	4.37	T	Clustered
74	■■■■■■■■■■■■■■■■■■■■□□□□□□□□■■■■□□□□■■■■■■■	777777600160771	1 (0.04)	6.25	Unknown	Unique
78	■■■■■■■■■■■■■■■■■■■■■■■■■■■■■■■■□□□□■■■□□■■	777777777760711	2 (0.09)	3.03	T	Clustered
81	■■■■■■■■■□■■■■■■■■■■□□□□■■■■■■■■□□□□■■■■■■■	777377607760771	1 (0.04)	2.78	LAM9	Unique
86	■■■■■■■■■■■■■■■■■■■■■□■■■■■■■■■■■□□□□■■■■■■■	777777737760771	7 (0.31)	7.69	T1	Clustered
88	■■■■■■■■□□■■□■■■■■■■■■■■■■■■■■■■■□□□□■■■□■■■	776337777760731	8 (0.35)	53.33	S	Clustered
89	■■□■■■■□□□□□□□□□□□□□□□□□□■■■■□□□□■□■■■■■■■■■	674000003413771	1 (0.04)	0.97	EAI2-nonthaburi	Unique
91	■■■□□□□□□□□□□■■■■□■■■■■■■■■■■■■■■□□□□■■■■■■■	700036777760771	1 (0.04)	0.4	X3	Unique
92	■■■□□□□□□□□□■■■■■□■■■■■■■■■■■■■■■□□□□■■■■■■■	700076777760771	3 (0.13)	0.7	X3	Clustered
93	■■■■■■■■■■■■□■■■■■■■□□□□■■■■■■■■□□□□■■■■■■■	777737607760771	10 (0.44)	2.83	LAM5	Clustered
95	■■■■■■■■■■■■■■■■■■■■□□□□■■■■□■■■■□□□□■■■□■■■	777777607560731	1 (0.04)	2.22	LAM6	Unique
99	■■■■□■■■■■■■■■■■■■■■■■■■■■■■■■□■□□□□■■■■■■■	757777777720771	7 (0.31)	9.33	H3	Clustered
101	■■■■■■■□□□□□■■■■■■■■□□□□■■□■■■■■■□■■■□□□■■■■	774077606767071	2 (0.09)	8.7	AFRI_2	Clustered
106	■■■■■■■■□□□■■■■■■■■□□□□□□□□□□□□□□□□□□□■■■■■	776177400000171	2 (0.09)	1.53	Unknown	Clustered
107	□□□□■■■■□□■■■■■■■■■■■■■■■■■■■■■■■□□□□■■■■■■■	036377777760771	2 (0.09)	28.57	S	Clustered
109	■□□■■■■■■■■■■■■■■■□■■■■■■■■□□□□■□■■■■■■■■■	477777377413771	6 (0.27)	5.66	EAI8-MDG	Clustered
112	■■■■■■■■■■■■□■■■■■■■■■■■□□□■■■■■■□□□□■■■■■■■	777737761760771	1 (0.04)	16.67	T	Unique
118	■■■■■■■■■■■■■■□■■■■■■■■■■■■■■■■■□□□□■■■■■■■	777767777760771	3 (0.13)	2.03	T1	Clustered
119	■■■■■■■■■■■■■■■■■□■■■■■■■■■■■■■■□□□□■■■■■■■	777776777760771	1 (0.04)	0.09	X1	Unique
120	■■■■■■■■■■■■■■■■■■■□■■■■■■■■■■■■■□□□□■■■■■■■	777777577760771	2 (0.09)	22.22	T1	Clustered
121	■■■■■■■■■■■■■■■■■■■■■■■■■□■■■■■□■□□□□■■■■■■■	777777775720771	2 (0.09)	4.08	H3	Clustered

124	■■■■■■■■■■■■■■■■■■■■■■■■■■■■■■□□□□□□■■■■■■■	777777777700771	1 (0.04)	1.56	Unknown	Unique
125	□□□□□□□□□□□□□□□□□□□□□□□□■■■■■■■■□□□□■■■□■■■	000000007760731	2 (0.09)	1.83	LAM3	Clustered
128	■■□□■■■■■■■■■■■■□■■■■■■■■■■■■■■■■□□□□■■■□■■□	637775777760730	5 (0.22)	16.13	T2-uganda	Clustered
129	■■■□□□□□□■■■■■■■■■■■■■□□■■■■□□□□■□■■■■■■■■■	700777747413771	1 (0.04)	2.13	EAI6-BGD1	Unique
130	■■■■■■■■□□□■■■■■■■■□□□□■■■■■■■■□□□□■■■□■■■	776177607760731	2 (0.09)	1.87	LAM3	Clustered
134	■■■■■■■■■■■■■■■■■■■■■■■■■■■■■■□■□□□□■■□□■■■	777777777720631	19 (0.84)	36.54	H3	Clustered
136	■■■■■■■■■■■□□□□□■■■□□□□□■□■■■■■■■□□□□■□□■■■■	777603405760471	2 (0.09)	4.55	LAM5	Clustered
137	■■■■■■■■■■■■■■■■■□■■■■■■■■■■■■■■■□□□□■■□□□□■	777776777760601	2 (0.09)	0.2	X2	Clustered
143	■■■■□■■■■■□□□□□□□□□□□□□□□■□□□□□□□■□□□□■■■■■■■	757400004020771	4 (0.18)	18.18	H1	Clustered
144	■■■■■■□□□□□□□□□□□□□□□□□□□■■■■■■■□□□□■■■■■■■	770000003760771	4 (0.18)	7.14	Unknown	Clustered
149	■■■■■■■■■□□□□□□□□□□■■■■■■■■■■■■■□□□□■■■■■■■	777000377760771	1 (0.04)	0.22	T3-ETH	Unique
152	■■■■■■■■■■■■■■■■■■■■■■■■■■□■□□□□■□■■■■■■■	777777776413771	5 (0.22)	11.63	EAI5	Clustered
153	■■■■□■■■■■■■■■■■■■■■■■■■■■■■■■■□□□□■■■□■■■	757777777760731	3 (0.13)	4.05	T2	Clustered
154	■■■■□■■■■■■■■■■■■■■■■■■■■■■■■■■□□□□■■■■■■■	757777777760771	6 (0.27)	5.41	T1	Clustered
156	■■■■■■■■□□□■■■■■■■■■■■■■■■■■■■■□□□□■■■■■■■	776177777760771	3 (0.13)	6	S	Clustered
162	■■■■■■■■■■■■■■■■■■■■□□□□■■■■■■■■□□□□■■□■■■■	777777607760671	1 (0.04)	3.33	LAM9	Unique
163	■■■■■■■■■■■■■■■■■■■■□□□□■■■■■■■■□□□□■■■□□□□	777777607760700	1 (0.04)	3.45	LAM4	Unique
164	■■■■■■■■■■■■■■■■■■■■■■■□□□□□□□■■□□□□■■■■■■■	777777760060771	2 (0.09)	4.44	T1	Clustered
167	■■■■■■■■■■■■■■■■■■■■■■■■■■■■■□■■□□□□■■■■■■■	777777777660771	8 (0.35)	11.11	T1	Clustered
172	■■■■■■■■■■■■■■■■■■■■■■■■■■■■■■■□□□□□■■■■■■■	777777777740771	1 (0.04)	1.82	T1	Unique
174	■■■■■■■■■■■■■■■■■■□□□□■■■■■■■■■■□□□□■■■■■■■	777777037760771	7 (0.31)	31.82	Unknown	Clustered
175	■■■■■■■■■■■■■■■■■■■■□■■■■■■■■■■■□□□□■■■□■■■	777777677760731	1 (0.04)	4.55	T2	Unique
177	□■■■■■■■■■■■■■■■■■■■□□□□■■■■■■■■□□□□■■■■■■■	377777607760771	8 (0.35)	7.84	LAM9	Clustered
180	■■□■■■■■■■■■■■■■■■■■■■■■■■■■■■□■□□□□■■■■■■■	677777777720771	4 (0.18)	6.78	H3	Clustered
181	■■■■■■□□□■■■■■■■■■■■■■■■■■■■■■■■■■■■■□■■■■	770777777777671	10 (0.44)	3.66	AFRI_1	Clustered
183	■■■■■■■■■■■■■■■■■■□■■■■■■■■■■■□■□□□□■■■■■■■	777777377720771	1 (0.04)	1.85	H3	Unique
187	■■■■■■□□□■■■■■■■■■■■■■■■■■■■■■■□■■■■■□■■■■	770777777767671	10 (0.44)	10.31	AFRI_1	Clustered
190	□□□□□□□□□□□□□□□□□□□□□□□□□□□□□□□□□□■■■■■■□■■■	000000000003731	2 (0.09)	1.1	Beijing	Clustered
195	■■□■■■■■■■■■■■■■■■■■□□□□■■■■■□■■□□□□■■■■■■■	677777607660771	1 (0.04)	6.67	LAM1	Unique
196	■■□■■■■■■■■■■■■■■■■■■■■■■■■■■■■■□□□□■■■■■■■	677777777760771	1 (0.04)	1.3	T1	Unique
203	■■■□□□□■■■■■■■■■■■■■■■□□□□□□□□□□□■■■■■■■	703777740001771	1 (0.04)	5.56	CAS	Unique
205	■■■□■■■■■■■■■■■■■■■■■■■■■■■■■■■□□□□■■■■■■■	737777777760771	6 (0.27)	11.54	T1	Clustered
207	■■■■■□■■■■■■■■■■■■■■■■■■■■■■□■□□□□■■■■■■■	767777777720771	1 (0.04)	3.23	H3	Unique
209	■■■■■■■■□□□□□□■■■■■■□□□□■■■■■■■■□□□□■■■■■■■	776017607760771	1 (0.04)	2.44	LAM12-Madrid1	Unique
210	■■■■■■■■□□□□□■■■■■■■■■■■■■■■■■■□□□□■■■■■■■	776037777760771	3 (0.13)	2.34	T1	Clustered
211	■■■■■■■■□□□■□■■■■■■■□□□□■■■■■■■■□□□□■■■■■■■	776137607760771	18 (0.8)	18.18	LAM3	Clustered
218	■■■■■■■■■■■■□■■■■■■■■■■□□□□□□■□□□□■■■■■■■	777737774020771	3 (0.13)	8.33	H1	Clustered
234	■■■■■■■■■■■■■■■■■■■■■■■■■■■■□□□□■□■■□■■■■■■	777777777413371	1 (0.04)	4.76	EAI5	Unique
236	■■■■■■■■■■■■■■■■■■■■■■■■■■■■□□□□■□■■■■■■■■■	777777777413771	5 (0.22)	3.18	EAI5	Clustered
237	■■■■■■■■■■■■■■■■■■■■■■■■■■■■■■□□□□□□□□□□□□□	777777777700000	3 (0.13)	2.83	Unknown	Clustered
238	■■■■■■■■■■■■■■■■■■■■■■■■■■■■■■□□□□□□□□■■■■■	777777777700171	3 (0.13)	17.65	Unknown	Clustered
241	■■■■■■■■■■■■■■■■■■■■■■■■■■■■■■■■□□□□■□□□□■■	777777777760411	3 (0.13)	13.04	T1	Clustered
243	■■■■■■■■■■■■■■■■■■■■■■■■■■■■■■■■□□□□■■□□□□□	777777777760600	1 (0.04)	4.55	T1	Unique
244	■■■■■■■■■■■■■■■■■■■■■■■■■■■■■■■■□□□□■■□□□□■	777777777760601	9 (0.4)	7.83	T1	Clustered
245	■■■■■■■■■■■■■■■■■■■■■■■■■■■■■■■■□□□□■■□■■■■	777777777760671	2 (0.09)	10	T1	Clustered
253	■■■■■■■■■■■■■■■■■■■■□■■□□■■■■■■■□□□□■■■■■■■	777777663760771	1 (0.04)	3.23	T1	Unique
262	■■■■■■■□□■■■■■■■■■■■■■■■■■□□□■□□□□■■■■■■■	774777777420771	3 (0.13)	1.69	Ural-1	Clustered
265	□□□□□□□□□□□□□□□□□□□□□□□□□□□□□□□□□□■■□■■■■■■	000000000003371	4 (0.18)	4.94	Beijing	Clustered
269	□□□□□□□□□□□□□□□□□□□□□□□□□□□□□□□□□□□□■■■■■■■	000000000000771	1 (0.04)	1.14	Beijing	Unique
273	■■■■■■■■■■■□□□■■■■■■□□□□■■■■■■■■□□□□■■■■■■■	777617607760771	1 (0.04)	16.67	LAM5	Unique
276	■■■■■■■■■■■■■■■■■■■■■■■■□□□□□□■■□□□□■■■■■■■	777777770060771	1 (0.04)	2.78	T1	Unique
277	■■■■■■■■■■■■■■■■■■■■■■■■■■■■□■■■□□□□■■■□□□□	777777777560700	2 (0.09)	22.22	T1	Clustered
281	■■■■■■■■■■■■■□■■■■■■■■■■■■■■■■■□□□□■■■■■■■	777757777760771	2 (0.09)	6.9	T1	Clustered
283	■■■■■■■■■■■■■■■■■■■■■□□□■□□□□□□■□□□□■■■■■■■	777777704020771	4 (0.18)	6.56	H1	Clustered
284	□□□□■■■■■■■■□□■■■■■■■■■■■■■■■■■□□□□■■■■■■■	037637777760771	6 (0.27)	3.75	T1	Clustered
291	■■■■■■■■■■■■■■■■■■■■□■■■■■■■■■■■□□□□■■■■■■■	777777677760771	1 (0.04)	1.16	T1	Unique
293	■■■■■■■■■■■■■■■■■■■■■■■■■■■■■■□■□□□□■■■■□■■	777777777720751	1 (0.04)	7.69	H3	Unique
294	■□■■■■■■■■■■■■■■■■■■■■■■■■■■■■□■□□□□■■■■■■■	577777777720771	1 (0.04)	3.7	H3	Unique
306	■■■■■■■■■■■■■■■■■■■■□□□□□□■■□■■■□□□□■■■■■■■	777777601560771	1 (0.04)	4.35	T1	Unique
308	■■□■■■■□□□□□□□□■■■■■■■■■■■■□■■■■■■■■□□□□□□□	674001777677600	1 (0.04)	12.5	BOV	Unique
314	■■■■■■■■■■■■■■■■■■■□■■■■□□□□□□□■□□□□■■■□■■■	777777570020731	2 (0.09)	33.33	H3	Clustered
315	■■■■■■■■■■■■■■■■■■■■■■■□■□□□□□□■□□□□■■■□■■■	777777764020731	2 (0.09)	12.5	H1	Clustered
316	■■■■■■■■■■■■■■■■■■■■■■■■□□□□□□□■□□□□■■■□■■■	777777770020731	3 (0.13)	6.38	H3	Clustered
317	■■■■■■■■■■■■■■■■■■■■■■■■■■■□■■■■□□□□■■■□■■■	777777777360731	3 (0.13)	15	T2	Clustered
319	■□■■■■■■□□□□□■■■■■■■□□□□■■■■■■■■■■■■□□□■■■■	574077607777071	1 (0.04)	7.14	AFRI_2	Unique
320	■■■■■■□□□□□□□□□□■■■■□□□□■■□□■■■■■■■■□□□■■■■	770003606377071	2 (0.09)	16.67	AFRI_2	Clustered
326	■■■■■■□□□■■■■■■■■■■■■■□□□■■■■■■■■■■■■■■□■■■■	770777707777671	3 (0.13)	6	AFRI_1	Clustered
334	■□■■■■■■■■■■■■■■■■■■■■■■■■■■■■■■□□□□■■■■■■■	577777777760771	3 (0.13)	3.16	T1	Clustered
335	■■■■■■■■■■■■■■■■■■■■□□□□■■■■■■□■□□□□■■■■■■■	777777607720771	5 (0.22)	12.2	LAM9	Clustered
340	■□□■■■■□□□■■■■■■■■■■■■■■■■■■■□□□□■□■■■■■■■■■	474377777413771	2 (0.09)	4.55	EAI5	Clustered
367	■■■■■■■■■■■■□■■■■■■■□□□□□■■■■■□□□□■■■■■■■	777737404760771	1 (0.04)	7.69	LAM7-TUR	Unique
370	■■■■■■■■■■■■■■■■■■■■■■□□■■■■■■■■□□□□■□□■■■■	777777747760471	1 (0.04)	5	T1	Unique
373	■■■■■■■■■■■■■■■■■■■■■■■□■■■■■■■■□□□□■■■■■■■	777777767760771	6 (0.27)	9.09	T1	Clustered
379	■■■■■■■■■■■■■□□□□■□□□□□■■■■■■■■■□□□□■■■■■■■	777741017760771	3 (0.13)	14.29	Unknown	Clustered
390	■■■■■■■■■■■■■■■■■■■■■■■■■■■■■□□■□□□□■■■■■■■	777777777620771	2 (0.09)	6.45	H3	Clustered
393	■■■■■■■■■■■■■□■■■■■■■■■■■■■■■■■□□□□■■■■■■■	777757777760771	1 (0.04)	2.78	T1	Unique

395	■■■■■■■■■■■■■■■■■■■■■■■■■■■■■■■■□□□□■□□□□□□	777777777760400	1 (0.04)	16.67	T1	Unique
398	■■■■■■■■■■■■■■■■■■■■□□□□■■■■■■■■□□□□■■□□■■■	777777607760631	1 (0.04)	5.88	LAM4	Unique
402	■■■■■■■■■■■■■■■■■■■■□□□□□□□□□□□□□□□□□□□□□□□	777777600000000	1 (0.04)	2.5	Unknown	Unique
403	■■■■■■■■■■■■■■■■■■■■■■□□□■■■■■■■□□□□■■■□■■■	777777743760731	1 (0.04)	2.17	LAM10-CAM	Unique
411	■□□□□□■■■■■■■■■■■■■■■■■■■■■■■■■■□□□□■■■■■■■	407777777760771	1 (0.04)	11.11	T1	Unique
428	■■■□□□□■■■■■■■■■■■■■■■□□□□□□□□□□□□■■□■■■■■■	703777740003371	1 (0.04)	4.55	CAS1-Delhi	Unique
433	■■■■□■■■■■■■■■■■■■■■■■■■■■■□■■□■□□□□■■■■■■■	757777777320771	1 (0.04)	2.22	H3	Unique
442	■■■■■■■■■■■■□■■■■■□■■■■■■■■■■■■■□□□□■■■■■■■	777737377760771	1 (0.04)	11.11	T	Unique
450	■■■■■■■■■■■■■■■■■□■■■■■■□□□□□□□□□□□□□□□□□□□	777776770000000	1 (0.04)	1.06	Unknown	Unique
459	■■■■■■■■■■■■■■■■■■■■■■■■■■■■□□□□■□□□□■■□■■■	777777777410331	2 (0.09)	22.22	EAI1-SOM	Clustered
462	■■■■■■■■■■■■■■■■■■■■■■■■■■■■□■■■□□□□■■■■■■■	777777777560771	3 (0.13)	5.45	T1	Clustered
466	■■■■■■■■□□■■■■■■■■□■■■■■■■■■■■■■□□□□■■■■■■■	776377377760771	3 (0.13)	8.57	S	Clustered
481	■■□■■■■■□■■■■■■□■■■■□■■■■■■■■■■■■■■■■■□□□□□	676773677777600	12 (0.53)	0.23	BOV_1	Clustered
482	■■□■■■■■□■■■■■■□■■■■■■■■■■■■■■■■■■■■■■□□□□□	676773777777600	53 (2.35)	3.84	BOV_1	Clustered
488	■■■■■□□□□■■■■■■■■■■■■■■■■□□□□□□■□□□□■■■■■■■	760777774020771	4 (0.18)	40	H1	Clustered
498	■■■■■■■■■■■□■■■■■■■■■■■■■■■■■■■■□□□□■■■■■■■	777677777760771	2 (0.09)	7.41	T1	Clustered
504	■■■■■■■■■■■■□■■■■■■■■□■■■■■■■■■■□□□□■■■■■■■	777737737760771	1 (0.04)	5.26	T3	Unique
509	■■■■■■■■■■■■■■■■■■■■□□□□■□■■■■■■□□□□■■■■■■■	777777605760771	1 (0.04)	8.33	LAM9	Unique
511	■■■■■■■■■■■■■■■■■■■■■□□□□□□□□□□■□□□□■■■■■■■	777777700020771	2 (0.09)	4.55	H3	Clustered
512	■■■■■■■■■■■■■■■■■■■■■□□□■■■■■■□■□□□□■■■■■■■	777777707720771	4 (0.18)	12.12	H3	Clustered
521	■■■■■■■■■■■■■■■■■■■■■■■■■■■■■■■■□□□□■■□□□■■	777777777760611	2 (0.09)	7.41	T1	Clustered
522	■■■■■■■■■■■■■■■■■■■■■■■■■■■■■■■■□□□□■■■■■■□	777777777760770	1 (0.04)	6.67	T1	Unique
531	■■■□■■■■■■■■■■■■■■■■■■■■■□□□□□□■□□□□■■■■■■■	737777774020771	1 (0.04)	14.29	H1	Unique
535	■■■■■■■■■■■■■■■■■■■■■□□□■■■■■■■■□□□□■■■■■■■	777777707760771	1 (0.04)	0.93	T1	Unique
560	■■■■■■■■■□□□□□□□□□□□□□□□□□□□□□□□□□□□□■■■■■■	777000000000371	1 (0.04)	5.88	Unknown	Unique
584	■■■■■■■■■■■■■■■■□■■■■■■■■■■■■■■■□□□□■■■□■■■	777775777760731	1 (0.04)	4.35	T2	Unique
589	■■■■■■■■□□□■■■□□□■□□□□□□□□□□□□□□□□□□□□□■■■■	776161000000071	9 (0.4)	56.25	Unknown	Clustered
591	■■■■■■■■■■■■■■■■■■■■■■□■■■■■□□□□■□■■■■■■■■■	777777757413771	1 (0.04)	1.47	EAI6-BGD1	Unique
597	■□■■■□■■■■■■■■■■■■■■■■■■■■■■□□□■□□□□■■■■■■■	567777777420771	1 (0.04)	25	Ural-2	Unique
602	■■■■■■■■■■■■■■■■■■■■■■■■□□□□□□□□□□□□■■■■■■■	777777770000771	3 (0.13)	2.94	Unknown	Clustered
608	■■■■■■■■□□□■■■■■■■■■■■■■■□□□□□□□□□□□□□□□□□□	776177770000000	2 (0.09)	15.38	Unknown	Clustered
612	■■■■■■■■■■■■■■■■■■■■■■■■■■■■■■■■□□□□■■■■□■■	777777777760751	1 (0.04)	3.7	T1	Unique
617	■■■■■■■■■□■■■■■■■■■■■■■■■■□■□□□□■□■■■■■■■■■	777377776413771	2 (0.09)	40	EAI5	Clustered
618	■■■■■■■■■□■■■■■■■■■■■■■■■■■■□□□□■□■■■■■■■■■	777377777413771	2 (0.09)	15.38	EAI5	Clustered
619	■■■■■■■■■■■■■■■■■■□□□□□□□■□□□□□□□□□□□□□□□■■	777777002000011	1 (0.04)	25	Unknown	Unique
621	□□□□□□□□□□□□□□□□□□□□□□□□□□□□□□□□□□■□■■■■■■■	000000000002771	1 (0.04)	3.03	Beijing	Unique
628	■■■■■■■■■■■■■■■■■■■■■■■■■■■■■■■■□□□□■■■■■□□	777777777760760	1 (0.04)	6.67	T1	Unique
632	□□□□□□□□□□□□□□□□□□□□□□□□□□□□□□□□□□■■■□■■■■■	000000000003571	1 (0.04)	3.85	Beijing	Unique
646	□■□□□□□□□□□□□□□□■■■■■■■■■■■□■□□□□■■■■■□□□□□	200003777207600	1 (0.04)	0.34	BOV_4-CAPRAE	Unique
661	■■□□□□□■□■■■■■■□■■■■■■■■■■■■■■■■■■■■■■□□□□□	602773777777600	1 (0.04)	1.72	BOV_1	Unique
665	■■□□□■■■□■■■■■■□■■■■■■■■■■■■■■■■■■■■■■□□□□□	616773777777600	7 (0.31)	0.34	BOV_1	Clustered
683	■■□■■□■□□□□□■■■□■■■■■■■■■■■■■■■■■■■■■■□□□□□	664073777777600	1 (0.04)	0.05	BOV_2	Unique
684	■■□■■□■■□■■■■■■□■■■■□■■■■■■■■■■■■■■■■■□□□□□	666773677777600	2 (0.09)	0.21	BOV_1	Clustered
685	■■□■■■■□□□□□■■■□■■■■■■■■■■■■■■■■■■■■■■□□□□□	674073777777600	1 (0.04)	7.69	BOV	Unique
697	■■□■■■■■□■■■■■■□■■■■□■■■■■□□□■■■■■■■■■□□□□□	676773676177600	1 (0.04)	3.33	BOV_1	Unique
702	■■■□□□□□□■■■■■■■□■■■■■□□■■■■□□□□■□■■■■■■■■■	700775747413771	1 (0.04)	2.94	EAI6-BGD1	Unique
708	■■■□■■■■■■■■■■■■■□■■■■■■■■■■■■■■□□□□■■■■■■■	737776777760771	1 (0.04)	8.33	X1	Unique
713	■■■■■□□■■■■■■■■■■■■■■■■■■■■■■■■■□□□□■■■■■■■	763777777760771	6 (0.27)	46.15	T1	Clustered
719	■■■■■■■■□□□■■■■■■■■□□□□□■■■■■■■■□□□□■■■■■■■	776177407760771	1 (0.04)	1.1	LAM3	Unique
731	■■■■■■■■■■■■■■□□□■■■□□□□■■■■■■■■□□□□■■■■■■■	777761607760771	3 (0.13)	16.67	LAM9	Clustered
732	■■■■■■■■■■■■■■□□■■■■■■■■■■■■■■■■□□□□■■■■■■■	777763777760771	2 (0.09)	8.33	T1	Clustered
737	■■■■■■■■■■■■■■■■■■■■□□□□■■■■■■■■□□□□■□■■■■■	777777607760571	5 (0.22)	27.78	LAM9	Clustered
741	■■■■■■■■■■■■■■■■■■■■■■□■■■■■■■□■□□□□■■■■■■■	777777757720771	1 (0.04)	6.67	H3	Unique
746	■■■■■■■■■■■■■■■■■■■■■■■■■■■■□■□■□□□□■■■■■■■	777777777520771	2 (0.09)	6.67	H3	Clustered
751	□□□■■■■■■■■■■■■■■■■■■■■■■■■■■■■■□□□□■■■■■■■	077777777760771	5 (0.22)	25	T	Clustered
760	■■■■■■■□□□□□□■■■■■□□□□□□□□□□□□□■□□□□■■■□□□□	774037000020700	2 (0.09)	18.18	Unknown	Clustered
765	■■■■■■■■■■■■■■□□□□□□□■■■■■■■■■■■□□□□■■■■■■■	777760077760771	1 (0.04)	10	T1	Unique
766	■■■■■■■■■■■■■■□□□■□□□□□□■■■■■■■■□□□□■■■■■■■	777761007760771	2 (0.09)	4.17	LAM9	Clustered
769	■■■■■■■■■■■■■■■■■■■□□■■■■□□□□□□■□□□□■■■■■■■	777777474020771	2 (0.09)	50	H1	Clustered
775	■■■■■■■■■■■■■■■■■■■■■■■■■■■□□□□□□□□□□■■■■■■	777777777000371	3 (0.13)	7.69	Unknown	Clustered
781	□□□□□□□□□□□□□□□□□□□□□□□□■□□□□□□■□□□□■■■□■■■	000000004020731	1 (0.04)	7.69	H2	Unique
782	■□□□□■■■■■■■■■■■■■■■□□□□■■■■■■■■□□□□■■■■■■■	417777607760771	1 (0.04)	25	LAM1	Unique
784	■■■■■■■■□□■■■■■■■■■■■■■■■■■■■■■■□□□□■■■□■■■	776377777760731	4 (0.18)	6.78	S	Clustered
791	■■■■■■■■■■■■■■■■■■■■■■■□□□□□□□□■□□□□■■■■■■■	777777760020771	2 (0.09)	20	H3	Clustered
801	■■■■■■■□□■■■■■■■■■■■■■■■■■■■■■■■□□□□■■■■■■■	774777777760771	1 (0.04)	11.11	T1	Unique
803	■■■■■■■■■■■■■□□□□□□□□□□□■■■■■■■■□□□□■■■■■■■	777740007760771	1 (0.04)	3.03	LAM9	Unique
804	■□□■■■■■■■■■■■■■■■■■■■■■■■■■■■■■□□□□■■■■■■■	477777777760771	2 (0.09)	8	T1	Clustered
806	■■■■□■■■■■■■■■■■■■■■■■■■■■■■□□□□■□■■■■■□■■■	757777777413731	1 (0.04)	1.92	EAI1-SOM	Unique
820	■■□■■■■■□■■■■■□□■■■■■■■■■■■■■■■■■■■■■■□□□□□	676763777777600	1 (0.04)	1.49	BOV	Unique
822	■■■■□■■■■■■■■■■■■■■■□□□□■■■■■■■■□□□□■■■■■■■	757777607760771	4 (0.18)	19.05	LAM9	Clustered
823	■■■■■■■■□□□□□□□□□□□□□□□□□■■■■■■■□□□□■■■■■■■	776000003760771	1 (0.04)	7.69	T1	Unique
831	■■■■■■■■□□■■■■□■■■■■■■■■■■■■■■■■□□□□■■■■■■■	776367777760771	1 (0.04)	8.33	S	Unique
838	■■■■■■■■■■■■■■■■■■■■■■□□□■■■■■■■□□□□■■■■□■■	777777743760751	1 (0.04)	3.57	LAM10-CAM	Unique
839	■■■■■■■■■■■■■■■■■■■■■■□□□■■■■■□□□□□□□□■■■■■	777777743700171	1 (0.04)	25	Unknown	Unique
843	■■■■■■■■■□□■■■■■■■■■■■■■■■■■■■□■□□□□■■■■■■■	777177777720771	1 (0.04)	20	H3	Unique

844	■■■■■□□□■■■■■■■■■■■■■■□□□■■■■■■■□□□□■■■□■■■	761777743760731	1 (0.04)	33.33	LAM10-CAM	Unique
852	■□□□□□□□□□□□□□□□■■■■■■□□□■■■■■■■□□□□■■■■■■■	400003743760771	1 (0.04)	3.7	LAM10-CAM	Unique
853	□■■■■■■■■■■■■■■■■■■■■■■■■■■■■■■■□□□□■■■□■■■	377777777760731	1 (0.04)	3.45	T2	Unique
866	■□■■■■■■■■■■■■■■■■■■□□□□■■■■■■■■□□□□■■■■■■■	577777607760771	4 (0.18)	8.33	LAM9	Clustered
871	■■■□■■■■■■■■■■■■■■■■■■■■■■■■■■□■□□□□■■■■■■■	737777777720771	2 (0.09)	9.52	H3	Clustered
872	■■■■■■□■■■■■■■■■■■■■■□□■□□■■■■■■■□□□□■■■■■■■	773777623760771	2 (0.09)	18.18	T1	Clustered
877	■■■■■■□□□□□□□□□□□□■■■■■■□□□□□□□□□□□□□□□□□□□	770000770000000	1 (0.04)	7.69	Unknown	Unique
891	■■■■■■■■■■■■■■■■■■■■□□□□■■■■■□■■□□□□■■■■■■■	777777607660771	1 (0.04)	3.57	LAM9	Unique
898	■■□■■■■■■■■■■■■■■■■■■■■■■□□□□□□■□□□□■■■■■■■	677777774020771	1 (0.04)	12.5	H1	Unique
910	■■■□□□□□□□□□□□□□□□□□□□□□■■■□□■■■■■■■■■■■■■■	700000007177771	2 (0.09)	8.7	Unknown	Clustered
911	■■■■■■■■■■■■■■■■■■■■□□□□□□□□□□■■□□□□■■■■■■■	777777600060771	1 (0.04)	2.94	Unknown	Unique
912	■■□■■■■■■■■■■■■■■■■■■■■■■■■□□□□■□■■□■■■■■■	677777777413371	4 (0.18)	40	EAI5	Clustered
918	■■■■■■□□□□□□□□□□□□■■□□□□■■■■■■■■□□□□■■■■■■■	770000607760771	2 (0.09)	11.11	Unknown	Clustered
921	■□□□■■■■■■■■□■■■■■■■■■■■■■■■□□□■□□□□■■■■■■■	437737777420771	1 (0.04)	14.29	Ural-2	Unique
925	■■■■■■■■■■■■□□■■■■■■■■■■■■■■■■□■□□□□■■■■■■■	777717777720771	1 (0.04)	16.67	H3	Unique
926	■■■■■■□■■■■■■■■■■■■■■■■■■■■■■■■□□□□■■■■■■■	773777777760771	1 (0.04)	3.13	T1	Unique
928	■■■■■■■■■■■■■■■■■■■■■■□■□□□□□□■■□□□□■■■■■■■	777777750060771	1 (0.04)	5	T	Unique
941	□□□□□□□□□□□□□□□□□□□□□□□□□□□□□□□□□□■■■■■■□■■	000000000003751	2 (0.09)	8.33	Beijing	Clustered
943	■■■■■■■■■■■■■■■■■■■■■■□■■■■■■■■■□□□□■■■□■■■	777777757760731	1 (0.04)	7.69	T	Unique
964	■□□□■■■■■■■■■■■■■■■■□□□□■■■■■■■■□□□□■■■■■■■	437777607760771	2 (0.09)	9.52	LAM1	Clustered
966	■■■■■■■□■■■■■■■■■■■■■■■■■■■■■■■■□□□□■■■■■■■	775777777760771	1 (0.04)	7.69	T1	Unique
980	■□□□□□□□□□□□□□□□□□□□□■■■■■■■■■■■■■■■■■□□□□□	400000077777600	1 (0.04)	0.28	BOV	Unique
1022	■■□■■■■■□■■■■■■□■■■■□■■■■■■■■■■□□□■■■■□□□□□	676773677743600	1 (0.04)	5.88	BOV_1	Unique
1043	■■□■■■■■□■■■■■■□■■■■■■■■■■■■■■■■□■■□■■□□□□□	676773777766600	1 (0.04)	16.67	BOV_1	Unique
1044	■■□■■■■■□■■■■■■□■■■■■■■■■■■■■■■■□■■■■■□□□□□	676773777767600	2 (0.09)	2	BOV_1	Clustered
1054	■■■■■■■■■■■■□■■■■■■■■■■□□□□□□□■□□□□■■■□■■■	777737770020731	1 (0.04)	11.11	H3	Unique
1056	■■■■■■■■■■□□■■■■■■■■■■■■■■■■■■■■□□□□■■■□■■■	777477777760731	3 (0.13)	30	T2	Clustered
1064	■■■■■■□□■■■■■■■■■■■■□□□□■■■■■■■■□□□□■■■■■■■	771777607760771	2 (0.09)	5.71	LAM9	Clustered
1067	■■■■■■■□□□□□□■■■■■■■■□□□■■■■■■■■□□□□■■■□□□□	774037707760700	3 (0.13)	8.82	Unknown	Clustered
1070	■■■■■■■■■■■■■■■■■■■■□□□□■■■■■■■■□□□□□■■■■■■	777777607760371	4 (0.18)	21.05	LAM9	Clustered
1071	■■■■■■■■■■■■■■■□□■■■□□□□■■■■■■■■□□□□■■■■■■■	777771607760771	1 (0.04)	16.67	LAM9	Unique
1073	■■■■■■■■■■■□□■■■■■■■■■■■■■■■■■■■□□□□■■■■■■■	777637777760771	1 (0.04)	14.29	T1	Unique
1074	■■■■■■■■■■■■■■■■■■■■□□□□■■■■■■■■□□□□■■■■■□■	777777607760761	6 (0.27)	19.35	LAM9	Clustered
1093	■■■□□□□■■■■■■■■■■■■■□□□□□□□□□□□□□□■■■■■■■■■	703777600003771	1 (0.04)	7.69	CAS1-Delhi	Unique
1122	■■■■■□■■■■■■■■■■■■■■■■■■■■■■■■■■□□□□■■■■■■■	767777777760771	1 (0.04)	1.72	T1	Unique
1125	■■■■■■□■■■■■□■■■■■■■■■■■■□□□□□□■□□□□■■■■■■■	773737774020771	1 (0.04)	20	H1	Unique
1128	■■■■■■■■□□■■■■■■■■■■■□■■□□□□■■■■□□□□■■■■■■■	776377730360771	1 (0.04)	16.67	S	Unique
1129	■■■■■■■■□■■■■■■■■■■■■■■■■■■■■■■■□□□□■■■■■■■	776777777760771	2 (0.09)	13.33	T1	Clustered
1135	■■■■■■■■■■■■□■■■■■■■■■■■■■■■■□■□□□□■■■□■■■	777737777720731	1 (0.04)	12.5	H3	Unique
1167	■■■■■■■■■■■■□□□□■■■■■□■■■■■■■■■■□□□□■■■■■■■	777703737760771	1 (0.04)	25	T1	Unique
1176	■■■■■■■■■■■■■□■■■■■■□□□□■■■■■■■■□□□□■■■■■■■	777757607760771	1 (0.04)	5	LAM9	Unique
1198	■■■□□□□■■■■■□■■■■■■■■■□□□□□□□□□□□□■■□□■■■■■	703737740003171	1 (0.04)	3.57	CAS1-Delhi	Unique
1200	■■■□□□□■■■■■■■■■■■■■■■□□■■■■■■■■■■■■■■■■■■■	703777747777771	2 (0.09)	18.18	Unknown	Clustered
1243	■■■■■■■■■□■■■■■■■■■■■■■■■■■■■■□■□□□□■■■■■■■	777377777720771	1 (0.04)	6.25	H3	Unique
1252	■■■■■■■■■■■■■■■■■■□□■■■■■■■■■■■■□□□□■■■■■■■	777777177760771	2 (0.09)	25	T1	Clustered
1253	■■□■■■■■□□■■■■■■■■■■■□■■■■■■■■■■□□□□■■■■■■■	676377737760771	1 (0.04)	5.88	S	Unique
1264	■■■□□□□■■■■■■■■■■■■■■■□□□□□□□□□□□□□□□□□□□□□	703777740000000	1 (0.04)	4.76	CAS1-Delhi	Unique
1278	□□□□■■■■■■■□■■■■■■■■■■■■■■■■■■■■□□□□■■■■■■■	037677777760771	4 (0.18)	25	T1	Clustered
1282	■■■■■■■■■■■■■■■■■■■□□□□□□□□□□□□■□□□□■■■■■■■	777777400020771	2 (0.09)	18.18	H3	Clustered
1284	■■■■□□□□■■■■□□□□■■■■■■■■■■■■■■■■□□□□■■■■■■■	741703777760771	1 (0.04)	8.33	T1	Unique
1321	■■□■■■■■■■■■■■■■■■■■□□□□■■■■■■■■□□□□■■■□■■■	677777607760731	1 (0.04)	5.88	LAM	Unique
1332	■■□■■■■■■■■■■■■■■■■■■■■■■■■■■■■■□□□□■■■□■■■	677777777760731	4 (0.18)	20	T2	Clustered
1367	□■■■■■■■■■■■□■■■■■■■□□□□■■■■■■■■□□□□■■■■■■■	377737607760771	1 (0.04)	25	LAM5	Unique
1392	■■■■■■■■■■■■■■■■■■■■■■■■■■■■□□□□■□■■■□□□■■■	777777777413431	1 (0.04)	14.29	EAI5	Unique
1471	■■■■■■■■■■■■□■■■■■■■□□□□■■□□□□■■□□□□■■■■■■■	777737606060771	1 (0.04)	7.69	LAM11-ZWE	Unique
1476	■■■□■■■■□□□■■■■■■■■■□□□□■■■■■■□□□□□□□□■■■■■	736177607700171	1 (0.04)	7.69	AFRI_2	Unique
1514	■■■■□■■■■■■■■■■■■■■■□□■■■■■■■■■■□□□□■■■■■■■	757777637760771	1 (0.04)	16.67	T1	Unique
1525	□■■■■■■■□□□■□■■■■■■■□□□□■■■■■■■■□□□□■■■■■■■	376137607760771	1 (0.04)	16.67	LAM3	Unique
1530	■■■■■■■■■■■■■■■■■■■■□□□□■■■■■■■■□□□□■■■□□■■	777777607760711	1 (0.04)	9.09	LAM4	Unique
1533	■■■■■■■■■■■■■■□□■■■■■■■■■■■■■■□■□□□□■■■■■■■	777763777720771	1 (0.04)	14.29	H3	Unique
1535	■■■■■■■■■■□■■■■■■■■■□□□□■■■■■■■■□□□□■■■■■■■	777577607760771	3 (0.13)	25	LAM9	Clustered
1538	■■■■■■■□□□□■□■■■■■■■□□□□□□□□□□□■□□□□■■■■■■■	774137600020771	2 (0.09)	14.29	Unknown	Clustered
1544	■■■■■■■■■■■■■■■■■■■■■■■■■■■■■■■■□□□□■□■□■■■	777777777760531	1 (0.04)	10	T2	Unique
1548	■■■■■■■■■■■■□□□□■■■□□□□□□□□□□□□□□□□□□■■■■□□	777703400000360	2 (0.09)	28.57	Unknown	Clustered
1549	■■■■■■■□■■■■■■■■■■■■□□□□■■□□□□■■□□□□■■■□■■■	775777606060731	1 (0.04)	11.11	LAM11-ZWE	Unique
1559	■■■■■■■■■■■■■■■■■■■□□□□□□□□□□□□□□□□□□□■■■■■	777777400000171	2 (0.09)	22.22	Unknown	Clustered
1574	■■■■■□□□□□□□□□□□□□□□□□□□■■■■■■■■□□□□■■■■■■■	760000007760771	1 (0.04)	12.5	LAM3	Unique
1578	■■■■■□■■■■■■■■■■■■■■■■■■■■■■■■■■□□□□■■■□■■■	767777777760731	2 (0.09)	28.57	T2	Clustered
1580	■■■■■■■■■■■■■■■■■■■■■■□□■■■■■■■■□□□□■■■■■■■	777777747760771	1 (0.04)	5.56	T	Unique
1609	■■■■■■■■■■■□□■■■■■■■■■■■■■■■□□□□■□■■■■■■■■■	777637777413771	1 (0.04)	25	EAI5	Unique
1629	■■■■■■■■■■■■■■■■■■■■■■■■■□□□□□□■□□□□■■■■□■■	777777774020751	1 (0.04)	14.29	H1	Unique
1640	■■■■■■■■■■■■■■■■■■■■■■■■■■■■■■□■□□□□■■■■□□□	777777777720740	1 (0.04)	25	H3	Unique
1657	■■■■■■■■■■■■■■■■■■□■■■■■■■■■□□□□□□□□□□■■■■■■	777777377000371	1 (0.04)	20	Unknown	Unique
1684	■■■■■■■■□□□□□□□□□□□□□□□□□□□□□□□□□□□□□□□□□□□	776000000000000	1 (0.04)	25	Unknown	Unique
1736	■■■■■■■■■■■■■■■■■■■■■□□□□□□□□□□□□□□□□■■■■■■	777777700000371	1 (0.04)	14.29	Unknown	Unique
1743	■■■■■□■■■■■■■■■■■■■■■■■■■■■■■■□■□□□□■■□□■■■	767777777720631	1 (0.04)	14.29	H3	Unique

1753	■■■□□□□□□□□□□■■■■■■■■■■■■■■■■■■■□□□□■■■■■■■	700037777760771	1 (0.04)	12.5	Unknown	Unique
1776	■■■■■■■■□□■■■■■■■■■■■■■□□□□□□□□□□□□□□□■■■■■	776377760000171	2 (0.09)	40	Unknown	Clustered
1789	■■■□□□□■■■■■■■■■■■■■■■□□□□□□□□□□□□□□□□■■■■■	703777740000171	3 (0.13)	15	CAS	Clustered
1798	■□■■■■■■■■■■■■■■■■■■■■□□■□□□□□□■□□□□■■■■■■■	577777744020771	1 (0.04)	33.33	H1	Unique
1803	■■□□□■■■■■■■■■■■■■■■□□□□■■■■■■■■□□□□■■■■■■■	617777607760771	1 (0.04)	9.09	LAM1	Unique
1832	■■■■■■■■■■■■■■■□■■■■□□□□■■■■■■■■□□□□■■■■■■■	777773607760771	2 (0.09)	10	LAM9	Clustered
1844	■■■■■■■■■■■■■■□□■■■■□□□□■■■■■■■■□□□□■■■■■■■	777763607760771	1 (0.04)	11.11	LAM9	Unique
1877	■■■□■■■■■□■■■■■■■■■■■■■■■■■■■■■■□□□□■■■■■■■	737377777760771	1 (0.04)	7.69	T1	Unique
1916	■■■■■■■■■■■■□□□□□■■■■■■■■■■■■■■□□□□■■■■■■■	777701777760771	1 (0.04)	12.5	T1	Unique
1926	■■■■■■■■■■■■■■■■■■■■■■■■■■■■■■■■□□□□■■■□□□■	777777777760701	1 (0.04)	14.29	T1	Unique
1933	■■■■■■■■■■■■■■■■■■■■□□□□■■■■■■■■□□□□■■■■■■□	777777607760770	1 (0.04)	25	LAM9	Unique
1951	■■■□□□□■■■■■■■■■■■□□□□□□□□□□□□□□□□■■■■■■■■■	703777000003771	1 (0.04)	11.11	CAS1-Delhi	Unique
1966	■□□■■■■■■■■■■■■■■■■■■■■■■■■■□□□□■□■■□□□□□■■	477777777413011	1 (0.04)	33.33	EAI5	Unique
1981	□□■□□□□■■■■■■■■■■■■■■■□□□□□□□□□□□□■■□□■■■■■	103777740003171	1 (0.04)	10	CAS1-Delhi	Unique
2007	■■■■■■■■■■■■□■■■■■■■□■■■■■■■■■■■□□□□■■■■■■■	777737677760771	1 (0.04)	20	T3	Unique
2037	■■■■■■■■■■■■■■■■■■■■■■■■■□□□■■■■□□□□■■■■■■■	777777774360771	1 (0.04)	16.67	T1	Unique
2045	■■■■■■■■□□□□□■■■■■■■□□□□■■■■■■■■□□□□■■■■■■■	776037607760771	5 (0.22)	33.33	LAM3	Clustered
2047	■■■■■■■■□□□■■■□□■■□□□□□□□□□□□□□□□□□□□□□■■■■	776163000000071	1 (0.04)	14.29	Unknown	Unique
2052	■■■□■■■■■■■■□■■■■■■■■■■■■■■■■■■■□□□□■■■■■■■	737737777760771	1 (0.04)	25	T3	Unique
2056	■■■■■■□□□■■□□■■■■■■■■■■■■■■■■■■■□■■■■■□■■■■	770637777767671	1 (0.04)	25	AFRI_1	Unique
2066	■■■■■■■■■■■■■■■■■■■□□□□□□□□□□□■■□□□□■■■■■■■	777777400060771	1 (0.04)	25	Unknown	Unique
2088	■■■■■■■■■■■■■■■■■■■■■□■■■■■■■■■■□□□□■■■□■■■	777777737760731	2 (0.09)	40	T2	Clustered
2089	■■■■■■■■■■■■■■■□■■■■■■■■■■■■■■□■□□□□■■■■■■■	777773777720771	2 (0.09)	10.53	H3	Clustered
2107	■■■■■■■■□□□■■■■■■■■■□□□□□□□□□□□□□□□□□□□■□■■■	776177400000131	1 (0.04)	33.33	Unknown	Unique
2191	■■■■■■■■■■■■■□□□□■□□□□□□■■■■■■■■□□□□■■■■■■■	777741007760771	1 (0.04)	33.33	LAM9	Unique
2234	■■■■■■■■■■■■□■■■■■■■■■□□□□□□□□□□□□□□□■■□■■	777737760000151	1 (0.04)	20	Unknown	Unique
2242	■■■■■■■■■■■■■■■■■■■■■■□□□■■■□□■□□□□□■■■■■■■	777777743440771	1 (0.04)	33.33	LAM10-CAM	Unique
2258	■■■■■■■■■■■■■■■■■■■■■■■■■■■■■■□□□□□□□□□□□■■	777777777700011	1 (0.04)	11.11	Unknown	Unique
2272	■■■■□□□□□■■■■■■■■■■■■■■■■■■■■■■□□□□■■■■■■■	740777777760771	1 (0.04)	33.33	T1	Unique
2275	■■■■□□□□□□□□□□□□□□□□□□□□■■■■■■■■□□□□■■■■■■■	740000007760771	1 (0.04)	25	LAM3	Unique
2333	■■■■■■■■■■■■■■■■■■■■■■■■■□□□□□□■□□□□■■■□□■■	777777774020711	1 (0.04)	9.09	H1	Unique
2337	■■■■■■□■■■■■■■■■■■■■■■■■■□□□□□□■□□□□■■■■■■■	773777774020771	1 (0.04)	12.5	H1	Unique
2338	■■■■■■■■■■■□□□□□□□■■■■■■■■■■■■□■□□□□■■■■■■■	777600777720771	3 (0.13)	33.33	H3	Clustered
2367	■■■■■□■■□□■■■■■■■■■■■■■■■■■■■■■■□□□□■■■■■■■	766377777760771	1 (0.04)	16.67	S	Unique
2382	■■■■■■■■■■■■□■□□■■■□□□□□□□□□□□□□□□□□□■■■■□□	777723400000360	2 (0.09)	50	Unknown	Clustered
2385	■■■■■■■■■■■■■■■■■■■■□□□□■■□□□□■■□□□□■□□■■■■	777777606060471	2 (0.09)	28.57	LAM11-ZWE	Clustered
2387	■■■■■■■■■■■■■■■■■■■■■■■■■□□□□□□□□□□□□■■□■■■	777777774000331	4 (0.18)	57.14	Unknown	Clustered
2401	■■■■■■■■■■■■■■■■■■■■□□□□■■■■■□□□□□□□□■■■■■■	777777607600371	1 (0.04)	33.33	Unknown	Unique
2423	■■■■■■■■■■■■■■■■■■■□□□■■■■■■■■■■□□□□■■■□■■■	777777437760731	1 (0.04)	33.33	T2	Unique
2424	■■■■■■■■■■■■■■■■□■■■■■■■■■■■■■■■□□□□■■■□□□□	777775777760700	1 (0.04)	33.33	T1	Unique
2445	■□□■■■■■■■■■■■■■■■■■■■■■□□□□□□□□□□□□□□□□□□□	477777770000000	1 (0.04)	20	Unknown	Unique
2551	■■■■■■■■■■■■■■■■■■■■□□□□■■■■■■■■□□□□■■■□□□■	777777607760701	1 (0.04)	16.67	LAM4	Unique
2572	■■□■■□■■■■■■■■■■■■■■□□□□■■■■■■■■□□□□■■■■■■■	667777607760771	1 (0.04)	33.33	LAM1	Unique
2577	■■■■■■■■■■□□■■■■■■■■■■■■■■■■■■■■□□□□■■■■■■■	777477777760771	1 (0.04)	10	T1	Unique
2632	■■■■■■■■■■■■■■■■■■■■□□□□■■■■□□□■□□□□■■■■■■■	777777607420771	2 (0.09)	33.33	Ural-1	Clustered
2644	□□■■■■■■■■■■■■■■■■■■■■■■■■■■■■□■□□□□■■■■■■■	177777777720771	3 (0.13)	42.86	H3	Clustered
2660	■■■■■■■■■■■■■■■■■■■■■■■■■■■■■□□□□□□□□□■□■■■	777777777600131	3 (0.13)	50	Unknown	Clustered
2721	■■■■■■■■■■■■■■■■■■■■■□□□□□□□□□□□□□□□□□■■■■■	777777700000171	1 (0.04)	25	Unknown	Unique
2733	■■■■■■■■■■■■■■■■■■■■■■■□□□□□□□□□□□□□□■■■■■■	777777760000371	1 (0.04)	33.33	Unknown	Unique
2735	■■■■■■■■■■□■■■■■■■■■■■■■■■■■■■□■□□□□■■■■■■■	777577777720771	1 (0.04)	33.33	H3	Unique
2765	■■■■■■□□□■■■■■■■■■■■□■■■■■■■■■■■■■■■■■□■■■■	770777677777671	1 (0.04)	33.33	AFRI_1	Unique
2791	■■■■■■■■■■■■■■■■■■■■■■■■□□□■■■■■□□□□■■■■■■■	777777770760771	1 (0.04)	25	T1	Unique
2797	■□■■■■■■■■■■■■■■■■■■■■■■□□□□□□□□□□□□□□□□□□□	577777770000000	1 (0.04)	33.33	Unknown	Unique
2819	■■■■■■□□□■■■■■■■■■■■■■□□□□■■□□□□□■■■■■■□■■■■	770777703017671	1 (0.04)	33.33	AFRI_1	Unique
2883	■■■■■■■□□□□□□□□□□□□□□□□■■■■■■■■■□□□□■■■■■■■	774000017760771	2 (0.09)	28.57	Unknown	Clustered
2889	■□□■■■■■□■■■■■■■■■■■■■■■■■■■□□□□■□■■■■■■■■■	476777777413771	1 (0.04)	33.33	EAI5	Unique
2899	■□□■■■■■■□■■■■■■■■■■■□□□■■■■■■■■□□□□■■■□■■■	477377707760731	1 (0.04)	33.33	T2	Unique
2905	□■■■■■■■■■■■□□■■■■■■■■■■■■■■■■■■□□□□■■■■■■■	377717777760771	2 (0.09)	25	T1	Clustered
2946	■■■■■■■■□□□■□■■■■■■□□□□□□□□□□□□□□□□□□□■■■■■	776137400000171	2 (0.09)	33.33	Unknown	Clustered
2953	■■■■■■■□□□□□□■■■■■■■□□□□■■■■■■■■□□□□■■■■■■■	774037607760771	1 (0.04)	33.33	LAM3	Unique
3020	■■■■■■■■■■■■■■■■■■■■□□□□■■■□■■■■□□□□■■■■■■■	777777607360771	1 (0.04)	25	LAM9	Unique
3076	■■■■■■■■■■■■■■■■■■■■■■□□□■□□□■■■□□□□■■■■■■■	777777742160771	1 (0.04)	11.11	T	Unique
3089	□■■□■■■■■■■■■■■■■■■■■■■■■■■■■■■■□□□□■■■■■■■	337777777760771	1 (0.04)	25	T1	Unique
3091	■■■■■■■■■■■■■■■■■■■■■■■■■■■■■■■□□□□□□□□□■■■	777777777740031	1 (0.04)	14.29	T	Unique
3102	■□■■■■■■■■■■■■■■■■■■□□□□■■■■□■■■□□□□■■■■■■■	577777607560771	1 (0.04)	33.33	LAM9	Unique
3127	□□□□□□□□□□□□□□□□□□□□□□□■■■■■■■■■□□□□■■■■■■■	000000017760771	1 (0.04)	25	Unknown	Unique
3164	■■■■■■■■■■■■■■■■■■■■□□□□■■■■■■■■□□□□■■□□□□□	777777607760600	1 (0.04)	33.33	LAM	Unique
3179	■■■■□■■■■■■■■■■■■■■■■■■■■■■■■■□■□□□□■■■□■■■	757777777720731	2 (0.09)	50	H3	Clustered
3214	■■■■■■■■■■■■■■■■■■■■■■■■■■□□□□■■□□□□■■■■■■■	777777776060771	1 (0.04)	33.33	T	Unique
3234	■■■■■■■■■■■■■■■■■■■■■■■■■■■■■□□□□□□□□■■■■■■	777777777600371	1 (0.04)	25	Unknown	Unique
3258	■■■■■■■■□□□□□□■■■■■■■■■■■□□□□□□■□□□□■■■■■■■	776017774020771	1 (0.04)	25	H1	Unique
3272	■■■■■■■■■■■■■■■■■■■■■□□□□□□□□□□□□□□□■■■□■■■	777777700000731	2 (0.09)	50	Unknown	Clustered
3313	□□□□□□□□□□□□□□□□□□□□□□□□■□□□□□□■□□□□■■□□■■■	000000004020631	4 (0.18)	66.67	H2	Clustered
3338	■■■□□□■■■■■■■■■■■■■■■■■■■■■■■■■■□□□□■■■□□□□	707777777760700	1 (0.04)	25	T1	Unique
3347	□□□□□□□□□□□□□□□□□□□□□□□□■□■■■■□■□□□□■■■■■■■	000000005720771	6 (0.27)	75	H3	Clustered
3436*	■■■■□□□□□□■■■■■■■■■■□□□□■■■■■■■■□□□□■■■■■■■	740377607760771	1 (0.04)	50	LAM9	Unique

3437*	■■■■■■■■■■■■■■■■■□■■■■■■■■■■■■■■■□□□□■■■□■■	777776777770351	2 (0.09)	100	Unknown	Clustered
3438*	■■■■■■■■■■■■■■■■■■■■■■■■■■■■□□□■□□□□■■■■■□□	777777777420760	2 (0.09)	100	Ural-1	Clustered
3439*	■■■■■■■■■■■■□■■■■■■■■■■■■□■■■■■□□□□■■■■■■■	777737776760771	1 (0.04)	50	T3	Unique
3440*	□■□■■■■■■■■■■■■■■■■■□□□□■■■■■■■■□□□□■■■■■■■	277777607760771	1 (0.04)	33.33	LAM1	Unique
3441*	■■■■■■■■■■■■■■■■■■□□□□■■■■■■■■■■□□□□■■■□■■■	777777037760731	6 (0.27)	100	T2	Clustered
3442*	■■■■■■■■■■■■□■■■■■■■■■■■■■□□□□□□□■■□□□□■■■■■■■	777737760060771	3 (0.13)	75	T3	Clustered
3443*	□■□□■■■■■■■■■■■■■■■■■■■■■■■■■■■■□□□□■■■■■■■	237777777760771	2 (0.09)	100	T1	Clustered
3444*	■■■■■■■■■■■■□■■■■■■■■■■■■■■■■■■■□□□□□□□■■■■	777737777760071	1 (0.04)	50	T3	Unique
3445*	□□□■□□■■■■■■■■■■■■■■□□□□■■■■■■■■□□□□■■■■■■■	047777607760771	1 (0.04)	50	LAM1	Unique
3446*	■■■■■■■□□□□■□■■■■■■■□□□□■■■■■■□■□□□□■■■■■■■	774137607720771	1 (0.04)	50	H3	Unique
3447*	■■■■■■■■■■■■■■■■■■□□■■■■■■■■■■□■□□□□■■■■■■■	777777177720771	4 (0.18)	100	H3	Clustered
3448*	■■■■■■■□□□□■■■■■■■■■■■■■■■■■■■□■□□□□■■■■■■■	774177777720771	2 (0.09)	66.67	H3	Clustered
3449*	■■■■■■■□□□□■□■■■■■■■■■■■■■■■■■□■□□□□■■■■■■■	774137777720771	3 (0.13)	100	H3	Clustered
3450*	■■■■■■■■■■■■■■■■■■■□□□□□■□■■■■□■□□□□■■■■■■■	777777405720771	1 (0.04)	50	H3	Unique
3451*	■□□■■■■■■■■■■■■■□■□■■■■■■□□□□□□■□□□□■■■■■■■	477775374020771	3 (0.13)	75	H1	Clustered
3452*	■■□■■■■■□□□■■■■□■■■■■■■■■■■■■■■■■■■■■■□□□□□	676173777777600	1 (0.04)	50	BOV	Unique
3453*	■■□□□□□■□■■■■■■□■■■■■■■□□□■□□□□□□□□□□■□□□□□	602773761000200	1 (0.04)	1.61	BOV_1	Unique
3454*	■■■■■■■■■■■■□■■■■■■■■■■■□□□□□□■■□□□□■■■■■■■	777737770060771	2 (0.09)	100	T3	Clustered
3455*	■■■■■■■■■■□□■■■■■■■■□■■■■■■■■■■□□□□■■■□■■■	777477677760731	1 (0.04)	50	T2	Unique
3456*	■■■■■■■■■■■■■■■■■■■□□□□□□□□■■■■■□□□□■■■■■■■	777777400760771	1 (0.04)	50	T1	Unique
3457*	■■□■■■■□■■■■■■■■■■■■□□□□■■■■■■■■□□□□■■■■■■■	675777607760771	1 (0.04)	50	LAM1	Unique
3458*	■■■■■■■■■■■■■■■□■■■■■■■■■■■■■■■■□□□□■■■□■■■	777773777760731	1 (0.04)	50	T2	Unique
3459*	■■■■■■■■■■■■■■■■■■■■■□□□□□□□□□■■□□□□■■■■■■■	777777700060771	1 (0.04)	50	T1	Unique
3460*	■■■■■■■■■■■■■■■■■■■■■■■■■■■■□□□□□□□□□■■■■■■	777777777400371	1 (0.04)	50	Unknown	Unique
3461*	■■■■□■■■■■■■■■■■■■■■■■□□□■■■■■■■□□□□■■■□■■■	757777743760731	2 (0.09)	100	LAM10-CAM	Clustered
3462*	■■■■■■■■■■■■■□□□□□□■■■■■■■■■■■■■□□□□■■■■■■■	777740377760771	1 (0.04)	50	T1	Unique
3463*	■■■■■■■■■■■■■■■■■■□□■■■■■■■■■■■■□□□□■■■□■■■	777777177760731	1 (0.04)	50	T2	Unique
3464*	■■■■■■■■■■■■■■■■■■■■■■□■□□□□□□□□□□□□□□□□□□□	777777750000000	1 (0.04)	50	Unknown	Unique
3465*	■■■■■■■■■■■■■■■■■■□■■■■□□□□□□□■■□□□□■■■■■■■	777777360060771	1 (0.04)	50	T4	Unique
3466*	■■■■■■■■■■■■■■■■■■■■■□□□□□□□□□□□□□□□■■■■■■■	777777700000771	1 (0.04)	50	Unknown	Unique
3467*	■■■■■■■■■■■■■■■■■■■■■■■■■■■■□□□□□□□□□□■□■■■	777777777400131	1 (0.04)	50	Unknown	Unique
3468*	■■■■■■■■■■■■□■■■■■■■■■■■■■■■■■■■□□□□■■■■■■□	777737777760770	2 (0.09)	100	T3	Clustered
3469*	■■■■■■■■□□□■■■■■■■■□□□□□□□□□□□□□□□□□□□□■■■■	776177400000071	1 (0.04)	50	Unknown	Unique
3470*	■■■■■■■■■■■■■□□□□■□□□□□□□■■■■■■■□□□□■■■□■■■	777741003760731	4 (0.18)	100	T2	Clustered
3471*	□□□□□□□□□□□□□□□□□□□□□□□□□□■■□■■■□□□□■■■□■■■	000000001560731	4 (0.18)	100	T2	Clustered
3472*	■■■■■■■■□□□■■■■■■■■■□□□□■■■■■■■■□□□□■■■■■■□	776177607760770	1 (0.04)	50	LAM3	Unique
3473*	■■■■□□□□□■■■■■■■■■■■□□□□■■□□□□□□□□□□□□□□□■■	740777606000011	2 (0.09)	66.67	Unknown	Clustered
3474*	■■■■■■■■■■■■■■□■■■■■■■■■■■■■■■■■□□□□■□□□■■■	777767777760431	1 (0.04)	50	T1	Unique
3475*	■■■■■■■■■■■■■■□□□■■■■■■■■■■■■■□■□□□□■■■■■■■	777761777720771	2 (0.09)	66.67	H3	Clustered
3476*	■■■■■■□□□■■■■■■■■■■■■□□□■■■■■■■■■■■■■■□□■■■	770777707777631	1 (0.04)	50	Unknown	Unique
3477*	■■□■■■□■□■■■■■■□■■■■■■■■■■■□■■■■■■■■■■□□□□□	672773777377600	2 (0.09)	100	BOV_1	Clustered
3478*	■■□■■■■■□■■■■■■□■■■■□■■■■■■■■■■□□■■■■■□□□□□	676773677747600	1 (0.04)	14.29	BOV_1	Unique
3479*	■■■■■■■■□□■■□■□□□■□■■■■■■■■■■■■■□□□□■■■■■■■	776321377760771	3 (0.13)	100	S	Clustered
3480*	■■■■■■■□□□□□□□□□□□□■■■■■■■■■■■■■□□□□■■■■■■■	774000377760771	2 (0.09)	100	T1	Clustered
3481*	□■■■■■■■■■■■□□□□■■■■■■■■■■■■■■■■□□□□■■□■■■■	377703777760671	1 (0.04)	50	T1	Unique
3482*	■■■■■■■■□□■■■■■■■■■■■■■□■■■■■■■■□□□□■■■■■■■	776377767760771	1 (0.04)	50	S	Unique
3483*	■□□■■■■■■■■■■■■■■■■■■■■■■□□□□□□■□■■□□□■■■■	477777776013071	2 (0.09)	66.67	EAI3-IND	Clustered
3484*	■■■□■■■■■■■■■■■■■■■■■■□■■■■■□□□□■□■■■■■■■■■	737777757413771	1 (0.04)	50	EAI6-BGD1	Unique
3485*	□□□□□□□□□□□□□□□□□□□□■■■■■■■■■■□■□□□□■■□□■■■	000000177720631	1 (0.04)	50	H3	Unique
3486*	■■■■■■■■■■■■■□□□□■□■■■■■■■■■■■■■□□□□■■■■■■■	777741377760771	1 (0.04)	50	T4	Unique
3487*	■■■■■■■■■■■■■■■■■■■■■■■■■■■■■□□□□□□□■■■□■■■	777777777600731	1 (0.04)	50	Unknown	Unique
3488*	■■■■□■■■■□■■■■■■■■■■■■■■■□□□□□□■□□□□■■■■■■■	757377774020771	1 (0.04)	50	H1	Unique
3489*	■■■■■■■■■■■■■■■■■■■■■■□■■■□■■■■■□□□□■■■■■■■	777777756760771	1 (0.04)	50	T5	Unique
3490*	■■■■■■■■■□■■■■■■■■■■■□□□■■■■■■■■□□□□■■■■■■■	777377707760771	1 (0.04)	50	T1	Unique
3491*	■■■■■■■■□□□■■■■■■■■■□□□□■■■■■■■■□□□□■■□■■■■	776177607760671	1 (0.04)	50	LAM3	Unique
3492*	■■■■■■■■□□■■□□□□■■■■■■■■■■■■■■■■□□□□■■■■■■■	776303777760771	1 (0.04)	50	S	Unique
3493*	■■■■■■■■■■■■■■■■■■■■□■■□□□□□□□□□□□□□□■■■■■■	777777660000371	1 (0.04)	50	Unknown	Unique
3494*	■□□■■■■■■■■■■■■■■■■■■■■■■□■□□□□■□■■■■■■■■■	477777776413771	1 (0.04)	50	EAI5	Unique
3495*	■■■■■■■■■■■■□■■■■■■■■■■■■■■■■■□□□□□■■■■■■■	777737777740771	1 (0.04)	50	T3	Unique
3496*	■■■■■■■■■■■■■■■■■■■■■■■■■■■■■■□□□□□□■■■□■■■	777777777700731	1 (0.04)	50	Unknown	Unique
3497*	■■■■■■■■■■■■□□■■■■■■■■■■■■■■■■□■□□□□■■■■■■■	777717777720771	1 (0.04)	50	H3	Unique
3498*	■■■■■■□□□■■■■■■■■■■■■□□□■■■■■■■■■■■□■■□■■■■	770777707776671	1 (0.04)	50	AFRI_1	Unique
3499*	■■■■■■■■■■■■■■■■■■■■■■■■■□■■■■■■□□□□■■■□□□□	777777775760700	1 (0.04)	50	T1	Unique
3709*	■■□■■■■■□■■■■■■□□■■■□■■■■■■■■■■■■■■■■■□□□□□	676771677777600	1 (0.04)	50	BOV	Unique

* A total of 368/433 SITs containing 1976 isolates matched a preexisting shared-type in the database, whereas 65/433 SITs (n=99 isolates) were newly created. A total of 198/433 SITs containing 1840 isolates were clustered within this study (2 to 346 isolates per cluster) while 235/433 SITs containing 235 strains were unique (for total unique strains, one should add to this number the 182 orphan strains, which brings the number of unclustered isolates in this study to 417/2257 or 18.48%, and clustered isolates to 1840/2257 or 81.52%). Note that SITs followed by an asterisk indicates "newly created" SITs due to 2 or more strains belonging to an identical new pattern within this study or after a match with an orphan in the database; SIT designations followed by number of strains:
3436* this study (n=1), MAR (n=1); 3437* this study (n=2); 3438* this study (n=2); 3439* this study (n=1), ETH (n=1); 3440* this study (n=1), GUF (n=1), USA (n=1); 3441* this study (n=6); 3442* this study (n=3), FIN (n=1); 3443* this study (n=2); 3444* this study (n=1), MTQ (n=1); 3445* this study (n=1), ESP (n=1); 3446* this study (n=1), NLD (n=1); 3447* this study (n=4); 3448* this study (n=2), ITA (n=1); 3449* this study (n=3); 3450* this study (n=1), ESP (n=1); 3451* this study (n=3), FXX (n=1); 3452* this study (n=1), FXX

(n=1); 3453* this study (n=1), ETH (n=59), SWE (n=2); 3454* this study (n=2); 3455* this study (n=1), CMR (n=1); 3456* this study (n=1), SAU (n=1); 3457* this study (n=1), VEN (n=1); 3458* this study (n=1), BGD (n=1); 3459* this study (n=1), AUT (n=1); 3460* this study (n=1), THA (n=1); 3461* this study (n=2); 3462* this study (n=1), COL (n=1); 3463* this study (n=1), NLD (n=1); 3464* this study (n=1), BGD (n=1); 3465* this study (n=1), FXX (n=1); 3466* this study (n=1), SWE (n=1); 3467* this study (n=1), CMR (n=1); 3468* this study (n=2); 3469* this study (n=1), ESP (n=1); 3470* this study (n=4); 3471* this study (n=4); 3472* this study (n=1), BRA (n=1); 3473* this study (n=2), HTI (n=1); 3474* this study (n=1), HTI (n=1); 3475* this study (n=2), GMB (n=1); 3476* this study (n=1), (n=1); 3477* this study (n=2); 3478* this study (n=1), ESP (n=5), NLD (n=1); 3479* this study (n=3); 3480* this study (n=2); 3481* this study (n=1), AUT (n=1); 3482* this study (n=1), ITA (n=1); 3483* this study (n=2), MYS (n=1); 3484* this study (n=1), MYS (n=1); 3485* this study (n=1), ESP (n=1); 3486* this study (n=1), SWE (n=1); 3487* this study (n=1), JPN (n=1); 3488* this study (n=1), TUR (n=1); 3489* this study (n=1), SWE (n=1); 3490* this study (n=1), BRA (n=1); 3491* this study (n=1), ZAF (n=1); 3492* this study (n=1), USA (n=1); 3493* this study (n=1), ARG (n=1); 3494* this study (n=1), ZAF (n=1); 3495* this study (n=1), BRA (n=1); 3496* this study (n=1), PER (n=1); 3497* this study (n=1), ITA (n=1); 3498* this study (n=1), GNB (n=1); 3499* this study (n=1), ITA (n=1); 3709* this study (n=1), ESP (n=1).

** Lineage designations according to SITVIT2 using revised SpolDB4 rules; “Unknown” designates patterns with signatures that do not belong to any of the major lineages described in the database.

*** Clustered strains correspond to a similar spoligotype pattern shared by 2 or more strains “within this study”; as opposed to unique strains harboring a spoligotype pattern that does not match with another strain from this study. Unique strains matching a preexisting pattern in the SITVIT2 database are classified as SITs, whereas in case of no match, they are designated as “orphan”.
