# Supplementary material for: Combined Genotypic, Phylogenetic, and Epidemiologic Analyses of Mycobacterium tuberculosis Genetic Diversity in the Rhône Alpes Region, France
Source: PLoS One. 2016 Apr 29;11(4):e0153580. doi: 10.1371/journal.pone.0153580 (PMC4851328; doi:10.1371/journal.pone.0153580)
Supplement: S3 Table — Detailed results obtained including demographic, epidemiologic, drug-resistance, and genotyping information on a total of 2257 M. tuberculosis strains isolated in the Rhône-Alpes Region, France. (PDF) [file pone.0153580.s007.pdf]

Supplemental Table S3. Detailed results obtained including demographic, epidemiologic, drug-resistance, and genotyping information on a total of 2257 M. tuberculosis strains isolated in the Rhône-Alpes Region, France

IsoNumber	Year	Sex	Age	Spoligotype Description	Octal code	SIT*	Lineage	MIRU12	12-MIT	MIRU15	15-MIT	DST Code**	DST Comment	Origin	Type of disease***
FXX162005200606	2005	F	20	□□□□□□□□□□□□□□□□□□□□□□□□□□□□□□□□□□■■■■■■■■■	000000000003771	1	Beijing	123325153433	Orphan	233543---------	Orphan	2	INH, RIF, SM, PZA	Chechnya	EP
FXX162005100656	2005	F	49	□□□□□□□□□□□□□□□□□□□□□□□□□□□□□□□□□□■■■■■■■■■	000000000003771	1	Beijing	223325003433	Orphan	233043---------	Orphan	1		Vietnam	EP
FXX162008101559	2008	M	26	□□□□□□□□□□□□□□□□□□□□□□□□□□□□□□□□□□■■■■■■■■■	000000000003771	1	Beijing	-233---7-5-3	Orphan	233753344124544	Orphan	1		unknown	EP
FXX162010102112	2010	M	16	□□□□□□□□□□□□□□□□□□□□□□□□□□□□□□□□□□■■■■■■■■■	000000000003771	1	Beijing	-232---7-5-3	Orphan	232753446824543	Orphan	1		Afghanistan	EP
FXX162010102356	2010	F	27	□□□□□□□□□□□□□□□□□□□□□□□□□□□□□□□□□□■■■■■■■■■	000000000003771	1	Beijing	-233---7-5-3	Orphan	233753445824543	327	1		Maghreb	EP
FXX162005000711	2005	F	43	□□□□□□□□□□□□□□□□□□□□□□□□□□□□□□□□□□■■■■■■■■■	000000000003771	1	Beijing	203325173433	Orphan	033743---------	Orphan	0		unknown	NA
FXX162006000929	2006	F	28	□□□□□□□□□□□□□□□□□□□□□□□□□□□□□□□□□□■■■■■■■■■	000000000003771	1	Beijing	222321173443	1550	223743---------	Orphan	0		unknown	NA
FXX162006000931	2006	F	76	□□□□□□□□□□□□□□□□□□□□□□□□□□□□□□□□□□■■■■■■■■■	000000000003771	1	Beijing	222321173443	1550	223743---------	Orphan	0		unknown	NA
FXX162006000936	2006	M	39	□□□□□□□□□□□□□□□□□□□□□□□□□□□□□□□□□□■■■■■■■■■	000000000003771	1	Beijing	222321173443	1550	223743---------	Orphan	0		unknown	NA
FXX162007001163	2007	M	17	□□□□□□□□□□□□□□□□□□□□□□□□□□□□□□□□□□■■■■■■■■■	000000000003771	1	Beijing	222325163544	282	223654---------	Orphan	0		unknown	NA
FXX162006000995	2006	M	36	□□□□□□□□□□□□□□□□□□□□□□□□□□□□□□□□□□■■■■■■■■■	000000000003771	1	Beijing	222325173433	Orphan	223743---------	Orphan	0		unknown	NA
FXX162005000727	2005	F	34	□□□□□□□□□□□□□□□□□□□□□□□□□□□□□□□□□□■■■■■■■■■	000000000003771	1	Beijing	223325163433	376	233643---------	Orphan	0		unknown	NA
FXX162007001266	2007	M	30	□□□□□□□□□□□□□□□□□□□□□□□□□□□□□□□□□□■■■■■■■■■	000000000003771	1	Beijing	223325163533	83	233653---------	Orphan	0		unknown	NA
FXX162007001154	2007	M	53	□□□□□□□□□□□□□□□□□□□□□□□□□□□□□□□□□□■■■■■■■■■	000000000003771	1	Beijing	223325173333	721	233733---------	Orphan	0		unknown	NA
FXX162005000607	2005	M	26	□□□□□□□□□□□□□□□□□□□□□□□□□□□□□□□□□□■■■■■■■■■	000000000003771	1	Beijing	223325173433	86	233743---------	Orphan	0		unknown	NA
FXX162006000996	2006	M	17	□□□□□□□□□□□□□□□□□□□□□□□□□□□□□□□□□□■■■■■■■■■	000000000003771	1	Beijing	223325173433	86	233743---------	Orphan	0		unknown	NA
FXX162007001244	2007	M	61	□□□□□□□□□□□□□□□□□□□□□□□□□□□□□□□□□□■■■■■■■■■	000000000003771	1	Beijing	223325173533	17	233753---------	Orphan	0		unknown	NA
FXX162007001247	2007	F	8	□□□□□□□□□□□□□□□□□□□□□□□□□□□□□□□□□□■■■■■■■■■	000000000003771	1	Beijing	22332-173533	Orphan	233753---------	Orphan	0		unknown	NA
FXX162005100602	2005	M	27	□□□□□□□□□□□□□□□□□□□□□□□□□□□□□□□□□□■■■■■■■■■	000000000003771	1	Beijing	223-25173433	Orphan	23-743---------	Orphan	1		unknown	NA
FXX162007001311	2007	M	25	□□□□□□□□□□□□□□□□□□□□□□□□□□□□□□□□□□■■■■■■■■■	000000000003771	1	Beijing	-223---7-5-3	Orphan	223753442824523	Orphan	0		unknown	NA
FXX162010002233	2010	M	29	□□□□□□□□□□□□□□□□□□□□□□□□□□□□□□□□□□■■■■■■■■■	000000000003771	1	Beijing	-223---7-5-3	Orphan	223753446824543	Orphan	0		unknown	NA
FXX162010002127	2010	M	28	□□□□□□□□□□□□□□□□□□□□□□□□□□□□□□□□□□■■■■■■■■■	000000000003771	1	Beijing	-233---7-3-2	Orphan	233732247842443	Orphan	0		unknown	NA
FXX162010002153	2010	M	57	□□□□□□□□□□□□□□□□□□□□□□□□□□□□□□□□□□■■■■■■■■■	000000000003771	1	Beijing	-233---7-5-3	Orphan	233753355824623	Orphan	0		unknown	NA
FXX162010002052	2010	M	55	□□□□□□□□□□□□□□□□□□□□□□□□□□□□□□□□□□■■■■■■■■■	000000000003771	1	Beijing	-233---5-5-3	Orphan	233553445824544	Orphan	0		unknown	NA
FXX162009002066	2009	M	51	□□□□□□□□□□□□□□□□□□□□□□□□□□□□□□□□□□■■■■■■■■■	000000000003771	1	Beijing	-233---6-5-3	Orphan	233653446724443	Orphan	0		unknown	NA
FXX162007001312	2007	M	26	□□□□□□□□□□□□□□□□□□□□□□□□□□□□□□□□□□■■■■■■■■■	000000000003771	1	Beijing	-233---7-5-3	Orphan	233753445824543	327	0		unknown	NA
FXX162008001607	2008	M	25	□□□□□□□□□□□□□□□□□□□□□□□□□□□□□□□□□□■■■■■■■■■	000000000003771	1	Beijing	-233---7-5-3	Orphan	233753446822544	Orphan	0		unknown	NA
FXX162009001778	2009	F	37	□□□□□□□□□□□□□□□□□□□□□□□□□□□□□□□□□□■■■■■■■■■	000000000003771	1	Beijing	-233---7-5-3	Orphan	233753445824543	327	0		unknown	NA
FXX162009001889	2009	M	68	□□□□□□□□□□□□□□□□□□□□□□□□□□□□□□□□□□■■■■■■■■■	000000000003771	1	Beijing	-233---7-5-3	Orphan	233753445824543	327	0		unknown	NA
FXX162005100661	2005	M	27	□□□□□□□□□□□□□□□□□□□□□□□□□□□□□□□□□□■■■■■■■■■	000000000003771	1	Beijing	221325183433	Orphan	213843---------	Orphan	1		China	P
FXX162006101011	2006	M	24	□□□□□□□□□□□□□□□□□□□□□□□□□□□□□□□□□□■■■■■■■■■	000000000003771	1	Beijing	223325173533	17	233753444-24543	Orphan	1		unknown	P
FXX162006100690	2006	M	38	□□□□□□□□□□□□□□□□□□□□□□□□□□□□□□□□□□■■■■■■■■■	000000000003771	1	Beijing	223125173233	Orphan	231723---------	Orphan	1		France	P
FXX162006100834	2006	M	50	□□□□□□□□□□□□□□□□□□□□□□□□□□□□□□□□□□■■■■■■■■■	000000000003771	1	Beijing	223325153433	592	233543---------	Orphan	1		Armenia	P
FXX162006301058	2006	M	36	□□□□□□□□□□□□□□□□□□□□□□□□□□□□□□□□□□■■■■■■■■■	000000000003771	1	Beijing	223325153533	16	233553---------	Orphan	3	SM	Azerbaijan	P
FXX162003100257	2003	M	41	□□□□□□□□□□□□□□□□□□□□□□□□□□□□□□□□□□■■■■■■■■■	000000000003771	1	Beijing	223325163333	1133	233633---------	Orphan	1		Senegal	P
FXX162005100538	2005	M	44	□□□□□□□□□□□□□□□□□□□□□□□□□□□□□□□□□□■■■■■■■■■	000000000003771	1	Beijing	223325163333	1133	233633---------	Orphan	1		France	P
FXX162006100782	2006	M	27	□□□□□□□□□□□□□□□□□□□□□□□□□□□□□□□□□□■■■■■■■■■	000000000003771	1	Beijing	223325163433	376	233643---------	Orphan	1		India	P
FXX162006100765	2006	F	49	□□□□□□□□□□□□□□□□□□□□□□□□□□□□□□□□□□■■■■■■■■■	000000000003771	1	Beijing	223325173433	86	233743---------	Orphan	1		France	P
FXX162006300829	2006	M	24	□□□□□□□□□□□□□□□□□□□□□□□□□□□□□□□□□□■■■■■■■■■	000000000003771	1	Beijing	223325173433	86	233743---------	Orphan	3	SM, INH	China	P
FXX162006100837	2006	M	18	□□□□□□□□□□□□□□□□□□□□□□□□□□□□□□□□□□■■■■■■■■■	000000000003771	1	Beijing	223325173433	86	233743---------	Orphan	1		France	P
FXX162006100976	2006	F	34	□□□□□□□□□□□□□□□□□□□□□□□□□□□□□□□□□□■■■■■■■■■	000000000003771	1	Beijing	223325173433	86	233743---------	Orphan	1		unknown	P
FXX162006000986	2006	M	28	□□□□□□□□□□□□□□□□□□□□□□□□□□□□□□□□□□■■■■■■■■■	000000000003771	1	Beijing	223325173433	86	233743---------	Orphan	0		France	P
FXX162006001022	2006	F	29	□□□□□□□□□□□□□□□□□□□□□□□□□□□□□□□□□□■■■■■■■■■	000000000003771	1	Beijing	223325173433	86	233743---------	Orphan	0		France	P
FXX162006100780	2006	M	23	□□□□□□□□□□□□□□□□□□□□□□□□□□□□□□□□□□■■■■■■■■■	000000000003771	1	Beijing	323325173633	Orphan	233763---------	Orphan	1		Korea	P
FXX162010202197	2010	M	30	□□□□□□□□□□□□□□□□□□□□□□□□□□□□□□□□□□■■■■■■■■■	000000000003771	1	Beijing	-233---5-4-3	Orphan	233543446824523	Orphan	2	SM, RIF, INH, ETH, PZA	unknown	P
FXX162009201720	2009	M	18	□□□□□□□□□□□□□□□□□□□□□□□□□□□□□□□□□□■■■■■■■■■	000000000003771	1	Beijing	-233---5-4-4	Orphan	233544442724543	Orphan	2	SM, RIF,INH,ETH,PZA	unknown	P
FXX162010102194	2010	M	37	□□□□□□□□□□□□□□□□□□□□□□□□□□□□□□□□□□■■■■■■■■■	000000000003771	1	Beijing	-233---5-5-3	Orphan	233553446824543	142	1		unknown	P
FXX162009101971	2009	M	19	□□□□□□□□□□□□□□□□□□□□□□□□□□□□□□□□□□■■■■■■■■■	000000000003771	1	Beijing	-233---7-2-3	Orphan	233723444824543	Orphan	1		unknown	P
FXX162010102023	2010	M	44	□□□□□□□□□□□□□□□□□□□□□□□□□□□□□□□□□□■■■■■■■■■	000000000003771	1	Beijing	-233---7-3-3	Orphan	23373344-334-43	Orphan	1		unknown	P
FXX162009101805	2009	M	48	□□□□□□□□□□□□□□□□□□□□□□□□□□□□□□□□□□■■■■■■■■■	000000000003771	1	Beijing	-233---7-4-3	Orphan	233743446824543	Orphan	1		unknown	P
FXX162008101593	2008	M	21	□□□□□□□□□□□□□□□□□□□□□□□□□□□□□□□□□□■■■■■■■■■	000000000003771	1	Beijing	-233---7-5-3	Orphan	233753445825543	Orphan	1		unknown	P
FXX162009101823	2009	M	33	□□□□□□□□□□□□□□□□□□□□□□□□□□□□□□□□□□■■■■■■■■■	000000000003771	1	Beijing	-233---7-5-3	Orphan	233753446A44443	Orphan	1		unknown	P
FXX162009101934	2009	F	39	□□□□□□□□□□□□□□□□□□□□□□□□□□□□□□□□□□■■■■■■■■■	000000000003771	1	Beijing	-233---7-5-3	Orphan	233753447A44443	Orphan	1		Thailand	P
FXX162010102242	2010	M	37	□□□□□□□□□□□□□□□□□□□□□□□□□□□□□□□□□□■■■■■■■■■	000000000003771	1	Beijing	-233---7-5-3	Orphan	233753445824543	327	1		Algeria	P

FXX162001100062	2001	F	27	□□□□□□□□□□□□□□□□□□□□□□□□□□□□□□□□□□■■■■■■■■■	000000000003771	1	Beijing					1		South Korea	P
FXX162002200066	2002	F	26	□□□□□□□□□□□□□□□□□□□□□□□□□□□□□□□□□□■■■■■■■■■	000000000003771	1	Beijing					2	SM, RIF,INH, ETH	Yugoslavia	P
FXX162001300099	2001	F	42	□□□□□□□□□□□□□□□□□□□□□□□□□□□□□□□□□□■■■■■■■■■	000000000003771	1	Beijing					3	SM, INH , ETH	Albania	P
FXX162001300100	2001	F	27	□□□□□□□□□□□□□□□□□□□□□□□□□□□□□□□□□□■■■■■■■■■	000000000003771	1	Beijing					3	SM	Cambodia	P
FXX162002100182	2002	M	77	□□□□□□□□□□□□□□□□□□□□□□□□□□□□□□□□□□■■■■■■■■■	000000000003771	1	Beijing					1		Poland	P
FXX162003100312	2003	M	43	□□□□□□□□□□□□□□□□□□□□□□□□□□□□□□□□□□■■■■■■■■■	000000000003771	1	Beijing					1		France	P
FXX162003100319	2003	M	57	□□□□□□□□□□□□□□□□□□□□□□□□□□□□□□□□□□■■■■■■■■■	000000000003771	1	Beijing					1		Algeria	P
FXX162000000397	2000	M	42	□□□□□□□□□□□□□□□□□□□□□□□□□□□□□□□□□□■■■■■■■■■	000000000003771	1	Beijing					0		Cambodia	P
FXX162004100482	2004	M	25	□□□□□□□□□□□□□□□□□□□□□□□□□□□□□□□□□□■■■■■■■■■	000000000003771	1	Beijing					1		Armenia	P
FXX162004300487	2004	M	35	□□□□□□□□□□□□□□□□□□□□□□□□□□□□□□□□□□■■■■■■■■■	000000000003771	1	Beijing					3	SM	Vietnam	P
FXX162004300537	2004	M	76	□□□□□□□□□□□□□□□□□□□□□□□□□□□□□□□□□□■■■■■■■■■	000000000003771	1	Beijing					3	SM	France	P
FXX162007201134	2007	F	32	□□□□□□□□□□□□□□□□□□□□□□□□□□□□□□□□□□■■■■■■■■■	000000000003771	1	Beijing	223325153533	16	233553446824543	142	2	SM, RIF, INH	Chechnya	P
FXX162008101543	2008	M	19	□□□□□□□□□□□□□□□□□□□□□□□□□□□□□□□□□□■■■■■■■■■	000000000003771	1	Beijing	-233---7-5-3	Orphan	233753345824443	Orphan	1		unknown	P
FXX162008101597	2008	M	26	□□□□□□□□□□□□□□□□□□□□□□□□□□□□□□□□□□■■■■■■■■■	000000000003771	1	Beijing	-233---5-5-3	Orphan	233553444833441	Orphan	1		unknown	P
FXX162008201374	2008	M	36	□□□□□□□□□□□□□□□□□□□□□□□□□□□□□□□□□□■■■■■■■■■	000000000003771	1	Beijing	-233---7-5-3	Orphan	233753446824643	Orphan	2	RIF, INH, SM, PZA	Moldova	P
FXX162008101625	2008	F	34	□□□□□□□□□□□□□□□□□□□□□□□□□□□□□□□□□□■■■■■■■■■	000000000003771	1	Beijing	-233---7-5-3	Orphan	233753444824543	Orphan	1		unknown	P
FXX162007101322	2007	M	42	□□□□□□□□□□□□□□□□□□□□□□□□□□□□□□□□□□■■■■■■■■■	000000000003771	1	Beijing	-233---7-5-3	Orphan	233753-45824543	Orphan	1		Algeria	P
FXX162008201395	2008	M	29	□□□□□□□□□□□□□□□□□□□□□□□□□□□□□□□□□□■■■■■■■■■	000000000003771	1	Beijing	-233---5-5-3	Orphan	233553446624543	Orphan	2	SM, RIF, INH,ETH, PZA	unknown	EP and P
FXX162006000863	2006	M	44	□□□□□□□□□□□□□□□□□□□□□□□□■□□□□□□■□□□□■■■■■■■	000000004020771	2	H2	223313153223	Orphan	233523---------	Orphan	0		unknown	NA
FXX162007001191	2007	M	29	□□□□□□□□□□□□□□□□□□□□□□□□■□□□□□□■□□□□■■■■■■■	000000004020771	2	H2	225--315-323	Orphan	25-533---------	Orphan	0		unknown	NA
FXX162009001914	2009	M	77	□□□□□□□□□□□□□□□□□□□□□□□□■□□□□□□■□□□□■■■■■■■	000000004020771	2	H2	-253---5-3-3	Orphan	253533334722243	152	0		unknown	NA
FXX162010002173	2010	F	69	□□□□□□□□□□□□□□□□□□□□□□□□■□□□□□□■□□□□■■■■■■■	000000004020771	2	H2	-253---5-3-3	Orphan	253533334732343	369	0		unknown	NA
FXX162005300604	2005	M	57	□□□□□□□□□□□□□□□□□□□□□□□□■□□□□□□■□□□□■■■■■■■	000000004020771	2	H2	225323153323	43	253533---------	Orphan	3	ETH	Romania	P
FXX162006101044	2006	M	36	□□□□□□□□□□□□□□□□□□□□□□□□■□□□□□□■□□□□■■■■■■■	000000004020771	2	H2	225325153323	45	253533---------	Orphan	1		France	P
FXX162010102198	2010	M	40	□□□□□□□□□□□□□□□□□□□□□□□□■□□□□□□■□□□□■■■■■■■	000000004020771	2	H2	-253---5-3-3	Orphan	253533336722244	372	1		unknown	P
FXX162010102249	2010	M	40	□□□□□□□□□□□□□□□□□□□□□□□□■□□□□□□■□□□□■■■■■■■	000000004020771	2	H2	-253---5-3-3	Orphan	253533334732343	369	1		France	P
FXX162000300328	2000	M	44	□□□□□□□□□□□□□□□□□□□□□□□□■□□□□□□■□□□□■■■■■■■	000000004020771	2	H2					3	SM	France	P
FXX162008101601	2008	M	65	□□□□□□□□□□□□□□□□□□□□□□□□■□□□□□□■□□□□■■■■■■■	000000004020771	2	H2	-253---5-3-3	Orphan	253533336722243	359	1		unknown	P
FXX162008101619	2008	F	29	□□□□□□□□□□□□□□□□□□□□□□□□■□□□□□□■□□□□■■■■■■■	000000004020771	2	H2	-263---5-3-3	Orphan	263533336722243	Orphan	1		unknown	P
FXX162000000150	2000	M	63	□□□□□□□□□□□□□□□□□□□□□□□□■■■■■■□■□□□□■■■■■■■	000000007720771	3	H3					0		France	EP
FXX162004100496	2004	M	84	□□□□□□□□□□□□□□□□□□□□□□□□■■■■■■□■□□□□■■■■■■■	000000007720771	3	H3					1		France	EP
FXX162006001048	2006	M	64	□□□□□□□□□□□□□□□□□□□□□□□□■■■■■■□■□□□□■■■■■■■	000000007720771	3	H3	224313153323	240	243533---------	Orphan	0		unknown	NA
FXX162008001463	2008	M	29	□□□□□□□□□□□□□□□□□□□□□□□□■■■■■■□■□□□□■■■■■■■	000000007720771	3	H3	-233---5-2-3	Orphan	233523334632343	Orphan	0		unknown	NA
FXX162009001751	2009	F	58	□□□□□□□□□□□□□□□□□□□□□□□□■■■■■■□■□□□□■■■■■■■	000000007720771	3	H3	-253---5-3-1	Orphan	253531336732343	Orphan	0		unknown	NA
FXX162008001681	2008	F	81	□□□□□□□□□□□□□□□□□□□□□□□□■■■■■■□■□□□□■■■■■■■	000000007720771	3	H3	-253---5-3-3	Orphan	253533334722243	152	0		unknown	NA
FXX162003100423	2003	F	89	□□□□□□□□□□□□□□□□□□□□□□□□■■■■■■□■□□□□■■■■■■■	000000007720771	3	H3					1		France	P
FXX162002100197	2002	M	25	□□□□□□□□□□□□□□□□□□□□□□□□■■■■■■■■□□□□■■■■■■■	000000007760771	4	Unknown					1		Algeria	EP
FXX162007001281	2007	M	56	□□□□□□□□□□□□□□□□□□□□□□□□■■■■■■■■□□□□■■■■■■■	000000007760771	4	Unknown	133325153324	1549	333534---------	Orphan	0		unknown	NA
FXX162007001088	2007	M	60	□□□□□□□□□□□□□□□□□□□□□□□□■■■■■■■■□□□□■■■■■■■	000000007760771	4	Unknown	233325153324	212	333534---------	Orphan	0		unknown	NA
FXX162010002142	2010	F	84	□□□□□□□□□□□□□□□□□□□□□□□□■■■■■■■■□□□□■■■■■■■	000000007760771	4	Unknown	-233---6-3-2	Orphan	233632243523223	Orphan	0		unknown	NA
FXX162010002292	2010	M	26	□□□□□□□□□□□□□□□□□□□□□□□□■■■■■■■■□□□□■■■■■■■	000000007760771	4	Unknown	-232---4-3-4	Orphan	232434334422223	Orphan	0		unknown	NA
FXX162010002302	2010	F	55	□□□□□□□□□□□□□□□□□□□□□□□□■■■■■■■■□□□□■■■■■■■	000000007760771	4	Unknown	-232---5-3-1	Orphan	232531345513225	Orphan	0		unknown	NA
FXX162008001610	2008	M	88	□□□□□□□□□□□□□□□□□□□□□□□□■■■■■■■■□□□□■■■■■■■	000000007760771	4	Unknown	-323---2-4-4	Orphan	323244444523125	Orphan	0		unknown	NA
FXX162008001540	2008	F	38	□□□□□□□□□□□□□□□□□□□□□□□□■■■■■■■■□□□□■■■■■■■	000000007760771	4	Unknown	-323---5-3-4	Orphan	323534445-23124	Orphan	0		unknown	NA
FXX162008101489	2008	M	70	□□□□□□□□□□□□□□□□□□□□□□□□■■■■■■■■□□□□■■■■■■■	000000007760771	4	Unknown	-233---6-3-2	Orphan	233632-43-23223	Orphan	1		unknown	P
FXX162008001716	2008	F	3	□□□□□□□□□■■□■■■■■■■■■■■■■■■■■■■■□□□□■■■■■■■	000677777760771	5	T1	-233---5-3-2	Orphan	233532453421324	Orphan	0		unknown	NA
FXX162000300334	2000	M	75	□■■■■■■■■■■■■■■■■■■■■■■■■■■■■■■□□□□■■■■■■■	377777777760771	7	T1					3	PZA	France	EP
FXX162006101043	2006	M	54	□■■■■■■■■■■■■■■■■■■■■■■■■■■■■■■□□□□■■■■■■■	377777777760771	7	T1	223128153325	Orphan	231535---------	Orphan	1		Turkey	P
FXX162009301809	2009	F	49	□■■■■■■■■■■■■■■■■■■■■■■■■■■■■■■□□□□■■■■■■■	377777777760771	7	T1	-234---4-3-2	Orphan	234432244522224	Orphan	3	SM	unknown	P
FXX162009101748	2009	M	47	□■■■■■■■■■■■■■■■■■■■■■■■■■■■■■■□□□□■■■■■■■	377777777760771	7	T1	-231---5-3-5	Orphan	231535342522223	Orphan	1		Turkey	P
FXX162000000372	2000	M	51	□■■■■■■■■■■■■■■■■■■■■■■■■■■■■■■□□□□■■■■■■■	377777777760771	7	T1					0		Algeria	P
FXX162007101213	2007	M	60	■□□■■■■■■■■■■■■■■■■■■■■■■■■■□□□□■□■■□□□■■■■	477777777413071	11	EAI3-IND	253324223524	Orphan	533254---------	Orphan	1		India	EP
FXX162004100459	2004	M	31	■□□■■■■■■■■■■■■■■■■■■■■■■■■■□□□□■□■■□□□■■■■	477777777413071	11	EAI3-IND					1		Senegal	EP
FXX162009101814	2009	M	39	■□□■■■■■■■■■■■■■■■■■■■■■■■■■□□□□■□■■□□□■■■■	477777777413071	11	EAI3-IND	-543---2-4-4	Orphan	543244643412614	Orphan	1		Réunion	P
FXX162003100135	2003	M	17	■■□■■■■■■■■■□■■■■■■■□□□□■■■■■■■■□□□□■■■■■■■	677737607760771	17	LAM2					1		Angola	EP
FXX162009001899	2009	M	42	■■□■■■■■■■■■□■■■■■■■□□□□■■■■■■■■□□□□■■■■■■■	677737607760771	17	LAM2	-242---5-3-1	Orphan	242531244723312	Orphan	0		unknown	NA
FXX162004100439	2004	M	29	■■□■■■■■■■■■□■■■■■■■□□□□■■■■■■■■□□□□■■■■■■■	677737607760771	17	LAM2					1		Angola	P

FXX162009001881	2009	M	27	■■□■■■■■■■■■■■■■■■■□□■■■■■■■□□□□■□■■■■■■■■■	677777477413771	19	EAI2-Manila	-541---2-4-2	Orphan	54124244A711A22	Orphan	0		unknown	NA
FXX162010002165	2010	F	51	■■□■■■■■■■■■■■■■■■■□□■■■■■■■□□□□■□■■■■■■■■■	677777477413771	19	EAI2-Manila	-543---2-4-2	Orphan	543242447511A23	Orphan	0		unknown	NA
FXX162005100576	2005	F	24	■■□■■■■■■■■■■■■■■■■□□■■■■■■■□□□□■□■■■■■■■■■	677777477413771	19	EAI2-Manila	252326223432	Orphan	523242---------	Orphan	1		Vietnam	P
FXX162000000401	2000	M	19	■■□■■■■■■■■■■■■■■■■□□■■■■■■■□□□□■□■■■■■■■■■	677777477413771	19	EAI2-Manila					0		Vietnam	P
FXX162001300013	2001	M	71	■■□■■■■■■■■■■■■■■■■■□□□□■■■■■■■■□□□□■■■■■■■	677777607760771	20	LAM1	-232---5-3-1	Orphan	23253124---1---	Orphan	3	SM	Algeria	EP
FXX162002300210	2002	M	37	■■□■■■■■■■■■■■■■■■■■□□□□■■■■■■■■□□□□■■■■■■■	677777607760771	20	LAM1	-232---5-3-1	Orphan	232531242421312	224	3	SM	Congo	EP
FXX162005000543	2005	M	4	■■□■■■■■■■■■■■■■■■■■□□□□■■■■■■■■□□□□■■■■■■■	677777607760771	20	LAM1	223226153321	128	232531242401312	Orphan	0		unknown	NA
FXX162006000861	2006	M	38	■■□■■■■■■■■■■■■■■■■■□□□□■■■■■■■■□□□□■■■■■■■	677777607760771	20	LAM1	223226153321	128	232531242421312	224	0		unknown	NA
FXX162007001272	2007	M	60	■■□■■■■■■■■■■■■■■■■■□□□□■■■■■■■■□□□□■■■■■■■	677777607760771	20	LAM1	223226153321	128	232531242421312	224	0		unknown	NA
FXX162006000768	2006	M	45	■■□■■■■■■■■■■■■■■■■■□□□□■■■■■■■■□□□□■■■■■■■	677777607760771	20	LAM1	223226153321	128	232531---------	Orphan	0		unknown	NA
FXX162007001237	2007	F	67	■■□■■■■■■■■■■■■■■■■■□□□□■■■■■■■■□□□□■■■■■■■	677777607760771	20	LAM1	323226163221	Orphan	232621244723312	Orphan	0		unknown	NA
FXX162008001419	2008	M	64	■■□■■■■■■■■■■■■■■■■■□□□□■■■■■■■■□□□□■■■■■■■	677777607760771	20	LAM1	-232---5-3-1	Orphan	232531242421312	224	0		unknown	NA
FXX162008001718	2008	M	49	■■□■■■■■■■■■■■■■■■■■□□□□■■■■■■■■□□□□■■■■■■■	677777607760771	20	LAM1	-232---5-3-1	Orphan	232531242421312	224	0		unknown	NA
FXX162010001956	2010	M	41	■■□■■■■■■■■■■■■■■■■■□□□□■■■■■■■■□□□□■■■■■■■	677777607760771	20	LAM1	-232---5-3-1	Orphan	232531242421312	224	0		unknown	NA
FXX162010002104	2010	M	34	■■□■■■■■■■■■■■■■■■■■□□□□■■■■■■■■□□□□■■■■■■■	677777607760771	20	LAM1	-232---5-3-1	Orphan	232531242421312	224	0		unknown	NA
FXX162010002344	2010	F	26	■■□■■■■■■■■■■■■■■■■■□□□□■■■■■■■■□□□□■■■■■■■	677777607760771	20	LAM1	-232---6-3-1	Orphan	232631243624312	Orphan	0		unknown	NA
FXX162009001924	2009	M	25	■■□■■■■■■■■■■■■■■■■■□□□□■■■■■■■■□□□□■■■■■■■	677777607760771	20	LAM1	-242---5-3-1	Orphan	242531243724312	330	0		unknown	NA
FXX162010002291	2010	M	22	■■□■■■■■■■■■■■■■■■■■□□□□■■■■■■■■□□□□■■■■■■■	677777607760771	20	LAM1	-242---5-3-1	Orphan	242531243724312	330	0		unknown	NA
FXX162006000825	2006	M	25	■■□■■■■■■■■■■■■■■■■■□□□□■■■■■■■■□□□□■■■■■■■	677777607760771	20	LAM1	223226153321	128	232531242421312	224	0		unknown	P
FXX162001300084	2001	M	63	■■□■■■■■■■■■■■■■■■■■□□□□■■■■■■■■□□□□■■■■■■■	677777607760771	20	LAM1	-232---5-3-1	Orphan	232531242421312	224	3	SM	France	P
FXX162002300169	2002	M	33	■■□■■■■■■■■■■■■■■■■■□□□□■■■■■■■■□□□□■■■■■■■	677777607760771	20	LAM1	-232---5-3-1	Orphan	232531242421312	224	3	SM	Portugal	P
FXX162002300199	2002	M	44	■■□■■■■■■■■■■■■■■■■■□□□□■■■■■■■■□□□□■■■■■■■	677777607760771	20	LAM1	-232---5-3-1	Orphan	232531242421312	224	3	SM	France	P
FXX162002300203	2002	M	17	■■□■■■■■■■■■■■■■■■■■□□□□■■■■■■■■□□□□■■■■■■■	677777607760771	20	LAM1	-232---5-3-1	Orphan	232531242421312	224	3	SM	Senegal	P
FXX162002000253	2002	F	64	■■□■■■■■■■■■■■■■■■■■□□□□■■■■■■■■□□□□■■■■■■■	677777607760771	20	LAM1	-232---5-3-1	Orphan	232531242421312	224	0		Angola	P
FXX162009101764	2009	M	29	■■□■■■■■■■■■■■■■■■■■□□□□■■■■■■■■□□□□■■■■■■■	677777607760771	20	LAM1	-242---5-3-1	Orphan	242531243724312	330	1		unknown	P
FXX162009101793	2009	M	29	■■□■■■■■■■■■■■■■■■■■□□□□■■■■■■■■□□□□■■■■■■■	677777607760771	20	LAM1	-242---5-3-1	Orphan	242531243724312	330	1		unknown	P
FXX162009101801	2009	M	51	■■□■■■■■■■■■■■■■■■■■□□□□■■■■■■■■□□□□■■■■■■■	677777607760771	20	LAM1	-242---5-3-1	Orphan	242531244824312	331	1		unknown	P
FXX162010101961	2010	F	1	■■□■■■■■■■■■■■■■■■■■□□□□■■■■■■■■□□□□■■■■■■■	677777607760771	20	LAM1	-242---5-3-1	Orphan	242531243724312	330	1		unknown	P
FXX162010102059	2010	M	27	■■□■■■■■■■■■■■■■■■■■□□□□■■■■■■■■□□□□■■■■■■■	677777607760771	20	LAM1	-242---5-3-1	Orphan	242531243724312	330	1		unknown	P
FXX162010102097	2010	M	35	■■□■■■■■■■■■■■■■■■■■□□□□■■■■■■■■□□□□■■■■■■■	677777607760771	20	LAM1	-242---5-3-1	Orphan	242531243724312	330	1		Chechnya	P
FXX162010102192	2010	M	62	■■□■■■■■■■■■■■■■■■■■□□□□■■■■■■■■□□□□■■■■■■■	677777607760771	20	LAM1	-243---7-4-4	Orphan	243744243524224	Orphan	1		Algeria	P
FXX162001300081	2001	M	28	■■□■■■■■■■■■■■■■■■■■□□□□■■■■■■■■□□□□■■■■■■■	677777607760771	20	LAM1					3	SM et INH	Congo	P
FXX162007001070	2007	M	55	■■□■■■■■■■■■■■■■■■■■□□□□■■■■■■■■□□□□■■■■■■■	677777607760771	20	LAM1	223226153321	128	232531242421312	224	0		Gulf of Guinea	P
FXX162007301211	2007	M	27	■■□■■■■■■■■■■■■■■■■■□□□□■■■■■■■■□□□□■■■■■■■	677777607760771	20	LAM1	324224171321	Orphan	2427312433-3312	Orphan	3	SM	Bulgaria	P
FXX162007101340	2007	M	51	■■□■■■■■■■■■■■■■■■■■□□□□■■■■■■■■□□□□■■■■■■■	677777607760771	20	LAM1	-242---5-3-1	Orphan	242531244-24312	Orphan	1		unknown	P
FXX162005300567	2005	M	30	■■■□□□□■■□■■■■■■■■■□□□□□□□□□□□□□□□□■■■■■■■■	703377400001771	21	CAS1-Kili	225325113434	Orphan	253144---------	Orphan	3	INH	Madagascar	EP
FXX162010102111	2010	F	19	■■■□□□□■■□■■■■■■■■■□□□□□□□□□□□□□□□□■■■■■■■■	703377400001771	21	CAS1-Kili	-274---1-4-4	Orphan	274144422544322	138	1		Comoros	EP
FXX162010102169	2010	F	10	■■■□□□□■■□■■■■■■■■■□□□□□□□□□□□□□□□□■■■■■■■■	703377400001771	21	CAS1-Kili	-274---1-4-4	Orphan	274144422544322	138	1		unknown	EP
FXX162008001510	2008	F	33	■■■□□□□■■□■■■■■■■■■□□□□□□□□□□□□□□□□■■■■■■■■	703377400001771	21	CAS1-Kili	-274---1-4-4	Orphan	274144422544322	138	0		unknown	NA
FXX162010002048	2010	F	27	■■■□□□□■■□■■■■■■■■■□□□□□□□□□□□□□□□□■■■■■■■■	703377400001771	21	CAS1-Kili	-284---1-4-4	Orphan	284144422524322	Orphan	0		unknown	NA
FXX162007301215	2007	F	15	■■■□□□□■■□■■■■■■■■■□□□□□□□□□□□□□□□□■■■■■■■■	703377400001771	21	CAS1-Kili	222425113434	1556	224144---------	Orphan	3	SM	Comoros	P
FXX162007101202	2007	F	21	■■■□□□□■■□■■■■■■■■■□□□□□□□□□□□□□□□□■■■■■■■■	703377400001771	21	CAS1-Kili	226425113434	1583	264144---------	Orphan	1		Madagascar	P
FXX162004100524	2004	M	54	■■■□□□□■■■■■■■■■■■■■■■□□□□□□□□□□□□■■□□■■■■■	703777740003171	25	CAS1-Delhi	226325163432	Orphan	263642---------	Orphan	1		Djibouti	EP
FXX162008001387	2008	F	38	■■■□□□□■■■■■■■■■■■■■■■□□□□□□□□□□□□■■□□■■■■■	703777740003171	25	CAS1-Delhi	-263---6-5-3	Orphan	263653422-43423	Orphan	0		unknown	NA
FXX162008001403	2008	M	38	■■■□□□□■■■■■■■■■■■■■■■□□□□□□□□□□□□■■□□■■■■■	703777740003171	25	CAS1-Delhi	-2-4---5-5-3	Orphan	2-4553422143423	Orphan	0		unknown	NA
FXX162008101649	2008	M	35	■■■□□□□■■■■■■■■■■■■■■■□□□□□□□□□□□□■■□□■■■■■	703777740003171	25	CAS1-Delhi	-365---5-5-3	Orphan	365553422843423	Orphan	1		unknown	P
FXX162000000403	2000	M	26	■■■□□□□■■■■■■■■■■■■■■■□□□□□□□□□□□□■■■■■■■■■	703777740003771	26	CAS1-Delhi					0		Pakistan	EP
FXX162006000808	2006	F	28	■■■□□□□■■■■■■■■■■■■■■■□□□□□□□□□□□□■■■■■■■■■	703777740003771	26	CAS1-Delhi	225325163433	Orphan	253643---------	Orphan	0		unknown	NA
FXX162007001156	2007	M	29	■■■□□□□■■■■■■■■■■■■■■■□□□□□□□□□□□□■■■■■■■■■	703777740003771	26	CAS1-Delhi	225425173433	410	254743---------	Orphan	0		unknown	NA
FXX162007001136	2007	M	26	■■■□□□□■■■■■■■■■■■■■■■□□□□□□□□□□□□■■■■■■■■■	703777740003771	26	CAS1-Delhi	225425173533	68	254753---------	Orphan	0		China (Tibet)	NA
FXX162009002042	2009	F	34	■■■□□□□■■■■■■■■■■■■■■■□□□□□□□□□□□□■■■■■■■■■	703777740003771	26	CAS1-Delhi	-253---6-5-3	Orphan	253653422434423	Orphan	0		unknown	NA
FXX162008001737	2008	M	54	■■■□□□□■■■■■■■■■■■■■■■□□□□□□□□□□□□■■■■■■■■■	703777740003771	26	CAS1-Delhi	-254---7-5-3	Orphan	254753422845422	Orphan	0		unknown	NA
FXX162008001684	2008	M	26	■■■□□□□■■■■■■■■■■■■■■■□□□□□□□□□□□□■■■■■■■■■	703777740003771	26	CAS1-Delhi	-265---7-5-2	Orphan	265752322848423	Orphan	0		unknown	NA
FXX162003100305	2003	F	38	■■■□□□□■■■■■■■■■■■■■■■□□□□□□□□□□□□■■■■■■■■■	703777740003771	26	CAS1-Delhi					1		Pakistan	P
FXX162008001508	2008	F	62	■■■■■□□□□□□□□□□□□■■□□□□□□□□□□□□□□□□□□□■■■■■	760001400000171	29	LAM	-243---3-2-4	Orphan	243324254723221	Orphan	0		unknown	NA
FXX162010102252	2010	M	50	■■■■■□□□□□□□□□□□□■■□□□□□□□□□□□□□□□□□□□■■■■■	760001400000171	29	LAM	-243---3-3-4	Orphan	243334242623221	Orphan	1		unknown	P
FXX162008301394	2008	M	54	■■■■■□□□□□□□□□□□□■■□□□□□□□□□□□□□□□□□□□■■■■■	760001400000171	29	LAM	-243---3-3-3	Orphan	243333244423221	Orphan	3	SM	unknown	P

FXX162007101112	2007	F	25	■■■■■■■□□□□□□■■■■■■■■■■■■■■■■■■■□□□□■■■□□□□	774037777760700	31	T	223222153323	9	232533---------	Orphan	1		Algeria	EP
FXX162001100029	2001	F	57	■■■■■■■□□□□□□■■■■■■■■■■■■■■■■■■■□□□□■■■□□□□	774037777760700	31	T					1		France	EP
FXX162003100307	2003	M	87	■■■■■■■□□□□□□■■■■■■■■■■■■■■■■■■■□□□□■■■□□□□	774037777760700	31	T					1		France	EP
FXX162000000392	2000	F	74	■■■■■■■□□□□□□■■■■■■■■■■■■■■■■■■■□□□□■■■□□□□	774037777760700	31	T					0		France	EP
FXX162007001252	2007	M	45	■■■■■■■□□□□□□■■■■■■■■■■■■■■■■■■■□□□□■■■□□□□	774037777760700	31	T	123325153323	897	233533---------	Orphan	0		unknown	NA
FXX162006000872	2006	F	67	■■■■■■■□□□□□□■■■■■■■■■■■■■■■■■■■□□□□■■■□□□□	774037777760700	31	T	223325123323	1557	233233343422226	Orphan	0		unknown	NA
FXX162006000840	2006	M	49	■■■■■■■□□□□□□■■■■■■■■■■■■■■■■■■■□□□□■■■□□□□	774037777760700	31	T	223325153325	219	233535---------	Orphan	0		unknown	NA
FXX162008001456	2008	F	84	■■■■■■■□□□□□□■■■■■■■■■■■■■■■■■■■□□□□■■■□□□□	774037777760700	31	T	-233---5-3-3	Orphan	233533343522223	322	0		unknown	NA
FXX162008001584	2008	M	62	■■■■■■■□□□□□□■■■■■■■■■■■■■■■■■■■□□□□■■■□□□□	774037777760700	31	T	-233---5-3-3	Orphan	233533343522223	322	0		unknown	NA
FXX162008001686	2008	F	61	■■■■■■■□□□□□□■■■■■■■■■■■■■■■■■■■□□□□■■■□□□□	774037777760700	31	T	-233---5-3-3	Orphan	233533343522223	322	0		unknown	NA
FXX162010002205	2010	F	78	■■■■■■■□□□□□□■■■■■■■■■■■■■■■■■■■□□□□■■■□□□□	774037777760700	31	T	-233---5-3-5	Orphan	233535344522223	Orphan	0		unknown	NA
FXX162005000668	2005	F	78	■■■■■■■□□□□□□■■■■■■■■■■■■■■■■■■■□□□□■■■□□□□	774037777760700	31	T					0		unknown	NA
FXX162001100094	2001	F	27	■■■■■■■□□□□□□■■■■■■■■■■■■■■■■■■■□□□□■■■□□□□	774037777760700	31	T					1		France	P
FXX162008101500	2008	M	61	■■■■■■■□□□□□□■■■■■■■■■■■■■■■■■■■□□□□■■■□□□□	774037777760700	31	T	-232---5-3-4	Orphan	232534362522223	Orphan	1		unknown	P
FXX162006000903	2006	F	31	■■■■■■■■□□□□□□□□□□□□□□□□□□□□□□□□□□□□□□■■■■■	776000000000171	32	Unknown					0		unknown	NA
FXX162008101173	2008	M	68	■■■■■■■■□□□■■■■■■■■■□□□□■■■■■■■■□□□□■■■■■■■	776177607760771	33	LAM3	224226153323	242	242533224724321	261	1		unknown	EP
FXX162005100659	2005	F	81	■■■■■■■■□□□■■■■■■■■■□□□□■■■■■■■■□□□□■■■■■■■	776177607760771	33	LAM3	224326153425	Orphan	243545---------	Orphan	1		Spain	EP
FXX162008001667	2008	F	18	■■■■■■■■□□□■■■■■■■■■□□□□■■■■■■■■□□□□■■■■■■■	776177607760771	33	LAM3	-242---5-3-4	Orphan	242534224624221	329	0		unknown	EP
FXX162008101596	2008	F	66	■■■■■■■■□□□■■■■■■■■■□□□□■■■■■■■■□□□□■■■■■■■	776177607760771	33	LAM3	-243---5-3-3	Orphan	243533243624321	339	1		unknown	EP
FXX162000000284	2000	F	76	■■■■■■■■□□□■■■■■■■■■□□□□■■■■■■■■□□□□■■■■■■■	776177607760771	33	LAM3					0		France	EP
FXX162007001157	2007	M	24	■■■■■■■■□□□■■■■■■■■■□□□□■■■■■■■■□□□□■■■■■■■	776177607760771	33	LAM3	224226153312	Orphan	242532---------	Orphan	0		unknown	NA
FXX162006000932	2006	F	86	■■■■■■■■□□□■■■■■■■■■□□□□■■■■■■■■□□□□■■■■■■■	776177607760771	33	LAM3	224226153323	242	242533---------	Orphan	0		unknown	NA
FXX162007001231	2007	M	79	■■■■■■■■□□□■■■■■■■■■□□□□■■■■■■■■□□□□■■■■■■■	776177607760771	33	LAM3	224326152323	226	243533---------	Orphan	0		unknown	NA
FXX162006000943	2006	M	79	■■■■■■■■□□□■■■■■■■■■□□□□■■■■■■■■□□□□■■■■■■■	776177607760771	33	LAM3	224326153323	213	243533---------	Orphan	0		unknown	NA
FXX162006000899	2006	M	56	■■■■■■■■□□□■■■■■■■■■□□□□■■■■■■■■□□□□■■■■■■■	776177607760771	33	LAM3	224336153323	Orphan	243533---------	Orphan	0		unknown	NA
FXX162007001092	2007	M	64	■■■■■■■■□□□■■■■■■■■■□□□□■■■■■■■■□□□□■■■■■■■	776177607760771	33	LAM3	225326153323	247	253533---------	Orphan	0		unknown	NA
FXX162008001412	2008	M	76	■■■■■■■■□□□■■■■■■■■■□□□□■■■■■■■■□□□□■■■■■■■	776177607760771	33	LAM3	-232---5-3-3	Orphan	232533243424321	Orphan	0		unknown	NA
FXX162010002297	2010	M	18	■■■■■■■■□□□■■■■■■■■■□□□□■■■■■■■■□□□□■■■■■■■	776177607760771	33	LAM3	-242---5-3-3	Orphan	242533224724321	261	0		unknown	NA
FXX162010002328	2010	M	36	■■■■■■■■□□□■■■■■■■■■□□□□■■■■■■■■□□□□■■■■■■■	776177607760771	33	LAM3	-242---5-3-3	Orphan	242533221423221	Orphan	0		unknown	NA
FXX162009002037	2009	F	34	■■■■■■■■□□□■■■■■■■■■□□□□■■■■■■■■□□□□■■■■■■■	776177607760771	33	LAM3	-242---5-3-4	Orphan	242534224624221	329	0		unknown	NA
FXX162010002340	2010	F	20	■■■■■■■■□□□■■■■■■■■■□□□□■■■■■■■■□□□□■■■■■■■	776177607760771	33	LAM3	-243---7-3-6	Orphan	243736242433312	Orphan	0		unknown	NA
FXX162006100683	2006	M	26	■■■■■■■■□□□■■■■■■■■■□□□□■■■■■■■■□□□□■■■■■■■	776177607760771	33	LAM3	224226153324	877	242534---------	Orphan	1		France	P
FXX162008101434	2008	F	30	■■■■■■■■□□□■■■■■■■■■□□□□■■■■■■■■□□□□■■■■■■■	776177607760771	33	LAM3	-242---5-3-3	Orphan	242533221-24321	Orphan	1		unknown	P
FXX162009301692	2009	M	24	■■■■■■■■□□□■■■■■■■■■□□□□■■■■■■■■□□□□■■■■■■■	776177607760771	33	LAM3	-242---5-3-4	Orphan	242534223326221	Orphan	3	SM, INH	unknown	P
FXX162009101817	2009	M	21	■■■■■■■■□□□■■■■■■■■■□□□□■■■■■■■■□□□□■■■■■■■	776177607760771	33	LAM3	-242---5-3-4	Orphan	242534223624221	328	1		France	P
FXX162010102282	2010	M	87	■■■■■■■■□□□■■■■■■■■■□□□□■■■■■■■■□□□□■■■■■■■	776177607760771	33	LAM3	-243---5-3-3	Orphan	243533224425421	Orphan	1		unknown	P
FXX162010102014	2010	F	81	■■■■■■■■□□□■■■■■■■■■□□□□■■■■■■■■□□□□■■■■■■■	776177607760771	33	LAM3	-243---5-3-5	Orphan	243535243623321	Orphan	1		France	P
FXX162003100058	2003	M	64	■■■■■■■■□□□■■■■■■■■■□□□□■■■■■■■■□□□□■■■■■■■	776177607760771	33	LAM3					1		France	P
FXX162002300189	2002	M	55	■■■■■■■■□□□■■■■■■■■■□□□□■■■■■■■■□□□□■■■■■■■	776177607760771	33	LAM3					3	SM	Spain	P
FXX162003000409	2003	M	71	■■■■■■■■□□□■■■■■■■■■□□□□■■■■■■■■□□□□■■■■■■■	776177607760771	33	LAM3					0		France	P
FXX162008001491	2008	M	23	■■■■■■■■□□□■■■■■■■■■□□□□■■■■■■■■□□□□■■■■■■■	776177607760771	33	LAM3	-242---5-3-4	Orphan	242534223624221	328	0		unknown	P
FXX162008101670	2008	F	2	■■■■■■■■□□□■■■■■■■■■□□□□■■■■■■■■□□□□■■■■■■■	776177607760771	33	LAM3	-242---5-3-4	Orphan	242534223624221	328	1		unknown	P
FXX162005100679	2005	M	25	■■■■■■■■□□■■■■■■■■■■■■■■■■■■■■■■□□□□■■■■■■■	776377777760771	34	S	231325153324	Orphan	313534---------	Orphan	1		Congo	EP
FXX162005000674	2005	F	73	■■■■■■■■□□■■■■■■■■■■■■■■■■■■■■■■□□□□■■■■■■■	776377777760771	34	S	233225134322	Orphan	332332---------	Orphan	0		unknown	NA
FXX162005000544	2005	M	42	■■■■■■■■□□■■■■■■■■■■■■■■■■■■■■■■□□□□■■■■■■■	776377777760771	34	S	233324143324	1587	333434---------	Orphan	0		unknown	NA
FXX162007001246	2007	F	46	■■■■■■■■□□■■■■■■■■■■■■■■■■■■■■■■□□□□■■■■■■■	776377777760771	34	S	233324143324	1587	333434---------	Orphan	0		unknown	NA
FXX162005000705	2005	M	20	■■■■■■■■□□■■■■■■■■■■■■■■■■■■■■■■□□□□■■■■■■■	776377777760771	34	S	333215153222	680	332522---------	Orphan	0		unknown	NA
FXX162006000934	2006	F	78	■■■■■■■■□□■■■■■■■■■■■■■■■■■■■■■■□□□□■■■■■■■	776377777760771	34	S	333215153222	680	332522---------	Orphan	0		unknown	NA
FXX162006000997	2006	M	35	■■■■■■■■□□■■■■■■■■■■■■■■■■■■■■■■□□□□■■■■■■■	776377777760771	34	S	-2--261533-1	Orphan	2--531---------	Orphan	0		unknown	NA
FXX162008001711	2008	M	31	■■■■■■■■□□■■■■■■■■■■■■■■■■■■■■■■□□□□■■■■■■■	776377777760771	34	S	-333---4-3-4	Orphan	333434344323123	Orphan	0		unknown	NA
FXX162008001647	2008	F	65	■■■■■■■■□□■■■■■■■■■■■■■■■■■■■■■■□□□□■■■■■■■	776377777760771	34	S	-333---5-2-2	Orphan	333522434223123	Orphan	0		unknown	NA
FXX162010002132	2010	F	81	■■■■■■■■□□■■■■■■■■■■■■■■■■■■■■■■□□□□■■■■■■■	776377777760771	34	S	-33----5-3-4	Orphan	33-534445923123	Orphan	0		unknown	NA
FXX162001000078	2001	M	24	■■■■■■■■□□■■■■■■■■■■■■■■■■■■■■■■□□□□■■■■■■■	776377777760771	34	S	233324143324	1587	333434---------	Orphan	0		Algeria	P
FXX162006101019	2006	M	38	■■■■■■■■□□■■■■■■■■■■■■■■■■■■■■■■□□□□■■■■■■■	776377777760771	34	S	233324143324	1587	333434---------	Orphan	1		Algeria	P
FXX162005100587	2005	M	59	■■■■■■■■□□■■■■■■■■■■■■■■■■■■■■■■□□□□■■■■■■■	776377777760771	34	S	233325153324	212	333534---------	Orphan	1		France	P
FXX162008101661	2008	M	41	■■■■■■■■□□■■■■■■■■■■■■■■■■■■■■■■□□□□■■■■■■■	776377777760771	34	S	-332---5-2-2	Orphan	332522445823123	Orphan	1		Morocco	P
FXX162001100054	2001	F	31	■■■■■■■■□□■■■■■■■■■■■■■■■■■■■■■■□□□□■■■■■■■	776377777760771	34	S					1		Algeria	P

FXX162002100212	2002	M	59	■■■■■■■■□□■■■■■■■■■■■■■■■■■■■■■■□□□□■■■■■■■	776377777760771	34	S					1		Cameroon	P
FXX162002100226	2002	M	74	■■■■■■■■□□■■■■■■■■■■■■■■■■■■■■■■□□□□■■■■■■■	776377777760771	34	S					1		Algeria	P
FXX162002100288	2002	F	46	■■■■■■■■□□■■■■■■■■■■■■■■■■■■■■■■□□□□■■■■■■■	776377777760771	34	S					1		France	P
FXX162000100341	2000	F	24	■■■■■■■■□□■■■■■■■■■■■■■■■■■■■■■■□□□□■■■■■■■	776377777760771	34	S					1		Albania	P
FXX162000000391	2000	M	69	■■■■■■■■□□■■■■■■■■■■■■■■■■■■■■■■□□□□■■■■■■■	776377777760771	34	S					0		Algeria	P
FXX162008101332	2008	M	80	■■■■■■■■□□■■■■■■■■■■■■■■■■■■■■■■□□□□■■■■■■■	776377777760771	34	S	-333---5-2-4	Orphan	333524342823124	Orphan	1		Italy	P
FXX162008101628	2008	M	78	■■■■■■■■□□■■■■■■■■■■■■■■■■■■■■■■□□□□■■■■■■■	776377777760771	34	S	-333---5-3-4	Orphan	333534343723123	Orphan	1		unknown	P
FXX162008301548	2008	M	24	■■■■■■■■□□■■■■■■■■■■■■■■■■■■■■■■□□□□■■■■■■■	776377777760771	34	S	-333---3-2-2	Orphan	333322444523123	Orphan	3	SM	unknown	P
FXX162010002140	2010	M	76	■■■■■■■■■■■■□■■■■■■■■■■■■■■■■□□□■□□□□■■■■■■■	777737777420771	35	Ural-1	-292---1-3-2	Orphan	292132442833333	Orphan	0		unknown	NA
FXX162008001406	2008	F	26	■■■■■■■■■■■■□■■■■■■■■■■■■■■■■□□□■□□□□■■■■■■■	777737777420771	35	Ural-1	-2B2---1-3-2	Orphan	2B2132442834343	381	0		unknown	NA
FXX162008001676	2008	F	26	■■■■■■■■■■■■□■■■■■■■■■■■■■■■■□□□■□□□□■■■■■■■	777737777420771	35	Ural-1	-2B2---1-3-2	Orphan	2B2132442834343	381	0		unknown	NA
FXX162000000148	2000	M	77	■■■■■■■■■■■■□■■■■■■■■■■■■■■■■□□□■□□□□■■■■■■■	777737777420771	35	Ural-1					0		France	P
FXX162001100093	2001	F	53	■■■■■■■■■■■■□■■■■■■■■■■■■■■■■■■□■□□□□■■■■■■■	777737777720771	36	H3					1		Algeria	EP
FXX162006000843	2006	M	73	■■■■■■■■■■■■□■■■■■■■■■■■■■■■■■■□■□□□□■■■■■■■	777737777720771	36	H3	225313153323	42	253533---------	Orphan	0		unknown	NA
FXX162006000871	2006	M	48	■■■■■■■■■■■■□■■■■■■■■■■■■■■■■■■□■□□□□■■■■■■■	777737777720771	36	H3	225313153323	42	253533---------	Orphan	0		unknown	NA
FXX162007001147	2007	F	60	■■■■■■■■■■■■□■■■■■■■■■■■■■■■■■■□■□□□□■■■■■■■	777737777720771	36	H3	225313153324	1093	253534---------	Orphan	0		unknown	NA
FXX162006000852	2006	M	55	■■■■■■■■■■■■□■■■■■■■■■■■■■■■■■■□■□□□□■■■■■■■	777737777720771	36	H3	225323153323	43	253533---------	Orphan	0		unknown	NA
FXX162007001239	2007	M	33	■■■■■■■■■■■■□■■■■■■■■■■■■■■■■■■□■□□□□■■■■■■■	777737777720771	36	H3	225323153323	43	253533---------	Orphan	0		unknown	NA
FXX162010002343	2010	M	31	■■■■■■■■■■■■□■■■■■■■■■■■■■■■■■■□■□□□□■■■■■■■	777737777720771	36	H3	-232---5-1-3	Orphan	232513245422321	Orphan	0		unknown	NA
FXX162009002032	2009	F	78	■■■■■■■■■■■■□■■■■■■■■■■■■■■■■■■□■□□□□■■■■■■■	777737777720771	36	H3	-253---5-3-3	Orphan	253533336822243	Orphan	0		unknown	NA
FXX162010002286	2010	M	49	■■■■■■■■■■■■□■■■■■■■■■■■■■■■■■■□■□□□□■■■■■■■	777737777720771	36	H3	-253---5-3-3	Orphan	253533335722243	150	0		unknown	NA
FXX162010002296	2010	F	64	■■■■■■■■■■■■□■■■■■■■■■■■■■■■■■■□■□□□□■■■■■■■	777737777720771	36	H3	-253---5-3-3	Orphan	253533335822243	373	0		unknown	NA
FXX162008001698	2008	M	81	■■■■■■■■■■■■□■■■■■■■■■■■■■■■■■■□■□□□□■■■■■■■	777737777720771	36	H3	-262---5-3-1	Orphan	262531235632343	Orphan	0		unknown	NA
FXX162005300562	2005	M	55	■■■■■■■■■■■■□■■■■■■■■■■■■■■■■■■□■□□□□■■■■■■■	777737777720771	36	H3	125313153323	1547	253533---------	Orphan	3	SM	unknown	P
FXX162009301940	2009	M	41	■■■■■■■■■■■■□■■■■■■■■■■■■■■■■■■□■□□□□■■■■■■■	777737777720771	36	H3	-253---5-3-3	Orphan	253533335722243	150	3	SM	unknown	P
FXX162000000280	2000	M	64	■■■■■■■■■■■■□■■■■■■■■■■■■■■■■■■□■□□□□■■■■■■■	777737777720771	36	H3					0		Tunisia	P
FXX162008301485	2008	M	80	■■■■■■■■■■■■□■■■■■■■■■■■■■■■■■■■■□□□□■■■■■■■	777737777760771	37	T3	-243---5-4-4	Orphan	243544133532343	Orphan	3	SM	unknown	EP
FXX162010102255	2010	M	69	■■■■■■■■■■■■□■■■■■■■■■■■■■■■■■■■■□□□□■■■■■■■	777737777760771	37	T3	-253---5-3-3	Orphan	253533335822243	373	1		unknown	EP
FXX162006000801	2006	M	47	■■■■■■■■■■■■□■■■■■■■■■■■■■■■■■■■■□□□□■■■■■■■	777737777760771	37	T3	223225153323	134	232533---------	Orphan	0		unknown	NA
FXX162006000809	2006	F	51	■■■■■■■■■■■■□■■■■■■■■■■■■■■■■■■■■□□□□■■■■■■■	777737777760771	37	T3	223325153222	335	233522---------	Orphan	0		unknown	NA
FXX162005000644	2005	F	84	■■■■■■■■■■■■□■■■■■■■■■■■■■■■■■■■■□□□□■■■■■■■	777737777760771	37	T3	224125153321	Orphan	241531---------	Orphan	0		unknown	NA
FXX162007001186	2007	F	86	■■■■■■■■■■■■□■■■■■■■■■■■■■■■■■■■■□□□□■■■■■■■	777737777760771	37	T3	228325163421	1584	283641---------	Orphan	0		unknown	NA
FXX162008001451	2008	M	82	■■■■■■■■■■■■□■■■■■■■■■■■■■■■■■■■■□□□□■■■■■■■	777737777760771	37	T3	-232---5-3-6	Orphan	232536342623223	Orphan	0		unknown	NA
FXX162010002268	2010	F	84	■■■■■■■■■■■■□■■■■■■■■■■■■■■■■■■■■□□□□■■■■■■■	777737777760771	37	T3	-233---5-3-2	Orphan	233532345522223	321	0		unknown	NA
FXX162006100948	2006	F	23	■■■■■■■■■■■■□■■■■■■■■■■■■■■■■■■■■□□□□■■■■■■■	777737777760771	37	T3	223325133322	938	233332---------	Orphan	1		Algeria	P
FXX162010102243	2010	F	24	■■■■■■■■■■■■□■■■■■■■■■■■■■■■■■■■■□□□□■■■■■■■	777737777760771	37	T3	-231---5-3-5	Orphan	231535244522225	Orphan	1		unknown	P
FXX162009101939	2009	M	62	■■■■■■■■■■■■□■■■■■■■■■■■■■■■■■■■■□□□□■■■■■■■	777737777760771	37	T3	-251---1-3-3	Orphan	251133342131243	Orphan	1		unknown	P
FXX162002100173	2002	F	82	■■■■■■■■■■■■□■■■■■■■■■■■■■■■■■■■■□□□□■■■■■■■	777737777760771	37	T3					1		France	P
FXX162003100297	2003	M	65	■■■■■■■■■■■■□■■■■■■■■■■■■■■■■■■■■□□□□■■■■■■■	777737777760771	37	T3					1		Morocco	P
FXX162004100462	2004	F	8	■■■■■■■■■■■■□■■■■■■■■■■■■■■■■■■■■□□□□■■■■■■■	777737777760771	37	T3					1		Ethiopia	P
FXX162001100049	2001	F	69	■■■■■■■■■■■■■■■■■■□■■■□□■■■■■■■■□□□□■□□■■■■	777777347760471	39	T4-CEU1					1		France	EP
FXX162006000851	2006	F	94	■■■■■■■■■■■■■■■■■■□■■■□□■■■■■■■■□□□□■□□■■■■	777777347760471	39	T4-CEU1	222323143324	Orphan	223434---------	Orphan	0		unknown	NA
FXX162008001410	2008	M	85	■■■■■■■■■■■■■■■■■■□■■■□□■■■■■■■■□□□□■□□■■■■	777777347760471	39	T4-CEU1	-243---4-3-4	Orphan	243434337532443	Orphan	0		unknown	NA
FXX162010002226	2010	M	74	■■■■■■■■■■■■■■■■■■□■■■□□■■■■■■■■□□□□■□□■■■■	777777347760471	39	T4-CEU1	-253---4-3-4	Orphan	253434332532444	Orphan	0		unknown	NA
FXX162003100325	2003	M	67	■■■■■■■■■■■■■■■■■■□■■■□□■■■■■■■■□□□□■□□■■■■	777777347760471	39	T4-CEU1					1		Yugoslavia	P
FXX162006001050	2006	F	34	■■■■■■■■■■■■■■■■■■□■■■■■■■■■■■■■■□□□□■■■■■■■	777777377760771	40	T4	223315143324	551	233434---------	Orphan	0		unknown	NA
FXX162007001084	2007	M	37	■■■■■■■■■■■■■■■■■■□■■■■■■■■■■■■■■□□□□■■■■■■■	777777377760771	40	T4	223425153323	382	234533---------	Orphan	0		Romania	NA
FXX162007101295	2007	M	22	■■■■■■■■■■■■■■■■■■□■■■■■■■■■■■■■■□□□□■■■■■■■	777777377760771	40	T4	214325153423	Orphan	143543---------	Orphan	1		Armenia	P
FXX162009001878	2009	F	21	■■■■■■■■■■■■■■■■■■■□□□□□■□□■■■■■□□□□■■■■■■■	777777404760771	41	LAM7-TUR	-151---1-3-2	Orphan	151132342734343	298	0		unknown	NA
FXX162010002208	2010	F	64	■■■■■■■■■■■■■■■■■■■□□□□□■□□■■■■■□□□□■■■■■■■	777777404760771	41	LAM7-TUR	-151---1-3-2	Orphan	151132342714343	Orphan	0		unknown	NA
FXX162006100776	2006	M	21	■■■■■■■■■■■■■■■■■■■□□□□□■□□■■■■■□□□□■■■■■■■	777777404760771	41	LAM7-TUR	214125113322	194	141132---------	Orphan	1		unknown	P
FXX162006100777	2006	F	27	■■■■■■■■■■■■■■■■■■■□□□□□■□□■■■■■□□□□■■■■■■■	777777404760771	41	LAM7-TUR	214125113322	194	141132---------	Orphan	1		unknown	P
FXX162006100971	2006	F	22	■■■■■■■■■■■■■■■■■■■□□□□□■□□■■■■■□□□□■■■■■■■	777777404760771	41	LAM7-TUR	214125113322	194	141132---------	Orphan	1		unknown	P
FXX162007101123	2007	F	19	■■■■■■■■■■■■■■■■■■■□□□□□■□□■■■■■□□□□■■■■■■■	777777404760771	41	LAM7-TUR	215125113322	310	151132---------	Orphan	1		Congo	P
FXX162006100988	2006	F	74	■■■■■■■■■■■■■■■■■■■■□□□□■■■■■■■■□□□□■■■■■■■	777777607760771	42	LAM9	124326153326	816	243536---------	Orphan	1		France	EP
FXX162005100568	2005	F	70	■■■■■■■■■■■■■■■■■■■■□□□□■■■■■■■■□□□□■■■■■■■	777777607760771	42	LAM9	224325132425	Orphan	243345---------	Orphan	1		Algeria	EP
FXX162007101176	2007	F	29	■■■■■■■■■■■■■■■■■■■■□□□□■■■■■■■■□□□□■■■■■■■	777777607760771	42	LAM9	224325182424	345	243844---------	Orphan	1		Morocco	EP

FXX162008101400	2008	F	76	■■■■■■■■■■■■■■■■■■■■□□□□■■■■■■■■□□□□■■■■■■■	777777607760771	42	LAM9	-233---5-3-7	Orphan	233537242633312	Orphan	1		unknown	EP
FXX162008101494	2008	F	27	■■■■■■■■■■■■■■■■■■■■□□□□■■■■■■■■□□□□■■■■■■■	777777607760771	42	LAM9	-233---8-3-7	Orphan	233837242434212	Orphan	1		unknown	EP
FXX162008101551	2008	F	41	■■■■■■■■■■■■■■■■■■■■□□□□■■■■■■■■□□□□■■■■■■■	777777607760771	42	LAM9	-242---5-3-1	Orphan	242531242324312	Orphan	1		unknown	EP
FXX162008101430	2008	M	37	■■■■■■■■■■■■■■■■■■■■□□□□■■■■■■■■□□□□■■■■■■■	777777607760771	42	LAM9	-243---8-4-4	Orphan	243844243524226	345	1		Morocco	EP
FXX162008101631	2008	F	48	■■■■■■■■■■■■■■■■■■■■□□□□■■■■■■■■□□□□■■■■■■■	777777607760771	42	LAM9	-243---8-4-5	Orphan	243845243525228	262	1		unknown	EP
FXX162010202098	2010	M	62	■■■■■■■■■■■■■■■■■■■■□□□□■■■■■■■■□□□□■■■■■■■	777777607760771	42	LAM9	-243---8-5-4	Orphan	243854242524224	Orphan	2	RIF, INH, SM	unknown	EP
FXX162001100097	2001	M	67	■■■■■■■■■■■■■■■■■■■■□□□□■■■■■■■■□□□□■■■■■■■	777777607760771	42	LAM9					1		Morocco	EP
FXX162003100118	2003	M	32	■■■■■■■■■■■■■■■■■■■■□□□□■■■■■■■■□□□□■■■■■■■	777777607760771	42	LAM9					1		Morocco	EP
FXX162000100159	2000	F	61	■■■■■■■■■■■■■■■■■■■■□□□□■■■■■■■■□□□□■■■■■■■	777777607760771	42	LAM9					1		France	EP
FXX162002100171	2002	M	55	■■■■■■■■■■■■■■■■■■■■□□□□■■■■■■■■□□□□■■■■■■■	777777607760771	42	LAM9					1		Tunisia	EP
FXX162002100179	2002	M	77	■■■■■■■■■■■■■■■■■■■■□□□□■■■■■■■■□□□□■■■■■■■	777777607760771	42	LAM9					1		Algeria	EP
FXX162003200270	2003	F	48	■■■■■■■■■■■■■■■■■■■■□□□□■■■■■■■■□□□□■■■■■■■	777777607760771	42	LAM9					2	INH, RIF, SM	Italy	EP
FXX162004100469	2004	M	7	■■■■■■■■■■■■■■■■■■■■□□□□■■■■■■■■□□□□■■■■■■■	777777607760771	42	LAM9					1		Morocco	EP
FXX162004100483	2004	F	55	■■■■■■■■■■■■■■■■■■■■□□□□■■■■■■■■□□□□■■■■■■■	777777607760771	42	LAM9					1		Vietnam	EP
FXX162006000963	2006	F	75	■■■■■■■■■■■■■■■■■■■■□□□□■■■■■■■■□□□□■■■■■■■	777777607760771	42	LAM9	124326153322	991	243532---------	Orphan	0		unknown	NA
FXX162007001243	2007	M	69	■■■■■■■■■■■■■■■■■■■■□□□□■■■■■■■■□□□□■■■■■■■	777777607760771	42	LAM9	124326153322	991	243532---------	Orphan	0		unknown	NA
FXX162007001260	2007	F	39	■■■■■■■■■■■■■■■■■■■■□□□□■■■■■■■■□□□□■■■■■■■	777777607760771	42	LAM9	124326153323	249	243533---------	Orphan	0		unknown	NA
FXX162006000905	2006	M	94	■■■■■■■■■■■■■■■■■■■■□□□□■■■■■■■■□□□□■■■■■■■	777777607760771	42	LAM9	124326163224	565	243624---------	Orphan	0		unknown	NA
FXX162006001054	2006	F	80	■■■■■■■■■■■■■■■■■■■■□□□□■■■■■■■■□□□□■■■■■■■	777777607760771	42	LAM9	221325133426	Orphan	213346---------	Orphan	0		unknown	NA
FXX162007001261	2007	F	56	■■■■■■■■■■■■■■■■■■■■□□□□■■■■■■■■□□□□■■■■■■■	777777607760771	42	LAM9	223126142321	1262	231431---------	Orphan	0		unknown	NA
FXX162006000842	2006	M	48	■■■■■■■■■■■■■■■■■■■■□□□□■■■■■■■■□□□□■■■■■■■	777777607760771	42	LAM9	223226133321	298	232331---------	Orphan	0		unknown	NA
FXX162006001007	2006	M	83	■■■■■■■■■■■■■■■■■■■■□□□□■■■■■■■■□□□□■■■■■■■	777777607760771	42	LAM9	223226153321	128	232531---------	Orphan	0		unknown	NA
FXX162005000718	2005	M	24	■■■■■■■■■■■■■■■■■■■■□□□□■■■■■■■■□□□□■■■■■■■	777777607760771	42	LAM9	223326163424	1500	233644---------	Orphan	0		unknown	NA
FXX162007001273	2007	M	23	■■■■■■■■■■■■■■■■■■■■□□□□■■■■■■■■□□□□■■■■■■■	777777607760771	42	LAM9	224325182425	1570	243845243525228	262	0		unknown	NA
FXX162007001102	2007	M	45	■■■■■■■■■■■■■■■■■■■■□□□□■■■■■■■■□□□□■■■■■■■	777777607760771	42	LAM9	224226143321	738	242431---------	Orphan	0		unknown	NA
FXX162007001253	2007	F	82	■■■■■■■■■■■■■■■■■■■■□□□□■■■■■■■■□□□□■■■■■■■	777777607760771	42	LAM9	224226143321	738	242431---------	Orphan	0		unknown	NA
FXX162007001269	2007	F	56	■■■■■■■■■■■■■■■■■■■■□□□□■■■■■■■■□□□□■■■■■■■	777777607760771	42	LAM9	224316153226	Orphan	243526---------	Orphan	0		unknown	NA
FXX162006000811	2006	F	47	■■■■■■■■■■■■■■■■■■■■□□□□■■■■■■■■□□□□■■■■■■■	777777607760771	42	LAM9	224324183422	1503	243842---------	Orphan	0		unknown	NA
FXX162006000771	2006	F	38	■■■■■■■■■■■■■■■■■■■■□□□□■■■■■■■■□□□□■■■■■■■	777777607760771	42	LAM9	224325133422	Orphan	243342---------	Orphan	0		unknown	NA
FXX162006000962	2006	F	51	■■■■■■■■■■■■■■■■■■■■□□□□■■■■■■■■□□□□■■■■■■■	777777607760771	42	LAM9	224325163323	343	243633---------	Orphan	0		unknown	NA
FXX162006000796	2006	M	60	■■■■■■■■■■■■■■■■■■■■□□□□■■■■■■■■□□□□■■■■■■■	777777607760771	42	LAM9	224325172424	965	243744---------	Orphan	0		unknown	NA
FXX162006000860	2006	M	58	■■■■■■■■■■■■■■■■■■■■□□□□■■■■■■■■□□□□■■■■■■■	777777607760771	42	LAM9	224326153325	843	243535---------	Orphan	0		unknown	NA
FXX162006000888	2006	M	31	■■■■■■■■■■■■■■■■■■■■□□□□■■■■■■■■□□□□■■■■■■■	777777607760771	42	LAM9	224326173424	347	243744---------	Orphan	0		unknown	NA
FXX162010002171	2010	M	21	■■■■■■■■■■■■■■■■■■■■□□□□■■■■■■■■□□□□■■■■■■■	777777607760771	42	LAM9	-213---6-2-6	Orphan	213626242734312	299	0		unknown	NA
FXX162008001578	2008	M	67	■■■■■■■■■■■■■■■■■■■■□□□□■■■■■■■■□□□□■■■■■■■	777777607760771	42	LAM9	-223---5-4-7	Orphan	223547242733312	Orphan	0		unknown	NA
FXX162010002225	2010	M	53	■■■■■■■■■■■■■■■■■■■■□□□□■■■■■■■■□□□□■■■■■■■	777777607760771	42	LAM9	-232---4-3-1	Orphan	232431243824312	Orphan	0		unknown	NA
FXX162007001308	2007	M	18	■■■■■■■■■■■■■■■■■■■■□□□□■■■■■■■■□□□□■■■■■■■	777777607760771	42	LAM9	-233---5-3-6	Orphan	233536242533312	Orphan	0		unknown	NA
FXX162009001876	2009	F	43	■■■■■■■■■■■■■■■■■■■■□□□□■■■■■■■■□□□□■■■■■■■	777777607760771	42	LAM9	-233---8-3-3	Orphan	233833242833212	Orphan	0		unknown	NA
FXX162008001638	2008	F	22	■■■■■■■■■■■■■■■■■■■■□□□□■■■■■■■■□□□□■■■■■■■	777777607760771	42	LAM9	-234---7-3-3	Orphan	234733245522224	Orphan	0		unknown	NA
FXX162009001782	2009	M	60	■■■■■■■■■■■■■■■■■■■■□□□□■■■■■■■■□□□□■■■■■■■	777777607760771	42	LAM9	-242---7-4-6	Orphan	242746223522225	Orphan	0		unknown	NA
FXX162008001445	2008	M	25	■■■■■■■■■■■■■■■■■■■■□□□□■■■■■■■■□□□□■■■■■■■	777777607760771	42	LAM9	-242---4-3-1	Orphan	242431244425312	Orphan	0		unknown	NA
FXX162008001525	2008	F	80	■■■■■■■■■■■■■■■■■■■■□□□□■■■■■■■■□□□□■■■■■■■	777777607760771	42	LAM9	-242---5-3-1	Orphan	242531246625312	Orphan	0		unknown	NA
FXX162007001346	2007	F	29	■■■■■■■■■■■■■■■■■■■■□□□□■■■■■■■■□□□□■■■■■■■	777777607760771	42	LAM9	-242---5-3-3	Orphan	242533242733312	Orphan	0		unknown	NA
FXX162009001868	2009	M	35	■■■■■■■■■■■■■■■■■■■■□□□□■■■■■■■■□□□□■■■■■■■	777777607760771	42	LAM9	-242---5-3-5	Orphan	242535242733112	332	0		unknown	NA
FXX162008001648	2008	M	84	■■■■■■■■■■■■■■■■■■■■□□□□■■■■■■■■□□□□■■■■■■■	777777607760771	42	LAM9	-242---8-4-4	Orphan	242844243524226	Orphan	0		unknown	NA
FXX162010002175	2010	M	49	■■■■■■■■■■■■■■■■■■■■□□□□■■■■■■■■□□□□■■■■■■■	777777607760771	42	LAM9	-243---5-2-4	Orphan	243524222423312	Orphan	0		unknown	NA
FXX162008001728	2008	M	42	■■■■■■■■■■■■■■■■■■■■□□□□■■■■■■■■□□□□■■■■■■■	777777607760771	42	LAM9	-243---7-4-6	Orphan	243746223522225	333	0		unknown	NA
FXX162010002149	2010	F	56	■■■■■■■■■■■■■■■■■■■■□□□□■■■■■■■■□□□□■■■■■■■	777777607760771	42	LAM9	-243---4-2-4	Orphan	243424243-24223	Orphan	0		unknown	NA
FXX162010002053	2010	M	26	■■■■■■■■■■■■■■■■■■■■□□□□■■■■■■■■□□□□■■■■■■■	777777607760771	42	LAM9	-243---5-2-2	Orphan	243522242833312	Orphan	0		unknown	NA
FXX162009001847	2009	F	59	■■■■■■■■■■■■■■■■■■■■□□□□■■■■■■■■□□□□■■■■■■■	777777607760771	42	LAM9	-243---5-3-2	Orphan	243532242733312	338	0		unknown	NA
FXX162007001344	2007	F	24	■■■■■■■■■■■■■■■■■■■■□□□□■■■■■■■■□□□□■■■■■■■	777777607760771	42	LAM9	-243---5-3-3	Orphan	243533242733312	340	0		unknown	NA
FXX162008001405	2008	M	34	■■■■■■■■■■■■■■■■■■■■□□□□■■■■■■■■□□□□■■■■■■■	777777607760771	42	LAM9	-243---5-3-3	Orphan	243533242733312	340	0		unknown	NA
FXX162010002131	2010	F	48	■■■■■■■■■■■■■■■■■■■■□□□□■■■■■■■■□□□□■■■■■■■	777777607760771	42	LAM9	-243---5-3-3	Orphan	243533242733312	340	0		unknown	NA
FXX162009001777	2009	M	60	■■■■■■■■■■■■■■■■■■■■□□□□■■■■■■■■□□□□■■■■■■■	777777607760771	42	LAM9	-243---5-3-5	Orphan	243535242634312	Orphan	0		unknown	NA
FXX162008001535	2008	M	78	■■■■■■■■■■■■■■■■■■■■□□□□■■■■■■■■□□□□■■■■■■■	777777607760771	42	LAM9	-243---5-3-6	Orphan	243536242634312	Orphan	0		unknown	NA
FXX162007001350	2007	M	55	■■■■■■■■■■■■■■■■■■■■□□□□■■■■■■■■□□□□■■■■■■■	777777607760771	42	LAM9	-243---6-3-3	Orphan	243633243724222	342	0		unknown	NA
FXX162009001781	2009	F	24	■■■■■■■■■■■■■■■■■■■■□□□□■■■■■■■■□□□□■■■■■■■	777777607760771	42	LAM9	-243---6-3-3	Orphan	243633243724222	342	0		unknown	NA

FXX162010002219	2010	M	84	■■■■■■■■■■■■■■■■■■■■□□□□■■■■■■■■□□□□■■■■■■■	777777607760771	42	LAM9	-243---6-3-4	Orphan	243634243525226	Orphan	0		unknown	NA
FXX162007001310	2007	F	33	■■■■■■■■■■■■■■■■■■■■□□□□■■■■■■■■□□□□■■■■■■■	777777607760771	42	LAM9	-243---6-3-7	Orphan	243637242633312	Orphan	0		unknown	NA
FXX162010002080	2010	M	20	■■■■■■■■■■■■■■■■■■■■□□□□■■■■■■■■□□□□■■■■■■■	777777607760771	42	LAM9	-243---6-4-4	Orphan	243644243524225	Orphan	0		unknown	NA
FXX162010002141	2010	M	77	■■■■■■■■■■■■■■■■■■■■□□□□■■■■■■■■□□□□■■■■■■■	777777607760771	42	LAM9	-243---7-2-5	Orphan	243725243526226	Orphan	0		unknown	NA
FXX162009001973	2009	M	64	■■■■■■■■■■■■■■■■■■■■□□□□■■■■■■■■□□□□■■■■■■■	777777607760771	42	LAM9	-243---7-4-4	Orphan	243744243524226	Orphan	0		unknown	NA
FXX162007001355	2007	M	40	■■■■■■■■■■■■■■■■■■■■□□□□■■■■■■■■□□□□■■■■■■■	777777607760771	42	LAM9	-243---8-4-4	Orphan	243844243522226	346	0		unknown	NA
FXX162008001519	2008	F	58	■■■■■■■■■■■■■■■■■■■■□□□□■■■■■■■■□□□□■■■■■■■	777777607760771	42	LAM9	-243---8-4-4	Orphan	243844243524226	345	0		unknown	NA
FXX162008001523	2008	F	64	■■■■■■■■■■■■■■■■■■■■□□□□■■■■■■■■□□□□■■■■■■■	777777607760771	42	LAM9	-243---8-4-4	Orphan	243844243524224	Orphan	0		unknown	NA
FXX162008001678	2008	M	23	■■■■■■■■■■■■■■■■■■■■□□□□■■■■■■■■□□□□■■■■■■■	777777607760771	42	LAM9	-243---8-4-4	Orphan	243844243522226	346	0		unknown	NA
FXX162009001983	2009	M	26	■■■■■■■■■■■■■■■■■■■■□□□□■■■■■■■■□□□□■■■■■■■	777777607760771	42	LAM9	-243---8-4-4	Orphan	24384424352-226	Orphan	0		unknown	NA
FXX162009002033	2009	M	80	■■■■■■■■■■■■■■■■■■■■□□□□■■■■■■■■□□□□■■■■■■■	777777607760771	42	LAM9	-243---8-4-4	Orphan	243844242524226	Orphan	0		unknown	NA
FXX162010002230	2010	F	15	■■■■■■■■■■■■■■■■■■■■□□□□■■■■■■■■□□□□■■■■■■■	777777607760771	42	LAM9	-243---8-4-4	Orphan	243844243522226	346	0		unknown	NA
FXX162010002276	2010	M	55	■■■■■■■■■■■■■■■■■■■■□□□□■■■■■■■■□□□□■■■■■■■	777777607760771	42	LAM9	-243---8-4-4	Orphan	243844243524227	Orphan	0		unknown	NA
FXX162008001609	2008	M	80	■■■■■■■■■■■■■■■■■■■■□□□□■■■■■■■■□□□□■■■■■■■	777777607760771	42	LAM9	-243---8-4-5	Orphan	243845243524226	Orphan	0		unknown	NA
FXX162008001402	2008	M	68	■■■■■■■■■■■■■■■■■■■■□□□□■■■■■■■■□□□□■■■■■■■	777777607760771	42	LAM9	-243---6-3-1	Orphan	243631253524222	Orphan	0		unknown	NA
FXX162009001873	2009	F	51	■■■■■■■■■■■■■■■■■■■■□□□□■■■■■■■■□□□□■■■■■■■	777777607760771	42	LAM9	-253---3-3-6	Orphan	253336242B33312	Orphan	0		unknown	NA
FXX162006300820	2006	M	31	■■■■■■■■■■■■■■■■■■■■□□□□■■■■■■■■□□□□■■■■■■■	777777607760771	42	LAM9	121126163326	Orphan	211636---------	Orphan	3	SM, INH	Morocco	P
FXX162006101017	2006	F	56	■■■■■■■■■■■■■■■■■■■■□□□□■■■■■■■■□□□□■■■■■■■	777777607760771	42	LAM9	121326153324	594	213534---------	Orphan	1		Vietnam	P
FXX162006101015	2006	M	31	■■■■■■■■■■■■■■■■■■■■□□□□■■■■■■■■□□□□■■■■■■■	777777607760771	42	LAM9	123326153324	323	233534243933512	Orphan	1		France	P
FXX162006200685	2006	M	56	■■■■■■■■■■■■■■■■■■■■□□□□■■■■■■■■□□□□■■■■■■■	777777607760771	42	LAM9	124325153225	1	243525---------	Orphan	2	SM, RIF, INH, ETH, PZA	Ukraine	P
FXX162005100588	2005	M	79	■■■■■■■■■■■■■■■■■■■■□□□□■■■■■■■■□□□□■■■■■■■	777777607760771	42	LAM9	124326153226	151	243526---------	Orphan	1		France	P
FXX162007101074	2007	F	8	■■■■■■■■■■■■■■■■■■■■□□□□■■■■■■■■□□□□■■■■■■■	777777607760771	42	LAM9	124326153323	249	243533---------	Orphan	1		Cameroon	P
FXX162006100983	2006	M	20	■■■■■■■■■■■■■■■■■■■■□□□□■■■■■■■■□□□□■■■■■■■	777777607760771	42	LAM9	223126152321	237	231531---------	Orphan	1		Congo	P
FXX162006100916	2006	M	19	■■■■■■■■■■■■■■■■■■■■□□□□■■■■■■■■□□□□■■■■■■■	777777607760771	42	LAM9	223226141323	Orphan	232433---------	Orphan	1		Algeria	P
FXX162005100579	2005	F	20	■■■■■■■■■■■■■■■■■■■■□□□□■■■■■■■■□□□□■■■■■■■	777777607760771	42	LAM9	223236141321	Orphan	232431---------	Orphan	1		France	P
FXX162005100613	2005	M	76	■■■■■■■■■■■■■■■■■■■■□□□□■■■■■■■■□□□□■■■■■■■	777777607760771	42	LAM9	223325180424	Orphan	233844---------	Orphan	1		unknown	P
FXX162005100315	2005	M	45	■■■■■■■■■■■■■■■■■■■■□□□□■■■■■■■■□□□□■■■■■■■	777777607760771	42	LAM9	224315183424	Orphan	243844---------	Orphan	1		France	P
FXX162003100298	2003	M	54	■■■■■■■■■■■■■■■■■■■■□□□□■■■■■■■■□□□□■■■■■■■	777777607760771	42	LAM9	224325182424	345	243844---------	Orphan	1		Morocco	P
FXX162005100565	2005	F	22	■■■■■■■■■■■■■■■■■■■■□□□□■■■■■■■■□□□□■■■■■■■	777777607760771	42	LAM9	224325182424	345	243844---------	Orphan	1		Central African Republic	P
FXX162006300989	2006	M	84	■■■■■■■■■■■■■■■■■■■■□□□□■■■■■■■■□□□□■■■■■■■	777777607760771	42	LAM9	224325182424	345	243844---------	Orphan	3	INH	Algeria	P
FXX162005100553	2005	M	53	■■■■■■■■■■■■■■■■■■■■□□□□■■■■■■■■□□□□■■■■■■■	777777607760771	42	LAM9	224325183424	1578	243844---------	Orphan	1		France	P
FXX162009101796	2009	M	30	■■■■■■■■■■■■■■■■■■■■□□□□■■■■■■■■□□□□■■■■■■■	777777607760771	42	LAM9	-213---6-2-6	Orphan	213626242734312	299	1		Algeria	P
FXX162010102095	2010	F	27	■■■■■■■■■■■■■■■■■■■■□□□□■■■■■■■■□□□□■■■■■■■	777777607760771	42	LAM9	-231---6-3-1	Orphan	231631144-25312	Orphan	1		Angola	P
FXX162009101756	2009	M	87	■■■■■■■■■■■■■■■■■■■■□□□□■■■■■■■■□□□□■■■■■■■	777777607760771	42	LAM9	-232---4-3-1	Orphan	232431244824312	Orphan	1		unknown	P
FXX162009101910	2009	F	28	■■■■■■■■■■■■■■■■■■■■□□□□■■■■■■■■□□□□■■■■■■■	777777607760771	42	LAM9	-242---5-3-1	Orphan	242531232733112	Orphan	1		unknown	P
FXX162008101420	2008	M	56	■■■■■■■■■■■■■■■■■■■■□□□□■■■■■■■■□□□□■■■■■■■	777777607760771	42	LAM9	-242---4-3-1	Orphan	242431243-24312	Orphan	1		unknown	P
FXX162010102094	2010	M	30	■■■■■■■■■■■■■■■■■■■■□□□□■■■■■■■■□□□□■■■■■■■	777777607760771	42	LAM9	-242---5-3-5	Orphan	242535242733112	332	1		unknown	P
FXX162009101747	2009	M	2	■■■■■■■■■■■■■■■■■■■■□□□□■■■■■■■■□□□□■■■■■■■	777777607760771	42	LAM9	-243---7-2-6	Orphan	243726223522225	Orphan	1		Algeria	P
FXX162009101723	2009	F	1	■■■■■■■■■■■■■■■■■■■■□□□□■■■■■■■■□□□□■■■■■■■	777777607760771	42	LAM9	-243---7-4-6	Orphan	243746223522225	333	1		unknown	P
FXX162009301821	2009	M	40	■■■■■■■■■■■■■■■■■■■■□□□□■■■■■■■■□□□□■■■■■■■	777777607760771	42	LAM9	-243---4-4-3	Orphan	243443242525224	Orphan	3	INH	unknown	P
FXX162010302013	2010	F	66	■■■■■■■■■■■■■■■■■■■■□□□□■■■■■■■■□□□□■■■■■■■	777777607760771	42	LAM9	-243---6-4-4	Orphan	243644243522225	Orphan	3	SM	Morocco	P
FXX162009101935	2009	F	71	■■■■■■■■■■■■■■■■■■■■□□□□■■■■■■■■□□□□■■■■■■■	777777607760771	42	LAM9	-243---8-3-2	Orphan	243832243524226	Orphan	1		Maghreb	P
FXX162010101996	2010	F	36	■■■■■■■■■■■■■■■■■■■■□□□□■■■■■■■■□□□□■■■■■■■	777777607760771	42	LAM9	-243---8-4-4	Orphan	243844243524226	345	1		unknown	P
FXX162010102022	2010	M	85	■■■■■■■■■■■■■■■■■■■■□□□□■■■■■■■■□□□□■■■■■■■	777777607760771	42	LAM9	-253---5-6-3	Orphan	253563336722244	Orphan	1		unknown	P
FXX162003100119	2003	M	82	■■■■■■■■■■■■■■■■■■■■□□□□■■■■■■■■□□□□■■■■■■■	777777607760771	42	LAM9					1		France	P
FXX162000100123	2000	M	26	■■■■■■■■■■■■■■■■■■■■□□□□■■■■■■■■□□□□■■■■■■■	777777607760771	42	LAM9					1		Morocco	P
FXX162000300146	2000	M	62	■■■■■■■■■■■■■■■■■■■■□□□□■■■■■■■■□□□□■■■■■■■	777777607760771	42	LAM9					3	SM	Algeria	P
FXX162002100187	2002	F	69	■■■■■■■■■■■■■■■■■■■■□□□□■■■■■■■■□□□□■■■■■■■	777777607760771	42	LAM9					1		Algeria	P
FXX162003100337	2003	F	41	■■■■■■■■■■■■■■■■■■■■□□□□■■■■■■■■□□□□■■■■■■■	777777607760771	42	LAM9					1		Algeria	P
FXX162000000394	2000	M	34	■■■■■■■■■■■■■■■■■■■■□□□□■■■■■■■■□□□□■■■■■■■	777777607760771	42	LAM9					0		Tunisia	P
FXX162003100412	2003	F	64	■■■■■■■■■■■■■■■■■■■■□□□□■■■■■■■■□□□□■■■■■■■	777777607760771	42	LAM9					1		Algeria	P
FXX162003100430	2003	F	67	■■■■■■■■■■■■■■■■■■■■□□□□■■■■■■■■□□□□■■■■■■■	777777607760771	42	LAM9					1		Algeria	P
FXX162004100458	2004	F	26	■■■■■■■■■■■■■■■■■■■■□□□□■■■■■■■■□□□□■■■■■■■	777777607760771	42	LAM9					1		Algeria	P
FXX162004100475	2004	M	25	■■■■■■■■■■■■■■■■■■■■□□□□■■■■■■■■□□□□■■■■■■■	777777607760771	42	LAM9					1		Cambodia	P
FXX162004100494	2004	M	30	■■■■■■■■■■■■■■■■■■■■□□□□■■■■■■■■□□□□■■■■■■■	777777607760771	42	LAM9					1		Azerbaijan	P
FXX162007100822	2007	M	27	■■■■■■■■■■■■■■■■■■■■□□□□■■■■■■■■□□□□■■■■■■■	777777607760771	42	LAM9	224325182424	345	243844---------	Orphan	1		unknown	P
FXX162008101435	2008	M	41	■■■■■■■■■■■■■■■■■■■■□□□□■■■■■■■■□□□□■■■■■■■	777777607760771	42	LAM9	-242---4-3-1	Orphan	242431244724312	Orphan	1		unknown	P

FXX162008101436	2008	F	25	■■■■■■■■■■■■■■■■■■■■□□□□■■■■■■■■□□□□■■■■■■■	777777607760771	42	LAM9	-242---5-3-1	Orphan	242531244825312	Orphan	1		Mexico	P
FXX162007101317	2007	F	56	■■■■■■■■■■■■■■■■■■■■□□□□■■■■■■■■□□□□■■■■■■■	777777607760771	42	LAM9	-243---8-4-4	Orphan	243844243524226	345	1		Maghreb	P
FXX162008101370	2008	M	80	■■■■■■■■■■■■■■■■■■■■□□□□■■■■■■■■□□□□■■■■■■■	777777607760771	42	LAM9	-243---8-4-4	Orphan	243844243524225	Orphan	1		Tunisia	P
FXX162008101542	2008	M	45	■■■■■■■■■■■■■■■■■■■■□□□□■■■■■■■■□□□□■■■■■■■	777777607760771	42	LAM9	-243---8-4-4	Orphan	243844243522226	346	1		unknown	P
FXX162008101595	2008	M	40	■■■■■■■■■■■■■■■■■■■■□□□□■■■■■■■■□□□□■■■■■■■	777777607760771	42	LAM9	-243---A-4-2	Orphan	243A42243524226	Orphan	1		unknown	P
FXX162007101321	2007	F	46	■■■■■■■■■■■■■■■■■■■■□□□□■■■■■■■■□□□□■■■■■■■	777777607760771	42	LAM9	-24------4-4	Orphan	24--44243524226	Orphan	1		Algeria	P
FXX162010102180	2010	M	48	■■■■■■■■■■■■■■■■■■■■□□□□■■■■■■■■□□□□■■■■■■■	777777607760771	42	LAM9	-243---5-2-6	Orphan	243526242722312	Orphan	1		unknown	EP and P
FXX162005100610	2005	M	66	■■■■■■■■■■■■■■■■■■■■□□□□■■■■■■■■□□□□■■■■■■■	777777607760771	42	LAM9	225326152324	Orphan	253534---------	Orphan	1		unknown	EP and P
FXX162007001255	2007	M	80	■■■■■■■■■■■■■■■■■■■■■■□■■■■■■■■■■□□□□■■■■■■■	777777757760771	44	T5	223025133322	Orphan	230332---------	Orphan	0		unknown	NA
FXX162007001162	2007	M	61	■■■■■■■■■■■■■■■■■■■■■■□■■■■■■■■■■□□□□■■■■■■■	777777757760771	44	T5	223326153323	236	233533---------	Orphan	0		unknown	NA
FXX162010002091	2010	M	82	■■■■■■■■■■■■■■■■■■■■■■□■■■■■■■■■■□□□□■■■■■■■	777777757760771	44	T5	-233---4-2-4	Orphan	23342434-322000	Orphan	0		unknown	NA
FXX162009002065	2009	F	85	■■■■■■■■■■■■■■■■■■■■■■□■■■■■■■■■■□□□□■■■■■■■	777777757760771	44	T5	-233---5-3-3	Orphan	233533343422224	Orphan	0		unknown	NA
FXX162010002134	2010	M	75	■■■■■■■■■■■■■■■■■■■■■■□■■■■■■■■■■□□□□■■■■■■■	777777757760771	44	T5	-233---5-3-4	Orphan	233534342522223	Orphan	0		unknown	NA
FXX162010002136	2010	F	75	■■■■■■■■■■■■■■■■■■■■■■□■■■■■■■■■■□□□□■■■■■■■	777777757760771	44	T5	-233---5-3-4	Orphan	233534441812222	Orphan	0		unknown	NA
FXX162003100138	2003	M	62	■■■■■■■■■■■■■■■■■■■■■■□■■■■■■■■■■□□□□■■■■■■■	777777757760771	44	T5					1		Madagascar	P
FXX162004100452	2004	F	27	■■■■■■■■■■■■■■■■■■■■■■□■■■■■■■■■■□□□□■■■■■■■	777777757760771	44	T5					1		Armenia	P
FXX162006100786	2006	F	78	■■■■■■■■■■■■■■■■■■■■■■□■■■■■■■■■■□□□□■■■■■■■	777777757760771	44	T5					1		France	P
FXX162007101290	2007	F	77	■■■■■■■■■■■■■■■■■■■■■■□■■■■■■■■■■□□□□■■■■■■■	777777757760771	44	T5	223326153322	289	233532---------	Orphan	1		unknown	EP and P
FXX162005000542	2005	F	63	■■■■■■■■■■■■■■■■■■■■■■■□■□□□□□□■□□□□■■■■■■■	777777764020771	45	H1	225313153325	860	253535---------	Orphan	0		France	P
FXX162001000005	2001	F	31	■■■■■■■■■■■■■■■■■■■■■■■■□□□□□□□□□□□□□□□□□□□	777777770000000	46	Unknown					0		Guinea	EP
FXX162005000726	2005	F	84	■■■■■■■■■■■■■■■■■■■■■■■■□□□□□□□□□□□□□□□□□□□	777777770000000	46	Unknown	224325143323	30	243433---------	Orphan	0		unknown	NA
FXX162005000699	2005	M	73	■■■■■■■■■■■■■■■■■■■■■■■■□□□□□□□□□□□□□□□□□□□	777777770000000	46	Unknown	225313153324	1093	253534---------	Orphan	0		unknown	NA
FXX162008001733	2008	F	32	■■■■■■■■■■■■■■■■■■■■■■■■□□□□□□□□□□□□□□□□□□□	777777770000000	46	Unknown	-234---5-3-2	Orphan	234532245522223	313	0		unknown	NA
FXX162008001712	2008	M	45	■■■■■■■■■■■■■■■■■■■■■■■■□□□□□□□□□□□□□□□□□□□	777777770000000	46	Unknown	-253---5-3-2	Orphan	253532333532343	354	0		unknown	NA
FXX162010102057	2010	F	40	■■■■■■■■■■■■■■■■■■■■■■■■□□□□□□□□□□□□□□□□□□□	777777770000000	46	Unknown	-223---5-3-3	Orphan	223533352422324	Orphan	1		Chad	P
FXX162010102259	2010	M	64	■■■■■■■■■■■■■■■■■■■■■■■■□□□□□□□□□□□□□□□□□□□	777777770000000	46	Unknown	-233---5-3-2	Orphan	233532244522223	305	1		unknown	P
FXX162008101592	2008	F	15	■■■■■■■■■■■■■■■■■■■■■■■■□□□□□□□□□□□□□□□□□□□	777777770000000	46	Unknown	-253---3-3-3	Orphan	253333333534343	Orphan	1		unknown	P
FXX162003100345	2003	F	45	■■■■■■■■■■■■■■■■■■■■■■■■□□□□□□□□□□□□□□□□□□□	777777770000000	46	Unknown					1		Madagascar	P
FXX162004300438	2004	M	27	■■■■■■■■■■■■■■■■■■■■■■■■□□□□□□□□□□□□□□□□□□□	777777770000000	46	Unknown					3	SM	France	P
FXX162008101672	2008	F	34	■■■■■■■■■■■■■■■■■■■■■■■■□□□□□□□□□□□□□□□□□□□	777777770000000	46	Unknown	-253---3-3-3	Orphan	253333332434243	Orphan	1		Cameroon	P
FXX162007101206	2007	F	58	■■■■■■■■■■■■■■■■■■■■■■■■■□□□□□□■□□□□■■■■■■■	777777774020771	47	H1	225323153323	43	253533---------	Orphan	1		Tunisia	EP
FXX162008101555	2008	M	81	■■■■■■■■■■■■■■■■■■■■■■■■■□□□□□□■□□□□■■■■■■■	777777774020771	47	H1	-243---5-3-3	Orphan	243533233732343	Orphan	1		unknown	EP
FXX162009101742	2009	F	37	■■■■■■■■■■■■■■■■■■■■■■■■■□□□□□□■□□□□■■■■■■■	777777774020771	47	H1	-253---5-3-2	Orphan	253532334622243	356	1		Maghreb	EP
FXX162010102000	2010	M	57	■■■■■■■■■■■■■■■■■■■■■■■■■□□□□□□■□□□□■■■■■■■	777777774020771	47	H1	-253---5-3-3	Orphan	253533335732343	363	1		unknown	EP
FXX162010102196	2010	F	59	■■■■■■■■■■■■■■■■■■■■■■■■■□□□□□□■□□□□■■■■■■■	777777774020771	47	H1	-253---5-3-3	Orphan	253533334232343	Orphan	1		unknown	EP
FXX162001300002	2001	M	22	■■■■■■■■■■■■■■■■■■■■■■■■■□□□□□□■□□□□■■■■■■■	777777774020771	47	H1					3	SM	Dem Republic Congo	EP
FXX162000100342	2000	F	17	■■■■■■■■■■■■■■■■■■■■■■■■■□□□□□□■□□□□■■■■■■■	777777774020771	47	H1					1		Tunisia	EP
FXX162004300474	2004	M	27	■■■■■■■■■■■■■■■■■■■■■■■■■□□□□□□■□□□□■■■■■■■	777777774020771	47	H1					3	SM, INH	Morocco	EP
FXX162006000868	2006	M	77	■■■■■■■■■■■■■■■■■■■■■■■■■□□□□□□■□□□□■■■■■■■	777777774020771	47	H1	215325153323	776	153533---------	Orphan	0		unknown	NA
FXX162005000666	2005	F	81	■■■■■■■■■■■■■■■■■■■■■■■■■□□□□□□■□□□□■■■■■■■	777777774020771	47	H1	223225173324	Orphan	232734---------	Orphan	0		unknown	NA
FXX162007001143	2007	M	84	■■■■■■■■■■■■■■■■■■■■■■■■■□□□□□□■□□□□■■■■■■■	777777774020771	47	H1	224315153323	27	243533---------	Orphan	0		unknown	NA
FXX162006000847	2006	M	66	■■■■■■■■■■■■■■■■■■■■■■■■■□□□□□□■□□□□■■■■■■■	777777774020771	47	H1	224325153322	32	243532---------	Orphan	0		unknown	NA
FXX162007001193	2007	F	25	■■■■■■■■■■■■■■■■■■■■■■■■■□□□□□□■□□□□■■■■■■■	777777774020771	47	H1	225323153323	43	253533334632343	199	0		unknown	NA
FXX162005000603	2005	F	81	■■■■■■■■■■■■■■■■■■■■■■■■■□□□□□□■□□□□■■■■■■■	777777774020771	47	H1	225323153322	1582	253532---------	Orphan	0		unknown	NA
FXX162007001133	2007	F	28	■■■■■■■■■■■■■■■■■■■■■■■■■□□□□□□■□□□□■■■■■■■	777777774020771	47	H1	225323153323	43	253533---------	Orphan	0		unknown	NA
FXX162007001233	2007	F	60	■■■■■■■■■■■■■■■■■■■■■■■■■□□□□□□■□□□□■■■■■■■	777777774020771	47	H1	225323153323	43	253533---------	Orphan	0		unknown	NA
FXX162007001218	2007	M	81	■■■■■■■■■■■■■■■■■■■■■■■■■□□□□□□■□□□□■■■■■■■	777777774020771	47	H1	225325151323	762	253533---------	Orphan	0		unknown	NA
FXX162007001240	2007	F	36	■■■■■■■■■■■■■■■■■■■■■■■■■□□□□□□■□□□□■■■■■■■	777777774020771	47	H1	225325153322	152	253532---------	Orphan	0		unknown	NA
FXX162006000926	2006	M	56	■■■■■■■■■■■■■■■■■■■■■■■■■□□□□□□■□□□□■■■■■■■	777777774020771	47	H1	225325153323	45	253533---------	Orphan	0		unknown	NA
FXX162006001000	2006	M	50	■■■■■■■■■■■■■■■■■■■■■■■■■□□□□□□■□□□□■■■■■■■	777777774020771	47	H1	225325153323	45	253533---------	Orphan	0		unknown	NA
FXX162006001039	2006	M	80	■■■■■■■■■■■■■■■■■■■■■■■■■□□□□□□■□□□□■■■■■■■	777777774020771	47	H1	225325153323	45	253533---------	Orphan	0		unknown	NA
FXX162007001094	2007	M	25	■■■■■■■■■■■■■■■■■■■■■■■■■□□□□□□■□□□□■■■■■■■	777777774020771	47	H1	225325153323	45	253533---------	Orphan	0		unknown	NA
FXX162007001228	2007	F	81	■■■■■■■■■■■■■■■■■■■■■■■■■□□□□□□■□□□□■■■■■■■	777777774020771	47	H1	225325153323	45	253533---------	Orphan	0		unknown	NA
FXX162007001232	2007	F	75	■■■■■■■■■■■■■■■■■■■■■■■■■□□□□□□■□□□□■■■■■■■	777777774020771	47	H1	225325153323	45	253533---------	Orphan	0		unknown	NA
FXX162007001179	2007	F	22	■■■■■■■■■■■■■■■■■■■■■■■■■□□□□□□■□□□□■■■■■■■	777777774020771	47	H1	225325154323	182	253533---------	Orphan	0		unknown	NA
FXX162007001142	2007	M	80	■■■■■■■■■■■■■■■■■■■■■■■■■□□□□□□■□□□□■■■■■■■	777777774020771	47	H1	225326153323	247	253533---------	Orphan	0		unknown	NA
FXX162006001002	2006	M	32	■■■■■■■■■■■■■■■■■■■■■■■■■□□□□□□■□□□□■■■■■■■	777777774020771	47	H1	226313153323	290	263533---------	Orphan	0		unknown	NA

FXX162007001096	2007	F	22	■■■■■■■■■■■■■■■■■■■■■■■■■□□□□□□■□□□□■■■■■■■	777777774020771	47	H1	226313153323	290	263533---------	Orphan	0		unknown	NA
FXX162009002071	2009	F	73	■■■■■■■■■■■■■■■■■■■■■■■■■□□□□□□■□□□□■■■■■■■	777777774020771	47	H1	-233---4-4-3	Orphan	233443336732343	Orphan	0		unknown	NA
FXX162010002217	2010	M	52	■■■■■■■■■■■■■■■■■■■■■■■■■□□□□□□■□□□□■■■■■■■	777777774020771	47	H1	-243---5-2-3	Orphan	243523336722243	Orphan	0		unknown	NA
FXX162009001883	2009	M	35	■■■■■■■■■■■■■■■■■■■■■■■■■□□□□□□■□□□□■■■■■■■	777777774020771	47	H1	-252---5-3-3	Orphan	252533336732333	Orphan	0		unknown	NA
FXX162008001586	2008	F	39	■■■■■■■■■■■■■■■■■■■■■■■■■□□□□□□■□□□□■■■■■■■	777777774020771	47	H1	-253---5-3-3	Orphan	253533336722243	359	0		unknown	NA
FXX162008001719	2008	F	56	■■■■■■■■■■■■■■■■■■■■■■■■■□□□□□□■□□□□■■■■■■■	777777774020771	47	H1	-253---5-3-3	Orphan	253533336732333	365	0		unknown	NA
FXX162008001730	2008	M	29	■■■■■■■■■■■■■■■■■■■■■■■■■□□□□□□■□□□□■■■■■■■	777777774020771	47	H1	-253---5-3-3	Orphan	253533335731342	Orphan	0		unknown	NA
FXX162008001740	2008	M	82	■■■■■■■■■■■■■■■■■■■■■■■■■□□□□□□■□□□□■■■■■■■	777777774020771	47	H1	-253---5-3-3	Orphan	253533335731343	Orphan	0		unknown	NA
FXX162009001920	2009	M	27	■■■■■■■■■■■■■■■■■■■■■■■■■□□□□□□■□□□□■■■■■■■	777777774020771	47	H1	-253---5-3-3	Orphan	253533339532243	Orphan	0		unknown	NA
FXX162009001923	2009	M	59	■■■■■■■■■■■■■■■■■■■■■■■■■□□□□□□■□□□□■■■■■■■	777777774020771	47	H1	-253---5-3-3	Orphan	253533335732343	363	0		unknown	NA
FXX162010002085	2010	M	71	■■■■■■■■■■■■■■■■■■■■■■■■■□□□□□□■□□□□■■■■■■■	777777774020771	47	H1	-253---5-3-3	Orphan	253533333512341	Orphan	0		unknown	NA
FXX162010002087	2010	M	84	■■■■■■■■■■■■■■■■■■■■■■■■■□□□□□□■□□□□■■■■■■■	777777774020771	47	H1	-253---5-3-3	Orphan	253533333532343	361	0		unknown	NA
FXX162009001848	2009	M	57	■■■■■■■■■■■■■■■■■■■■■■■■■□□□□□□■□□□□■■■■■■■	777777774020771	47	H1	-262---5-3-2	Orphan	262532233532343	Orphan	0		unknown	NA
FXX162009002029	2009	F	51	■■■■■■■■■■■■■■■■■■■■■■■■■□□□□□□■□□□□■■■■■■■	777777774020771	47	H1	-263---5-3-2	Orphan	263532334632243	Orphan	0		unknown	NA
FXX162008001700	2008	F	26	■■■■■■■■■■■■■■■■■■■■■■■■■□□□□□□■□□□□■■■■■■■	777777774020771	47	H1	-263---6-3-3	Orphan	263633333722243	Orphan	0		unknown	NA
FXX162007001125	2007	F	86	■■■■■■■■■■■■■■■■■■■■■■■■■□□□□□□■□□□□■■■■■■■	777777774020771	47	H1	224325153312	1408	243532---------	Orphan	0		unknown	P
FXX162005300678	2005	F	33	■■■■■■■■■■■■■■■■■■■■■■■■■□□□□□□■□□□□■■■■■■■	777777774020771	47	H1	225225153323	188	252533---------	Orphan	3	INH	France	P
FXX162005100526	2005	F	33	■■■■■■■■■■■■■■■■■■■■■■■■■□□□□□□■□□□□■■■■■■■	777777774020771	47	H1	225323153323	43	253533---------	Orphan	1		Morocco	P
FXX162005100631	2005	M	58	■■■■■■■■■■■■■■■■■■■■■■■■■□□□□□□■□□□□■■■■■■■	777777774020771	47	H1	225323153323	43	253533---------	Orphan	1		Tunisia	P
FXX162005100589	2005	M	76	■■■■■■■■■■■■■■■■■■■■■■■■■□□□□□□■□□□□■■■■■■■	777777774020771	47	H1	225325153323	45	253533---------	Orphan	1		France	P
FXX162006100743	2006	F	27	■■■■■■■■■■■■■■■■■■■■■■■■■□□□□□□■□□□□■■■■■■■	777777774020771	47	H1	226313163323	545	263633---------	Orphan	1		France	P
FXX162010102056	2010	M	83	■■■■■■■■■■■■■■■■■■■■■■■■■□□□□□□■□□□□■■■■■■■	777777774020771	47	H1	-243---5-3-3	Orphan	243533331832343	Orphan	1		unknown	P
FXX162010102093	2010	M	25	■■■■■■■■■■■■■■■■■■■■■■■■■□□□□□□■□□□□■■■■■■■	777777774020771	47	H1	-253---5-3-3	Orphan	253533332532243	Orphan	1		unknown	P
FXX162010102200	2010	F	32	■■■■■■■■■■■■■■■■■■■■■■■■■□□□□□□■□□□□■■■■■■■	777777774020771	47	H1	-253---5-3-3	Orphan	253533333432343	Orphan	1		unknown	P
FXX162010102261	2010	M	66	■■■■■■■■■■■■■■■■■■■■■■■■■□□□□□□■□□□□■■■■■■■	777777774020771	47	H1	-253---5-3-4	Orphan	253534334732343	375	1		unknown	P
FXX162001100044	2001	M	33	■■■■■■■■■■■■■■■■■■■■■■■■■□□□□□□■□□□□■■■■■■■	777777774020771	47	H1					1		France	P
FXX162001100056	2001	M	45	■■■■■■■■■■■■■■■■■■■■■■■■■□□□□□□■□□□□■■■■■■■	777777774020771	47	H1					1		Turkey	P
FXX162002100188	2002	M	80	■■■■■■■■■■■■■■■■■■■■■■■■■□□□□□□■□□□□■■■■■■■	777777774020771	47	H1					1		France	P
FXX162002100233	2002	M	27	■■■■■■■■■■■■■■■■■■■■■■■■■□□□□□□■□□□□■■■■■■■	777777774020771	47	H1					1		Romania	P
FXX162002100244	2002	M	38	■■■■■■■■■■■■■■■■■■■■■■■■■□□□□□□■□□□□■■■■■■■	777777774020771	47	H1					1		Tunisia	P
FXX162000100264	2000	M	57	■■■■■■■■■■■■■■■■■■■■■■■■■□□□□□□■□□□□■■■■■■■	777777774020771	47	H1					1		Algeria	P
FXX162000100268	2000	F	67	■■■■■■■■■■■■■■■■■■■■■■■■■□□□□□□■□□□□■■■■■■■	777777774020771	47	H1					1		France	P
FXX162002100291	2002	M	18	■■■■■■■■■■■■■■■■■■■■■■■■■□□□□□□■□□□□■■■■■■■	777777774020771	47	H1					1		Turkey	P
FXX162003100346	2003	M	34	■■■■■■■■■■■■■■■■■■■■■■■■■□□□□□□■□□□□■■■■■■■	777777774020771	47	H1					1		France	P
FXX162003000347	2003	F	26	■■■■■■■■■■■■■■■■■■■■■■■■■□□□□□□■□□□□■■■■■■■	777777774020771	47	H1					0		Central African Republic	P
FXX162001300352	2001	F	28	■■■■■■■■■■■■■■■■■■■■■■■■■□□□□□□■□□□□■■■■■■■	777777774020771	47	H1					3	SM	Algeria	P
FXX162003100424	2003	M	56	■■■■■■■■■■■■■■■■■■■■■■■■■□□□□□□■□□□□■■■■■■■	777777774020771	47	H1					1		Gabon	P
FXX162004100451	2004	F	89	■■■■■■■■■■■■■■■■■■■■■■■■■□□□□□□■□□□□■■■■■■■	777777774020771	47	H1					1		France	P
FXX162004100454	2004	M	97	■■■■■■■■■■■■■■■■■■■■■■■■■□□□□□□■□□□□■■■■■■■	777777774020771	47	H1					1		France	P
FXX162004100478	2004	F	42	■■■■■■■■■■■■■■■■■■■■■■■■■□□□□□□■□□□□■■■■■■■	777777774020771	47	H1					1		Yugoslavia	P
FXX162004100506	2004	M	66	■■■■■■■■■■■■■■■■■■■■■■■■■□□□□□□■□□□□■■■■■■■	777777774020771	47	H1					1		France	P
FXX162006000767	2006	M	22	■■■■■■■■■■■■■■■■■■■■■■■■■□□□□□□■□□□□■■■■■■■	777777774020771	47	H1					0		Congo	P
FXX162007101204	2007	M	43	■■■■■■■■■■■■■■■■■■■■■■■■■□□□□□□■□□□□■■■■■■■	777777774020771	47	H1	225323153324	222	253534---------	Orphan	1		Turkey	P
FXX162007101319	2007	F	69	■■■■■■■■■■■■■■■■■■■■■■■■■□□□□□□■□□□□■■■■■■■	777777774020771	47	H1	-253---5-3-3	Orphan	253533337333243	Orphan	1		unknown	P
FXX162009101841	2009	F	50	■■■■■■■■■■■■■■■■■■■■■■■■■■■■□□□□■□■■■■■□■■■	777777777413731	48	EAI1-SOM	-742---2-5-4	Orphan	742254642612524	Orphan	1		Laos	EP
FXX162003100311	2003	F	42	■■■■■■■■■■■■■■■■■■■■■■■■■■■■□□□□■□■■■■■□■■■	777777777413731	48	EAI1-SOM					1		Ethiopia	EP
FXX162008101330	2008	M	72	■■■■■■■■■■■■■■■■■■■■■■■■■■■■□□□□■□■■■■■□■■■	777777777413731	48	EAI1-SOM	-742---2-5-3	Orphan	742253642612524	393	1		Cambodia	P
FXX162008101565	2008	M	82	■■■■■■■■■■■■■■■■■■■■■■■■■■■■□□□□■□■■■■■□■■■	777777777413731	48	EAI1-SOM	-742---2-5-3	Orphan	742253642612524	393	1		unknown	P
FXX162008101622	2008	M	21	■■■■■■■■■■■■■■■■■■■■■■■■■■■■□□□□■□■■■■■□■■■	777777777413731	48	EAI1-SOM	-752---2-5-3	Orphan	752253642612524	Orphan	1		unknown	P
FXX162005100657	2005	M	22	■■■■■■■■■■■■■■■■■■■■■■■■■■■■□□□□■□■■■■■□■■■	777777777413731	48	EAI1-SOM	274225223433	864	742243---------	Orphan	1		Cambodia	EP and P
FXX162001100040	2001	F	19	■■■■■■■■■■■■■■■■■■■■■■■■■■■■■■□■□□□□■■■□■■■	777777777720731	49	H3					1		Cameroon	EP
FXX162006000810	2006	M	51	■■■■■■■■■■■■■■■■■■■■■■■■■■■■■■□■□□□□■■■□■■■	777777777720731	49	H3	225313153322	184	253532---------	Orphan	0		unknown	NA
FXX162009001863	2009	F	46	■■■■■■■■■■■■■■■■■■■■■■■■■■■■■■□■□□□□■■■□■■■	777777777720731	49	H3	-153---5-3-3	Orphan	153533235622243	Orphan	0		unknown	NA
FXX162008001480	2008	F	47	■■■■■■■■■■■■■■■■■■■■■■■■■■■■■■□■□□□□■■■□■■■	777777777720731	49	H3	-233---3-3-3	Orphan	233333351422322	Orphan	0		unknown	NA
FXX162008001509	2008	F	103	■■■■■■■■■■■■■■■■■■■■■■■■■■■■■■□■□□□□■■■□■■■	777777777720731	49	H3	-233---3-3-3	Orphan	23333335142232A	Orphan	0		unknown	NA
FXX162009002041	2009	F	18	■■■■■■■■■■■■■■■■■■■■■■■■■■■■■■□■□□□□■■■□■■■	777777777720731	49	H3	-233---3-3-3	Orphan	233333351422329	Orphan	0		unknown	NA
FXX162009001986	2009	M	83	■■■■■■■■■■■■■■■■■■■■■■■■■■■■■■□■□□□□■■■□■■■	777777777720731	49	H3	-243---5-3-3	Orphan	243533336722243	Orphan	0		unknown	NA

FXX162010002167	2010	F	49	■■■■■■■■■■■■■■■■■■■■■■■■■■■■■■□■□□□□■■■□■■■	777777777720731	49	H3	-253---5-3-3	Orphan	253533335-32343	Orphan	0		unknown	NA
FXX162008001572	2008	F	40	■■■■■■■■■■■■■■■■■■■■■■■■■■■■■■□■□□□□■■■□■■■	777777777720731	49	H3	-2-3---5-3-3	Orphan	2-3533336-22243	Orphan	0		unknown	NA
FXX162006300821	2006	M	80	■■■■■■■■■■■■■■■■■■■■■■■■■■■■■■□■□□□□■■■□■■■	777777777720731	49	H3	222313153223	Orphan	223523---------	Orphan	3	INH	France	P
FXX162006101023	2006	F	29	■■■■■■■■■■■■■■■■■■■■■■■■■■■■■■□■□□□□■■■□■■■	777777777720731	49	H3	224225153323	22	242533---------	Orphan	1		unknown	P
FXX162005000682	2005	F	78	■■■■■■■■■■■■■■■■■■■■■■■■■■■■■■□■□□□□■■■□■■■	777777777720731	49	H3	225313152323	1580	253533---------	Orphan	0		France	P
FXX162006301028	2006	F		■■■■■■■■■■■■■■■■■■■■■■■■■■■■■■□■□□□□■■■□■■■	777777777720731	49	H3	225323153323	43	253533---------	Orphan	3	SM	Algeria	P
FXX162008101668	2008	M	74	■■■■■■■■■■■■■■■■■■■■■■■■■■■■■■□■□□□□■■■□■■■	777777777720731	49	H3	-253---5-3-3	Orphan	253533334632343	199	1		France	P
FXX162007101150	2007	M	21	■■■■■■■■■■■■■■■■■■■■■■■■■■■■■■□■□□□□■■■■■■■	777777777720771	50	H3	224325153323	33	243533---------	Orphan	1		Mali	EP
FXX162005100536	2005	M	85	■■■■■■■■■■■■■■■■■■■■■■■■■■■■■■□■□□□□■■■■■■■	777777777720771	50	H3	225125153323	785	251533---------	Orphan	1		France	EP
FXX162005100577	2005	F	62	■■■■■■■■■■■■■■■■■■■■■■■■■■■■■■□■□□□□■■■■■■■	777777777720771	50	H3	225213153322	Orphan	252532---------	Orphan	1		Algeria	EP
FXX162005100605	2005	M	77	■■■■■■■■■■■■■■■■■■■■■■■■■■■■■■□■□□□□■■■■■■■	777777777720771	50	H3	225313153323	42	253533---------	Orphan	1		Algeria	EP
FXX162000100163	2000	M	58	■■■■■■■■■■■■■■■■■■■■■■■■■■■■■■□■□□□□■■■■■■■	777777777720771	50	H3	225323153323	43	253533---------	Orphan	1		Maghreb	EP
FXX162006100815	2006	M	54	■■■■■■■■■■■■■■■■■■■■■■■■■■■■■■□■□□□□■■■■■■■	777777777720771	50	H3	225323153323	43	253533---------	Orphan	1		Algeria	EP
FXX162005000638	2005	F	91	■■■■■■■■■■■■■■■■■■■■■■■■■■■■■■□■□□□□■■■■■■■	777777777720771	50	H3	225325153323	45	253533---------	Orphan	0		France	EP
FXX162006100959	2006	M	79	■■■■■■■■■■■■■■■■■■■■■■■■■■■■■■□■□□□□■■■■■■■	777777777720771	50	H3	225325153323	45	253533---------	Orphan	1		unknown	EP
FXX162007101073	2007	F	70	■■■■■■■■■■■■■■■■■■■■■■■■■■■■■■□■□□□□■■■■■■■	777777777720771	50	H3	225325153323	45	253533---------	Orphan	1		unknown	EP
FXX162006100781	2006	M	33	■■■■■■■■■■■■■■■■■■■■■■■■■■■■■■□■□□□□■■■■■■■	777777777720771	50	H3	235323153323	1239	353533---------	Orphan	1		Côte d'Ivoire	EP
FXX162009101792	2009	M	56	■■■■■■■■■■■■■■■■■■■■■■■■■■■■■■□■□□□□■■■■■■■	777777777720771	50	H3	-243---5-3-3	Orphan	243533335722243	Orphan	1		unknown	EP
FXX162010102360	2010	F	30	■■■■■■■■■■■■■■■■■■■■■■■■■■■■■■□■□□□□■■■■■■■	777777777720771	50	H3	-253---5-3-2	Orphan	253532333522342	Orphan	1		Guinea	EP
FXX162010101967	2010	F	2	■■■■■■■■■■■■■■■■■■■■■■■■■■■■■■□■□□□□■■■■■■■	777777777720771	50	H3	-253---5-3-3	Orphan	253533333522323	Orphan	1		unknown	EP
FXX162010102062	2010	F	79	■■■■■■■■■■■■■■■■■■■■■■■■■■■■■■□■□□□□■■■■■■■	777777777720771	50	H3	-253---5-3-3	Orphan	253533336532243	Orphan	1		unknown	EP
FXX162001300035	2001	M	31	■■■■■■■■■■■■■■■■■■■■■■■■■■■■■■□■□□□□■■■■■■■	777777777720771	50	H3					3	SM	Guinea	EP
FXX162001100042	2001	M	44	■■■■■■■■■■■■■■■■■■■■■■■■■■■■■■□■□□□□■■■■■■■	777777777720771	50	H3					1		France	EP
FXX162001100057	2001	F	84	■■■■■■■■■■■■■■■■■■■■■■■■■■■■■■□■□□□□■■■■■■■	777777777720771	50	H3					1		France	EP
FXX162002100175	2002	M	49	■■■■■■■■■■■■■■■■■■■■■■■■■■■■■■□■□□□□■■■■■■■	777777777720771	50	H3					1		Guinea	EP
FXX162002100191	2002	F	42	■■■■■■■■■■■■■■■■■■■■■■■■■■■■■■□■□□□□■■■■■■■	777777777720771	50	H3					1		Morocco	EP
FXX162002100219	2002	F	74	■■■■■■■■■■■■■■■■■■■■■■■■■■■■■■□■□□□□■■■■■■■	777777777720771	50	H3					1		France	EP
FXX162002100236	2002	M	72	■■■■■■■■■■■■■■■■■■■■■■■■■■■■■■□■□□□□■■■■■■■	777777777720771	50	H3					1		France	EP
FXX162003100255	2003	M	34	■■■■■■■■■■■■■■■■■■■■■■■■■■■■■■□■□□□□■■■■■■■	777777777720771	50	H3					1		India	EP
FXX162000100267	2000	F	42	■■■■■■■■■■■■■■■■■■■■■■■■■■■■■■□■□□□□■■■■■■■	777777777720771	50	H3					1		Morocco	EP
FXX162003100306	2003	F	64	■■■■■■■■■■■■■■■■■■■■■■■■■■■■■■□■□□□□■■■■■■■	777777777720771	50	H3					1		Tunisia	EP
FXX162000100329	2000	M	71	■■■■■■■■■■■■■■■■■■■■■■■■■■■■■■□■□□□□■■■■■■■	777777777720771	50	H3					1		France	EP
FXX162000300351	2000	F	73	■■■■■■■■■■■■■■■■■■■■■■■■■■■■■■□■□□□□■■■■■■■	777777777720771	50	H3					3	ETH	Algeria	EP
FXX162000000367	2000	F	50	■■■■■■■■■■■■■■■■■■■■■■■■■■■■■■□■□□□□■■■■■■■	777777777720771	50	H3					0		Algeria	EP
FXX162004100516	2004	M	30	■■■■■■■■■■■■■■■■■■■■■■■■■■■■■■□■□□□□■■■■■■■	777777777720771	50	H3					1		Benin	EP
FXX162006000928	2006	F	14	■■■■■■■■■■■■■■■■■■■■■■■■■■■■■■□■□□□□■■■■■■■	777777777720771	50	H3	222325132326	Orphan	223336---------	Orphan	0		unknown	NA
FXX162007001238	2007	F	85	■■■■■■■■■■■■■■■■■■■■■■■■■■■■■■□■□□□□■■■■■■■	777777777720771	50	H3	223313153323	837	233533---------	Orphan	0		unknown	NA
FXX162006001052	2006	M	19	■■■■■■■■■■■■■■■■■■■■■■■■■■■■■■□■□□□□■■■■■■■	777777777720771	50	H3	224325153323	33	243533---------	Orphan	0		unknown	NA
FXX162007001189	2007	M	79	■■■■■■■■■■■■■■■■■■■■■■■■■■■■■■□■□□□□■■■■■■■	777777777720771	50	H3	224325153323	33	243533---------	Orphan	0		unknown	NA
FXX162007001091	2007	F	75	■■■■■■■■■■■■■■■■■■■■■■■■■■■■■■□■□□□□■■■■■■■	777777777720771	50	H3	225225153323	188	252533---------	Orphan	0		unknown	NA
FXX162006000804	2006	M	33	■■■■■■■■■■■■■■■■■■■■■■■■■■■■■■□■□□□□■■■■■■■	777777777720771	50	H3	225225153325	1579	252535---------	Orphan	0		unknown	NA
FXX162006000855	2006	M	76	■■■■■■■■■■■■■■■■■■■■■■■■■■■■■■□■□□□□■■■■■■■	777777777720771	50	H3	225312153321	Orphan	253531---------	Orphan	0		unknown	NA
FXX162005000706	2005	M	23	■■■■■■■■■■■■■■■■■■■■■■■■■■■■■■□■□□□□■■■■■■■	777777777720771	50	H3	225313153223	810	253523---------	Orphan	0		unknown	NA
FXX162006000849	2006	F	21	■■■■■■■■■■■■■■■■■■■■■■■■■■■■■■□■□□□□■■■■■■■	777777777720771	50	H3	225313153223	810	253523---------	Orphan	0		unknown	NA
FXX162006000965	2006	F	89	■■■■■■■■■■■■■■■■■■■■■■■■■■■■■■□■□□□□■■■■■■■	777777777720771	50	H3	225313153321	174	253531---------	Orphan	0		unknown	NA
FXX162007301095	2007	M	77	■■■■■■■■■■■■■■■■■■■■■■■■■■■■■■□■□□□□■■■■■■■	777777777720771	50	H3	225313153322	184	253532---------	Orphan	3	SM	unknown	NA
FXX162006000906	2006	M	69	■■■■■■■■■■■■■■■■■■■■■■■■■■■■■■□■□□□□■■■■■■■	777777777720771	50	H3	225313153323	42	253533---------	Orphan	0		unknown	NA
FXX162006000908	2006	F	78	■■■■■■■■■■■■■■■■■■■■■■■■■■■■■■□■□□□□■■■■■■■	777777777720771	50	H3	225313153323	42	253533---------	Orphan	0		unknown	NA
FXX162007001078	2007	F	67	■■■■■■■■■■■■■■■■■■■■■■■■■■■■■■□■□□□□■■■■■■■	777777777720771	50	H3	225313153323	42	253533---------	Orphan	0		unknown	NA
FXX162007001194	2007	M		■■■■■■■■■■■■■■■■■■■■■■■■■■■■■■□■□□□□■■■■■■■	777777777720771	50	H3	225313153323	42	253533---------	Orphan	0		unknown	NA
FXX162007001262	2007	M	84	■■■■■■■■■■■■■■■■■■■■■■■■■■■■■■□■□□□□■■■■■■■	777777777720771	50	H3	225313153323	42	253533---------	Orphan	0		unknown	NA
FXX162007001283	2007	M	73	■■■■■■■■■■■■■■■■■■■■■■■■■■■■■■□■□□□□■■■■■■■	777777777720771	50	H3	225319153323	Orphan	253533---------	Orphan	0		unknown	NA
FXX162005000707	2005	M	48	■■■■■■■■■■■■■■■■■■■■■■■■■■■■■■□■□□□□■■■■■■■	777777777720771	50	H3	225323153322	1582	253532---------	Orphan	0		unknown	NA
FXX162007001226	2007	M	15	■■■■■■■■■■■■■■■■■■■■■■■■■■■■■■□■□□□□■■■■■■■	777777777720771	50	H3	225323153323	43	253533---------	Orphan	0		unknown	NA
FXX162007001277	2007	M	50	■■■■■■■■■■■■■■■■■■■■■■■■■■■■■■□■□□□□■■■■■■■	777777777720771	50	H3	225323153323	43	253533---------	Orphan	0		unknown	NA
FXX162007001135	2007	M	87	■■■■■■■■■■■■■■■■■■■■■■■■■■■■■■□■□□□□■■■■■■■	777777777720771	50	H3	225323163223	Orphan	253623---------	Orphan	0		unknown	NA
FXX162006000802	2006	F	79	■■■■■■■■■■■■■■■■■■■■■■■■■■■■■■□■□□□□■■■■■■■	777777777720771	50	H3	225325153323	45	253533---------	Orphan	0		unknown	NA

FXX162006000887	2006	M	45	■■■■■■■■■■■■■■■■■■■■■■■■■■■■■■□■□□□□■■■■■■■	777777777720771	50	H3	225325153323	45	253533---------	Orphan	0		unknown	NA
FXX162006000895	2006	M	47	■■■■■■■■■■■■■■■■■■■■■■■■■■■■■■□■□□□□■■■■■■■	777777777720771	50	H3	225325153323	45	253533---------	Orphan	0		unknown	NA
FXX162006000998	2006	F	58	■■■■■■■■■■■■■■■■■■■■■■■■■■■■■■□■□□□□■■■■■■■	777777777720771	50	H3	225325153323	45	253533---------	Orphan	0		unknown	NA
FXX162007001077	2007	M	27	■■■■■■■■■■■■■■■■■■■■■■■■■■■■■■□■□□□□■■■■■■■	777777777720771	50	H3	225325153323	45	253533---------	Orphan	0		unknown	NA
FXX162007001224	2007	M	75	■■■■■■■■■■■■■■■■■■■■■■■■■■■■■■□■□□□□■■■■■■■	777777777720771	50	H3	225325153324	46	253534---------	Orphan	0		unknown	NA
FXX162005000646	2005	M	44	■■■■■■■■■■■■■■■■■■■■■■■■■■■■■■□■□□□□■■■■■■■	777777777720771	50	H3	225325154323	182	253533---------	Orphan	0		unknown	NA
FXX162007001066	2007	M	4	■■■■■■■■■■■■■■■■■■■■■■■■■■■■■■□■□□□□■■■■■■■	777777777720771	50	H3	2253-3153323	Orphan	253533---------	Orphan	0		unknown	NA
FXX162005000654	2005	F	78	■■■■■■■■■■■■■■■■■■■■■■■■■■■■■■□■□□□□■■■■■■■	777777777720771	50	H3	226313153323	290	263533---------	Orphan	0		unknown	NA
FXX162008001679	2008	F	79	■■■■■■■■■■■■■■■■■■■■■■■■■■■■■■□■□□□□■■■■■■■	777777777720771	50	H3	-153---5-3-3	Orphan	153533234632343	Orphan	0		unknown	NA
FXX162008001729	2008	F		■■■■■■■■■■■■■■■■■■■■■■■■■■■■■■□■□□□□■■■■■■■	777777777720771	50	H3	-213---5-3-3	Orphan	213533335733355	Orphan	0		unknown	NA
FXX162010002227	2010	F	73	■■■■■■■■■■■■■■■■■■■■■■■■■■■■■■□■□□□□■■■■■■■	777777777720771	50	H3	-223---4-3-3	Orphan	223433336732243	Orphan	0		unknown	NA
FXX162009001892	2009	F	86	■■■■■■■■■■■■■■■■■■■■■■■■■■■■■■□■□□□□■■■■■■■	777777777720771	50	H3	-233---5-3-3	Orphan	233533333732253	Orphan	0		unknown	NA
FXX162009001901	2009	F	90	■■■■■■■■■■■■■■■■■■■■■■■■■■■■■■□■□□□□■■■■■■■	777777777720771	50	H3	-243---9-3-3	Orphan	243933242733312	Orphan	0		unknown	NA
FXX162010002338	2010	F	64	■■■■■■■■■■■■■■■■■■■■■■■■■■■■■■□■□□□□■■■■■■■	777777777720771	50	H3	-253---5-3-3	Orphan	253533235723243	351	0		unknown	NA
FXX162010002088	2010	M	80	■■■■■■■■■■■■■■■■■■■■■■■■■■■■■■□■□□□□■■■■■■■	777777777720771	50	H3	-252---5-3-3	Orphan	252533336533343	Orphan	0		unknown	NA
FXX162010002278	2010	M	51	■■■■■■■■■■■■■■■■■■■■■■■■■■■■■■□■□□□□■■■■■■■	777777777720771	50	H3	-252---5-3-3	Orphan	252533336732543	Orphan	0		unknown	NA
FXX162008001541	2008	M	45	■■■■■■■■■■■■■■■■■■■■■■■■■■■■■■□■□□□□■■■■■■■	777777777720771	50	H3	-253---5-2-3	Orphan	253523335632343	Orphan	0		unknown	NA
FXX162009001869	2009	F	38	■■■■■■■■■■■■■■■■■■■■■■■■■■■■■■□■□□□□■■■■■■■	777777777720771	50	H3	-253---5-2-3	Orphan	253523336732343	Orphan	0		unknown	NA
FXX162009001890	2009	F	80	■■■■■■■■■■■■■■■■■■■■■■■■■■■■■■□■□□□□■■■■■■■	777777777720771	50	H3	-253---5-3-2	Orphan	253532335622243	355	0		unknown	NA
FXX162009001900	2009	F	88	■■■■■■■■■■■■■■■■■■■■■■■■■■■■■■□■□□□□■■■■■■■	777777777720771	50	H3	-253---5-3-2	Orphan	253532333722243	Orphan	0		unknown	NA
FXX162009001927	2009	M	75	■■■■■■■■■■■■■■■■■■■■■■■■■■■■■■□■□□□□■■■■■■■	777777777720771	50	H3	-253---5-3-2	Orphan	253532335522243	Orphan	0		unknown	NA
FXX162010001958	2010	M	65	■■■■■■■■■■■■■■■■■■■■■■■■■■■■■■□■□□□□■■■■■■■	777777777720771	50	H3	-253---5-3-2	Orphan	253532339732243	Orphan	0		unknown	NA
FXX162010002002	2010	F	25	■■■■■■■■■■■■■■■■■■■■■■■■■■■■■■□■□□□□■■■■■■■	777777777720771	50	H3	-253---5-3-2	Orphan	253532333622243	Orphan	0		unknown	NA
FXX162010002047	2010	M	47	■■■■■■■■■■■■■■■■■■■■■■■■■■■■■■□■□□□□■■■■■■■	777777777720771	50	H3	-253---5-3-2	Orphan	253532336532343	Orphan	0		unknown	NA
FXX162010002105	2010	F	39	■■■■■■■■■■■■■■■■■■■■■■■■■■■■■■□■□□□□■■■■■■■	777777777720771	50	H3	-253---5-3-2	Orphan	253532336522343	Orphan	0		unknown	NA
FXX162010002166	2010	M	49	■■■■■■■■■■■■■■■■■■■■■■■■■■■■■■□■□□□□■■■■■■■	777777777720771	50	H3	-253---5-3-2	Orphan	253532334622243	356	0		unknown	NA
FXX162010002174	2010	M	25	■■■■■■■■■■■■■■■■■■■■■■■■■■■■■■□■□□□□■■■■■■■	777777777720771	50	H3	-253---5-3-2	Orphan	253532333532343	354	0		unknown	NA
FXX162007001366	2007	M	39	■■■■■■■■■■■■■■■■■■■■■■■■■■■■■■□■□□□□■■■■■■■	777777777720771	50	H3	-253---5-3-3	Orphan	253533333722243	357	0		unknown	NA
FXX162008001382	2008	F	61	■■■■■■■■■■■■■■■■■■■■■■■■■■■■■■□■□□□□■■■■■■■	777777777720771	50	H3	-253---5-3-3	Orphan	253533333722243	357	0		unknown	NA
FXX162008001385	2008	M	47	■■■■■■■■■■■■■■■■■■■■■■■■■■■■■■□■□□□□■■■■■■■	777777777720771	50	H3	-253---5-3-3	Orphan	253533332342342	Orphan	0		unknown	NA
FXX162008001531	2008	M	61	■■■■■■■■■■■■■■■■■■■■■■■■■■■■■■□■□□□□■■■■■■■	777777777720771	50	H3	-253---5-3-3	Orphan	253533337633243	Orphan	0		unknown	NA
FXX162008001582	2008	M	86	■■■■■■■■■■■■■■■■■■■■■■■■■■■■■■□■□□□□■■■■■■■	777777777720771	50	H3	-253---5-3-3	Orphan	253533333732343	Orphan	0		unknown	NA
FXX162008001616	2008	M	43	■■■■■■■■■■■■■■■■■■■■■■■■■■■■■■□■□□□□■■■■■■■	777777777720771	50	H3	-253---5-3-3	Orphan	253533333342342	364	0		unknown	NA
FXX162008001645	2008	F	40	■■■■■■■■■■■■■■■■■■■■■■■■■■■■■■□■□□□□■■■■■■■	777777777720771	50	H3	-253---5-3-3	Orphan	2535333346-2343	Orphan	0		unknown	NA
FXX162008001688	2008	F	15	■■■■■■■■■■■■■■■■■■■■■■■■■■■■■■□■□□□□■■■■■■■	777777777720771	50	H3	-253---5-3-3	Orphan	253533335422243	366	0		unknown	NA
FXX162009001819	2009	M	4	■■■■■■■■■■■■■■■■■■■■■■■■■■■■■■□■□□□□■■■■■■■	777777777720771	50	H3	-253---5-3-3	Orphan	253533335731743	368	0		unknown	NA
FXX162009001820	2009	M	34	■■■■■■■■■■■■■■■■■■■■■■■■■■■■■■□■□□□□■■■■■■■	777777777720771	50	H3	-253---5-3-3	Orphan	253533335731743	368	0		unknown	NA
FXX162009001870	2009	F	40	■■■■■■■■■■■■■■■■■■■■■■■■■■■■■■□■□□□□■■■■■■■	777777777720771	50	H3	-253---5-3-3	Orphan	253533334732343	369	0		unknown	NA
FXX162009201968	2009	M	55	■■■■■■■■■■■■■■■■■■■■■■■■■■■■■■□■□□□□■■■■■■■	777777777720771	50	H3	-253---5-3-3	Orphan	253533337632343	371	2	SM, RIF, INH, ETH	unknown	NA
FXX162009001988	2009	M	35	■■■■■■■■■■■■■■■■■■■■■■■■■■■■■■□■□□□□■■■■■■■	777777777720771	50	H3	-253---5-3-3	Orphan	253533333532343	361	0		unknown	NA
FXX162009002073	2009	F	50	■■■■■■■■■■■■■■■■■■■■■■■■■■■■■■□■□□□□■■■■■■■	777777777720771	50	H3	-253---5-3-3	Orphan	253533333532343	361	0		unknown	NA
FXX162010002137	2010	M	45	■■■■■■■■■■■■■■■■■■■■■■■■■■■■■■□■□□□□■■■■■■■	777777777720771	50	H3	-253---5-3-3	Orphan	253533337632343	371	0		unknown	NA
FXX162010002341	2010	F	92	■■■■■■■■■■■■■■■■■■■■■■■■■■■■■■□■□□□□■■■■■■■	777777777720771	50	H3	-253---5-3-3	Orphan	253533333722243	357	0		unknown	NA
FXX162009001774	2009	F	38	■■■■■■■■■■■■■■■■■■■■■■■■■■■■■■□■□□□□■■■■■■■	777777777720771	50	H3	-253---5-3-4	Orphan	253534333732343	Orphan	0		unknown	NA
FXX162009001928	2009	M	74	■■■■■■■■■■■■■■■■■■■■■■■■■■■■■■□■□□□□■■■■■■■	777777777720771	50	H3	-253---6-3-3	Orphan	253633345732343	Orphan	0		unknown	NA
FXX162008001449	2008	M	49	■■■■■■■■■■■■■■■■■■■■■■■■■■■■■■□■□□□□■■■■■■■	777777777720771	50	H3	-253---5-3-3	Orphan	253533-36-32543	Orphan	0		unknown	NA
FXX162008001528	2008	F	86	■■■■■■■■■■■■■■■■■■■■■■■■■■■■■■□■□□□□■■■■■■■	777777777720771	50	H3	-263---4-3-3	Orphan	263433235722243	Orphan	0		unknown	NA
FXX162008001534	2008	M	79	■■■■■■■■■■■■■■■■■■■■■■■■■■■■■■□■□□□□■■■■■■■	777777777720771	50	H3	-263---5-3-3	Orphan	263533236722141	Orphan	0		unknown	NA
FXX162008001611	2008	M	77	■■■■■■■■■■■■■■■■■■■■■■■■■■■■■■□■□□□□■■■■■■■	777777777720771	50	H3	-263---5-3-3	Orphan	263533234722243	Orphan	0		unknown	NA
FXX162008001464	2008	M	20	■■■■■■■■■■■■■■■■■■■■■■■■■■■■■■□■□□□□■■■■■■■	777777777720771	50	H3	-263---5-3-3	Orphan	263533336632343	214	0		unknown	NA
FXX162010002086	2010	M	64	■■■■■■■■■■■■■■■■■■■■■■■■■■■■■■□■□□□□■■■■■■■	777777777720771	50	H3	-263---5-3-3	Orphan	263533335732343	Orphan	0		unknown	NA
FXX162009001978	2009	M	41	■■■■■■■■■■■■■■■■■■■■■■■■■■■■■■□■□□□□■■■■■■■	777777777720771	50	H3	-263---5-3-4	Orphan	263534335632242	Orphan	0		unknown	NA
FXX162009002040	2009	F	61	■■■■■■■■■■■■■■■■■■■■■■■■■■■■■■□■□□□□■■■■■■■	777777777720771	50	H3	-264---5-3-3	Orphan	264533333732343	Orphan	0		unknown	NA
FXX162009001872	2009	F	34	■■■■■■■■■■■■■■■■■■■■■■■■■■■■■■□■□□□□■■■■■■■	777777777720771	50	H3	-263---5-3-3	Orphan	263533x36632343	Orphan	0		unknown	NA
FXX162008001444	2008	F	71	■■■■■■■■■■■■■■■■■■■■■■■■■■■■■■□■□□□□■■■■■■■	777777777720771	50	H3	-2-----5-3-2	Orphan	2--532325-22243	Orphan	0		unknown	NA
FXX162009001826	2009	M	39	■■■■■■■■■■■■■■■■■■■■■■■■■■■■■■□■□□□□■■■■■■■	777777777720771	50	H3	-353---5-3-3	Orphan	353533334832343	Orphan	0		unknown	NA
FXX162008001467	2008	M	77	■■■■■■■■■■■■■■■■■■■■■■■■■■■■■■□■□□□□■■■■■■■	777777777720771	50	H3	--53---5-3-3	Orphan	-53533335722344	Orphan	0		unknown	NA

FXX162009001984	2009	F	23	■■■■■■■■■■■■■■■■■■■■■■■■■■■■■■□■□□□□■■■■■■■	777777777720771	50	H3					0		unknown	NA
FXX162005000660	2005	F	42	■■■■■■■■■■■■■■■■■■■■■■■■■■■■■■□■□□□□■■■■■■■	777777777720771	50	H3	125225153325	Orphan	252535---------	Orphan	0		France	P
FXX162006100778	2006	F	36	■■■■■■■■■■■■■■■■■■■■■■■■■■■■■■□■□□□□■■■■■■■	777777777720771	50	H3	126335153323	Orphan	263533336632343	214	1		Spain	P
FXX162006100793	2006	F	40	■■■■■■■■■■■■■■■■■■■■■■■■■■■■■■□■□□□□■■■■■■■	777777777720771	50	H3	224325143333	1577	243433---------	Orphan	1		France	P
FXX162007101209	2007	F	63	■■■■■■■■■■■■■■■■■■■■■■■■■■■■■■□■□□□□■■■■■■■	777777777720771	50	H3	225313153322	184	253532---------	Orphan	1		Algeria	P
FXX162005100546	2005	F	34	■■■■■■■■■■■■■■■■■■■■■■■■■■■■■■□■□□□□■■■■■■■	777777777720771	50	H3	225313153323	42	253533---------	Orphan	1		Morocco	P
FXX162006000680	2006	F	32	■■■■■■■■■■■■■■■■■■■■■■■■■■■■■■□■□□□□■■■■■■■	777777777720771	50	H3	225313153323	42	253533---------	Orphan	0		France	P
FXX162005100688	2005	M	21	■■■■■■■■■■■■■■■■■■■■■■■■■■■■■■□■□□□□■■■■■■■	777777777720771	50	H3	225313153323	42	253533---------	Orphan	1		Turkey	P
FXX162006000789	2006	M	71	■■■■■■■■■■■■■■■■■■■■■■■■■■■■■■□■□□□□■■■■■■■	777777777720771	50	H3	225313153323	42	253533---------	Orphan	0		France	P
FXX162006100982	2006	F	34	■■■■■■■■■■■■■■■■■■■■■■■■■■■■■■□■□□□□■■■■■■■	777777777720771	50	H3	225313153323	42	253533---------	Orphan	1		Algeria	P
FXX162006100993	2006	M	48	■■■■■■■■■■■■■■■■■■■■■■■■■■■■■■□■□□□□■■■■■■■	777777777720771	50	H3	225313153323	42	253533---------	Orphan	1		Algeria	P
FXX162005100554	2005	M	20	■■■■■■■■■■■■■■■■■■■■■■■■■■■■■■□■□□□□■■■■■■■	777777777720771	50	H3	225325153224	260	253524---------	Orphan	1		France	P
FXX162006100736	2006	M	36	■■■■■■■■■■■■■■■■■■■■■■■■■■■■■■□■□□□□■■■■■■■	777777777720771	50	H3	225325153321	742	253531---------	Orphan	1		Algeria	P
FXX162006100779	2006	M	56	■■■■■■■■■■■■■■■■■■■■■■■■■■■■■■□■□□□□■■■■■■■	777777777720771	50	H3	225325153323	45	253533---------	Orphan	1		France	P
FXX162005100549	2005	F	76	■■■■■■■■■■■■■■■■■■■■■■■■■■■■■■□■□□□□■■■■■■■	777777777720771	50	H3	225325153324	46	253534---------	Orphan	1		Germany	P
FXX162009101970	2009	M	80	■■■■■■■■■■■■■■■■■■■■■■■■■■■■■■□■□□□□■■■■■■■	777777777720771	50	H3	-234---5-3-2	Orphan	234532242522223	Orphan	1		unknown	P
FXX162009101839	2009	M	67	■■■■■■■■■■■■■■■■■■■■■■■■■■■■■■□■□□□□■■■■■■■	777777777720771	50	H3	-233---5-3-3	Orphan	233533347732343	Orphan	1		unknown	P
FXX162002100232	2002	M	43	■■■■■■■■■■■■■■■■■■■■■■■■■■■■■■□■□□□□■■■■■■■	777777777720771	50	H3	-243---5-3-3	Orphan	243533333212313	Orphan	1		France	P
FXX162008101659	2008	F	26	■■■■■■■■■■■■■■■■■■■■■■■■■■■■■■□■□□□□■■■■■■■	777777777720771	50	H3	-243---5-3-3	Orphan	243533335632342	Orphan	1		France	P
FXX162009101794	2009	M	80	■■■■■■■■■■■■■■■■■■■■■■■■■■■■■■□■□□□□■■■■■■■	777777777720771	50	H3	-253---5-3-3	Orphan	253533235723243	351	1		France	P
FXX162009101966	2009	M	31	■■■■■■■■■■■■■■■■■■■■■■■■■■■■■■□■□□□□■■■■■■■	777777777720771	50	H3	-253---3-3-2	Orphan	253332335622243	Orphan	1		unknown	P
FXX162008101561	2008	M	41	■■■■■■■■■■■■■■■■■■■■■■■■■■■■■■□■□□□□■■■■■■■	777777777720771	50	H3	-253---5-3-2	Orphan	253532332622244	Orphan	1		unknown	P
FXX162009101843	2009	F	27	■■■■■■■■■■■■■■■■■■■■■■■■■■■■■■□■□□□□■■■■■■■	777777777720771	50	H3	-253---5-3-2	Orphan	253532333532343	354	1		unknown	P
FXX162010301955	2010	M	50	■■■■■■■■■■■■■■■■■■■■■■■■■■■■■■□■□□□□■■■■■■■	777777777720771	50	H3	-253---5-3-2	Orphan	253532332522243	Orphan	3	SM	France	P
FXX162008101563	2008	M	69	■■■■■■■■■■■■■■■■■■■■■■■■■■■■■■□■□□□□■■■■■■■	777777777720771	50	H3	-253---5-3-3	Orphan	253533333342342	364	1		unknown	P
FXX162008301650	2008	M	59	■■■■■■■■■■■■■■■■■■■■■■■■■■■■■■□■□□□□■■■■■■■	777777777720771	50	H3	-253---5-3-3	Orphan	253533335532333	Orphan	3	SM	Romania	P
FXX162009101844	2009	M	38	■■■■■■■■■■■■■■■■■■■■■■■■■■■■■■□■□□□□■■■■■■■	777777777720771	50	H3	-253---5-3-3	Orphan	253533336722243	359	1		unknown	P
FXX162009102008	2009	M	82	■■■■■■■■■■■■■■■■■■■■■■■■■■■■■■□■□□□□■■■■■■■	777777777720771	50	H3	-253---5-3-3	Orphan	253533335722243	150	1		France	P
FXX162010102185	2010	M	58	■■■■■■■■■■■■■■■■■■■■■■■■■■■■■■□■□□□□■■■■■■■	777777777720771	50	H3	-253---5-3-3	Orphan	253533335422243	366	1		Senegal	P
FXX162001100048	2001	M	37	■■■■■■■■■■■■■■■■■■■■■■■■■■■■■■□■□□□□■■■■■■■	777777777720771	50	H3					1		France	P
FXX162001100095	2001	F	21	■■■■■■■■■■■■■■■■■■■■■■■■■■■■■■□■□□□□■■■■■■■	777777777720771	50	H3					1		France	P
FXX162001300105	2001	F	46	■■■■■■■■■■■■■■■■■■■■■■■■■■■■■■□■□□□□■■■■■■■	777777777720771	50	H3					3	SM	France	P
FXX162000300143	2000	M	42	■■■■■■■■■■■■■■■■■■■■■■■■■■■■■■□■□□□□■■■■■■■	777777777720771	50	H3					3	SM	Azerbaijan	P
FXX162000100160	2000	F	70	■■■■■■■■■■■■■■■■■■■■■■■■■■■■■■□■□□□□■■■■■■■	777777777720771	50	H3					1		France	P
FXX162002100196	2002	M	38	■■■■■■■■■■■■■■■■■■■■■■■■■■■■■■□■□□□□■■■■■■■	777777777720771	50	H3					1		Morocco	P
FXX162002100205	2002	M	43	■■■■■■■■■■■■■■■■■■■■■■■■■■■■■■□■□□□□■■■■■■■	777777777720771	50	H3					1		France	P
FXX162002100242	2002	M	49	■■■■■■■■■■■■■■■■■■■■■■■■■■■■■■□■□□□□■■■■■■■	777777777720771	50	H3					1		France	P
FXX162002100252	2002	F	99	■■■■■■■■■■■■■■■■■■■■■■■■■■■■■■□■□□□□■■■■■■■	777777777720771	50	H3					1		France	P
FXX162003100258	2003	M	35	■■■■■■■■■■■■■■■■■■■■■■■■■■■■■■□■□□□□■■■■■■■	777777777720771	50	H3					1		Tunisia	P
FXX162000100277	2000	F	29	■■■■■■■■■■■■■■■■■■■■■■■■■■■■■■□■□□□□■■■■■■■	777777777720771	50	H3					1		France	P
FXX162002100292	2002	M	53	■■■■■■■■■■■■■■■■■■■■■■■■■■■■■■□■□□□□■■■■■■■	777777777720771	50	H3					1		Tunisia	P
FXX162003100309	2003	F	72	■■■■■■■■■■■■■■■■■■■■■■■■■■■■■■□■□□□□■■■■■■■	777777777720771	50	H3					1		France	P
FXX162000000375	2000	F	45	■■■■■■■■■■■■■■■■■■■■■■■■■■■■■■□■□□□□■■■■■■■	777777777720771	50	H3					0		Algeria	P
FXX162000000378	2000	M	87	■■■■■■■■■■■■■■■■■■■■■■■■■■■■■■□■□□□□■■■■■■■	777777777720771	50	H3					0		France	P
FXX162000000380	2000	F	44	■■■■■■■■■■■■■■■■■■■■■■■■■■■■■■□■□□□□■■■■■■■	777777777720771	50	H3					0		France	P
FXX162000000386	2000	M	47	■■■■■■■■■■■■■■■■■■■■■■■■■■■■■■□■□□□□■■■■■■■	777777777720771	50	H3					0		France	P
FXX162003100422	2003	M	47	■■■■■■■■■■■■■■■■■■■■■■■■■■■■■■□■□□□□■■■■■■■	777777777720771	50	H3					1		Yugoslavia	P
FXX162004100445	2004	F	67	■■■■■■■■■■■■■■■■■■■■■■■■■■■■■■□■□□□□■■■■■■■	777777777720771	50	H3					1		France	P
FXX162004100502	2004	M	31	■■■■■■■■■■■■■■■■■■■■■■■■■■■■■■□■□□□□■■■■■■■	777777777720771	50	H3					1		France	P
FXX162004100517	2004	M	46	■■■■■■■■■■■■■■■■■■■■■■■■■■■■■■□■□□□□■■■■■■■	777777777720771	50	H3					1		France	P
FXX162007101068	2007	F	37	■■■■■■■■■■■■■■■■■■■■■■■■■■■■■■□■□□□□■■■■■■■	777777777720771	50	H3	224313153221	Orphan	243521---------	Orphan	1		unknown	P
FXX162007001167	2007	M	41	■■■■■■■■■■■■■■■■■■■■■■■■■■■■■■□■□□□□■■■■■■■	777777777720771	50	H3	225225153323	188	252533---------	Orphan	0		Hungary	P
FXX162007101201	2007	M	37	■■■■■■■■■■■■■■■■■■■■■■■■■■■■■■□■□□□□■■■■■■■	777777777720771	50	H3	225313153323	42	253533---------	Orphan	1		unknown	P
FXX162007101298	2007	F	33	■■■■■■■■■■■■■■■■■■■■■■■■■■■■■■□■□□□□■■■■■■■	777777777720771	50	H3	226313153423	Orphan	263543---------	Orphan	1		unknown	P
FXX162008101398	2008	M	22	■■■■■■■■■■■■■■■■■■■■■■■■■■■■■■□■□□□□■■■■■■■	777777777720771	50	H3	-223---5-3-3	Orphan	223533222722322	Orphan	1		unknown	P
FXX162008101553	2008	M	66	■■■■■■■■■■■■■■■■■■■■■■■■■■■■■■□■□□□□■■■■■■■	777777777720771	50	H3	-243---5-3-2	Orphan	243532336622243	Orphan	1		unknown	P
FXX162007001343	2007	M	40	■■■■■■■■■■■■■■■■■■■■■■■■■■■■■■□■□□□□■■■■■■■	777777777720771	50	H3	-252---5-3-3	Orphan	252533334643343	Orphan	0		unknown	P

FXX162008101371	2008	F	24	■■■■■■■■■■■■■■■■■■■■■■■■■■■■■■□■□□□□■■■■■■■	777777777720771	50	H3	-253---5-3-2	Orphan	253532333532343	354	1		Senegal	P
FXX162008101564	2008	F	33	■■■■■■■■■■■■■■■■■■■■■■■■■■■■■■□■□□□□■■■■■■■	777777777720771	50	H3	-253---5-3-2	Orphan	253532336622244	Orphan	1		unknown	P
FXX162007101121	2007	F	78	■■■■■■■■■■■■■■■■■■■■■■■■■■■■■■■■□□□□■■■□□□□	777777777760700	51	T1	223325153324	125	233534---------	Orphan	1		unknown	EP
FXX162008001455	2008	F	77	■■■■■■■■■■■■■■■■■■■■■■■■■■■■■■■■□□□□■■■□□□□	777777777760700	51	T1	-223---4-3-6	Orphan	223436343522224	Orphan	0		unknown	NA
FXX162009001830	2009	F	40	■■■■■■■■■■■■■■■■■■■■■■■■■■■■■■■■□□□□■■■□□□□	777777777760700	51	T1	-233---5-3-2	Orphan	233532245522223	Orphan	0		unknown	NA
FXX162009001903	2009	M	39	■■■■■■■■■■■■■■■■■■■■■■■■■■■■■■■■□□□□■■■□□□□	777777777760700	51	T1	-232---5-3-4	Orphan	232534334422223	Orphan	0		unknown	NA
FXX162008001709	2008	M	47	■■■■■■■■■■■■■■■■■■■■■■■■■■■■■■■■□□□□■■■□□□□	777777777760700	51	T1	-233---5-3-3	Orphan	233533343522223	322	0		unknown	NA
FXX162008001677	2008	F	90	■■■■■■■■■■■■■■■■■■■■■■■■■■■■■■■■□□□□■■■□□□□	777777777760700	51	T1	-243---5-3-4	Orphan	243534333532343	Orphan	0		unknown	NA
FXX162005100614	2005	M	85	■■■■■■■■■■■■■■■■■■■■■■■■■■■■■■■■□□□□■■■□□□□	777777777760700	51	T1	223325153324	125	233534---------	Orphan	1		unknown	P
FXX162002100228	2002	M	30	■■■■■■■■■■■■■■■■■■■■■■■■■■■■■■■■□□□□■■■□□□□	777777777760700	51	T1					1		Angola	P
FXX162000300281	2000	M	84	■■■■■■■■■■■■■■■■■■■■■■■■■■■■■■■■□□□□■■■□□□□	777777777760700	51	T1					3	SM	France	P
FXX162006300881	2006	F	60	■■■■■■■■■■■■■■■■■■■■■■■■■■■■■■■■□□□□■■■□■■■	777777777760731	52	T2	223325153224	13	233524---------	Orphan	3	PZA	unknown	EP
FXX162007101148	2007	M	38	■■■■■■■■■■■■■■■■■■■■■■■■■■■■■■■■□□□□■■■□■■■	777777777760731	52	T2	223325153423	230	233543---------	Orphan	1		Congo	EP
FXX162007101207	2007	F	24	■■■■■■■■■■■■■■■■■■■■■■■■■■■■■■■■□□□□■■■□■■■	777777777760731	52	T2	223325153423	230	233543---------	Orphan	1		Central African Republic	EP
FXX162008101552	2008	F	41	■■■■■■■■■■■■■■■■■■■■■■■■■■■■■■■■□□□□■■■□■■■	777777777760731	52	T2	-233---4-4-3	Orphan	233443363723323	Orphan	1		unknown	EP
FXX162000300149	2000	M	75	■■■■■■■■■■■■■■■■■■■■■■■■■■■■■■■■□□□□■■■□■■■	777777777760731	52	T2					3	SM	France	EP
FXX162005000645	2005	F	84	■■■■■■■■■■■■■■■■■■■■■■■■■■■■■■■■□□□□■■■□■■■	777777777760731	52	T2	222322153322	Orphan	223532---------	Orphan	0		unknown	NA
FXX162005000653	2005	M	75	■■■■■■■■■■■■■■■■■■■■■■■■■■■■■■■■□□□□■■■□■■■	777777777760731	52	T2	222327143323	Orphan	223433---------	Orphan	0		unknown	NA
FXX162007001140	2007	F	80	■■■■■■■■■■■■■■■■■■■■■■■■■■■■■■■■□□□□■■■□■■■	777777777760731	52	T2	222425163422	Orphan	224642---------	Orphan	0		unknown	NA
FXX162007001221	2007	M	80	■■■■■■■■■■■■■■■■■■■■■■■■■■■■■■■■□□□□■■■□■■■	777777777760731	52	T2	223225143322	783	232432---------	Orphan	0		unknown	NA
FXX162005000643	2005	M	82	■■■■■■■■■■■■■■■■■■■■■■■■■■■■■■■■□□□□■■■□■■■	777777777760731	52	T2	223325113322	1560	233132---------	Orphan	0		unknown	NA
FXX162006000894	2006	F	72	■■■■■■■■■■■■■■■■■■■■■■■■■■■■■■■■□□□□■■■□■■■	777777777760731	52	T2	223325153221	Orphan	233521---------	Orphan	0		unknown	NA
FXX162005000619	2005	M	34	■■■■■■■■■■■■■■■■■■■■■■■■■■■■■■■■□□□□■■■□■■■	777777777760731	52	T2	223325153224	13	233524---------	Orphan	0		unknown	NA
FXX162006000964	2006	M	83	■■■■■■■■■■■■■■■■■■■■■■■■■■■■■■■■□□□□■■■□■■■	777777777760731	52	T2	223325153322	15	233532---------	Orphan	0		unknown	NA
FXX162006001051	2006	M	83	■■■■■■■■■■■■■■■■■■■■■■■■■■■■■■■■□□□□■■■□■■■	777777777760731	52	T2	223326153321	224	233531---------	Orphan	0		unknown	NA
FXX162006000870	2006	M	82	■■■■■■■■■■■■■■■■■■■■■■■■■■■■■■■■□□□□■■■□■■■	777777777760731	52	T2	223425143322	160	234432---------	Orphan	0		unknown	NA
FXX162007001259	2007	M	30	■■■■■■■■■■■■■■■■■■■■■■■■■■■■■■■■□□□□■■■□■■■	777777777760731	52	T2	224325153325	35	243535---------	Orphan	0		unknown	NA
FXX162008001545	2008	M	63	■■■■■■■■■■■■■■■■■■■■■■■■■■■■■■■■□□□□■■■□■■■	777777777760731	52	T2	-233---5-3-4	Orphan	233534221621223	Orphan	0		unknown	NA
FXX162009001919	2009	F	48	■■■■■■■■■■■■■■■■■■■■■■■■■■■■■■■■□□□□■■■□■■■	777777777760731	52	T2	-233-----3-2	Orphan	233-32244522223	Orphan	0		unknown	NA
FXX162009001849	2009	F	89	■■■■■■■■■■■■■■■■■■■■■■■■■■■■■■■■□□□□■■■□■■■	777777777760731	52	T2	-234---3-3-2	Orphan	234332243422222	Orphan	0		unknown	NA
FXX162008001200	2008	F	85	■■■■■■■■■■■■■■■■■■■■■■■■■■■■■■■■□□□□■■■□■■■	777777777760731	52	T2	-234---4-3-2	Orphan	234432244522223	309	0		unknown	NA
FXX162007001365	2007	M	71	■■■■■■■■■■■■■■■■■■■■■■■■■■■■■■■■□□□□■■■□■■■	777777777760731	52	T2	-234---5-3-2	Orphan	234532244522223	311	0		unknown	NA
FXX162009001893	2009	F	82	■■■■■■■■■■■■■■■■■■■■■■■■■■■■■■■■□□□□■■■□■■■	777777777760731	52	T2	-234---5-3-2	Orphan	234532244522223	311	0		unknown	NA
FXX162009001906	2009	F	25	■■■■■■■■■■■■■■■■■■■■■■■■■■■■■■■■□□□□■■■□■■■	777777777760731	52	T2	-232---5-3-4	Orphan	232534344522223	Orphan	0		unknown	NA
FXX162008001699	2008	M	83	■■■■■■■■■■■■■■■■■■■■■■■■■■■■■■■■□□□□■■■□■■■	777777777760731	52	T2	-233---4-3-4	Orphan	233434342523225	Orphan	0		unknown	NA
FXX162008001408	2008	F	88	■■■■■■■■■■■■■■■■■■■■■■■■■■■■■■■■□□□□■■■□■■■	777777777760731	52	T2	-233---5-3-2	Orphan	233532344222223	Orphan	0		unknown	NA
FXX162008001511	2008	F	26	■■■■■■■■■■■■■■■■■■■■■■■■■■■■■■■■□□□□■■■□■■■	777777777760731	52	T2	-233---5-4-5	Orphan	233545343421221	Orphan	0		unknown	NA
FXX162009001865	2009	F	40	■■■■■■■■■■■■■■■■■■■■■■■■■■■■■■■■□□□□■■■□■■■	777777777760731	52	T2	-233---5-4-3	Orphan	233543442822323	Orphan	0		unknown	NA
FXX162009001895	2009	M	35	■■■■■■■■■■■■■■■■■■■■■■■■■■■■■■■■□□□□■■■□■■■	777777777760731	52	T2	-243---5-3-5	Orphan	243535344323223	Orphan	0		unknown	NA
FXX162008001614	2008	M	54	■■■■■■■■■■■■■■■■■■■■■■■■■■■■■■■■□□□□■■■□■■■	777777777760731	52	T2					0		unknown	NA
FXX162005100620	2005	F	80	■■■■■■■■■■■■■■■■■■■■■■■■■■■■■■■■□□□□■■■□■■■	777777777760731	52	T2	223225153322	296	232532---------	Orphan	1		France	P
FXX162005100608	2005	M	90	■■■■■■■■■■■■■■■■■■■■■■■■■■■■■■■■□□□□■■■□■■■	777777777760731	52	T2	223324153324	317	233534---------	Orphan	1		Algeria	P
FXX162005200557	2005	M	40	■■■■■■■■■■■■■■■■■■■■■■■■■■■■■■■■□□□□■■■□■■■	777777777760731	52	T2	223-25153423	Orphan	23-543---------	Orphan	2	INH, RIF	Rwanda	P
FXX162005100732	2005	M	74	■■■■■■■■■■■■■■■■■■■■■■■■■■■■■■■■□□□□■■■□■■■	777777777760731	52	T2	242325122322	303	423232---------	Orphan	1		Cambodia	P
FXX162009101812	2009	F	82	■■■■■■■■■■■■■■■■■■■■■■■■■■■■■■■■□□□□■■■□■■■	777777777760731	52	T2	-232---4-3-4	Orphan	232434322622223	Orphan	1		unknown	P
FXX162001300011	2001	M	19	■■■■■■■■■■■■■■■■■■■■■■■■■■■■■■■■□□□□■■■□■■■	777777777760731	52	T2					3	SM	Tunisia	P
FXX162001000067	2001	F	88	■■■■■■■■■■■■■■■■■■■■■■■■■■■■■■■■□□□□■■■□■■■	777777777760731	52	T2					0		France	P
FXX162000300158	2000	M	58	■■■■■■■■■■■■■■■■■■■■■■■■■■■■■■■■□□□□■■■□■■■	777777777760731	52	T2					3	SM	Algeria	P
FXX162003100217	2003	M	74	■■■■■■■■■■■■■■■■■■■■■■■■■■■■■■■■□□□□■■■□■■■	777777777760731	52	T2					1		Algeria	P
FXX162002300249	2002	M	27	■■■■■■■■■■■■■■■■■■■■■■■■■■■■■■■■□□□□■■■□■■■	777777777760731	52	T2					3	INH	Congo	P
FXX162003300322	2003	M	45	■■■■■■■■■■■■■■■■■■■■■■■■■■■■■■■■□□□□■■■□■■■	777777777760731	52	T2					3	SM, INH, ETH	Africa	P
FXX162000000395	2000	M	34	■■■■■■■■■■■■■■■■■■■■■■■■■■■■■■■■□□□□■■■□■■■	777777777760731	52	T2					0		Central African Republic	P
FXX162007101168	2007	M	34	■■■■■■■■■■■■■■■■■■■■■■■■■■■■■■■■□□□□■■■□■■■	777777777760731	52	T2	223325153224	13	233524---------	Orphan	1		Morocco	P
FXX162007101086	2007	M	87	■■■■■■■■■■■■■■■■■■■■■■■■■■■■■■■■□□□□■■■□■■■	777777777760731	52	T2	223425153221	586	234521---------	Orphan	1		France	P
FXX162005100550	2005	M	40	■■■■■■■■■■■■■■■■■■■■■■■■■■■■■■■■□□□□■■■■■■■	777777777760771	53	T1	222314031321	Orphan	223331---------	Orphan	1		France	EP
FXX162006100956	2006	F	46	■■■■■■■■■■■■■■■■■■■■■■■■■■■■■■■■□□□□■■■■■■■	777777777760771	53	T1	223225153324	169	232534---------	Orphan	1		Yugoslavia	EP
FXX162005100548	2005	F	74	■■■■■■■■■■■■■■■■■■■■■■■■■■■■■■■■□□□□■■■■■■■	777777777760771	53	T1	223325153322	15	233532---------	Orphan	1		Algeria	EP

FXX162000100262	2000	F	51	■■■■■■■■■■■■■■■■■■■■■■■■■■■■■■■■□□□□■■■■■■■	777777777760771	53	T1	223325153323	116	233533---------	Orphan	1		Maghreb	EP
FXX162005100601	2005	F	58	■■■■■■■■■■■■■■■■■■■■■■■■■■■■■■■■□□□□■■■■■■■	777777777760771	53	T1	223326151311	Orphan	233531---------	Orphan	1		Tunisia	EP
FXX162007101289	2007	M	96	■■■■■■■■■■■■■■■■■■■■■■■■■■■■■■■■□□□□■■■■■■■	777777777760771	53	T1	223415153322	1567	234532---------	Orphan	1		unknown	EP
FXX162006300792	2006	F	44	■■■■■■■■■■■■■■■■■■■■■■■■■■■■■■■■□□□□■■■■■■■	777777777760771	53	T1	225125113322	40	251132---------	Orphan	3	SM	Algeria	EP
FXX162009102005	2009	F	77	■■■■■■■■■■■■■■■■■■■■■■■■■■■■■■■■□□□□■■■■■■■	777777777760771	53	T1	-222---5-3-4	Orphan	222534336623223	Orphan	1		France	EP
FXX162009101962	2009	M	73	■■■■■■■■■■■■■■■■■■■■■■■■■■■■■■■■□□□□■■■■■■■	777777777760771	53	T1	-222---1-3-4	Orphan	222134343622223	Orphan	1		unknown	EP
FXX162008101663	2008	F	37	■■■■■■■■■■■■■■■■■■■■■■■■■■■■■■■■□□□□■■■■■■■	777777777760771	53	T1	-223---4-3-4	Orphan	223434346723223	Orphan	1		unknown	EP
FXX162008101627	2008	F	22	■■■■■■■■■■■■■■■■■■■■■■■■■■■■■■■■□□□□■■■■■■■	777777777760771	53	T1	-233---5-3-2	Orphan	233532244522222	Orphan	1		Angola	EP
FXX162009101749	2009	F	91	■■■■■■■■■■■■■■■■■■■■■■■■■■■■■■■■□□□□■■■■■■■	777777777760771	53	T1	-234---4-3-2	Orphan	234432245522223	310	1		France	EP
FXX162008001423	2008	M	64	■■■■■■■■■■■■■■■■■■■■■■■■■■■■■■■■□□□□■■■■■■■	777777777760771	53	T1	-234---5-2-2	Orphan	234522244523223	Orphan	0		unknown	EP
FXX162009101945	2009	F	80	■■■■■■■■■■■■■■■■■■■■■■■■■■■■■■■■□□□□■■■■■■■	777777777760771	53	T1	-231---5-3-5	Orphan	231535344522226	Orphan	1		unknown	EP
FXX162008101426	2008	F	61	■■■■■■■■■■■■■■■■■■■■■■■■■■■■■■■■□□□□■■■■■■■	777777777760771	53	T1	-233---4-3-4	Orphan	233434344523223	Orphan	1		Maghreb	EP
FXX162009101811	2009	F	88	■■■■■■■■■■■■■■■■■■■■■■■■■■■■■■■■□□□□■■■■■■■	777777777760771	53	T1	-233---5-3-3	Orphan	233533346522223	Orphan	1		France	EP
FXX162010102238	2010	M	33	■■■■■■■■■■■■■■■■■■■■■■■■■■■■■■■■□□□□■■■■■■■	777777777760771	53	T1	-233---5-3-3	Orphan	233533343522223	322	1		unknown	EP
FXX162010102121	2010	M	40	■■■■■■■■■■■■■■■■■■■■■■■■■■■■■■■■□□□□■■■■■■■	777777777760771	53	T1	-243---5-3-3	Orphan	243533333712343	263	1		unknown	EP
FXX162009101773	2009	F	28	■■■■■■■■■■■■■■■■■■■■■■■■■■■■■■■■□□□□■■■■■■■	777777777760771	53	T1	-264---6-4-3	Orphan	264643232732648	Orphan	1		unknown	EP
FXX162008101359	2008	F	62	■■■■■■■■■■■■■■■■■■■■■■■■■■■■■■■■□□□□■■■■■■■	777777777760771	53	T1	-283---6-4-3	Orphan	283643332632547	Orphan	1		unknown	EP
FXX162010102253	2010	M	61	■■■■■■■■■■■■■■■■■■■■■■■■■■■■■■■■□□□□■■■■■■■	777777777760771	53	T1	-283---6-4-3	Orphan	283643332632744	Orphan	1		unknown	EP
FXX162001100007	2001	M	23	■■■■■■■■■■■■■■■■■■■■■■■■■■■■■■■■□□□□■■■■■■■	777777777760771	53	T1					1		Algeria	EP
FXX162001300009	2001	M	1	■■■■■■■■■■■■■■■■■■■■■■■■■■■■■■■■□□□□■■■■■■■	777777777760771	53	T1					3	SM	Côte d'Ivoire	EP
FXX162001300036	2001	M	65	■■■■■■■■■■■■■■■■■■■■■■■■■■■■■■■■□□□□■■■■■■■	777777777760771	53	T1					3	SM	France	EP
FXX162001100069	2001	F	24	■■■■■■■■■■■■■■■■■■■■■■■■■■■■■■■■□□□□■■■■■■■	777777777760771	53	T1					1		Morocco	EP
FXX162001100098	2001	M	79	■■■■■■■■■■■■■■■■■■■■■■■■■■■■■■■■□□□□■■■■■■■	777777777760771	53	T1					1		France	EP
FXX162001000115	2001	M	67	■■■■■■■■■■■■■■■■■■■■■■■■■■■■■■■■□□□□■■■■■■■	777777777760771	53	T1					0		France	EP
FXX162003100137	2003	M	31	■■■■■■■■■■■■■■■■■■■■■■■■■■■■■■■■□□□□■■■■■■■	777777777760771	53	T1					1		Congo	EP
FXX162003100271	2003	M	39	■■■■■■■■■■■■■■■■■■■■■■■■■■■■■■■■□□□□■■■■■■■	777777777760771	53	T1					1		Algeria	EP
FXX162003100316	2003	F	53	■■■■■■■■■■■■■■■■■■■■■■■■■■■■■■■■□□□□■■■■■■■	777777777760771	53	T1					1		Algeria	EP
FXX162000000384	2000	M	73	■■■■■■■■■■■■■■■■■■■■■■■■■■■■■■■■□□□□■■■■■■■	777777777760771	53	T1					0		France	EP
FXX162003100432	2003	F	78	■■■■■■■■■■■■■■■■■■■■■■■■■■■■■■■■□□□□■■■■■■■	777777777760771	53	T1					1		France	EP
FXX162004100455	2004	M	81	■■■■■■■■■■■■■■■■■■■■■■■■■■■■■■■■□□□□■■■■■■■	777777777760771	53	T1					1		Poland	EP
FXX162004100466	2004	F	63	■■■■■■■■■■■■■■■■■■■■■■■■■■■■■■■■□□□□■■■■■■■	777777777760771	53	T1					1		Italy	EP
FXX162004100470	2004	F	81	■■■■■■■■■■■■■■■■■■■■■■■■■■■■■■■■□□□□■■■■■■■	777777777760771	53	T1					1		France	EP
FXX162005000696	2005	F	92	■■■■■■■■■■■■■■■■■■■■■■■■■■■■■■■■□□□□■■■■■■■	777777777760771	53	T1	123325153322	Orphan	233532---------	Orphan	0		unknown	NA
FXX162006000891	2006	F	39	■■■■■■■■■■■■■■■■■■■■■■■■■■■■■■■■□□□□■■■■■■■	777777777760771	53	T1	123326153323	Orphan	233533---------	Orphan	0		unknown	NA
FXX162005000640	2005	M	31	■■■■■■■■■■■■■■■■■■■■■■■■■■■■■■■■□□□□■■■■■■■	777777777760771	53	T1	215125113322	310	151132---------	Orphan	0		unknown	NA
FXX162007001276	2007	F	34	■■■■■■■■■■■■■■■■■■■■■■■■■■■■■■■■□□□□■■■■■■■	777777777760771	53	T1	215125113322	310	151132---------	Orphan	0		unknown	NA
FXX162007001288	2007	F	88	■■■■■■■■■■■■■■■■■■■■■■■■■■■■■■■■□□□□■■■■■■■	777777777760771	53	T1	222325153323	7	223533244623222	Orphan	0		unknown	NA
FXX162007001141	2007	M	91	■■■■■■■■■■■■■■■■■■■■■■■■■■■■■■■■□□□□■■■■■■■	777777777760771	53	T1	222325153323	7	223533341623222	Orphan	0		unknown	NA
FXX162007001236	2007	M	81	■■■■■■■■■■■■■■■■■■■■■■■■■■■■■■■■□□□□■■■■■■■	777777777760771	53	T1	222225143422	Orphan	222442---------	Orphan	0		unknown	NA
FXX162005000652	2005	F	89	■■■■■■■■■■■■■■■■■■■■■■■■■■■■■■■■□□□□■■■■■■■	777777777760771	53	T1	222325154322	Orphan	223532---------	Orphan	0		unknown	NA
FXX162005000708	2005	M	101	■■■■■■■■■■■■■■■■■■■■■■■■■■■■■■■■□□□□■■■■■■■	777777777760771	53	T1	222425143321	Orphan	224431---------	Orphan	0		unknown	NA
FXX162005000671	2005	F	60	■■■■■■■■■■■■■■■■■■■■■■■■■■■■■■■■□□□□■■■■■■■	777777777760771	53	T1	223325153323	116	233533343522224	Orphan	0		unknown	NA
FXX162005000728	2005	M	33	■■■■■■■■■■■■■■■■■■■■■■■■■■■■■■■■□□□□■■■■■■■	777777777760771	53	T1	223125143324	231	231434---------	Orphan	0		unknown	NA
FXX162006000973	2006	M	32	■■■■■■■■■■■■■■■■■■■■■■■■■■■■■■■■□□□□■■■■■■■	777777777760771	53	T1	223125153323	396	231533---------	Orphan	0		unknown	NA
FXX162005000650	2005	M	65	■■■■■■■■■■■■■■■■■■■■■■■■■■■■■■■■□□□□■■■■■■■	777777777760771	53	T1	223125153324	8	231534---------	Orphan	0		unknown	NA
FXX162007001075	2007	M	77	■■■■■■■■■■■■■■■■■■■■■■■■■■■■■■■■□□□□■■■■■■■	777777777760771	53	T1	223125153324	8	231534---------	Orphan	0		unknown	NA
FXX162007001076	2007	M	48	■■■■■■■■■■■■■■■■■■■■■■■■■■■■■■■■□□□□■■■■■■■	777777777760771	53	T1	223125153324	8	231534---------	Orphan	0		unknown	NA
FXX162007001256	2007	M	29	■■■■■■■■■■■■■■■■■■■■■■■■■■■■■■■■□□□□■■■■■■■	777777777760771	53	T1	223125153325	228	231535---------	Orphan	0		unknown	NA
FXX162007001270	2007	F	40	■■■■■■■■■■■■■■■■■■■■■■■■■■■■■■■■□□□□■■■■■■■	777777777760771	53	T1	223125153326	733	231536---------	Orphan	0		unknown	NA
FXX162006000896	2006	F	20	■■■■■■■■■■■■■■■■■■■■■■■■■■■■■■■■□□□□■■■■■■■	777777777760771	53	T1	223125154324	1035	231534---------	Orphan	0		unknown	NA
FXX162005000712	2005	M	62	■■■■■■■■■■■■■■■■■■■■■■■■■■■■■■■■□□□□■■■■■■■	777777777760771	53	T1	223225143321	333	232431---------	Orphan	0		unknown	NA
FXX162007001196	2007	M	28	■■■■■■■■■■■■■■■■■■■■■■■■■■■■■■■■□□□□■■■■■■■	777777777760771	53	T1	223225153322	296	232532---------	Orphan	0		unknown	NA
FXX162005000702	2005	F	76	■■■■■■■■■■■■■■■■■■■■■■■■■■■■■■■■□□□□■■■■■■■	777777777760771	53	T1	223225153324	169	232534---------	Orphan	0		unknown	NA
FXX162006000797	2006	F	57	■■■■■■■■■■■■■■■■■■■■■■■■■■■■■■■■□□□□■■■■■■■	777777777760771	53	T1	223225153324	169	232534---------	Orphan	0		unknown	NA
FXX162006000799	2006	M	56	■■■■■■■■■■■■■■■■■■■■■■■■■■■■■■■■□□□□■■■■■■■	777777777760771	53	T1	223225153324	169	232534---------	Orphan	0		unknown	NA
FXX162007001199	2007	M	76	■■■■■■■■■■■■■■■■■■■■■■■■■■■■■■■■□□□□■■■■■■■	777777777760771	53	T1	223225153324	169	232534---------	Orphan	0		unknown	NA
FXX162006000939	2006	M	53	■■■■■■■■■■■■■■■■■■■■■■■■■■■■■■■■□□□□■■■■■■■	777777777760771	53	T1	223226143424	1558	232444---------	Orphan	0		unknown	NA

FXX162006000942	2006	nsp	3	■■■■■■■■■■■■■■■■■■■■■■■■■■■■■■■■□□□□■■■■■■■	777777777760771	53	T1	223226143424	1558	232444---------	Orphan	0	unknown	NA
FXX162006000865	2006	F	35	■■■■■■■■■■■■■■■■■■■■■■■■■■■■■■■■□□□□■■■■■■■	777777777760771	53	T1	223322143322	1166	233432---------	Orphan	0	unknown	NA
FXX162006000875	2006	M	87	■■■■■■■■■■■■■■■■■■■■■■■■■■■■■■■■□□□□■■■■■■■	777777777760771	53	T1	223325143122	Orphan	233412---------	Orphan	0	unknown	NA
FXX162006001038	2006	M	72	■■■■■■■■■■■■■■■■■■■■■■■■■■■■■■■■□□□□■■■■■■■	777777777760771	53	T1	223325143321	796	233431---------	Orphan	0	unknown	NA
FXX162006000925	2006	F	38	■■■■■■■■■■■■■■■■■■■■■■■■■■■■■■■■□□□□■■■■■■■	777777777760771	53	T1	223325143323	121	233433---------	Orphan	0	unknown	NA
FXX162005000710	2005	M	83	■■■■■■■■■■■■■■■■■■■■■■■■■■■■■■■■□□□□■■■■■■■	777777777760771	53	T1	223325143325	118	233435---------	Orphan	0	unknown	NA
FXX162007001195	2007	M	54	■■■■■■■■■■■■■■■■■■■■■■■■■■■■■■■■□□□□■■■■■■■	777777777760771	53	T1	223325151324	Orphan	233534---------	Orphan	0	unknown	NA
FXX162007001158	2007	M	94	■■■■■■■■■■■■■■■■■■■■■■■■■■■■■■■■□□□□■■■■■■■	777777777760771	53	T1	223325153222	335	233522---------	Orphan	0	unknown	NA
FXX162005000675	2005	F	79	■■■■■■■■■■■■■■■■■■■■■■■■■■■■■■■■□□□□■■■■■■■	777777777760771	53	T1	223325153322	15	233532---------	Orphan	0	unknown	NA
FXX162006000805	2006	M	69	■■■■■■■■■■■■■■■■■■■■■■■■■■■■■■■■□□□□■■■■■■■	777777777760771	53	T1	223325153322	15	233532---------	Orphan	0	unknown	NA
FXX162006000846	2006	F	79	■■■■■■■■■■■■■■■■■■■■■■■■■■■■■■■■□□□□■■■■■■■	777777777760771	53	T1	223325153322	15	233532---------	Orphan	0	unknown	NA
FXX162006000866	2006	M	85	■■■■■■■■■■■■■■■■■■■■■■■■■■■■■■■■□□□□■■■■■■■	777777777760771	53	T1	223325153322	15	233532---------	Orphan	0	unknown	NA
FXX162006000930	2006	F	86	■■■■■■■■■■■■■■■■■■■■■■■■■■■■■■■■□□□□■■■■■■■	777777777760771	53	T1	223325153322	15	233532---------	Orphan	0	unknown	NA
FXX162006000940	2006	M	73	■■■■■■■■■■■■■■■■■■■■■■■■■■■■■■■■□□□□■■■■■■■	777777777760771	53	T1	223325153322	15	233532---------	Orphan	0	unknown	NA
FXX162006000967	2006	M	69	■■■■■■■■■■■■■■■■■■■■■■■■■■■■■■■■□□□□■■■■■■■	777777777760771	53	T1	223325153322	15	233532---------	Orphan	0	unknown	NA
FXX162006001003	2006	F	75	■■■■■■■■■■■■■■■■■■■■■■■■■■■■■■■■□□□□■■■■■■■	777777777760771	53	T1	223325153322	15	233532---------	Orphan	0	unknown	NA
FXX162006001005	2006	F	29	■■■■■■■■■■■■■■■■■■■■■■■■■■■■■■■■□□□□■■■■■■■	777777777760771	53	T1	223325153324	125	233534---------	Orphan	0	unknown	NA
FXX162006000890	2006	M	33	■■■■■■■■■■■■■■■■■■■■■■■■■■■■■■■■□□□□■■■■■■■	777777777760771	53	T1	223325154322	209	233532---------	Orphan	0	unknown	NA
FXX162006000966	2006	M	39	■■■■■■■■■■■■■■■■■■■■■■■■■■■■■■■■□□□□■■■■■■■	777777777760771	53	T1	223325154322	209	233532---------	Orphan	0	unknown	NA
FXX162006001006	2006	M	28	■■■■■■■■■■■■■■■■■■■■■■■■■■■■■■■■□□□□■■■■■■■	777777777760771	53	T1	223325154324	1562	233534---------	Orphan	0	unknown	NA
FXX162007001267	2007	F	57	■■■■■■■■■■■■■■■■■■■■■■■■■■■■■■■■□□□□■■■■■■■	777777777760771	53	T1	223326143324	1068	233434---------	Orphan	0	unknown	NA
FXX162005000704	2005	M	56	■■■■■■■■■■■■■■■■■■■■■■■■■■■■■■■■□□□□■■■■■■■	777777777760771	53	T1	223326143326	1564	233436---------	Orphan	0	unknown	NA
FXX162006000769	2006	M		■■■■■■■■■■■■■■■■■■■■■■■■■■■■■■■■□□□□■■■■■■■	777777777760771	53	T1	223326153223	1565	233523---------	Orphan	0	unknown	NA
FXX162007001090	2007	F	80	■■■■■■■■■■■■■■■■■■■■■■■■■■■■■■■■□□□□■■■■■■■	777777777760771	53	T1	223425143222	Orphan	234422---------	Orphan	0	unknown	NA
FXX162007001286	2007	M	83	■■■■■■■■■■■■■■■■■■■■■■■■■■■■■■■■□□□□■■■■■■■	777777777760771	53	T1	223425153222	189	234522---------	Orphan	0	unknown	NA
FXX162007001081	2007	F	80	■■■■■■■■■■■■■■■■■■■■■■■■■■■■■■■■□□□□■■■■■■■	777777777760771	53	T1	223425153322	157	234532---------	Orphan	0	unknown	NA
FXX162007001103	2007	M	79	■■■■■■■■■■■■■■■■■■■■■■■■■■■■■■■■□□□□■■■■■■■	777777777760771	53	T1	223425153322	157	234532---------	Orphan	0	unknown	NA
FXX162007001190	2007	M	67	■■■■■■■■■■■■■■■■■■■■■■■■■■■■■■■■□□□□■■■■■■■	777777777760771	53	T1	223425153322	157	234532---------	Orphan	0	unknown	NA
FXX162007001222	2007	M	41	■■■■■■■■■■■■■■■■■■■■■■■■■■■■■■■■□□□□■■■■■■■	777777777760771	53	T1	223425153323	382	234533---------	Orphan	0	unknown	NA
FXX162007001223	2007	F	79	■■■■■■■■■■■■■■■■■■■■■■■■■■■■■■■■□□□□■■■■■■■	777777777760771	53	T1	223525153312	Orphan	235532---------	Orphan	0	unknown	NA
FXX162006000850	2006	M	41	■■■■■■■■■■■■■■■■■■■■■■■■■■■■■■■■□□□□■■■■■■■	777777777760771	53	T1	224325153323	33	243533333712343	263	0	unknown	NA
FXX162007001284	2007	F	89	■■■■■■■■■■■■■■■■■■■■■■■■■■■■■■■■□□□□■■■■■■■	777777777760771	53	T1	224325153323	33	243533333612343	Orphan	0	unknown	NA
FXX162007001257	2007	F	32	■■■■■■■■■■■■■■■■■■■■■■■■■■■■■■■■□□□□■■■■■■■	777777777760771	53	T1	2243251-3323	Orphan	243-33333712343	Orphan	0	unknown	NA
FXX162005000722	2005	M	61	■■■■■■■■■■■■■■■■■■■■■■■■■■■■■■■■□□□□■■■■■■■	777777777760771	53	T1	224225153326	1573	242536---------	Orphan	0	unknown	NA
FXX162006000857	2006	M	63	■■■■■■■■■■■■■■■■■■■■■■■■■■■■■■■■□□□□■■■■■■■	777777777760771	53	T1	224315153322	648	243532---------	Orphan	0	unknown	NA
FXX162007001274	2007	M	60	■■■■■■■■■■■■■■■■■■■■■■■■■■■■■■■■□□□□■■■■■■■	777777777760771	53	T1	225125113322	40	251132---------	Orphan	0	unknown	NA
FXX162007001264	2007	M	87	■■■■■■■■■■■■■■■■■■■■■■■■■■■■■■■■□□□□■■■■■■■	777777777760771	53	T1	226325163423	1106	263643---------	Orphan	0	unknown	NA
FXX162007001275	2007	M	45	■■■■■■■■■■■■■■■■■■■■■■■■■■■■■■■■□□□□■■■■■■■	777777777760771	53	T1	227325163423	802	273643---------	Orphan	0	unknown	NA
FXX162006000902	2006	F	23	■■■■■■■■■■■■■■■■■■■■■■■■■■■■■■■■□□□□■■■■■■■	777777777760771	53	T1	228325163423	415	283643---------	Orphan	0	unknown	NA
FXX162007001089	2007	F	84	■■■■■■■■■■■■■■■■■■■■■■■■■■■■■■■■□□□□■■■■■■■	777777777760771	53	T1	228325163423	415	283643---------	Orphan	0	unknown	NA
FXX162009001916	2009	M	77	■■■■■■■■■■■■■■■■■■■■■■■■■■■■■■■■□□□□■■■■■■■	777777777760771	53	T1	-223---5-3-6	Orphan	223536245623223	Orphan	0	unknown	NA
FXX162009002074	2009	F	49	■■■■■■■■■■■■■■■■■■■■■■■■■■■■■■■■□□□□■■■■■■■	777777777760771	53	T1	-221---5-3-4	Orphan	221534343322224	Orphan	0	unknown	NA
FXX162010002102	2010	F	36	■■■■■■■■■■■■■■■■■■■■■■■■■■■■■■■■□□□□■■■■■■■	777777777760771	53	T1	-223---4-2-2	Orphan	223422344623223	Orphan	0	unknown	NA
FXX162009001784	2009	F	78	■■■■■■■■■■■■■■■■■■■■■■■■■■■■■■■■□□□□■■■■■■■	777777777760771	53	T1	-223---4-3-3	Orphan	223433344623222	Orphan	0	unknown	NA
FXX162010002288	2010	F	inconnu	■■■■■■■■■■■■■■■■■■■■■■■■■■■■■■■■□□□□■■■■■■■	777777777760771	53	T1	-223---5-2-3	Orphan	223523345723222	Orphan	0	unknown	NA
FXX162009002025	2009	M	76	■■■■■■■■■■■■■■■■■■■■■■■■■■■■■■■■□□□□■■■■■■■	777777777760771	53	T1	-223---5-3-2	Orphan	223532346723223	Orphan	0	unknown	NA
FXX162008001407	2008	M	83	■■■■■■■■■■■■■■■■■■■■■■■■■■■■■■■■□□□□■■■■■■■	777777777760771	53	T1	-223---5-3-3	Orphan	223533345623222	Orphan	0	unknown	NA
FXX162008001417	2008	M	84	■■■■■■■■■■■■■■■■■■■■■■■■■■■■■■■■□□□□■■■■■■■	777777777760771	53	T1	-223---6-3-5	Orphan	223635345522223	Orphan	0	unknown	NA
FXX162010002334	2010	F	87	■■■■■■■■■■■■■■■■■■■■■■■■■■■■■■■■□□□□■■■■■■■	777777777760771	53	T1	-223---5-3-3	Orphan	223533355923223	Orphan	0	unknown	NA
FXX162010002129	2010	F	89	■■■■■■■■■■■■■■■■■■■■■■■■■■■■■■■■□□□□■■■■■■■	777777777760771	53	T1	-234---4-3-2	Orphan	234432131522322	Orphan	0	unknown	NA
FXX162008001568	2008	F	39	■■■■■■■■■■■■■■■■■■■■■■■■■■■■■■■■□□□□■■■■■■■	777777777760771	53	T1	-231---5-2-4	Orphan	231524242422225	Orphan	0	unknown	NA
FXX162009002070	2009	F	74	■■■■■■■■■■■■■■■■■■■■■■■■■■■■■■■■□□□□■■■■■■■	777777777760771	53	T1	-232---5-3-2	Orphan	232532245421223	Orphan	0	unknown	NA
FXX162010002237	2010	F	78	■■■■■■■■■■■■■■■■■■■■■■■■■■■■■■■■□□□□■■■■■■■	777777777760771	53	T1	-232---5-3-3	Orphan	232533245422223	Orphan	0	unknown	NA
FXX162009001854	2009	F	82	■■■■■■■■■■■■■■■■■■■■■■■■■■■■■■■■□□□□■■■■■■■	777777777760771	53	T1	-233---5-3-2	Orphan	233532234522223	303	0	unknown	NA
FXX162009001860	2009	F	91	■■■■■■■■■■■■■■■■■■■■■■■■■■■■■■■■□□□□■■■■■■■	777777777760771	53	T1	-233---5-3-2	Orphan	233532234522223	303	0	unknown	NA
FXX162009001915	2009	F	67	■■■■■■■■■■■■■■■■■■■■■■■■■■■■■■■■□□□□■■■■■■■	777777777760771	53	T1	-233---3-2-2	Orphan	233322245322222	Orphan	0	unknown	NA
FXX162010002158	2010	M	82	■■■■■■■■■■■■■■■■■■■■■■■■■■■■■■■■□□□□■■■■■■■	777777777760771	53	T1	-233---4-3-2	Orphan	233432243522223	Orphan	0	unknown	NA

FXX162009001779	2009	F	79	■■■■■■■■■■■■■■■■■■■■■■■■■■■■■■■■□□□□■■■■■■■	777777777760771	53	T1	-233---5-2-2	Orphan	233522243322223	Orphan	0	unknown	NA
FXX162008001539	2008	F	71	■■■■■■■■■■■■■■■■■■■■■■■■■■■■■■■■□□□□■■■■■■■	777777777760771	53	T1	-233---5-3-2	Orphan	233532243522223	Orphan	0	unknown	NA
FXX162009001875	2009	F	85	■■■■■■■■■■■■■■■■■■■■■■■■■■■■■■■■□□□□■■■■■■■	777777777760771	53	T1	-233---5-3-2	Orphan	233532245523223	Orphan	0	unknown	NA
FXX162010002103	2010	F	85	■■■■■■■■■■■■■■■■■■■■■■■■■■■■■■■■□□□□■■■■■■■	777777777760771	53	T1	-233---5-3-2	Orphan	23353224-503224	Orphan	0	unknown	NA
FXX162008001482	2008	F	88	■■■■■■■■■■■■■■■■■■■■■■■■■■■■■■■■□□□□■■■■■■■	777777777760771	53	T1	-233---5-3-3	Orphan	233533245623223	Orphan	0	unknown	NA
FXX162009001985	2009	F	47	■■■■■■■■■■■■■■■■■■■■■■■■■■■■■■■■□□□□■■■■■■■	777777777760771	53	T1	-233---5-3-4	Orphan	233534242422225	Orphan	0	unknown	NA
FXX162008001639	2008	M	86	■■■■■■■■■■■■■■■■■■■■■■■■■■■■■■■■□□□□■■■■■■■	777777777760771	53	T1	-234---5-3-2	Orphan	234532224521223	Orphan	0	unknown	NA
FXX162008001454	2008	M	54	■■■■■■■■■■■■■■■■■■■■■■■■■■■■■■■■□□□□■■■■■■■	777777777760771	53	T1	-234---4-3-2	Orphan	234432233421223	Orphan	0	unknown	NA
FXX162009001912	2009	M	72	■■■■■■■■■■■■■■■■■■■■■■■■■■■■■■■■□□□□■■■■■■■	777777777760771	53	T1	-234---4-3-2	Orphan	234432233521223	Orphan	0	unknown	NA
FXX162008101433	2008	F	93	■■■■■■■■■■■■■■■■■■■■■■■■■■■■■■■■□□□□■■■■■■■	777777777760771	53	T1	-234---5-3-2	Orphan	234532233522223	306	1	unknown	NA
FXX162010002228	2010	M	48	■■■■■■■■■■■■■■■■■■■■■■■■■■■■■■■■□□□□■■■■■■■	777777777760771	53	T1	-234---5-3-2	Orphan	234532234524223	Orphan	0	unknown	NA
FXX162010002236	2010	F	27	■■■■■■■■■■■■■■■■■■■■■■■■■■■■■■■■□□□□■■■■■■■	777777777760771	53	T1	-234---5-3-2	Orphan	234532233522223	306	0	unknown	NA
FXX162010002345	2010	F	76	■■■■■■■■■■■■■■■■■■■■■■■■■■■■■■■■□□□□■■■■■■■	777777777760771	53	T1	-234---5-3-2	Orphan	234532233522223	306	0	unknown	NA
FXX162008001589	2008	M	88	■■■■■■■■■■■■■■■■■■■■■■■■■■■■■■■■□□□□■■■■■■■	777777777760771	53	T1	-234---3-3-2	Orphan	234332243522223	Orphan	0	unknown	NA
FXX162009001965	2009	M	74	■■■■■■■■■■■■■■■■■■■■■■■■■■■■■■■■□□□□■■■■■■■	777777777760771	53	T1	-234---3-3-2	Orphan	234332245522223	308	0	unknown	NA
FXX162008001415	2008	M	84	■■■■■■■■■■■■■■■■■■■■■■■■■■■■■■■■□□□□■■■■■■■	777777777760771	53	T1	-234---4-3-1	Orphan	234431244522223	Orphan	0	unknown	NA
FXX162010002202	2010	F	29	■■■■■■■■■■■■■■■■■■■■■■■■■■■■■■■■□□□□■■■■■■■	777777777760771	53	T1	-234---4-3-2	Orphan	234432243522223	Orphan	0	France	NA
FXX162010002220	2010	F	81	■■■■■■■■■■■■■■■■■■■■■■■■■■■■■■■■□□□□■■■■■■■	777777777760771	53	T1	-234---4-3-2	Orphan	234432244522223	309	0	unknown	NA
FXX162010002309	2010	F	58	■■■■■■■■■■■■■■■■■■■■■■■■■■■■■■■■□□□□■■■■■■■	777777777760771	53	T1	-234---4-3-2	Orphan	234432244522223	309	0	unknown	NA
FXX162008001507	2008	F	69	■■■■■■■■■■■■■■■■■■■■■■■■■■■■■■■■□□□□■■■■■■■	777777777760771	53	T1	-234---5-2-2	Orphan	234522242522223	Orphan	0	unknown	NA
FXX162010002293	2010	M	81	■■■■■■■■■■■■■■■■■■■■■■■■■■■■■■■■□□□□■■■■■■■	777777777760771	53	T1	-234---5-2-2	Orphan	234522244422223	Orphan	0	unknown	NA
FXX162009001787	2009	M	40	■■■■■■■■■■■■■■■■■■■■■■■■■■■■■■■■□□□□■■■■■■■	777777777760771	53	T1	-234---5-3-2	Orphan	234532243522223	Orphan	0	unknown	NA
FXX162009001851	2009	F	97	■■■■■■■■■■■■■■■■■■■■■■■■■■■■■■■■□□□□■■■■■■■	777777777760771	53	T1	-234---5-3-2	Orphan	234532242523223	Orphan	0	unknown	NA
FXX162010002298	2010	F	91	■■■■■■■■■■■■■■■■■■■■■■■■■■■■■■■■□□□□■■■■■■■	777777777760771	53	T1	-234---5-3-2	Orphan	234532245522223	313	0	unknown	NA
FXX162010002300	2010	F	96	■■■■■■■■■■■■■■■■■■■■■■■■■■■■■■■■□□□□■■■■■■■	777777777760771	53	T1	-234---5-3-2	Orphan	234532244522223	311	0	unknown	NA
FXX162010002305	2010	M	74	■■■■■■■■■■■■■■■■■■■■■■■■■■■■■■■■□□□□■■■■■■■	777777777760771	53	T1	-234---5-3-2	Orphan	234532245422223	Orphan	0	unknown	NA
FXX162008001383	2008	M	58	■■■■■■■■■■■■■■■■■■■■■■■■■■■■■■■■□□□□■■■■■■■	777777777760771	53	T1	-234---5-3-3	Orphan	234533245523223	314	0	unknown	NA
FXX162010002156	2010	M	31	■■■■■■■■■■■■■■■■■■■■■■■■■■■■■■■■□□□□■■■■■■■	777777777760771	53	T1	-234---5-3-3	Orphan	234533245523223	314	0	unknown	NA
FXX162010002222	2010	F	15	■■■■■■■■■■■■■■■■■■■■■■■■■■■■■■■■□□□□■■■■■■■	777777777760771	53	T1	-234---5-3-3	Orphan	234533245523223	314	0	unknown	NA
FXX162010002330	2010	F	81	■■■■■■■■■■■■■■■■■■■■■■■■■■■■■■■■□□□□■■■■■■■	777777777760771	53	T1	-234---5-3-2	Orphan	234532254322223	Orphan	0	unknown	NA
FXX162010002299	2010	M	77	■■■■■■■■■■■■■■■■■■■■■■■■■■■■■■■■□□□□■■■■■■■	777777777760771	53	T1	-235---6-3-2	Orphan	235632242532223	Orphan	0	unknown	NA
FXX162008001409	2008	F	83	■■■■■■■■■■■■■■■■■■■■■■■■■■■■■■■■□□□□■■■■■■■	777777777760771	53	T1	-23----5-3-2	Orphan	23-532221522223	Orphan	0	unknown	NA
FXX162008001571	2008	M	52	■■■■■■■■■■■■■■■■■■■■■■■■■■■■■■■■□□□□■■■■■■■	777777777760771	53	T1	-231---4-3-4	Orphan	231434323522225	Orphan	0	unknown	NA
FXX162008001447	2008	M	28	■■■■■■■■■■■■■■■■■■■■■■■■■■■■■■■■□□□□■■■■■■■	777777777760771	53	T1	-231---4-3-5	Orphan	231435324522223	Orphan	0	unknown	NA
FXX162008001646	2008	F	58	■■■■■■■■■■■■■■■■■■■■■■■■■■■■■■■■□□□□■■■■■■■	777777777760771	53	T1	-231---4-3-4	Orphan	231434344722224	Orphan	0	unknown	NA
FXX162010002138	2010	F	21	■■■■■■■■■■■■■■■■■■■■■■■■■■■■■■■■□□□□■■■■■■■	777777777760771	53	T1	-231---4-3-4	Orphan	231434341522225	Orphan	0	unknown	NA
FXX162008001706	2008	M	41	■■■■■■■■■■■■■■■■■■■■■■■■■■■■■■■■□□□□■■■■■■■	777777777760771	53	T1	-231---5-2-4	Orphan	231524343522225	Orphan	0	unknown	NA
FXX162008001416	2008	M	48	■■■■■■■■■■■■■■■■■■■■■■■■■■■■■■■■□□□□■■■■■■■	777777777760771	53	T1	-231---5-3-2	Orphan	231532344522223	Orphan	0	unknown	NA
FXX162009002026	2009	F	78	■■■■■■■■■■■■■■■■■■■■■■■■■■■■■■■■□□□□■■■■■■■	777777777760771	53	T1	-231---5-3-4	Orphan	231534343322224	Orphan	0	unknown	NA
FXX162009002039	2009	M	91	■■■■■■■■■■■■■■■■■■■■■■■■■■■■■■■■□□□□■■■■■■■	777777777760771	53	T1	-231---5-3-4	Orphan	231534343422223	316	0	unknown	NA
FXX162009001885	2009	M	42	■■■■■■■■■■■■■■■■■■■■■■■■■■■■■■■■□□□□■■■■■■■	777777777760771	53	T1	-231---5-3-4	Orphan	231534354532225	Orphan	0	unknown	NA
FXX162008001530	2008	F	34	■■■■■■■■■■■■■■■■■■■■■■■■■■■■■■■■□□□□■■■■■■■	777777777760771	53	T1	-232---4-4-4	Orphan	232444333422223	Orphan	0	unknown	NA
FXX162010001954	2010	M	35	■■■■■■■■■■■■■■■■■■■■■■■■■■■■■■■■□□□□■■■■■■■	777777777760771	53	T1	-232---5-3-2	Orphan	232532334422223	Orphan	0	unknown	NA
FXX162010002335	2010	M	72	■■■■■■■■■■■■■■■■■■■■■■■■■■■■■■■■□□□□■■■■■■■	777777777760771	53	T1	-232---7-3-1	Orphan	232731343522327	Orphan	0	unknown	NA
FXX162008001689	2008	M	72	■■■■■■■■■■■■■■■■■■■■■■■■■■■■■■■■□□□□■■■■■■■	777777777760771	53	T1	-233---3-3-5	Orphan	233335346623223	Orphan	0	unknown	NA
FXX162010002106	2010	F	38	■■■■■■■■■■■■■■■■■■■■■■■■■■■■■■■■□□□□■■■■■■■	777777777760771	53	T1	-233---4-3-2	Orphan	233432343502223	Orphan	0	unknown	NA
FXX162009001877	2009	F	87	■■■■■■■■■■■■■■■■■■■■■■■■■■■■■■■■□□□□■■■■■■■	777777777760771	53	T1	-233---5-3-1	Orphan	233531343522227	Orphan	0	unknown	NA
FXX162009002034	2009	M	40	■■■■■■■■■■■■■■■■■■■■■■■■■■■■■■■■□□□□■■■■■■■	777777777760771	53	T1	-233---5-3-1	Orphan	233531344422224	320	0	unknown	NA
FXX162007001352	2007	F	51	■■■■■■■■■■■■■■■■■■■■■■■■■■■■■■■■□□□□■■■■■■■	777777777760771	53	T1	-233---5-3-2	Orphan	233532344522223	Orphan	0	unknown	NA
FXX162009001898	2009	F	88	■■■■■■■■■■■■■■■■■■■■■■■■■■■■■■■■□□□□■■■■■■■	777777777760771	53	T1	-233---5-3-2	Orphan	233532343321225	Orphan	0	unknown	NA
FXX162007001361	2007	F	78	■■■■■■■■■■■■■■■■■■■■■■■■■■■■■■■■□□□□■■■■■■■	777777777760771	53	T1	-233---5-3-4	Orphan	233534345523223	324	0	unknown	NA
FXX162010002050	2010	M	30	■■■■■■■■■■■■■■■■■■■■■■■■■■■■■■■■□□□□■■■■■■■	777777777760771	53	T1	-233---5-4-4	Orphan	233544343222223	Orphan	0	unknown	NA
FXX162008001633	2008	F	70	■■■■■■■■■■■■■■■■■■■■■■■■■■■■■■■■□□□□■■■■■■■	777777777760771	53	T1	-233---7-2-4	Orphan	233724344322223	Orphan	0	unknown	NA
FXX162009001930	2009	F	68	■■■■■■■■■■■■■■■■■■■■■■■■■■■■■■■■□□□□■■■■■■■	777777777760771	53	T1	-233---4-3-4	Orphan	233434366522223	Orphan	0	unknown	NA
FXX162008001452	2008	F	93	■■■■■■■■■■■■■■■■■■■■■■■■■■■■■■■■□□□□■■■■■■■	777777777760771	53	T1	-234---4-2-2	Orphan	234422-4-521223	Orphan	0	unknown	NA
FXX162008001465	2008	M	26	■■■■■■■■■■■■■■■■■■■■■■■■■■■■■■■■□□□□■■■■■■■	777777777760771	53	T1	-243---5-3-3	Orphan	243533333712343	263	0	unknown	NA
FXX162008001567	2008	M	31	■■■■■■■■■■■■■■■■■■■■■■■■■■■■■■■■□□□□■■■■■■■	777777777760771	53	T1	-243---5-3-3	Orphan	243533333712343	263	0	unknown	NA

FXX162008001696	2008	F	73	■■■■■■■■■■■■■■■■■■■■■■■■■■■■■■■■□□□□■■■■■■■	777777777760771	53	T1	-243---5-3-3	Orphan	243533333712343	263	0		unknown	NA
FXX162010002304	2010	M	63	■■■■■■■■■■■■■■■■■■■■■■■■■■■■■■■■□□□□■■■■■■■	777777777760771	53	T1	-243---5-3-3	Orphan	243533333812343	Orphan	0		unknown	NA
FXX162010002176	2010	M	26	■■■■■■■■■■■■■■■■■■■■■■■■■■■■■■■■□□□□■■■■■■■	777777777760771	53	T1	-243---6-3-5	Orphan	243635333434343	Orphan	0		unknown	NA
FXX162009002075	2009	M	55	■■■■■■■■■■■■■■■■■■■■■■■■■■■■■■■■□□□□■■■■■■■	777777777760771	53	T1	-251---1-1-2	Orphan	251112322931443	Orphan	0		unknown	NA
FXX162009001995	2009	M	37	■■■■■■■■■■■■■■■■■■■■■■■■■■■■■■■■□□□□■■■■■■■	777777777760771	53	T1	-283---6-4-3	Orphan	283643232632742	Orphan	0		unknown	NA
FXX162007001347	2007	M	41	■■■■■■■■■■■■■■■■■■■■■■■■■■■■■■■■□□□□■■■■■■■	777777777760771	53	T1	-283---6-4-2	Orphan	283642332632648	379	0		unknown	NA
FXX162009001862	2009	M	21	■■■■■■■■■■■■■■■■■■■■■■■■■■■■■■■■□□□□■■■■■■■	777777777760771	53	T1	-283---6-4-2	Orphan	283642332632648	379	0		unknown	NA
FXX162008001316	2008	F	81	■■■■■■■■■■■■■■■■■■■■■■■■■■■■■■■■□□□□■■■■■■■	777777777760771	53	T1	-283---6-4-3	Orphan	283643332632648	380	0		unknown	NA
FXX162008001483	2008	M	64	■■■■■■■■■■■■■■■■■■■■■■■■■■■■■■■■□□□□■■■■■■■	777777777760771	53	T1	-283---6-4-3	Orphan	283643332632648	380	0		unknown	NA
FXX162008001579	2008	M	38	■■■■■■■■■■■■■■■■■■■■■■■■■■■■■■■■□□□□■■■■■■■	777777777760771	53	T1	-283---6-4-3	Orphan	283643332632648	380	0		unknown	NA
FXX162008001606	2008	M	63	■■■■■■■■■■■■■■■■■■■■■■■■■■■■■■■■□□□□■■■■■■■	777777777760771	53	T1	-283---6-4-3	Orphan	283643332632648	380	0		unknown	NA
FXX162008001640	2008	M	49	■■■■■■■■■■■■■■■■■■■■■■■■■■■■■■■■□□□□■■■■■■■	777777777760771	53	T1	-283---6-4-3	Orphan	283643332632648	380	0		unknown	NA
FXX162009001835	2009	M	67	■■■■■■■■■■■■■■■■■■■■■■■■■■■■■■■■□□□□■■■■■■■	777777777760771	53	T1	-283---6-4-3	Orphan	283643332732747	Orphan	0		unknown	NA
FXX162009001858	2009	M	47	■■■■■■■■■■■■■■■■■■■■■■■■■■■■■■■■□□□□■■■■■■■	777777777760771	53	T1	-283---6-4-3	Orphan	283643332632648	380	0		unknown	NA
FXX162010002162	2010	M	63	■■■■■■■■■■■■■■■■■■■■■■■■■■■■■■■■□□□□■■■■■■■	777777777760771	53	T1	-283---6-4-3	Orphan	283643332632647	Orphan	0		unknown	NA
FXX162010002308	2010	M	65	■■■■■■■■■■■■■■■■■■■■■■■■■■■■■■■■□□□□■■■■■■■	777777777760771	53	T1	-283---6-4-3	Orphan	283643332632646	Orphan	0		unknown	NA
FXX162009001979	2009	M	36	■■■■■■■■■■■■■■■■■■■■■■■■■■■■■■■■□□□□■■■■■■■	777777777760771	53	T1	-28----6-4-3	Orphan	28-643332632648	Orphan	0		unknown	NA
FXX162010002082	2010	F	38	■■■■■■■■■■■■■■■■■■■■■■■■■■■■■■■■□□□□■■■■■■■	777777777760771	53	T1	-293---6-5-3	Orphan	293653332632747	Orphan	0		unknown	NA
FXX162010002150	2010	M	85	■■■■■■■■■■■■■■■■■■■■■■■■■■■■■■■■□□□□■■■■■■■	777777777760771	53	T1	-334---5-3-2	Orphan	334532243522223	388	0		unknown	NA
FXX162010002234	2010	F	82	■■■■■■■■■■■■■■■■■■■■■■■■■■■■■■■■□□□□■■■■■■■	777777777760771	53	T1	-334---5-3-2	Orphan	334532243522223	388	0		unknown	NA
FXX162009002038	2009	M	80	■■■■■■■■■■■■■■■■■■■■■■■■■■■■■■■■□□□□■■■■■■■	777777777760771	53	T1	-334---5-3-2	Orphan	334532245422224	Orphan	0		unknown	NA
FXX162005000641	2005	M	82	■■■■■■■■■■■■■■■■■■■■■■■■■■■■■■■■□□□□■■■■■■■	777777777760771	53	T1					0		unknown	NA
FXX162000100132	2000	M	28	■■■■■■■■■■■■■■■■■■■■■■■■■■■■■■■■□□□□■■■■■■■	777777777760771	53	T1	222325143222	1551	223422---------	Orphan	1		France	P
FXX162006300766	2006	M	84	■■■■■■■■■■■■■■■■■■■■■■■■■■■■■■■■□□□□■■■■■■■	777777777760771	53	T1	222325153324	146	223534---------	Orphan	3	ETH, PZA	France	P
FXX162007101115	2007	M	27	■■■■■■■■■■■■■■■■■■■■■■■■■■■■■■■■□□□□■■■■■■■	777777777760771	53	T1	223326164322	Orphan	233632345422223	227	1		Romania	P
FXX162005100584	2005	M	78	■■■■■■■■■■■■■■■■■■■■■■■■■■■■■■■■□□□□■■■■■■■	777777777760771	53	T1	223425133322	340	234332342521223	Orphan	1		France	P
FXX162005000662	2005	M	46	■■■■■■■■■■■■■■■■■■■■■■■■■■■■■■■■□□□□■■■■■■■	777777777760771	53	T1	223125143324	231	231434---------	Orphan	0		Algeria	P
FXX162005300677	2005	F	35	■■■■■■■■■■■■■■■■■■■■■■■■■■■■■■■■□□□□■■■■■■■	777777777760771	53	T1	223125143324	231	231434---------	Orphan	3	INH	France	P
FXX162006300759	2006	M	64	■■■■■■■■■■■■■■■■■■■■■■■■■■■■■■■■□□□□■■■■■■■	777777777760771	53	T1	223125143324	231	231434---------	Orphan	3	INH	unknown	P
FXX162006300949	2006	F	40	■■■■■■■■■■■■■■■■■■■■■■■■■■■■■■■■□□□□■■■■■■■	777777777760771	53	T1	223125143324	231	231434---------	Orphan	3	INH	Algeria	P
FXX162006301057	2006	M	57	■■■■■■■■■■■■■■■■■■■■■■■■■■■■■■■■□□□□■■■■■■■	777777777760771	53	T1	223215153324	263	232534---------	Orphan	3	ETH	France	P
FXX162003100420	2003	F	45	■■■■■■■■■■■■■■■■■■■■■■■■■■■■■■■■□□□□■■■■■■■	777777777760771	53	T1	223225153324	169	232534---------	Orphan	1		unknown	P
FXX162005100629	2005	M	61	■■■■■■■■■■■■■■■■■■■■■■■■■■■■■■■■□□□□■■■■■■■	777777777760771	53	T1	223225153324	169	232534---------	Orphan	1		France	P
FXX162006100761	2006	F	79	■■■■■■■■■■■■■■■■■■■■■■■■■■■■■■■■□□□□■■■■■■■	777777777760771	53	T1	223225153324	169	232534---------	Orphan	1		France	P
FXX162007101214	2007	M	85	■■■■■■■■■■■■■■■■■■■■■■■■■■■■■■■■□□□□■■■■■■■	777777777760771	53	T1	223324153325	1559	233535---------	Orphan	1		Italy	P
FXX162005100539	2005	F	57	■■■■■■■■■■■■■■■■■■■■■■■■■■■■■■■■□□□□■■■■■■■	777777777760771	53	T1	223325123323	1557	233233---------	Orphan	1		Senegal	P
FXX162006100980	2006	M	77	■■■■■■■■■■■■■■■■■■■■■■■■■■■■■■■■□□□□■■■■■■■	777777777760771	53	T1	223325133324	204	233334---------	Orphan	1		unknown	P
FXX162005100625	2005	M	64	■■■■■■■■■■■■■■■■■■■■■■■■■■■■■■■■□□□□■■■■■■■	777777777760771	53	T1	223325143223	1072	233423---------	Orphan	1		unknown	P
FXX162005100582	2005	M	94	■■■■■■■■■■■■■■■■■■■■■■■■■■■■■■■■□□□□■■■■■■■	777777777760771	53	T1	223325143322	120	233432---------	Orphan	1		unknown	P
FXX162005100563	2005	F	81	■■■■■■■■■■■■■■■■■■■■■■■■■■■■■■■■□□□□■■■■■■■	777777777760771	53	T1	223325153321	401	233531---------	Orphan	1		Germany	P
FXX162005100535	2005	M	90	■■■■■■■■■■■■■■■■■■■■■■■■■■■■■■■■□□□□■■■■■■■	777777777760771	53	T1	223325153322	15	233532---------	Orphan	1		France	P
FXX162005100590	2005	M	35	■■■■■■■■■■■■■■■■■■■■■■■■■■■■■■■■□□□□■■■■■■■	777777777760771	53	T1	223325153322	15	233532---------	Orphan	1		Algeria	P
FXX162005100623	2005	M	46	■■■■■■■■■■■■■■■■■■■■■■■■■■■■■■■■□□□□■■■■■■■	777777777760771	53	T1	223325153322	15	233532---------	Orphan	1		France	P
FXX162006100783	2006	M	71	■■■■■■■■■■■■■■■■■■■■■■■■■■■■■■■■□□□□■■■■■■■	777777777760771	53	T1	223325153322	15	233532---------	Orphan	1		unknown	P
FXX162006100990	2006	F	59	■■■■■■■■■■■■■■■■■■■■■■■■■■■■■■■■□□□□■■■■■■■	777777777760771	53	T1	223325153322	15	233532---------	Orphan	1		Algeria	P
FXX162006001027	2006	F	83	■■■■■■■■■■■■■■■■■■■■■■■■■■■■■■■■□□□□■■■■■■■	777777777760771	53	T1	223325153322	15	233532---------	Orphan	0		France	P
FXX162006300764	2006	M	22	■■■■■■■■■■■■■■■■■■■■■■■■■■■■■■■■□□□□■■■■■■■	777777777760771	53	T1	223325153336	Orphan	233536---------	Orphan	3	SM, INH	Benin	P
FXX162005100615	2005	F	26	■■■■■■■■■■■■■■■■■■■■■■■■■■■■■■■■□□□□■■■■■■■	777777777760771	53	T1	224325153323	33	243533333512243	Orphan	1		France	P
FXX162006300957	2006	M	83	■■■■■■■■■■■■■■■■■■■■■■■■■■■■■■■■□□□□■■■■■■■	777777777760771	53	T1	224325153323	33	243533333712343	263	3	PZA	Morocco	P
FXX162006100681	2006	M	76	■■■■■■■■■■■■■■■■■■■■■■■■■■■■■■■■□□□□■■■■■■■	777777777760771	53	T1	224125143222	Orphan	241422---------	Orphan	1		Spain	P
FXX162006100788	2006	M	79	■■■■■■■■■■■■■■■■■■■■■■■■■■■■■■■■□□□□■■■■■■■	777777777760771	53	T1	224325153425	Orphan	243545---------	Orphan	1		Algeria	P
FXX162006100831	2006	M	33	■■■■■■■■■■■■■■■■■■■■■■■■■■■■■■■■□□□□■■■■■■■	777777777760771	53	T1	225325153323	45	253533332412443	Orphan	1		Côte d'Ivoire	P
FXX162000300125	2000	M	34	■■■■■■■■■■■■■■■■■■■■■■■■■■■■■■■■□□□□■■■■■■■	777777777760771	53	T1	227315161423	Orphan	273643---------	Orphan	3	SM	Algeria	P
FXX162006100975	2006	M	58	■■■■■■■■■■■■■■■■■■■■■■■■■■■■■■■■□□□□■■■■■■■	777777777760771	53	T1	227325163423	802	273643---------	Orphan	1		France	P
FXX162006100692	2006	F	34	■■■■■■■■■■■■■■■■■■■■■■■■■■■■■■■■□□□□■■■■■■■	777777777760771	53	T1	227325163523	Orphan	273653---------	Orphan	1		France	P
FXX162005100616	2005	F	18	■■■■■■■■■■■■■■■■■■■■■■■■■■■■■■■■□□□□■■■■■■■	777777777760771	53	T1	262125143024	Orphan	621404---------	Orphan	1		Angola	P
FXX162006100687	2006	M	67	■■■■■■■■■■■■■■■■■■■■■■■■■■■■■■■■□□□□■■■■■■■	777777777760771	53	T1	323125133325	Orphan	231335---------	Orphan	1		Guinea	P

FXX162008101693	2008	F	86	■■■■■■■■■■■■■■■■■■■■■■■■■■■■■■■■□□□□■■■■■■■	777777777760771	53	T1	-145---5-3-2	Orphan	145532221622222	Orphan	1		unknown	P
FXX162009101759	2009	F	81	■■■■■■■■■■■■■■■■■■■■■■■■■■■■■■■■□□□□■■■■■■■	777777777760771	53	T1	-223---5-2-1	Orphan	223521344623224	Orphan	1		France	P
FXX162010102244	2010	M	78	■■■■■■■■■■■■■■■■■■■■■■■■■■■■■■■■□□□□■■■■■■■	777777777760771	53	T1	-233---5-3-2	Orphan	233532232512123	Orphan	1		France	P
FXX162008101660	2008	M	39	■■■■■■■■■■■■■■■■■■■■■■■■■■■■■■■■□□□□■■■■■■■	777777777760771	53	T1	-233---4-2-2	Orphan	233422244422224	304	1		Germany	P
FXX162009101947	2009	F	25	■■■■■■■■■■■■■■■■■■■■■■■■■■■■■■■■□□□□■■■■■■■	777777777760771	53	T1	-233---4-2-2	Orphan	233422244422224	304	1		Congo	P
FXX162009101944	2009	M	90	■■■■■■■■■■■■■■■■■■■■■■■■■■■■■■■■□□□□■■■■■■■	777777777760771	53	T1	-234---3-3-2	Orphan	234332244522223	Orphan	1		unknown	P
FXX162008101695	2008	F	77	■■■■■■■■■■■■■■■■■■■■■■■■■■■■■■■■□□□□■■■■■■■	777777777760771	53	T1	-234---5-3-2	Orphan	234532245522223	313	1		unknown	P
FXX162010102247	2010	F	90	■■■■■■■■■■■■■■■■■■■■■■■■■■■■■■■■□□□□■■■■■■■	777777777760771	53	T1	-234---5-3-2	Orphan	234532244522223	311	1		France	P
FXX162008101653	2008	M	49	■■■■■■■■■■■■■■■■■■■■■■■■■■■■■■■■□□□□■■■■■■■	777777777760771	53	T1	-231---5-3-5	Orphan	231535344522223	Orphan	1		unknown	P
FXX162010102118	2010	M	48	■■■■■■■■■■■■■■■■■■■■■■■■■■■■■■■■□□□□■■■■■■■	777777777760771	53	T1	-233---5-3-4	Orphan	233534323522223	Orphan	1		unknown	P
FXX162010102354	2010	M	64	■■■■■■■■■■■■■■■■■■■■■■■■■■■■■■■■□□□□■■■■■■■	777777777760771	53	T1	-233---3-2-4	Orphan	233324343532122	Orphan	1		unknown	P
FXX162010102061	2010	M	79	■■■■■■■■■■■■■■■■■■■■■■■■■■■■■■■■□□□□■■■■■■■	777777777760771	53	T1	-233---5-2-4	Orphan	233524342522126	Orphan	1		Algeria	P
FXX162009101993	2009	M	2	■■■■■■■■■■■■■■■■■■■■■■■■■■■■■■■■□□□□■■■■■■■	777777777760771	53	T1	-233---5-3-1	Orphan	233531344422224	320	1		unknown	P
FXX162008001429	2008	M	22	■■■■■■■■■■■■■■■■■■■■■■■■■■■■■■■■□□□□■■■■■■■	777777777760771	53	T1	-233---5-3-3	Orphan	233533343522223	322	0		unknown	P
FXX162009101815	2009	M	26	■■■■■■■■■■■■■■■■■■■■■■■■■■■■■■■■□□□□■■■■■■■	777777777760771	53	T1	-233---5-3-3	Orphan	233533343522223	322	1		France	P
FXX162009102007	2009	M	23	■■■■■■■■■■■■■■■■■■■■■■■■■■■■■■■■□□□□■■■■■■■	777777777760771	53	T1	-233---5-3-3	Orphan	233533343522223	322	1		unknown	P
FXX162010002359	2010	M	30	■■■■■■■■■■■■■■■■■■■■■■■■■■■■■■■■□□□□■■■■■■■	777777777760771	53	T1	-233---5-3-3	Orphan	233533343522223	322	0		unknown	P
FXX162008101594	2008	M	31	■■■■■■■■■■■■■■■■■■■■■■■■■■■■■■■■□□□□■■■■■■■	777777777760771	53	T1	-242---5-3-4	Orphan	242534342522223	Orphan	1		unknown	P
FXX162008101431	2008	M	27	■■■■■■■■■■■■■■■■■■■■■■■■■■■■■■■■□□□□■■■■■■■	777777777760771	53	T1	-243---5-3-3	Orphan	243533332712343	347	1		unknown	P
FXX162009101772	2009	F	30	■■■■■■■■■■■■■■■■■■■■■■■■■■■■■■■■□□□□■■■■■■■	777777777760771	53	T1	-243---5-3-3	Orphan	243533332712343	347	1		unknown	P
FXX162010102001	2010	F	24	■■■■■■■■■■■■■■■■■■■■■■■■■■■■■■■■□□□□■■■■■■■	777777777760771	53	T1	-243---5-3-3	Orphan	243533333712343	263	1		unknown	P
FXX162010102018	2010	F	3	■■■■■■■■■■■■■■■■■■■■■■■■■■■■■■■■□□□□■■■■■■■	777777777760771	53	T1	-243---5-3-3	Orphan	243533333712343	263	1		unknown	P
FXX162010102117	2010	M	28	■■■■■■■■■■■■■■■■■■■■■■■■■■■■■■■■□□□□■■■■■■■	777777777760771	53	T1	-243---5-3-3	Orphan	243533333712343	263	1		France	P
FXX162010102245	2010	M	33	■■■■■■■■■■■■■■■■■■■■■■■■■■■■■■■■□□□□■■■■■■■	777777777760771	53	T1	-251---1-3-2	Orphan	251132322431343	Orphan	1		Algeria	P
FXX162008101662	2008	M	38	■■■■■■■■■■■■■■■■■■■■■■■■■■■■■■■■□□□□■■■■■■■	777777777760771	53	T1	-251---1-3-4	Orphan	251134322931343	352	1		unknown	P
FXX162008101665	2008	M	34	■■■■■■■■■■■■■■■■■■■■■■■■■■■■■■■■□□□□■■■■■■■	777777777760771	53	T1	-251---1-3-4	Orphan	251134322931343	352	1		unknown	P
FXX162009101803	2009	M	36	■■■■■■■■■■■■■■■■■■■■■■■■■■■■■■■■□□□□■■■■■■■	777777777760771	53	T1	-251---1-3-4	Orphan	251134322931343	352	1		unknown	P
FXX162009101808	2009	M	42	■■■■■■■■■■■■■■■■■■■■■■■■■■■■■■■■□□□□■■■■■■■	777777777760771	53	T1	-251---1-3-4	Orphan	251134322931343	352	1		France	P
FXX162009101909	2009	M	47	■■■■■■■■■■■■■■■■■■■■■■■■■■■■■■■■□□□□■■■■■■■	777777777760771	53	T1	-283---6-4-3	Orphan	28364333261274B	Orphan	1		Tunisia	P
FXX162001300008	2001	F	85	■■■■■■■■■■■■■■■■■■■■■■■■■■■■■■■■□□□□■■■■■■■	777777777760771	53	T1					3	SM	France	P
FXX162001100010	2001	M	39	■■■■■■■■■■■■■■■■■■■■■■■■■■■■■■■■□□□□■■■■■■■	777777777760771	53	T1					1		Algeria	P
FXX162001100016	2001	M	22	■■■■■■■■■■■■■■■■■■■■■■■■■■■■■■■■□□□□■■■■■■■	777777777760771	53	T1					1		France	P
FXX162001300017	2001	M	55	■■■■■■■■■■■■■■■■■■■■■■■■■■■■■■■■□□□□■■■■■■■	777777777760771	53	T1					3	SM	Algeria	P
FXX162003100019	2003	M	61	■■■■■■■■■■■■■■■■■■■■■■■■■■■■■■■■□□□□■■■■■■■	777777777760771	53	T1					1		France	P
FXX162003100024	2003	M	41	■■■■■■■■■■■■■■■■■■■■■■■■■■■■■■■■□□□□■■■■■■■	777777777760771	53	T1					1		France	P
FXX162001100026	2001	M	51	■■■■■■■■■■■■■■■■■■■■■■■■■■■■■■■■□□□□■■■■■■■	777777777760771	53	T1					1		Tunisia	P
FXX162001300032	2001	M	20	■■■■■■■■■■■■■■■■■■■■■■■■■■■■■■■■□□□□■■■■■■■	777777777760771	53	T1					3	SM	Côte d'Ivoire	P
FXX162001100037	2001	M	76	■■■■■■■■■■■■■■■■■■■■■■■■■■■■■■■■□□□□■■■■■■■	777777777760771	53	T1					1		France	P
FXX162001100041	2001	M	49	■■■■■■■■■■■■■■■■■■■■■■■■■■■■■■■■□□□□■■■■■■■	777777777760771	53	T1					1		France	P
FXX162001300052	2001	M	71	■■■■■■■■■■■■■■■■■■■■■■■■■■■■■■■■□□□□■■■■■■■	777777777760771	53	T1					3	SM	Algeria	P
FXX162001100064	2001	M	84	■■■■■■■■■■■■■■■■■■■■■■■■■■■■■■■■□□□□■■■■■■■	777777777760771	53	T1					1		France	P
FXX162001100071	2001	M	34	■■■■■■■■■■■■■■■■■■■■■■■■■■■■■■■■□□□□■■■■■■■	777777777760771	53	T1					1		France	P
FXX162003100074	2003	F	80	■■■■■■■■■■■■■■■■■■■■■■■■■■■■■■■■□□□□■■■■■■■	777777777760771	53	T1					1		France	P
FXX162003100116	2003	M	67	■■■■■■■■■■■■■■■■■■■■■■■■■■■■■■■■□□□□■■■■■■■	777777777760771	53	T1					1		France	P
FXX162000100124	2000	M	40	■■■■■■■■■■■■■■■■■■■■■■■■■■■■■■■■□□□□■■■■■■■	777777777760771	53	T1					1		France	P
FXX162000300131	2000	F	84	■■■■■■■■■■■■■■■■■■■■■■■■■■■■■■■■□□□□■■■■■■■	777777777760771	53	T1					3	SM	France	P
FXX162003100136	2003	M	56	■■■■■■■■■■■■■■■■■■■■■■■■■■■■■■■■□□□□■■■■■■■	777777777760771	53	T1					1		France	P
FXX162000300142	2000	F	86	■■■■■■■■■■■■■■■■■■■■■■■■■■■■■■■■□□□□■■■■■■■	777777777760771	53	T1					3	SM	France	P
FXX162000300144	2000	M	68	■■■■■■■■■■■■■■■■■■■■■■■■■■■■■■■■□□□□■■■■■■■	777777777760771	53	T1					3	SM	Algeria	P
FXX162000100152	2000	M	78	■■■■■■■■■■■■■■■■■■■■■■■■■■■■■■■■□□□□■■■■■■■	777777777760771	53	T1					1		Algeria	P
FXX162000100157	2000	M	45	■■■■■■■■■■■■■■■■■■■■■■■■■■■■■■■■□□□□■■■■■■■	777777777760771	53	T1					1		France	P
FXX162000300161	2000	M	73	■■■■■■■■■■■■■■■■■■■■■■■■■■■■■■■■□□□□■■■■■■■	777777777760771	53	T1					3	SM	Algeria	P
FXX162000300168	2000	F	67	■■■■■■■■■■■■■■■■■■■■■■■■■■■■■■■■□□□□■■■■■■■	777777777760771	53	T1					3	PZA	Algeria	P
FXX162002100172	2002	M	70	■■■■■■■■■■■■■■■■■■■■■■■■■■■■■■■■□□□□■■■■■■■	777777777760771	53	T1					1		France	P
FXX162002100186	2002	F	72	■■■■■■■■■■■■■■■■■■■■■■■■■■■■■■■■□□□□■■■■■■■	777777777760771	53	T1					1		France	P
FXX162002100192	2002	F	77	■■■■■■■■■■■■■■■■■■■■■■■■■■■■■■■■□□□□■■■■■■■	777777777760771	53	T1					1		France	P
FXX162002100195	2002	M	76	■■■■■■■■■■■■■■■■■■■■■■■■■■■■■■■■□□□□■■■■■■■	777777777760771	53	T1					1		France	P

FXX162002100208	2002	M	77	■■■■■■■■■■■■■■■■■■■■■■■■■■■■■■■■□□□□■■■■■■■	777777777760771	53	T1					1		Spain	P
FXX162002100211	2002	M	58	■■■■■■■■■■■■■■■■■■■■■■■■■■■■■■■■□□□□■■■■■■■	777777777760771	53	T1					1		Maghreb	P
FXX162002100213	2002	M	27	■■■■■■■■■■■■■■■■■■■■■■■■■■■■■■■■□□□□■■■■■■■	777777777760771	53	T1					1		Turkey	P
FXX162002100221	2002	F	83	■■■■■■■■■■■■■■■■■■■■■■■■■■■■■■■■□□□□■■■■■■■	777777777760771	53	T1					1		France	P
FXX162002100231	2002	M	36	■■■■■■■■■■■■■■■■■■■■■■■■■■■■■■■■□□□□■■■■■■■	777777777760771	53	T1					1		Algeria	P
FXX162002100239	2002	F	76	■■■■■■■■■■■■■■■■■■■■■■■■■■■■■■■■□□□□■■■■■■■	777777777760771	53	T1					1		France	P
FXX162002100250	2002	F	82	■■■■■■■■■■■■■■■■■■■■■■■■■■■■■■■■□□□□■■■■■■■	777777777760771	53	T1					1		France	P
FXX162000100269	2000	F	38	■■■■■■■■■■■■■■■■■■■■■■■■■■■■■■■■□□□□■■■■■■■	777777777760771	53	T1					1		USA	P
FXX162000100285	2000	M	70	■■■■■■■■■■■■■■■■■■■■■■■■■■■■■■■■□□□□■■■■■■■	777777777760771	53	T1					1		France	P
FXX162003100318	2003	F	89	■■■■■■■■■■■■■■■■■■■■■■■■■■■■■■■■□□□□■■■■■■■	777777777760771	53	T1					1		France	P
FXX162000100332	2000	M	28	■■■■■■■■■■■■■■■■■■■■■■■■■■■■■■■■□□□□■■■■■■■	777777777760771	53	T1					1		France	P
FXX162000300335	2000	M	79	■■■■■■■■■■■■■■■■■■■■■■■■■■■■■■■■□□□□■■■■■■■	777777777760771	53	T1					3	SM	France	P
FXX162000000343	2000	F	37	■■■■■■■■■■■■■■■■■■■■■■■■■■■■■■■■□□□□■■■■■■■	777777777760771	53	T1					0		France	P
FXX162000000357	2000	M	78	■■■■■■■■■■■■■■■■■■■■■■■■■■■■■■■■□□□□■■■■■■■	777777777760771	53	T1					0		Italy	P
FXX162000000361	2000	M	37	■■■■■■■■■■■■■■■■■■■■■■■■■■■■■■■■□□□□■■■■■■■	777777777760771	53	T1					0		Vietnam	P
FXX162000000364	2000	M	29	■■■■■■■■■■■■■■■■■■■■■■■■■■■■■■■■□□□□■■■■■■■	777777777760771	53	T1					0		France	P
FXX162000000379	2000	M	77	■■■■■■■■■■■■■■■■■■■■■■■■■■■■■■■■□□□□■■■■■■■	777777777760771	53	T1					0		France	P
FXX162000000387	2000	M	58	■■■■■■■■■■■■■■■■■■■■■■■■■■■■■■■■□□□□■■■■■■■	777777777760771	53	T1					0		France	P
FXX162000000393	2000	M	59	■■■■■■■■■■■■■■■■■■■■■■■■■■■■■■■■□□□□■■■■■■■	777777777760771	53	T1					0		France	P
FXX162000000399	2000	F	23	■■■■■■■■■■■■■■■■■■■■■■■■■■■■■■■■□□□□■■■■■■■	777777777760771	53	T1					0		France	P
FXX162003100413	2003	M	80	■■■■■■■■■■■■■■■■■■■■■■■■■■■■■■■■□□□□■■■■■■■	777777777760771	53	T1					1		Algeria	P
FXX162003100435	2003	F	69	■■■■■■■■■■■■■■■■■■■■■■■■■■■■■■■■□□□□■■■■■■■	777777777760771	53	T1					1		France	P
FXX162004100441	2004	M	24	■■■■■■■■■■■■■■■■■■■■■■■■■■■■■■■■□□□□■■■■■■■	777777777760771	53	T1					1		Romania	P
FXX162004100443	2004	M	85	■■■■■■■■■■■■■■■■■■■■■■■■■■■■■■■■□□□□■■■■■■■	777777777760771	53	T1					1		Italy	P
FXX162004100448	2004	F	91	■■■■■■■■■■■■■■■■■■■■■■■■■■■■■■■■□□□□■■■■■■■	777777777760771	53	T1					1		France	P
FXX162004100453	2004	F	94	■■■■■■■■■■■■■■■■■■■■■■■■■■■■■■■■□□□□■■■■■■■	777777777760771	53	T1					1		France	P
FXX162004100460	2004	M	62	■■■■■■■■■■■■■■■■■■■■■■■■■■■■■■■■□□□□■■■■■■■	777777777760771	53	T1					1		France	P
FXX162004100468	2004	M	84	■■■■■■■■■■■■■■■■■■■■■■■■■■■■■■■■□□□□■■■■■■■	777777777760771	53	T1					1		France	P
FXX162004300485	2004	M	27	■■■■■■■■■■■■■■■■■■■■■■■■■■■■■■■■□□□□■■■■■■■	777777777760771	53	T1					3	SM	Congo	P
FXX162004000493	2004	M	73	■■■■■■■■■■■■■■■■■■■■■■■■■■■■■■■■□□□□■■■■■■■	777777777760771	53	T1					0		France	P
FXX162004100503	2004	F	91	■■■■■■■■■■■■■■■■■■■■■■■■■■■■■■■■□□□□■■■■■■■	777777777760771	53	T1					1		Algeria	P
FXX162004100505	2004	M	38	■■■■■■■■■■■■■■■■■■■■■■■■■■■■■■■■□□□□■■■■■■■	777777777760771	53	T1					1		France	P
FXX162004100507	2004	M	3	■■■■■■■■■■■■■■■■■■■■■■■■■■■■■■■■□□□□■■■■■■■	777777777760771	53	T1					1		Somalia	P
FXX162008101537	2008	F	33	■■■■■■■■■■■■■■■■■■■■■■■■■■■■■■■■□□□□■■■■■■■	777777777760771	53	T1					1		unknown	P
FXX162007101104	2007	F	22	■■■■■■■■■■■■■■■■■■■■■■■■■■■■■■■■□□□□■■■■■■■	777777777760771	53	T1	215125113322	310	151132---------	Orphan	1		unknown	P
FXX162007001301	2007	F	82	■■■■■■■■■■■■■■■■■■■■■■■■■■■■■■■■□□□□■■■■■■■	777777777760771	53	T1	2234251533-2	Orphan	234532234522223	225	0		Algeria	P
FXX162007101296	2007	M	55	■■■■■■■■■■■■■■■■■■■■■■■■■■■■■■■■□□□□■■■■■■■	777777777760771	53	T1	22332616-3-2	Orphan	233632345422223	227	1		Romania	P
FXX162007101166	2007	M	63	■■■■■■■■■■■■■■■■■■■■■■■■■■■■■■■■□□□□■■■■■■■	777777777760771	53	T1	223125152225	Orphan	231525---------	Orphan	1		Maghreb	P
FXX162007101208	2007	M	28	■■■■■■■■■■■■■■■■■■■■■■■■■■■■■■■■□□□□■■■■■■■	777777777760771	53	T1	223425153323	382	234533---------	Orphan	1		Romania	P
FXX162007101169	2007	M	23	■■■■■■■■■■■■■■■■■■■■■■■■■■■■■■■■□□□□■■■■■■■	777777777760771	53	T1	224325153323	33	243533333712343	263	1		Maghreb	P
FXX162007101069	2007	M	18	■■■■■■■■■■■■■■■■■■■■■■■■■■■■■■■■□□□□■■■■■■■	777777777760771	53	T1	224325154223	845	243523333534443	Orphan	1		unknown	P
FXX162007101118	2007	F	84	■■■■■■■■■■■■■■■■■■■■■■■■■■■■■■■■□□□□■■■■■■■	777777777760771	53	T1	226325163423	1106	263643---------	Orphan	1		unknown	P
FXX162008101557	2008	M	24	■■■■■■■■■■■■■■■■■■■■■■■■■■■■■■■■□□□□■■■■■■■	777777777760771	53	T1	-231---5-3-2	Orphan	231532242522225	Orphan	1		Russie	P
FXX162008101401	2008	M	72	■■■■■■■■■■■■■■■■■■■■■■■■■■■■■■■■□□□□■■■■■■■	777777777760771	53	T1	-234---5-3-2	Orphan	234532233522223	306	1		unknown	P
FXX162008001671	2008	M	47	■■■■■■■■■■■■■■■■■■■■■■■■■■■■■■■■□□□□■■■■■■■	777777777760771	53	T1	-234---5-3-2	Orphan	234532234522223	225	0		Algeria	P
FXX162008001674	2008	M	15	■■■■■■■■■■■■■■■■■■■■■■■■■■■■■■■■□□□□■■■■■■■	777777777760771	53	T1	-234---5-3-2	Orphan	234532234522223	225	0		Romania	P
FXX162008001504	2008	M	80	■■■■■■■■■■■■■■■■■■■■■■■■■■■■■■■■□□□□■■■■■■■	777777777760771	53	T1	-234---5-3-2	Orphan	234532243422223	Orphan	0		unknown	P
FXX162008001460	2008	M	52	■■■■■■■■■■■■■■■■■■■■■■■■■■■■■■■■□□□□■■■■■■■	777777777760771	53	T1	-234---5-3-3	Orphan	234533245523223	314	0		unknown	P
FXX162008101389	2008	F	28	■■■■■■■■■■■■■■■■■■■■■■■■■■■■■■■■□□□□■■■■■■■	777777777760771	53	T1	-232---5-3-4	Orphan	232534334322223	Orphan	1		unknown	P
FXX162008101391	2008	M	47	■■■■■■■■■■■■■■■■■■■■■■■■■■■■■■■■□□□□■■■■■■■	777777777760771	53	T1	-233---3-3-3	Orphan	233333343522221	Orphan	1		unknown	P
FXX162008101331	2008	M	89	■■■■■■■■■■■■■■■■■■■■■■■■■■■■■■■■□□□□■■■■■■■	777777777760771	53	T1	-233---5-3-2	Orphan	233532344623224	Orphan	1		unknown	P
FXX162008101673	2008	M	32	■■■■■■■■■■■■■■■■■■■■■■■■■■■■■■■■□□□□■■■■■■■	777777777760771	53	T1	-233---5-3-3	Orphan	233533343522223	322	1		Central African Republic	P
FXX162008001496	2008	F	23	■■■■■■■■■■■■■■■■■■■■■■■■■■■■■■■■□□□□■■■■■■■	777777777760771	53	T1	-251---1-3-4	Orphan	251134322-31343	Orphan	0		unknown	P
FXX162007001334	2007	F	23	■■■■■■■■■■■■■■■■■■■■■■■■■■■■■■■■□□□□■■■■■■■	777777777760771	53	T1	-273---6-4-3	Orphan	273643332632647	Orphan	0		unknown	P
FXX162008101486	2008	F	68	■■■■■■■■■■■■■■■■■■■■■■■■■■■■■■■■□□□□■■■■■■■	777777777760771	53	T1	-423---5-3-2	Orphan	423532445822121	Orphan	1		unknown	P
FXX162006100958	2006	M	37	■■■■■■■■■■■■■■■■■■■■■■■■■■■■■■■■□□□□■■■■■■■	777777777760771	53	T1	2231-5153324	Orphan	231534---------	Orphan	1		Morocco	EP and P
FXX162006100784	2006	M	18	■■■■■■■■■■■■■■■■■■■■■■■■■■■■■■■■□□□□■■■■■■■	777777777760771	53	T1	227525163423	Orphan	275643---------	Orphan	1		unknown	EP and P

FXX162008101498	2008	M	72	■■■■■■■■■■■■■■■■■■■■■■■■■■■■■■■■□□□□■■■■■■■■■■■	777777777760771	53	T1	-233---5-3-4	Orphan	233534244-23223	Orphan	1		unknown	EP and P
FXX162008001473	2008	M	31	■■■■■■■■■■■■■■■■■■□□■■□□□■■■■■■■□□□□■■■■■■■	777777143760771	57	LAM10-CAM	-233---4-3-1	Orphan	233431446522322	Orphan	0		unknown	NA
FXX162004300531	2004	M	64	■■■■■■■■■■■■■■■■■■■□■■□■■■■■■■■■□□□□■■■■■■■	777777557760771	58	T5-Madrid2					3	SM	France	EP
FXX162005000597	2005	F		■■■■■■■■■■■■■■■■■■■□■■□■■■■■■■■■□□□□■■■■■■■	777777557760771	58	T5-Madrid2	223326153324	18	233534---------	Orphan	0		unknown	NA
FXX162005000598	2005	M		■■■■■■■■■■■■■■■■■■■□■■□■■■■■■■■■□□□□■■■■■■■	777777557760771	58	T5-Madrid2	223326153324	18	233534---------	Orphan	0		unknown	NA
FXX162005000599	2005	nsp		■■■■■■■■■■■■■■■■■■■□■■□■■■■■■■■■□□□□■■■■■■■	777777557760771	58	T5-Madrid2	223326153324	18	233534---------	Orphan	0		unknown	NA
FXX162007001235	2007	F	74	■■■■■■■■■■■■■■■■■■■□■■□■■■■■■■■■□□□□■■■■■■■	777777557760771	58	T5-Madrid2	223326153325	339	233535---------	Orphan	0		unknown	NA
FXX162005000720	2005	M	19	■■■■■■■■■■■■■■■■■■■□■■□■■■■■■■■■□□□□■■■■■■■	777777557760771	58	T5-Madrid2	223326153326	19	233536---------	Orphan	0		unknown	NA
FXX162008001615	2008	M	38	■■■■■■■■■■■■■■■■■■■□■■□■■■■■■■■■□□□□■■■■■■■	777777557760771	58	T5-Madrid2	-233---5-3-6	Orphan	233536343522222	Orphan	0		unknown	NA
FXX162005100528	2005	M	19	■■■■■■■■■■■■■■■■■■■□■■□■■■■■■■■■□□□□■■■■■■■	777777557760771	58	T5-Madrid2	223326153326	19	233536---------	Orphan	1		Angola	P
FXX162002100237	2002	M	23	■■■■■■■■■■■■■■■■■■■□■■□■■■■■■■■■□□□□■■■■■■■	777777557760771	58	T5-Madrid2					1		Algeria	P
FXX162005300527	2005	M	1	■■■■■■■■■■■■■■■■■■■■□□□□■■□□□□■■□□□□■■■■■■■	777777606060771	59	LAM11-ZWE	224126142321	454	241431---------	Orphan	3	SM, INH	France	P
FXX162005100560	2005	M	48	■■■■■■■■■■■■■■■■■■■■□□□□■■■■■■■■□□□□■■■□■■■	777777607760731	60	LAM4	224216143321	1572	242431---------	Orphan	1		France	EP
FXX162007101106	2007	F	64	■■■■■■■■■■■■■■■■■■■■□□□□■■■■■■■■□□□□■■■□■■■	777777607760731	60	LAM4	226326163325	Orphan	263635---------	Orphan	1		unknown	EP
FXX162006000798	2006	F	26	■■■■■■■■■■■■■■■■■■■■□□□□■■■■■■■■□□□□■■■□■■■	777777607760731	60	LAM4	223226143321	10	232431---------	Orphan	0		unknown	NA
FXX162006001042	2006	F	43	■■■■■■■■■■■■■■■■■■■■□□□□■■■■■■■■□□□□■■■□■■■	777777607760731	60	LAM4	223226143321	10	232431---------	Orphan	0		unknown	NA
FXX162010002128	2010	M	36	■■■■■■■■■■■■■■■■■■■■□□□□■■■■■■■■□□□□■■■□■■■	777777607760731	60	LAM4	-232---4-3-1	Orphan	232431144704312	Orphan	0		unknown	NA
FXX162010002209	2010	M	49	■■■■■■■■■■■■■■■■■■■■□□□□■■■■■■■■□□□□■■■□■■■	777777607760731	60	LAM4	-231---5-3-1	Orphan	231531242825312	Orphan	0		unknown	NA
FXX162010002083	2010	F	86	■■■■■■■■■■■■■■■■■■■■□□□□■■■■■■■■□□□□■■■□■■■	777777607760731	60	LAM4	-242---2-3-1	Orphan	242231244824312	Orphan	0		unknown	NA
FXX162009001783	2009	M	75	■■■■■■■■■■■■■■■■■■■■□□□□■■■■■■■■□□□□■■■□■■■	777777607760731	60	LAM4	-242---4-3-1	Orphan	242431243924312	Orphan	0		unknown	NA
FXX162006100794	2006	M	39	■■■■■■■■■■■■■■■■■■■■□□□□■■■■■■■■□□□□■■■□■■■	777777607760731	60	LAM4	223124152321	675	231531---------	Orphan	1		unknown	P
FXX162005000593	2005	M	78	■■■■■■■■■■■■■■■■■■■■□□□□■■■■■■■■□□□□■■■□■■■	777777607760731	60	LAM4	224216143323	Orphan	242433---------	Orphan	0		France	P
FXX162001100055	2001	F	69	■■■■■■■■■■■■■■■■■■■■□□□□■■■■■■■■□□□□■■■□■■■	777777607760731	60	LAM4					1		France	P
FXX162001000077	2001	F	30	■■■■■■■■■■■■■■■■■■■■□□□□■■■■■■■■□□□□■■■□■■■	777777607760731	60	LAM4					0		Morocco	P
FXX162000100122	2000	M	48	■■■■■■■■■■■■■■■■■■■■□□□□■■■■■■■■□□□□■■■□■■■	777777607760731	60	LAM4					1		Tunisia	P
FXX162008101632	2008	M	43	■■■■■■■■■■■■■■■■■■■■□□□□■■■■■■■■□□□□■■■□■■■	777777607760731	60	LAM4	-243---8-4-5	Orphan	243845243525228	262	1		unknown	P
FXX162006100991	2006	M	54	■■■■■■■■■■■■■■■■■■■■■■□□□■■■■■■■□□□□■■■■■■■	777777743760771	61	LAM10-CAM	223215153324	263	232534---------	Orphan	1		Gabon	EP
FXX162007101318	2007	F	69	■■■■■■■■■■■■■■■■■■■■■■□□□■■■■■■■□□□□■■■■■■■	777777743760771	61	LAM10-CAM	-232---5-3-3	Orphan	23253336352232A	Orphan	1		Cameroon	EP
FXX162009101911	2009	M	26	■■■■■■■■■■■■■■■■■■■■■■□□□■■■■■■■□□□□■■■■■■■	777777743760771	61	LAM10-CAM	-233---5-3-3	Orphan	233533446522323	Orphan	1		unknown	EP
FXX162008101602	2008	F	39	■■■■■■■■■■■■■■■■■■■■■■□□□■■■■■■■□□□□■■■■■■■	777777743760771	61	LAM10-CAM	-233---5-3-4	Orphan	233534444522324	Orphan	1		unknown	EP
FXX162002100096	2002	F	32	■■■■■■■■■■■■■■■■■■■■■■□□□■■■■■■■□□□□■■■■■■■	777777743760771	61	LAM10-CAM					1		Côte d'Ivoire	EP
FXX162002100185	2002	F	40	■■■■■■■■■■■■■■■■■■■■■■□□□■■■■■■■□□□□■■■■■■■	777777743760771	61	LAM10-CAM					1		Côte d'Ivoire	EP
FXX162004100480	2004	M	9	■■■■■■■■■■■■■■■■■■■■■■□□□■■■■■■■□□□□■■■■■■■	777777743760771	61	LAM10-CAM					1		France	EP
FXX162006000907	2006	M	19	■■■■■■■■■■■■■■■■■■■■■■□□□■■■■■■■□□□□■■■■■■■	777777743760771	61	LAM10-CAM	223215153224	Orphan	232524---------	Orphan	0		unknown	NA
FXX162005000649	2005	M	38	■■■■■■■■■■■■■■■■■■■■■■□□□■■■■■■■□□□□■■■■■■■	777777743760771	61	LAM10-CAM	223215153323	264	232533---------	Orphan	0		unknown	NA
FXX162007001271	2007	nsp	6	■■■■■■■■■■■■■■■■■■■■■■□□□■■■■■■■□□□□■■■■■■■	777777743760771	61	LAM10-CAM	223215153324	263	232534---------	Orphan	0		unknown	NA
FXX162006000915	2006	F	59	■■■■■■■■■■■■■■■■■■■■■■□□□■■■■■■■□□□□■■■■■■■	777777743760771	61	LAM10-CAM	223315153323	12	233533---------	Orphan	0		unknown	NA
FXX162010002148	2010	M	27	■■■■■■■■■■■■■■■■■■■■■■□□□■■■■■■■□□□□■■■■■■■	777777743760771	61	LAM10-CAM	-223---5-3-1	Orphan	2235313445-2322	Orphan	0		unknown	NA
FXX162009002036	2009	F	21	■■■■■■■■■■■■■■■■■■■■■■□□□■■■■■■■□□□□■■■■■■■	777777743760771	61	LAM10-CAM	-223---5-3-1	Orphan	223531444522322	Orphan	0		unknown	NA
FXX162010001953	2010	M	12	■■■■■■■■■■■■■■■■■■■■■■□□□■■■■■■■□□□□■■■■■■■	777777743760771	61	LAM10-CAM	-233---5-3-3	Orphan	233533246422322	Orphan	0		unknown	NA
FXX162009001866	2009	F	30	■■■■■■■■■■■■■■■■■■■■■■□□□■■■■■■■□□□□■■■■■■■	777777743760771	61	LAM10-CAM	-232---5-3-3	Orphan	23253334452232-	Orphan	0		unknown	NA
FXX162010002331	2010	F	25	■■■■■■■■■■■■■■■■■■■■■■□□□■■■■■■■□□□□■■■■■■■	777777743760771	61	LAM10-CAM	-232---5-3-3	Orphan	232533344522329	Orphan	0		unknown	NA
FXX162010002152	2010	F	23	■■■■■■■■■■■■■■■■■■■■■■□□□■■■■■■■□□□□■■■■■■■	777777743760771	61	LAM10-CAM	-232---5-3-4	Orphan	232534343522327	Orphan	0		unknown	NA
FXX162009002068	2009	F	61	■■■■■■■■■■■■■■■■■■■■■■□□□■■■■■■■□□□□■■■■■■■	777777743760771	61	LAM10-CAM	-232---5-3-1	Orphan	23253144-42232A	Orphan	0		unknown	NA
FXX162007001304	2007	F	31	■■■■■■■■■■■■■■■■■■■■■■□□□■■■■■■■□□□□■■■■■■■	777777743760771	61	LAM10-CAM	-233---4-3-3	Orphan	233433443522325	Orphan	0		unknown	NA
FXX162010002164	2010	M	45	■■■■■■■■■■■■■■■■■■■■■■□□□■■■■■■■□□□□■■■■■■■	777777743760771	61	LAM10-CAM	-233---4-3-3	Orphan	233433444522324	Orphan	0		unknown	NA
FXX162008001450	2008	F	41	■■■■■■■■■■■■■■■■■■■■■■□□□■■■■■■■□□□□■■■■■■■	777777743760771	61	LAM10-CAM	-233---5-3-3	Orphan	23353344552242-	Orphan	0		unknown	NA
FXX162008001735	2008	M	50	■■■■■■■■■■■■■■■■■■■■■■□□□■■■■■■■□□□□■■■■■■■	777777743760771	61	LAM10-CAM	-233---5-3-3	Orphan	233533444522329	Orphan	0		unknown	NA
FXX162009001853	2009	F	46	■■■■■■■■■■■■■■■■■■■■■■□□□■■■■■■■□□□□■■■■■■■	777777743760771	61	LAM10-CAM	-233---5-3-3	Orphan	23353344452232C	Orphan	0		unknown	NA
FXX162009001932	2009	F	28	■■■■■■■■■■■■■■■■■■■■■■□□□■■■■■■■□□□□■■■■■■■	777777743760771	61	LAM10-CAM	-233---5-3-3	Orphan	23353344652232-	Orphan	0		unknown	NA
FXX162009002069	2009	M	26	■■■■■■■■■■■■■■■■■■■■■■□□□■■■■■■■□□□□■■■■■■■	777777743760771	61	LAM10-CAM	-233---5-3-3	Orphan	233533442522327	Orphan	0		unknown	NA
FXX162006101025	2006	F		■■■■■■■■■■■■■■■■■■■■■■□□□■■■■■■■□□□□■■■■■■■	777777743760771	61	LAM10-CAM	222315153321	980	223531---------	Orphan	1		Sub-Saharan Africa	P
FXX162006300819	2006	M	29	■■■■■■■■■■■■■■■■■■■■■■□□□■■■■■■■□□□□■■■■■■■	777777743760771	61	LAM10-CAM	223215153324	263	232534---------	Orphan	3	SM	Cameroon	P
FXX162005100530	2005	M	33	■■■■■■■■■■■■■■■■■■■■■■□□□■■■■■■■□□□□■■■■■■■	777777743760771	61	LAM10-CAM	223315153323	12	233533---------	Orphan	1		France	P
FXX162006100774	2006	M	23	■■■■■■■■■■■■■■■■■■■■■■□□□■■■■■■■□□□□■■■■■■■	777777743760771	61	LAM10-CAM	223315153323	12	233533---------	Orphan	1		unknown	P
FXX162006100775	2006	M	18	■■■■■■■■■■■■■■■■■■■■■■□□□■■■■■■■□□□□■■■■■■■	777777743760771	61	LAM10-CAM	223315153323	12	233533---------	Orphan	1		unknown	P
FXX162006101012	2006	M	28	■■■■■■■■■■■■■■■■■■■■■■□□□■■■■■■■□□□□■■■■■■■	777777743760771	61	LAM10-CAM	223315153323	12	233533---------	Orphan	1		Togo	P

FXX162010102279	2010	M	21	■■■■■■■■■■■■■■■■■■■■■■□□□■■■■■■■□□□□■■■■■■■	777777743760771	61	LAM10-CAM	-223---5-3-1	Orphan	223531445522322	Orphan	1		Gabon	P
FXX162002100001	2002	M	41	■■■■■■■■■■■■■■■■■■■■■■□□□■■■■■■■□□□□■■■■■■■	777777743760771	61	LAM10-CAM					1		Côte d'Ivoire	P
FXX162000300167	2000	F	24	■■■■■■■■■■■■■■■■■■■■■■□□□■■■■■■■□□□□■■■■■■■	777777743760771	61	LAM10-CAM					3	SM	Cameroon	P
FXX162002100176	2002	F	32	■■■■■■■■■■■■■■■■■■■■■■□□□■■■■■■■□□□□■■■■■■■	777777743760771	61	LAM10-CAM					1		Senegal	P
FXX162000000381	2000	M	30	■■■■■■■■■■■■■■■■■■■■■■□□□■■■■■■■□□□□■■■■■■■	777777743760771	61	LAM10-CAM					0		Senegal	P
FXX162000000402	2000	F	23	■■■■■■■■■■■■■■■■■■■■■■□□□■■■■■■■□□□□■■■■■■■	777777743760771	61	LAM10-CAM					0		Cameroon	P
FXX162003100427	2003	M	27	■■■■■■■■■■■■■■■■■■■■■■□□□■■■■■■■□□□□■■■■■■■	777777743760771	61	LAM10-CAM					1		France	P
FXX162004100440	2004	F	33	■■■■■■■■■■■■■■■■■■■■■■□□□■■■■■■■□□□□■■■■■■■	777777743760771	61	LAM10-CAM					1		Central African Republic	P
FXX162004100520	2004	M	34	■■■■■■■■■■■■■■■■■■■■■■□□□■■■■■■■□□□□■■■■■■■	777777743760771	61	LAM10-CAM					1		Cameroon	P
FXX162007101064	2007	F	21	■■■■■■■■■■■■■■■■■■■■■■□□□■■■■■■■□□□□■■■■■■■	777777743760771	61	LAM10-CAM	223315153323	12	233533---------	Orphan	1		Togo	P
FXX162008101560	2008	F	23	■■■■■■■■■■■■■■■■■■■■■■□□□■■■■■■■□□□□■■■■■■■	777777743760771	61	LAM10-CAM	-233---5-2-1	Orphan	233521446522320	Orphan	1		unknown	P
FXX162005100578	2005	F	56	■■■■■■■■■■■■■■■■■■■■■■■■■□□□□□□■□□□□■■■□■■■	777777774020731	62	H1	225323153323	43	253533---------	Orphan	1		Algeria	EP
FXX162008301549	2008	F	57	■■■■■■■■■■■■■■■■■■■■■■■■■□□□□□□■□□□□■■■□■■■	777777774020731	62	H1	-243---5-3-3	Orphan	243533333732333	Orphan	3	SM, INH	unknown	EP
FXX162008101329	2008	F	54	■■■■■■■■■■■■■■■■■■■■■■■■■□□□□□□■□□□□■■■□■■■	777777774020731	62	H1	-253---5-3-3	Orphan	253533336812333	Orphan	1		unknown	EP
FXX162008001499	2008	F	82	■■■■■■■■■■■■■■■■■■■■■■■■■□□□□□□■□□□□■■■□■■■	777777774020731	62	H1	-253---5-3-3	Orphan	253533333-32333	Orphan	0		unknown	EP
FXX162008301664	2008	F	66	■■■■■■■■■■■■■■■■■■■■■■■■■□□□□□□■□□□□■■■□■■■	777777774020731	62	H1	-253---5-3-3	Orphan	253533336732333	365	3	SM	France	EP
FXX162010302241	2010	M	63	■■■■■■■■■■■■■■■■■■■■■■■■■□□□□□□■□□□□■■■□■■■	777777774020731	62	H1	-253---5-3-3	Orphan	253533335432344	Orphan	3	SM	unknown	EP
FXX162009101946	2009	F	71	■■■■■■■■■■■■■■■■■■■■■■■■■□□□□□□■□□□□■■■□■■■	777777774020731	62	H1	-254---5-3-4	Orphan	254534336732333	Orphan	1		unknown	EP
FXX162001100089	2001	M	86	■■■■■■■■■■■■■■■■■■■■■■■■■□□□□□□■□□□□■■■□■■■	777777774020731	62	H1					1		France	EP
FXX162000300151	2000	M	70	■■■■■■■■■■■■■■■■■■■■■■■■■□□□□□□■□□□□■■■□■■■	777777774020731	62	H1					3	SM	France	EP
FXX162000000398	2000	F	61	■■■■■■■■■■■■■■■■■■■■■■■■■□□□□□□■□□□□■■■□■■■	777777774020731	62	H1					0		Algeria	EP
FXX162004100518	2004	F	40	■■■■■■■■■■■■■■■■■■■■■■■■■□□□□□□■□□□□■■■□■■■	777777774020731	62	H1					1		Central African Republic	EP
FXX162007001083	2007	F	46	■■■■■■■■■■■■■■■■■■■■■■■■■□□□□□□■□□□□■■■□■■■	777777774020731	62	H1	224226143323	1279	242433---------	Orphan	0		unknown	NA
FXX162007001085	2007	M	19	■■■■■■■■■■■■■■■■■■■■■■■■■□□□□□□■□□□□■■■□■■■	777777774020731	62	H1	224323173323	1576	243733---------	Orphan	0		unknown	NA
FXX162007001160	2007	F	26	■■■■■■■■■■■■■■■■■■■■■■■■■□□□□□□■□□□□■■■□■■■	777777774020731	62	H1	224323173323	1576	243733---------	Orphan	0		unknown	NA
FXX162007001280	2007	F	56	■■■■■■■■■■■■■■■■■■■■■■■■■□□□□□□■□□□□■■■□■■■	777777774020731	62	H1	225323153323	43	2535333357-2334	Orphan	0		unknown	NA
FXX162007001245	2007	F	82	■■■■■■■■■■■■■■■■■■■■■■■■■□□□□□□■□□□□■■■□■■■	777777774020731	62	H1	225323150424	Orphan	253544---------	Orphan	0		unknown	NA
FXX162007001198	2007	F	80	■■■■■■■■■■■■■■■■■■■■■■■■■□□□□□□■□□□□■■■□■■■	777777774020731	62	H1	225323153313	477	253533---------	Orphan	0		unknown	NA
FXX162007001153	2007	M	83	■■■■■■■■■■■■■■■■■■■■■■■■■□□□□□□■□□□□■■■□■■■	777777774020731	62	H1	225323153323	43	253533---------	Orphan	0		unknown	NA
FXX162010002275	2010	F	83	■■■■■■■■■■■■■■■■■■■■■■■■■□□□□□□■□□□□■■■□■■■	777777774020731	62	H1	-243---4-3-3	Orphan	243433333732343	Orphan	0		unknown	NA
FXX162010002287	2010	F	75	■■■■■■■■■■■■■■■■■■■■■■■■■□□□□□□■□□□□■■■□■■■	777777774020731	62	H1	-243---5-3-3	Orphan	243533335732334	Orphan	0		unknown	NA
FXX162009001845	2009	M	59	■■■■■■■■■■■■■■■■■■■■■■■■■□□□□□□■□□□□■■■□■■■	777777774020731	62	H1	-252---5-3-3	Orphan	252533333731333	Orphan	0		unknown	NA
FXX162007001348	2007	M	76	■■■■■■■■■■■■■■■■■■■■■■■■■□□□□□□■□□□□■■■□■■■	777777774020731	62	H1	-253---5-3-3	Orphan	253533334732333	183	0		unknown	NA
FXX162007001369	2007	F	28	■■■■■■■■■■■■■■■■■■■■■■■■■□□□□□□■□□□□■■■□■■■	777777774020731	62	H1	-253---5-3-3	Orphan	253533336712336	Orphan	0		unknown	NA
FXX162008001533	2008	M	81	■■■■■■■■■■■■■■■■■■■■■■■■■□□□□□□■□□□□■■■□■■■	777777774020731	62	H1	-253---5-3-3	Orphan	253533336731333	362	0		unknown	NA
FXX162008001585	2008	F	24	■■■■■■■■■■■■■■■■■■■■■■■■■□□□□□□■□□□□■■■□■■■	777777774020731	62	H1	-253---5-3-3	Orphan	253533335742343	Orphan	0		unknown	NA
FXX162009001864	2009	F	88	■■■■■■■■■■■■■■■■■■■■■■■■■□□□□□□■□□□□■■■□■■■	777777774020731	62	H1	-253---5-3-3	Orphan	253533336732333	365	0		unknown	NA
FXX162009002027	2009	M	57	■■■■■■■■■■■■■■■■■■■■■■■■■□□□□□□■□□□□■■■□■■■	777777774020731	62	H1	-253---5-3-3	Orphan	253533335732334	Orphan	0		unknown	NA
FXX162009002030	2009	M	82	■■■■■■■■■■■■■■■■■■■■■■■■■□□□□□□■□□□□■■■□■■■	777777774020731	62	H1	-253---5-3-3	Orphan	253533335732333	370	0		unknown	NA
FXX162010002049	2010	M	109	■■■■■■■■■■■■■■■■■■■■■■■■■□□□□□□■□□□□■■■□■■■	777777774020731	62	H1	-253---5-3-3	Orphan	253533334732333	183	0		unknown	NA
FXX162010002157	2010	F	60	■■■■■■■■■■■■■■■■■■■■■■■■■□□□□□□■□□□□■■■□■■■	777777774020731	62	H1	-253---5-3-3	Orphan	253533336732433	Orphan	0		unknown	NA
FXX162010002212	2010	F	87	■■■■■■■■■■■■■■■■■■■■■■■■■□□□□□□■□□□□■■■□■■■	777777774020731	62	H1	-253---5-3-3	Orphan	253533336712334	Orphan	0		unknown	NA
FXX162010002294	2010	F	76	■■■■■■■■■■■■■■■■■■■■■■■■■□□□□□□■□□□□■■■□■■■	777777774020731	62	H1	-253---5-3-3	Orphan	253533336732332	Orphan	0		unknown	NA
FXX162010002342	2010	M	64	■■■■■■■■■■■■■■■■■■■■■■■■■□□□□□□■□□□□■■■□■■■	777777774020731	62	H1	-253---5-3-3	Orphan	253533334732333	183	0		unknown	NA
FXX162008001613	2008	M	86	■■■■■■■■■■■■■■■■■■■■■■■■■□□□□□□■□□□□■■■□■■■	777777774020731	62	H1	-263---5-3-3	Orphan	263533336632343	214	0		unknown	NA
FXX162005100618	2005	F	91	■■■■■■■■■■■■■■■■■■■■■■■■■□□□□□□■□□□□■■■□■■■	777777774020731	62	H1	225313153323	42	253533---------	Orphan	1		France	P
FXX162005100583	2005	M	57	■■■■■■■■■■■■■■■■■■■■■■■■■□□□□□□■□□□□■■■□■■■	777777774020731	62	H1	225323153323	43	253533---------	Orphan	1		Morocco	P
FXX162005100612	2005	M	42	■■■■■■■■■■■■■■■■■■■■■■■■■□□□□□□■□□□□■■■□■■■	777777774020731	62	H1	225323153323	43	253533---------	Orphan	1		Morocco	P
FXX162006100984	2006	M	20	■■■■■■■■■■■■■■■■■■■■■■■■■□□□□□□■□□□□■■■□■■■	777777774020731	62	H1	225325153323	45	253533---------	Orphan	1		Venezuela	P
FXX162001100114	2001	M	61	■■■■■■■■■■■■■■■■■■■■■■■■■□□□□□□■□□□□■■■□■■■	777777774020731	62	H1					1		France	P
FXX162003100134	2003	F	70	■■■■■■■■■■■■■■■■■■■■■■■■■□□□□□□■□□□□■■■□■■■	777777774020731	62	H1					1		France	P
FXX162002100243	2002	M	33	■■■■■■■■■■■■■■■■■■■■■■■■■□□□□□□■□□□□■■■□■■■	777777774020731	62	H1					1		Algeria	P
FXX162000100331	2000	M	85	■■■■■■■■■■■■■■■■■■■■■■■■■□□□□□□■□□□□■■■□■■■	777777774020731	62	H1					1		Algeria	P
FXX162000000359	2000	M	58	■■■■■■■■■■■■■■■■■■■■■■■■■□□□□□□■□□□□■■■□■■■	777777774020731	62	H1					0		France	P
FXX162003100426	2003	M	88	■■■■■■■■■■■■■■■■■■■■■■■■■□□□□□□■□□□□■■■□■■■	777777774020731	62	H1					1		France	P
FXX162004100467	2004	M	25	■■■■■■■■■■■■■■■■■■■■■■■■■□□□□□□■□□□□■■■□■■■	777777774020731	62	H1					1		Central African Republic	P
FXX162004100479	2004	M	25	■■■■■■■■■■■■■■■■■■■■■■■■■□□□□□□■□□□□■■■□■■■	777777774020731	62	H1					1		Morocco	P

FXX162007101177	2007	F	93	■■■■■■■■■■■■■■■■■■■■■■■■■□□□□□□■□□□□■■■□■■■	777777774020731	62	H1	225324153423	Orphan	253543---------	Orphan	1		unknown	P
FXX162008101392	2008	M	63	■■■■■■■■■■■■■■■■■■■■■■■■■□□□□□□■□□□□■■■□■■■	777777774020731	62	H1	-243---5-3-3	Orphan	243533334632333	Orphan	1		unknown	P
FXX162007101065	2007	M	51	■■■■■■■■■■■■■■■■■■■■□□□□■■■■□■■■□□□□■■■■■■■	777777607560771	64	LAM6	122326153224	836	223524---------	Orphan	1		unknown	EP
FXX162007001229	2007	F	80	■■■■■■■■■■■■■■■■■■■■□□□□■■■■□■■■□□□□■■■■■■■	777777607560771	64	LAM6	124326153224	140	243524---------	Orphan	0		unknown	NA
FXX162008001520	2008	F	33	■■■■■■■■■■■■■■■■■■■■□□□□■■■■□■■■□□□□■■■■■■■	777777607560771	64	LAM6	-212---4-3-1	Orphan	212431244624312	Orphan	0		unknown	NA
FXX162008001516	2008	M	63	■■■■■■■■■■■■■■■■■■■■□□□□■■■■□■■■□□□□■■■■■■■	777777607560771	64	LAM6	-223---5-2-3	Orphan	223523242623312	Orphan	0		unknown	NA
FXX162010002267	2010	F	34	■■■■■■■■■■■■■■■■■■■■□□□□■■■■□■■■□□□□■■■■■■■	777777607560771	64	LAM6	-223---5-2-4	Orphan	223524242723312	Orphan	0		unknown	NA
FXX162008001617	2008	M	78	■■■■■■■■■■■■■■■■■■■■□□□□■■■■□■■■□□□□■■■■■■■	777777607560771	64	LAM6	-243---5-2-4	Orphan	243524262723312	Orphan	0		unknown	NA
FXX162010002079	2010	M	92	■■■■■■■■■■■■■■■■■■■■□□□□■■■■□■■■□□□□■■■■■■■	777777607560771	64	LAM6	-252---5-2-4	Orphan	252524243823312	Orphan	0		unknown	NA
FXX162006300955	2006	M	23	■■■■■■■■■■■■■■■■■■■■□□□□■■■■□■■■□□□□■■■■■■■	777777607560771	64	LAM6	122326153224	836	223524---------	Orphan	3	SM	Congo	P
FXX162010102240	2010	F	70	■■■■■■■■■■■■■■■■■■■■□□□□■■■■□■■■□□□□■■■■■■■	777777607560771	64	LAM6	-223---5-2-4	Orphan	223524242623312	Orphan	1		unknown	P
FXX162003100117	2003	F	74	■■■■■■■■■■■■■■■■■■■■□□□□■■■■□■■■□□□□■■■■■■■	777777607560771	64	LAM6					1		France	P
FXX162004100511	2004	M	23	■■■■■■■■■■■■■■■■■■■■□□□□■■■■□■■■□□□□■■■■■■■	777777607560771	64	LAM6					1		India	P
FXX162006100981	2006	F	77	■■■■■■■■■■■■■■■■■■■■■■■■■■■■■■■■□□□□■□□■■■■	777777777760471	65	T1					1		France	EP
FXX162009002072	2009	F	33	■■■■■■■■■■■■■■■■■■■■■■■■■■■■■■■■□□□□■□□■■■■	777777777760471	65	T1	-243---4-2-3	Orphan	243423336232444	Orphan	0		unknown	NA
FXX162009001859	2009	M	61	■■■■■■■■■■■■■■■■■■■■■■■■■■■■■■■■□□□□■□□■■■■	777777777760471	65	T1	-243---4-3-3	Orphan	243433336832443	Orphan	0		unknown	NA
FXX162001000106	2001	M	39	■■■■■■■■■■■■■■■■■■■■■■■■■■■■■■■■□□□□■□□■■■■	777777777760471	65	T1					0		France	P
FXX162004100464	2004	F	70	■■■■■■■□■■■■■■■■■■■■■■□■■■■■■■■■■□□□□■■■■■■■	775777757760771	68	T5					1		Algeria	EP
FXX162006000914	2006	M	56	■■■■■■■□■■■■■■■■■■■■■■□■■■■■■■■■■□□□□■■■■■■■	775777757760771	68	T5	223326153424	Orphan	233544---------	Orphan	0		unknown	NA
FXX162010002231	2010	F	79	■■■■■■■□■■■■■■■■■■■■■■□■■■■■■■■■■□□□□■■■■■■■	775777757760771	68	T5	-233---5-4-5	Orphan	233545343524224	Orphan	0		unknown	NA
FXX162000000140	2000	M	50	■■■■■■■□■■■■■■■■■■■■■■□■■■■■■■■■■□□□□■■■■■■■	775777757760771	68	T5					0		France	P
FXX162000100276	2000	F	44	■■■■■■■□■■■■■■■■■■■■■■□■■■■■■■■■■□□□□■■■■■■■	775777757760771	68	T5					1		France	P
FXX162009001816	2009	M	69	■■■■■■■■□□■■□■■■■■■■■■■■■■■■■■■■■□□□□■■■■■■■	776337777760771	71	S	-333---5-3-2	Orphan	333532-4-622123	Orphan	0		France	EP
FXX162007001219	2007	F	70	■■■■■■■■□□■■□■■■■■■■■■■■■■■■■■■■■□□□□■■■■■■■	776337777760771	71	S	333315153222	185	333522344524123	295	0		unknown	NA
FXX162008001379	2008	M	30	■■■■■■■■□□■■□■■■■■■■■■■■■■■■■■■■■□□□□■■■■■■■	776337777760771	71	S	-333---5-2-2	Orphan	333522344524123	295	0		unknown	NA
FXX162009001775	2009	M	63	■■■■■■■■□□■■□■■■■■■■■■■■■■■■■■■■■□□□□■■■■■■■	776337777760771	71	S	-333---5-2-2	Orphan	333522345B23123	Orphan	0		unknown	NA
FXX162009001974	2009	F	43	■■■■■■■■□□■■□■■■■■■■■■■■■■■■■■■■■□□□□■■■■■■■	776337777760771	71	S	-333---5-2-2	Orphan	333522344524123	295	0		unknown	NA
FXX162010002306	2010	M	91	■■■■■■■■□□■■□■■■■■■■■■■■■■■■■■■■■□□□□■■■■■■■	776337777760771	71	S	-333---5-2-2	Orphan	333522445-23123	Orphan	0		unknown	NA
FXX162003200104	2003	M	49	■■■■■■■■□□■■□■■■■■■■■■■■■■■■■■■■■□□□□■■■■■■■	776337777760771	71	S					2	INH, RIF, SM	France	P
FXX162007101109	2007	F	80	■■■■■■■■□□■■□■■■■■■■■■■■■■■■■■■■■□□□□■■■■■■■	776337777760771	71	S	333316133222	Orphan	333322---------	Orphan	1		Poland	P
FXX162010102114	2010	F	60	■■■■■■■■■■■■□■■■■■■■■■■■■■■■■■■■□□□□■■■□■■■	777737777760731	73	T	-24----5-3-1	Orphan	24-531332833342	Orphan	1		unknown	EP
FXX162008001315	2008	M	7	■■■■■■■■■■■■□■■■■■■■■■■■■■■■■■■■□□□□■■■□■■■	777737777760731	73	T	-223---4-3-5	Orphan	223435343222323	Orphan	0		unknown	NA
FXX162008001574	2008	M	87	■■■■■■■■■■■■□■■■■■■■■■■■■■■■■■■■□□□□■■■□■■■	777737777760731	73	T	-233---5-3-1	Orphan	233531234633242	Orphan	0		unknown	NA
FXX162007001358	2007	M	61	■■■■■■■■■■■■□■■■■■■■■■■■■■■■■■■■□□□□■■■□■■■	777737777760731	73	T	-233---6-3-1	Orphan	233631334833242	Orphan	0		unknown	NA
FXX162008001727	2008	F	87	■■■■■■■■■■■■□■■■■■■■■■■■■■■■■■■■□□□□■■■□■■■	777737777760731	73	T	-233---6-3-1	Orphan	233631334633242	Orphan	0		unknown	NA
FXX162007101212	2007	F	56	■■■■■■■■■■■■□■■■■■■■■■■■■■■■■■■■□□□□■■■□■■■	777737777760731	73	T	222325154422	1554	223542---------	Orphan	1		France	P
FXX162005100558	2005	F	33	■■■■■■■■■■■■□■■■■■■■■■■■■■■■■■■■□□□□■■■□■■■	777737777760731	73	T	224325153221	Orphan	243521---------	Orphan	1		Angola	P
FXX162002100245	2002	M	32	■■■■■■■■■■■■□■■■■■■■■■■■■■■■■■■■□□□□■■■□■■■	777737777760731	73	T					1		France	P
FXX162003100348	2003	M	34	■■■■■■■■■■■■□■■■■■■■■■■■■■■■■■■■□□□□■■■□■■■	777737777760731	73	T					1		France	P
FXX162004100444	2004	F	27	■■■■■■■■■■■■□■■■■■■■■■■■■■■■■■■■□□□□■■■□■■■	777737777760731	73	T					1		Madagascar	P
FXX162008101437	2008	F	42	■■■■■■■■■■■■□■■■■■■■■■■■■■■■■■■■□□□□■■■□■■■	777737777760731	73	T	-243---6-2-1	Orphan	243621343833242	Orphan	1		unknown	P
FXX162004100484	2004	F	33	■■■■■■■■■■■■■■■■■■■■□□□□□□□□□■■■□□□□■■■■■■■	777777600160771	74	Unknown					1		France	EP
FXX162010102063	2010	M	24	■■■■■■■■■■■■■■■■■■■■■■■■■■■■■■■■□□□□■■■□□■■	777777777760711	78	T	-251---1-3-2	Orphan	251132332333242	Orphan	1		Haiti	EP
FXX162010002084	2010	M	32	■■■■■■■■■■■■■■■■■■■■■■■■■■■■■■■■□□□□■■■□□■■	777777777760711	78	T	-233---4-3-7	Orphan	233437334612443	Orphan	0		unknown	NA
FXX162005100556	2005	F	64	■■■■■■■■■□■■■■■■■■■■□□□□■■■■□■■■□□□□■■■■■■■	777377607560771	81	LAM9	114326153322	1544	143532---------	Orphan	1		Algeria	P
FXX162000000405	2000	M	29	■■■■■■■■■■■■■■■■■■■■■□■■■■■■■■■■□□□□■■■■■■■	777777737760771	86	T1					0		Madagascar	EP
FXX162003100415	2003	F	74	■■■■■■■■■■■■■■■■■■■■■□■■■■■■■■■■□□□□■■■■■■■	777777737760771	86	T1					1		France	EP
FXX162009001857	2009	F	94	■■■■■■■■■■■■■■■■■■■■■□■■■■■■■■■■□□□□■■■■■■■	777777737760771	86	T1	-224---4-3-2	Orphan	224432244522221	Orphan	0		unknown	NA
FXX162009001829	2009	F	45	■■■■■■■■■■■■■■■■■■■■■□■■■■■■■■■■□□□□■■■■■■■	777777737760771	86	T1	-234---5-3-2	Orphan	234532233522224	Orphan	0		unknown	NA
FXX162009001981	2009	M	89	■■■■■■■■■■■■■■■■■■■■■□■■■■■■■■■■□□□□■■■■■■■	777777737760771	86	T1	-234---5-3-2	Orphan	234532235522224	Orphan	0		unknown	NA
FXX162002100070	2002	F	24	■■■■■■■■■■■■■■■■■■■■■□■■■■■■■■■■□□□□■■■■■■■	777777737760771	86	T1					1		France	P
FXX162008101440	2008	M	29	■■■■■■■■■■■■■■■■■■■■■□■■■■■■■■■■□□□□■■■■■■■	777777737760771	86	T1	-234---5-3-2	Orphan	234532234722224	Orphan	1		unknown	P
FXX162007101062	2007	M	69	■■■■■■■■□□■■□■■■■■■■■■■■■■■■■■■■■□□□□■■■□■■■	776337777760731	88	S	333325153223	1590	333523---------	Orphan	1		unknown	EP
FXX162008101624	2008	F	65	■■■■■■■■□□■■□■■■■■■■■■■■■■■■■■■■■□□□□■■■□■■■	776337777760731	88	S	-333---5-2-2	Orphan	333522446923124	390	1		unknown	EP
FXX162010102179	2010	M	55	■■■■■■■■□□■■□■■■■■■■■■■■■■■■■■■■■□□□□■■■□■■■	776337777760731	88	S	-333---5-2-2	Orphan	333522446923124	390	1		Turkey	P
FXX162009301690	2009	M	71	■■■■■■■■□□■■□■■■■■■■■■■■■■■■■■■■■□□□□■■■□■■■	776337777760731	88	S	-333---5-2-3	Orphan	333523446923123	Orphan	3	INH	Algeria	P
FXX162001100031	2001	F	39	■■■■■■■■□□■■□■■■■■■■■■■■■■■■■■■■■□□□□■■■□■■■	776337777760731	88	S					1		France	P

FXX162003100133	2003	M	33	■■■■■■■■□□■■□■■■■■■■■■■■■■■■■■■■□□□□■■■□■■■	776337777760731	88	S					1		France	P
FXX162003100326	2003	M	29	■■■■■■■■□□■■□■■■■■■■■■■■■■■■■■■■□□□□■■■□■■■	776337777760731	88	S					1		Turkey	P
FXX162007101325	2007	M	76	■■■■■■■■□□■■□■■■■■■■■■■■■■■■■■■■□□□□■■■□■■■	776337777760731	88	S	-333---5-2-2	Orphan	333522445823123	Orphan	1		France	P
FXX162010002135	2010	F	51	■■□■■■■□□□□□□□□□□□□□□□□□□■■■□□□□■□■■■■■■■■■	674000003413771	89	EAI2-nonthaburi	-553---2-4-2	Orphan	553242441811222	Orphan	0		unknown	NA
FXX162006100795	2006	M	42	■■■□□□□□□□□□□■■■■□■■■■■■■■■■■■■■□□□□■■■■■■■	700036777760771	91	X3	224324153324	815	243534---------	Orphan	1		unknown	EP
FXX162010102168	2010	M	22	■■■□□□□□□□□□■■■■■□■■■■■■■■■■■■■■□□□□■■■■■■■	700076777760771	92	X3	-243---5-3-2	Orphan	243532333834344	Orphan	1		unknown	EP
FXX162007101210	2007	F	19	■■■□□□□□□□□□■■■■■□■■■■■■■■■■■■■■□□□□■■■■■■■	700076777760771	92	X3	224325153322	32	243532---------	Orphan	1		unknown	P
FXX162006100954	2006	F	38	■■■□□□□□□□□□■■■■■□■■■■■■■■■■■■■■□□□□■■■■■■■	700076777760771	92	X3	-243---5-3-2	Orphan	243532333-34344	Orphan	1		Angola	P
FXX162000300162	2000	M	48	■■■■■■■■■■■■□■■■■■■□□□□■■■■■■■■□□□□■■■■■■■	777737607760771	93	LAM5					3	SM	France	EP
FXX162006000864	2006	F	27	■■■■■■■■■■■■□■■■■■■□□□□■■■■■■■■□□□□■■■■■■■	777737607760771	93	LAM5	114326153322	1544	143532---------	Orphan	0		unknown	NA
FXX162006000898	2006	M	31	■■■■■■■■■■■■□■■■■■■□□□□■■■■■■■■□□□□■■■■■■■	777737607760771	93	LAM5	114326153322	1544	143532---------	Orphan	0		unknown	NA
FXX162006000912	2006	M	71	■■■■■■■■■■■■□■■■■■■□□□□■■■■■■■■□□□□■■■■■■■	777737607760771	93	LAM5	124326123325	Orphan	243235---------	Orphan	0		unknown	NA
FXX162006000904	2006	M	62	■■■■■■■■■■■■□■■■■■■□□□□■■■■■■■■□□□□■■■■■■■	777737607760771	93	LAM5	124326153225	427	243525---------	Orphan	0		unknown	NA
FXX162008001411	2008	M	85	■■■■■■■■■■■■□■■■■■■□□□□■■■■■■■■□□□□■■■■■■■	777737607760771	93	LAM5	-242---5-3-1	Orphan	242531242624312	Orphan	0		unknown	NA
FXX162010002178	2010	F	79	■■■■■■■■■■■■□■■■■■■□□□□■■■■■■■■□□□□■■■■■■■	777737607760771	93	LAM5	-242---5-3-1	Orphan	242531243624212	Orphan	0		unknown	NA
FXX162008001536	2008	F	87	■■■■■■■■■■■■□■■■■■■□□□□■■■■■■■■□□□□■■■■■■■	777737607760771	93	LAM5	-243---5-2-4	Orphan	243524242523312	Orphan	0		unknown	NA
FXX162001100039	2001	M	25	■■■■■■■■■■■■□■■■■■■□□□□■■■■■■■■□□□□■■■■■■■	777737607760771	93	LAM5					1		Algeria	P
FXX162007101293	2007	M	72	■■■■■■■■■■■■□■■■■■■□□□□■■■■■■■■□□□□■■■■■■■	777737607760771	93	LAM5	224325182425	1570	243845---------	Orphan	1		unknown	P
FXX162010302193	2010	M	28	■■■■■■■■■■■■■■■■■■■■□□□□■■■■□■■■□□□□■■■□■■■	777777607560731	95	LAM6	-243---6-2-5	Orphan	243625242723312	Orphan	3	SM, INH	unknown	P
FXX162007001368	2007	F	29	■■■■□■■■■■■■■■■■■■■■■■■■■■■■■□■□□□□■■■■■■■	757777777720771	99	H3	-253---5-3-3	Orphan	253533334632343	199	0		unknown	NA
FXX162008001538	2008	F	41	■■■■□■■■■■■■■■■■■■■■■■■■■■■■■□■□□□□■■■■■■■	757777777720771	99	H3	-253---5-3-3	Orphan	253533335732343	363	0		unknown	NA
FXX162006000977	2006	F	32	■■■■□■■■■■■■■■■■■■■■■■■■■■■■■□■□□□□■■■■■■■	757777777720771	99	H3	225313153323	42	253533---------	Orphan	0		France	P
FXX162004100449	2004	M	29	■■■■□■■■■■■■■■■■■■■■■■■■■■■■■□■□□□□■■■■■■■	757777777720771	99	H3	225315153321	1581	253531---------	Orphan	1		France	P
FXX162004100463	2004	F	28	■■■■□■■■■■■■■■■■■■■■■■■■■■■■■□■□□□□■■■■■■■	757777777720771	99	H3	225315153321	1581	253531---------	Orphan	1		France	P
FXX162007101119	2007	M	45	■■■■□■■■■■■■■■■■■■■■■■■■■■■■■□■□□□□■■■■■■■	757777777720771	99	H3	225315153321	1581	253531---------	Orphan	1		unknown	P
FXX162007101192	2007	M	30	■■■■□■■■■■■■■■■■■■■■■■■■■■■■■□■□□□□■■■■■■■	757777777720771	99	H3	22531515332-	Orphan	25353----------	Orphan	1		Romania	P
FXX162003100083	2003	F	32	■■■■■■■□□□□□■■■■■■■■□□□□■■□■■■■■□■■■□□□■■■■	774077606767071	101	AFRI_2					1		Cameroon	EP
FXX162000100340	2000	M	43	■■■■■■■□□□□□■■■■■■■■□□□□■■□■■■■■□■■■□□□■■■■	774077606767071	101	AFRI_2					1		Africa	P
FXX162005100533	2005	F	54	■■■■■■■■□□□■■■■■■■■■□□□□□□□□□□□□□□□□□□□■■■■■	776177400000171	106	Unknown	224226133323	880	242333---------	Orphan	1		Morocco	P
FXX162001000080	2001	M	31	■■■■■■■■□□□■■■■■■■■■□□□□□□□□□□□□□□□□□□□■■■■■	776177400000171	106	Unknown					0		Morocco	P
FXX162006000937	2006	F	21	□□□□■■■■□□■■■■■■■■■■■■■■■■■■■■■■□□□□■■■■■■■	036377777760771	107	S	233325153324	212	333534---------	Orphan	0		unknown	NA
FXX162007101059	2007	M	20	□□□□■■■■□□■■■■■■■■■■■■■■■■■■■■■■□□□□■■■■■■■	036377777760771	107	S	233325153324	212	333534---------	Orphan	1		Congo	P
FXX162010102186	2010	M	20	■□□■■■■■■■■■■■■■■□■■■■■■■■■□□□□■□■■■■■■■■■	477777377413771	109	EAI8-MDG	-433---2-4-3	Orphan	433243643612514	Orphan	1		Madagascar	EP
FXX162010002289	2010	F	45	■□□■■■■■■■■■■■■■■□■■■■■■■■■□□□□■□■■■■■■■■■	477777377413771	109	EAI8-MDG	-443---2-5-3	Orphan	443253643712614	Orphan	0		unknown	NA
FXX162008001471	2008	M	38	■□□■■■■■■■■■■■■■■□■■■■■■■■■□□□□■□■■■■■■■■■	477777377413771	109	EAI8-MDG	-443---2-6-3	Orphan	443263643612616	Orphan	0		unknown	NA
FXX162010002045	2010	F	30	■□□■■■■■■■■■■■■■■□■■■■■■■■■□□□□■□■■■■■■■■■	477777377413771	109	EAI8-MDG	-443---2-5-3	Orphan	443253643612614	Orphan	0		unknown	P
FXX162010102010	2010	F	40	■□□■■■■■■■■■■■■■■□■■■■■■■■■□□□□■□■■■■■■■■■	477777377413771	109	EAI8-MDG	-543---2-2-3	Orphan	543223643512614	Orphan	1		unknown	P
FXX162002100235	2002	M	63	■□□■■■■■■■■■■■■■■□■■■■■■■■■□□□□■□■■■■■■■■■	477777377413771	109	EAI8-MDG					1		France	P
FXX162007001285	2007	M	28	■■■■■■■■■■■■□■■■■■■■■■□□□■■■■■■□□□□■■■■■■■	777737761760771	112	T	223325143322	120	233432---------	Orphan	0		unknown	NA
FXX162007001099	2007	M	25	■■■■■■■■■■■■■■□■■■■■■■■■■■■■■■■■□□□□■■■■■■■	777767777760771	118	T1	225225153322	Orphan	252532---------	Orphan	0		unknown	NA
FXX162002100209	2002	M	78	■■■■■■■■■■■■■■□■■■■■■■■■■■■■■■■■□□□□■■■■■■■	777767777760771	118	T1	222325153222	1553	223522---------	Orphan	1		France	P
FXX162000100272	2000	M	78	■■■■■■■■■■■■■■□■■■■■■■■■■■■■■■■■□□□□■■■■■■■	777767777760771	118	T1					1		France	P
FXX162009001921	2009	M	77	■■■■■■■■■■■■■■■■■□■■■■■■■■■■■■■■□□□□■■■■■■■	777776777760771	119	X1	-233---5-3-3	Orphan	233533243522224	Orphan	0		unknown	NA
FXX162007001335	2007	M	84	■■■■■■■■■■■■■■■■■■■□■■■■■■■■■■■■□□□□■■■■■■■	777777577760771	120	T1	-233---5-3-2	Orphan	233532234502223	Orphan	0		unknown	NA
FXX162007101291	2007	M	79	■■■■■■■■■■■■■■■■■■■□■■■■■■■■■■■■□□□□■■■■■■■	777777577760771	120	T1	223325143322	120	233432---------	Orphan	1		unknown	P
FXX162008001469	2008	M	46	■■■■■■■■■■■■■■■■■■■■■■■■■□■■■■□■□□□□■■■■■■■	777777775720771	121	H3	-252---3-2-1	Orphan	252321333612343	Orphan	0		unknown	NA
FXX162008001732	2008	F	83	■■■■■■■■■■■■■■■■■■■■■■■■■□■■■■□■□□□□■■■■■■■	777777775720771	121	H3	-253---5-3-3	Orphan	253533335432343	Orphan	0		unknown	NA
FXX162006001033	2006	M	36	■■■■■■■■■■■■■■■■■■■■■■■■■■■■■■□□□□□□■■■■■■■	777777777700771	124	Unknown					0		unknown	NA
FXX162001300076	2001	M	23	□□□□□□□□□□□□□□□□□□□□□□□□■■■■■■■■□□□□■■■□■■■	000000007760731	125	LAM3					3	SM, INH	Congo	EP
FXX162008001580	2008	M	38	□□□□□□□□□□□□□□□□□□□□□□□□■■■■■■■■□□□□■■■□■■■	000000007760731	125	LAM3	-233---5-5-2	Orphan	233552463622224	Orphan	0		unknown	NA
FXX162006000854	2006	M	83	■■□□■■■■■■■■■■■■□■■■■■■■■■■■■■■■□□□□■■■□■■□	637775777760730	128	T2-uganda	224315142423	Orphan	243443---------	Orphan	0		unknown	NA
FXX162009001882	2009	M	55	■■□□■■■■■■■■■■■■□■■■■■■■■■■■■■■■□□□□■■■□■■□	637775777760730	128	T2-uganda	-233---4-4-4	Orphan	233444452822325	Orphan	0		unknown	NA
FXX162004100481	2004	F	38	■■□□■■■■■■■■■■■■□■■■■■■■■■■■■■■■□□□□■■■□■■□	637775777760730	128	T2-uganda	222304143323	Orphan	223433---------	Orphan	1		Rwanda	P
FXX162004100499	2004	F	28	■■□□■■■■■■■■■■■■□■■■■■■■■■■■■■■■□□□□■■■□■■□	637775777760730	128	T2-uganda	223305123523	Orphan	233253---------	Orphan	1		Burundi	P
FXX162007101299	2007	F	25	■■□□■■■■■■■■■■■■□■■■■■■■■■■■■■■■□□□□■■■□■■□	637775777760730	128	T2-uganda	223315143323	784	233433---------	Orphan	1		Uganda	P
FXX162001100046	2001	M	43	■■■□□□□□□■■■■■■■■■■■■■□□■■■■□□□□■□■■■■■■■■■	700777747413771	129	EAI6-BGD1					1		Algeria	P
FXX162005100635	2005	F	72	■■■■■■■■□□□■■■■■■■■■■□□□□■■■■■■■■□□□□■■■□■■■	776177607760731	130	LAM3	224316133323	Orphan	243333---------	Orphan	1		unknown	NA

FXX162007001305	2007	M	60	■■■■■■■■□□□■■■■■■■■■□□□□■■■■■■■■□□□□■■■□■■■	776177607760731	130	LAM3	-242---5-3-3	Orphan	242533223524321	Orphan	0		unknown	NA
FXX162008101487	2008	M	50	■■■■■■■■■■■■■■■■■■■■■■■■■■■■■■□■□□□□■■□□■■■	777777777720631	134	H3	-253---5-3-4	Orphan	253534333722243	376	1		unknown	EP
FXX162002100240	2002	F	56	■■■■■■■■■■■■■■■■■■■■■■■■■■■■■■□■□□□□■■□□■■■	777777777720631	134	H3					1		Algeria	EP
FXX162007101114	2007	F	66	■■■■■■■■■■■■■■■■■■■■■■■■■■■■■■□■□□□□■■□□■■■	777777777720631	134	H3	224213152323	1571	242533---------	Orphan	1		Algeria	NA
FXX162008001641	2008	M	56	■■■■■■■■■■■■■■■■■■■■■■■■■■■■■■□■□□□□■■□□■■■	777777777720631	134	H3	-253---5-3-3	Orphan	253533333722243	357	0		unknown	NA
FXX162010002126	2010	F	44	■■■■■■■■■■■■■■■■■■■■■■■■■■■■■■□■□□□□■■□□■■■	777777777720631	134	H3	-253---5-3-3	Orphan	253533333722243	357	0		unknown	NA
FXX162010002206	2010	F	94	■■■■■■■■■■■■■■■■■■■■■■■■■■■■■■□■□□□□■■□□■■■	777777777720631	134	H3	-253---5-3-3	Orphan	253533336722243	359	0		unknown	NA
FXX162009001980	2009	M	44	■■■■■■■■■■■■■■■■■■■■■■■■■■■■■■□■□□□□■■□□■■■	777777777720631	134	H3	-253---5-3-4	Orphan	253534333722243	376	0		unknown	NA
FXX162010002089	2010	F	38	■■■■■■■■■■■■■■■■■■■■■■■■■■■■■■□■□□□□■■□□■■■	777777777720631	134	H3	-253---5-3-4	Orphan	253534332722-43	Orphan	0		unknown	NA
FXX162008001675	2008	M	77	■■■■■■■■■■■■■■■■■■■■■■■■■■■■■■□■□□□□■■□□■■■	777777777720631	134	H3					0		unknown	NA
FXX162005300580	2005	M	61	■■■■■■■■■■■■■■■■■■■■■■■■■■■■■■□■□□□□■■□□■■■	777777777720631	134	H3	225213153323	350	252533---------	Orphan	3	SM	Algeria	P
FXX162002100181	2002	M	45	■■■■■■■■■■■■■■■■■■■■■■■■■■■■■■□■□□□□■■□□■■■	777777777720631	134	H3	225313153323	42	253533---------	Orphan	1		France	P
FXX162005100634	2005	F	71	■■■■■■■■■■■■■■■■■■■■■■■■■■■■■■□■□□□□■■□□■■■	777777777720631	134	H3	225313153323	42	253533---------	Orphan	1		France	P
FXX162005100636	2005	F	93	■■■■■■■■■■■■■■■■■■■■■■■■■■■■■■□■□□□□■■□□■■■	777777777720631	134	H3	225313153323	42	253533---------	Orphan	1		France	P
FXX162005100621	2005	M	42	■■■■■■■■■■■■■■■■■■■■■■■■■■■■■■□■□□□□■■□□■■■	777777777720631	134	H3	225313154324	Orphan	253534---------	Orphan	1		France	P
FXX162008301425	2008	F	89	■■■■■■■■■■■■■■■■■■■■■■■■■■■■■■□■□□□□■■□□■■■	777777777720631	134	H3	-252---5-3-3	Orphan	252533334722243	Orphan	3	SM	unknown	P
FXX162009101840	2009	F	42	■■■■■■■■■■■■■■■■■■■■■■■■■■■■■■□■□□□□■■□□■■■	777777777720631	134	H3	-253---5-3-3	Orphan	253533334733243	Orphan	1		unknown	P
FXX162001000018	2001	F	57	■■■■■■■■■■■■■■■■■■■■■■■■■■■■■■□■□□□□■■□□■■■	777777777720631	134	H3					0		France	P
FXX162000100333	2000	M	73	■■■■■■■■■■■■■■■■■■■■■■■■■■■■■■□■□□□□■■□□■■■	777777777720631	134	H3					1		France	P
FXX162008101376	2008	M	25	■■■■■■■■■■■■■■■■■■■■■■■■■■■■■■□■□□□□■■□□■■■	777777777720631	134	H3	-253---5-3-4	Orphan	253534333722241	Orphan	1		France	P
FXX162008101630	2008	F	73	■■■■■■■■■■■□□□□□■■■□□□□□■□■■■■■■□□□□■□□■■■■	777603405760471	136	LAM5	-243---4-3-1	Orphan	243431336832443	Orphan	1		unknown	EP
FXX162002100224	2002	M	61	■■■■■■■■■■■□□□□□■■■□□□□□■□■■■■■■□□□□■□□■■■■	777603405760471	136	LAM5					1		Guinea	P
FXX162009001852	2009	M	79	■■■■■■■■■■■■■■■■■□■■■■■■■■■■■■■■□□□□■■□□□□■	777776777760601	137	X2	-243---3-3-4	Orphan	243334343434343	Orphan	0		unknown	NA
FXX162008001514	2008	M	42	■■■■■■■■■■■■■■■■■□■■■■■■■■■■■■■■□□□□■■□□□□■	777776777760601	137	X2	-243---5-3-4	Orphan	243534343931426	Orphan	0		unknown	NA
FXX162010102120	2010	M	38	■■■■□■■■■■□□□□□□□□□□□□□□■□□□□□□■□□□□■■■■■■■	757400004020771	143	H1	-237---5-3-3	Orphan	237533333-32343	Orphan	1		Morocco	EP
FXX162007001180	2007	F	39	■■■■□■■■■■□□□□□□□□□□□□□□■□□□□□□■□□□□■■■■■■■	757400004020771	143	H1	224315153323	27	243533---------	Orphan	0		unknown	NA
FXX162007001241	2007	M	29	■■■■□■■■■■□□□□□□□□□□□□□□■□□□□□□■□□□□■■■■■■■	757400004020771	143	H1	224315153323	27	243533---------	Orphan	0		unknown	NA
FXX162008101421	2008	M	78	■■■■□■■■■■□□□□□□□□□□□□□□■□□□□□□■□□□□■■■■■■■	757400004020771	143	H1	-243---3-3-3	Orphan	243333333732333	Orphan	1		unknown	P
FXX162007001306	2007	M	25	■■■■■■□□□□□□□□□□□□□□□□□□□■■■■■■■□□□□■■■■■■■	770000003760771	144	Unknown	-233---5-3-5	Orphan	233535343522226	325	0		unknown	NA
FXX162006000968	2006	F	30	■■■■■■□□□□□□□□□□□□□□□□□□□■■■■■■■□□□□■■■■■■■	770000003760771	144	Unknown					0		unknown	NA
FXX162002300247	2002	M	17	■■■■■■□□□□□□□□□□□□□□□□□□□■■■■■■■□□□□■■■■■■■	770000003760771	144	Unknown					3	SM	Senegal	P
FXX162002100294	2002	M	37	■■■■■■□□□□□□□□□□□□□□□□□□□■■■■■■■□□□□■■■■■■■	770000003760771	144	Unknown					1		Angola	P
FXX162002300296	2002	F	25	■■■■■■■■■□□□□□□□□□□■■■■■■■■■■■■■□□□□■■■■■■■	777000377760771	149	T3-ETH					3	SM, INH	Ethiopia	EP
FXX162008001457	2008	F	85	■■■■■■■■■■■■■■■■■■■■■■■■■■□■□□□□■□■■■■■■■■■	777777776413771	152	EAI5	-642---2-5-3	Orphan	642253742612524	392	0		unknown	NA
FXX162006101016	2006	M	46	■■■■■■■■■■■■■■■■■■■■■■■■■■□■□□□□■□■■■■■■■■■	777777776413771	152	EAI5	273225221434	Orphan	732244---------	Orphan	1		Vietnam	P
FXX162006301072	2006	M	49	■■■■■■■■■■■■■■■■■■■■■■■■■■□■□□□□■□■■■■■■■■■	777777776413771	152	EAI5	364226223431	Orphan	642241---------	Orphan	3	SM	unknown	P
FXX162003100418	2003	M	76	■■■■■■■■■■■■■■■■■■■■■■■■■■□■□□□□■□■■■■■■■■■	777777776413771	152	EAI5					1		Vietnam	P
FXX162007101120	2007	M	57	■■■■■■■■■■■■■■■■■■■■■■■■■■□■□□□□■□■■■■■■■■■	777777776413771	152	EAI5	224225223513	Orphan	242253---------	Orphan	1		China	P
FXX162005000716	2005	F	30	■■■■□■■■■■■■■■■■■■■■■■■■■■■■■■■□□□□■■■□■■■	757777777760731	153	T2	223325143324	112	233434---------	Orphan	0		unknown	NA
FXX162006101045	2006	F	40	■■■■□■■■■■■■■■■■■■■■■■■■■■■■■■■□□□□■■■□■■■	757777777760731	153	T2	223325153324	125	233534---------	Orphan	1		unknown	NA
FXX162004100471	2004	M	33	■■■■□■■■■■■■■■■■■■■■■■■■■■■■■■■□□□□■■■□■■■	757777777760731	153	T2	223325143324	112	233434---------	Orphan	1		France	P
FXX162006000999	2006	M	49	■■■■□■■■■■■■■■■■■■■■■■■■■■■■■■■□□□□■■■■■■■	757777777760771	154	T1	223326154323	1566	233533---------	Orphan	0		unknown	NA
FXX162010102248	2010	M	58	■■■■□■■■■■■■■■■■■■■■■■■■■■■■■■■□□□□■■■■■■■	757777777760771	154	T1	-334---4-2-5	Orphan	334425342522124	Orphan	1		France	P
FXX162003100300	2003	M	32	■■■■□■■■■■■■■■■■■■■■■■■■■■■■■■■□□□□■■■■■■■	757777777760771	154	T1					1		Albania	P
FXX162004100442	2004	M	54	■■■■□■■■■■■■■■■■■■■■■■■■■■■■■■■□□□□■■■■■■■	757777777760771	154	T1					1		Poland	P
FXX162006300772	2006	M	24	■■■■□■■■■■■■■■■■■■■■■■■■■■■■■■■□□□□■■■■■■■	757777777760771	154	T1					3	INH	Romania	P
FXX162006100817	2006	M	31	■■■■□■■■■■■■■■■■■■■■■■■■■■■■■■■□□□□■■■■■■■	757777777760771	154	T1					1		Algeria	P
FXX162010002133	2010	F	60	■■■■■■■■□□□■■■■■■■■■■■■■■■■■■■■□□□□■■■■■■■	776177777760771	156	S	-243---5-3-3	Orphan	243533333733443	349	0		unknown	NA
FXX162010002161	2010	F	72	■■■■■■■■□□□■■■■■■■■■■■■■■■■■■■■□□□□■■■■■■■	776177777760771	156	S	-243---5-3-3	Orphan	243533333733443	349	0		unknown	NA
FXX162010202124	2010	M	50	■■■■■■■■□□□■■■■■■■■■■■■■■■■■■■■□□□□■■■■■■■	776177777760771	156	S	-231---4-3-4	Orphan	231434343522227	Orphan	2	RIF, INH, SM	Georgia	P
FXX162003100308	2003	M	29	■■■■■■■■■■■■■■■■■■■■□□□□■■■■■■■■□□□□■■□■■■■	777777607760671	162	LAM9					1		Algeria	P
FXX162006000858	2006	F	80	■■■■■■■■■■■■■■■■■■■■□□□□■■■■■■■■□□□□■■■□□□□	777777607760700	163	LAM4					0		unknown	NA
FXX162008001704	2008	M	81	■■■■■■■■■■■■■■■■■■■■■■■□□□□□□□■■□□□□■■■■■■■	777777760060771	164	T1	-233---5-3-0	Orphan	233530233622223	Orphan	0		unknown	NA
FXX162010002215	2010	F	92	■■■■■■■■■■■■■■■■■■■■■■■□□□□□□□■■□□□□■■■■■■■	777777760060771	164	T1	-233---6-3-4	Orphan	233634344621223	326	0		unknown	NA
FXX162007101067	2007	F	67	■■■■■■■■■■■■■■■■■■■■■■■■■■■■■□■■□□□□■■■■■■■	777777777660771	167	T1	223425163322	455	234632---------	Orphan	1		unknown	EP
FXX162006001047	2006	M	85	■■■■■■■■■■■■■■■■■■■■■■■■■■■■■□■■□□□□■■■■■■■	777777777660771	167	T1	223325153323	116	233533---------	Orphan	0		unknown	NA
FXX162006000892	2006	F	56	■■■■■■■■■■■■■■■■■■■■■■■■■■■■■□■■□□□□■■■■■■■	777777777660771	167	T1	223325155324	Orphan	233534---------	Orphan	0		unknown	NA

FXX162007001101	2007	F	36	■■■■■■■■■■■■■■■■■■■■■■■■■■■■■□■■□□□□■■■■■■■	777777777660771	167	T1	223425153323	382	234533---------	Orphan	0		unknown	NA
FXX162005100545	2005	M	44	■■■■■■■■■■■■■■■■■■■■■■■■■■■■■□■■□□□□■■■■■■■	777777777660771	167	T1	223325153323	116	233533---------	Orphan	1		France	P
FXX162006101018	2006	M	55	■■■■■■■■■■■■■■■■■■■■■■■■■■■■■□■■□□□□■■■■■■■	777777777660771	167	T1	223325153323	116	233533---------	Orphan	1		Algeria	P
FXX162009101936	2009	F	54	■■■■■■■■■■■■■■■■■■■■■■■■■■■■■□■■□□□□■■■■■■■	777777777660771	167	T1	-234---5-3-3	Orphan	234533243522225	Orphan	1		unknown	P
FXX162000000374	2000	M	53	■■■■■■■■■■■■■■■■■■■■■■■■■■■■■□■■□□□□■■■■■■■	777777777660771	167	T1					0		France	P
FXX162009101744	2009	M	39	■■■■■■■■■■■■■■■■■■■■■■■■■■■■■■■□□□□□■■■■■■■	777777777740771	172	T1	-233---5-3-5	Orphan	233535324522223	Orphan	1		unknown	P
FXX162008001642	2008	F	85	■■■■■■■■■■■■■■■■■■□□□□■■■■■■■■■■□□□□■■■■■■■	777777037760771	174	Unknown	-233---5-3-2	Orphan	233532244322223	Orphan	0		unknown	NA
FXX162009002077	2009	M	72	■■■■■■■■■■■■■■■■■■□□□□■■■■■■■■■■□□□□■■■■■■■	777777037760771	174	Unknown	-234---4-3-3	Orphan	234433242522223	Orphan	0		unknown	NA
FXX162007001327	2007	F	82	■■■■■■■■■■■■■■■■■■□□□□■■■■■■■■■■□□□□■■■■■■■	777777037760771	174	Unknown	-234---6-3-2	Orphan	234632244322223	315	0		unknown	NA
FXX162008001484	2008	M	80	■■■■■■■■■■■■■■■■■■□□□□■■■■■■■■■■□□□□■■■■■■■	777777037760771	174	Unknown	-234---9-3-2	Orphan	234932243322223	Orphan	0		unknown	NA
FXX162009101818	2009	F	50	■■■■■■■■■■■■■■■■■■□□□□■■■■■■■■■■□□□□■■■■■■■	777777037760771	174	Unknown	-234---4-3-2	Orphan	234432244522223	309	1		France	P
FXX162001300075	2001	M	70	■■■■■■■■■■■■■■■■■■□□□□■■■■■■■■■■□□□□■■■■■■■	777777037760771	174	Unknown					3	SM	France	P
FXX162000100141	2000	F	73	■■■■■■■■■■■■■■■■■■□□□□■■■■■■■■■■□□□□■■■■■■■	777777037760771	174	Unknown					1		France	P
FXX162009001824	2009	M	73	■■■■■■■■■■■■■■■■■■■■□■■■■■■■■■■■■□□□□■■■□■■■	777777677760731	175	T2	-243---5-3-1	Orphan	243531233433422	Orphan	0		unknown	NA
FXX162005100611	2005	M	56	□■■■■■■■■■■■■■■■■■■■□□□□■■■■■■■■□□□□■■■■■■■	377777607760771	177	LAM9	224325163323	343	243633---------	Orphan	1		Morocco	EP
FXX162010102011	2010	F	61	□■■■■■■■■■■■■■■■■■■■□□□□■■■■■■■■□□□□■■■■■■■	377777607760771	177	LAM9	-243---7-3-3	Orphan	243733222723222	Orphan	1		unknown	EP
FXX162001000109	2001	F	46	□■■■■■■■■■■■■■■■■■■■□□□□■■■■■■■■□□□□■■■■■■■	377777607760771	177	LAM9					0		France	EP
FXX162001100111	2001	F	48	□■■■■■■■■■■■■■■■■■■■□□□□■■■■■■■■□□□□■■■■■■■	377777607760771	177	LAM9					1		Algeria	EP
FXX162003100313	2003	F	51	□■■■■■■■■■■■■■■■■■■■□□□□■■■■■■■■□□□□■■■■■■■	377777607760771	177	LAM9					1		Algeria	EP
FXX162006100735	2006	F	65	□■■■■■■■■■■■■■■■■■■■□□□□■■■■■■■■□□□□■■■■■■■	377777607760771	177	LAM9					1		Algeria	EP
FXX162005100633	2005	F	54	□■■■■■■■■■■■■■■■■■■■□□□□■■■■■■■■□□□□■■■■■■■	377777607760771	177	LAM9	124326143322	327	243432---------	Orphan	1		Algeria	P
FXX162010101960	2010	M	47	□■■■■■■■■■■■■■■■■■■■□□□□■■■■■■■■□□□□■■■■■■■	377777607760771	177	LAM9	-243---4-3-2	Orphan	243432242833312	Orphan	1		unknown	P
FXX162007001188	2007	M	77	■■□■■■■■■■■■■■■■■■■■■■■■■■■■■□■□□□□■■■■■■■	677777777720771	180	H3	222425153323	445	224533---------	Orphan	0		unknown	NA
FXX162007001351	2007	F	78	■■□■■■■■■■■■■■■■■■■■■■■■■■■■■□■□□□□■■■■■■■	677777777720771	180	H3	-262---5-3-3	Orphan	262533235732244	Orphan	0		unknown	NA
FXX162005000672	2005	M	21	■■□■■■■■■■■■■■■■■■■■■■■■■■■■■□■□□□□■■■■■■■	677777777720771	180	H3					0		unknown	NA
FXX162007101113	2007	M	23	■■□■■■■■■■■■■■■■■■■■■■■■■■■■■□■□□□□■■■■■■■	677777777720771	180	H3	224313153323	240	243533---------	Orphan	1		Algeria	P
FXX162006101056	2006	M	61	■■■■■■□□□■■■■■■■■■■■■■■■■■■■■■■■■■■■□■■■■	770777777777671	181	AFRI_1	236224533422	Orphan	362342---------	Orphan	1		Guinea	EP
FXX162001100063	2001	M	21	■■■■■■□□□■■■■■■■■■■■■■■■■■■■■■■■■■■■□■■■■	770777777777671	181	AFRI_1					1		Guinea	EP
FXX162000000376	2000	F	31	■■■■■■□□□■■■■■■■■■■■■■■■■■■■■■■■■■■■□■■■■	770777777777671	181	AFRI_1					0		Niger	EP
FXX162006001061	2006	M	60	■■■■■■□□□■■■■■■■■■■■■■■■■■■■■■■■■■■■□■■■■	770777777777671	181	AFRI_1	226224243521	1466	262451---------	Orphan	0		unknown	NA
FXX162008001470	2008	F	16	■■■■■■□□□■■■■■■■■■■■■■■■■■■■■■■■■■■■□■■■■	770777777777671	181	AFRI_1	-362---3-4-2	Orphan	362342654432344	Orphan	0		unknown	NA
FXX162009301907	2009	M	28	■■■■■■□□□■■■■■■■■■■■■■■■■■■■■■■■■■■■□■■■■	770777777777671	181	AFRI_1	-272---4-5-1	Orphan	272451754432444	Orphan	3	SM	unknown	P
FXX162002100230	2002	M	26	■■■■■■□□□■■■■■■■■■■■■■■■■■■■■■■■■■■■□■■■■	770777777777671	181	AFRI_1					1		Côte d'Ivoire	P
FXX162002100238	2002	M	25	■■■■■■□□□■■■■■■■■■■■■■■■■■■■■■■■■■■■□■■■■	770777777777671	181	AFRI_1					1		Mauritania	P
FXX162002100260	2002	F	5	■■■■■■□□□■■■■■■■■■■■■■■■■■■■■■■■■■■■□■■■■	770777777777671	181	AFRI_1					1		Guinea	P
FXX162006100950	2006	M	61	■■■■■■□□□■■■■■■■■■■■■■■■■■■■■■■■■■■■□■■■■	770777777777671	181	AFRI_1					1		Senegal	P
FXX162005000739	2005	M	33	■■■■■■■■■■■■■■■■■■□■■■■■■■■■■■■□■□□□□■■■■■■■	777777377720771	183	H3					0		Liberia	EP
FXX162000000404	2000	M	23	■■■■■■□□□■■■■■■■■■■■■■■■■■■■■■■■□■■■■■□■■■■	770777777767671	187	AFRI_1					0		Cambodia	EP
FXX162006000901	2006	M	44	■■■■■■□□□■■■■■■■■■■■■■■■■■■■■■■■□■■■■■□■■■■	770777777767671	187	AFRI_1	236214333422	1589	362342---------	Orphan	0		unknown	NA
FXX162006000938	2006	M	28	■■■■■■□□□■■■■■■■■■■■■■■■■■■■■■■■□■■■■■□■■■■	770777777767671	187	AFRI_1	236214333422	1589	362342---------	Orphan	0		unknown	NA
FXX162007001227	2007	F	32	■■■■■■□□□■■■■■■■■■■■■■■■■■■■■■■■□■■■■■□■■■■	770777777767671	187	AFRI_1	236214333522	359	362352---------	Orphan	0		unknown	NA
FXX162007001302	2007	M	40	■■■■■■□□□■■■■■■■■■■■■■■■■■■■■■■■□■■■■■□■■■■	770777777767671	187	AFRI_1	-362---3-5-2	Orphan	362352454632344	391	0		unknown	NA
FXX162006300920	2006	F	29	■■■■■■□□□■■■■■■■■■■■■■■■■■■■■■■■□■■■■■□■■■■	770777777767671	187	AFRI_1	236214333422	1589	362342---------	Orphan	3	ETH	France	P
FXX162009101948	2009	F	42	■■■■■■□□□■■■■■■■■■■■■■■■■■■■■■■■□■■■■■□■■■■	770777777767671	187	AFRI_1	-362---3-5-2	Orphan	362352454632344	391	1		unknown	P
FXX162003100065	2003	M	34	■■■■■■□□□■■■■■■■■■■■■■■■■■■■■■■■□■■■■■□■■■■	770777777767671	187	AFRI_1					1		Algeria	P
FXX162007101297	2007	F	27	■■■■■■□□□■■■■■■■■■■■■■■■■■■■■■■■□■■■■■□■■■■	770777777767671	187	AFRI_1	2362143305--	Orphan	36235----------	Orphan	1		France	P
FXX162008101441	2008	F	20	■■■■■■□□□■■■■■■■■■■■■■■■■■■■■■■■□■■■■■□■■■■	770777777767671	187	AFRI_1	-362---3-5-2	Orphan	362352454632344	391	1		unknown	P
FXX162006000824	2006	F		□□□□□□□□□□□□□□□□□□□□□□□□□□□□□□□□□□■■■■■□■■■	000000000003731	190	Beijing	123325173632	Orphan	233762---------	Orphan	0		unknown	NA
FXX162010002155	2010	F	76	□□□□□□□□□□□□□□□□□□□□□□□□□□□□□□□□□□■■■■■□■■■	000000000003731	190	Beijing	-233---7-6-2	Orphan	233762455933543	Orphan	0		unknown	NA
FXX162007001217	2007	M	42	■■□■■■■■■■■■■■■■■■■□□□□■■■■■□■■□□□□■■■■■■■	677777607660771	195	LAM1	223226153321	128	232531---------	Orphan	0		unknown	NA
FXX162002100108	2002	F	63	■■□■■■■■■■■■■■■■■■■■■■■■■■■■■■■□□□□■■■■■■■	677777777760771	196	T1					1		Turkey	P
FXX162010102184	2010	M	33	■■■□□□□■■■■■■■■■■■■■■■□□□□□□□□□□□□□■■■■■■■■	703777740001771	203	CAS	-243---8-5-3	Orphan	243853422322423	Orphan	1		Somalia	EP
FXX162010102122	2010	M	26	■■■□■■■■■■■■■■■■■■■■■■■■■■■■■■■■□□□□■■■■■■■	737777777760771	205	T1	-231---5-3-4	Orphan	231534343522225	211	1		Cameroon	EP
FXX162009002067	2009	M	70	■■■□■■■■■■■■■■■■■■■■■■■■■■■■■■■■□□□□■■■■■■■	737777777760771	205	T1	-222---5-3-2	Orphan	222532243522223	Orphan	0		unknown	NA
FXX162008001573	2008	M	76	■■■□■■■■■■■■■■■■■■■■■■■■■■■■■■■■□□□□■■■■■■■	737777777760771	205	T1	-223---5-2-2	Orphan	223522243-23223	Orphan	0		unknown	NA
FXX162006000841	2006	M	49	■■■□■■■■■■■■■■■■■■■■■■■■■■■■■■■■□□□□■■■■■■■	737777777760771	205	T1	-233---4-3-2	Orphan	233432245322224	Orphan	0		unknown	NA
FXX162009001767	2009	M	74	■■■□■■■■■■■■■■■■■■■■■■■■■■■■■■■■□□□□■■■■■■■	737777777760771	205	T1	-233---5-3-4	Orphan	233534345523223	324	0		unknown	NA

FXX162008001707	2008	M	29	■■■□■■■■■■■■■■■■■■■■■■■■■■■■■■■■■■□□□□■■■■■■■	737777777760771	205	T1	-243---5-3-4	Orphan	243534338834446	Orphan	0		unknown	NA
FXX162001100072	2001	F	32	■■■■■□■■■■■■■■■■■■■■■■■■■■■■■■□■□□□□■■■■■■■	767777777720771	207	H3					1		Argentina	P
FXX162008001576	2008	F	64	■■■■■■■■□□□□□□■■■■■■□□□□■■■■■■■■□□□□■■■■■■■	776017607760771	209	LAM12-Madrid1	-242---4-3-3	Orphan	242433244422321	Orphan	0		unknown	NA
FXX162009001837	2009	M	48	■■■■■■■■□□□□□■■■■■■■■■■■■■■■■■■■□□□□■■■■■■■	776037777760771	210	T1	-223---5-3-2	Orphan	223532243623223	300	0		unknown	NA
FXX162009001992	2009	M	53	■■■■■■■■□□□□□■■■■■■■■■■■■■■■■■■■□□□□■■■■■■■	776037777760771	210	T1	-223---5-3-2	Orphan	223532243623223	300	0		unknown	NA
FXX162010002144	2010	M	41	■■■■■■■■□□□□□■■■■■■■■■■■■■■■■■■■□□□□■■■■■■■	776037777760771	210	T1	-223---5-3-2	Orphan	223532243623223	300	0		unknown	NA
FXX162006000877	2006	M	44	■■■■■■■■□□□■□■■■■■■■□□□□■■■■■■■■□□□□■■■■■■■	776137607760771	211	LAM3	224226152323	1574	242533---------	Orphan	0		unknown	NA
FXX162006000972	2006	M	30	■■■■■■■■□□□■□■■■■■■■□□□□■■■■■■■■□□□□■■■■■■■	776137607760771	211	LAM3	224226152323	1574	242533---------	Orphan	0		unknown	NA
FXX162006000800	2006	M	53	■■■■■■■■□□□■□■■■■■■■□□□□■■■■■■■■□□□□■■■■■■■	776137607760771	211	LAM3	224326152323	226	243533---------	Orphan	0		unknown	NA
FXX162006000880	2006	M	64	■■■■■■■■□□□■□■■■■■■■□□□□■■■■■■■■□□□□■■■■■■■	776137607760771	211	LAM3	224326152323	226	243533---------	Orphan	0		unknown	NA
FXX162006000952	2006	F	43	■■■■■■■■□□□■□■■■■■■■□□□□■■■■■■■■□□□□■■■■■■■	776137607760771	211	LAM3	224326152323	226	243533---------	Orphan	0		unknown	NA
FXX162007001287	2007	M	37	■■■■■■■■□□□■□■■■■■■■□□□□■■■■■■■■□□□□■■■■■■■	776137607760771	211	LAM3	224326152323	226	243533---------	Orphan	0		unknown	NA
FXX162008001506	2008	F	29	■■■■■■■■□□□■□■■■■■■■□□□□■■■■■■■■□□□□■■■■■■■	776137607760771	211	LAM3	-243---5-3-3	Orphan	243533243624321	339	0		unknown	NA
FXX162008001643	2008	F	41	■■■■■■■■□□□■□■■■■■■■□□□□■■■■■■■■□□□□■■■■■■■	776137607760771	211	LAM3	-243---5-3-3	Orphan	243533243-24321	Orphan	0		unknown	NA
FXX162009001856	2009	M	16	■■■■■■■■□□□■□■■■■■■■□□□□■■■■■■■■□□□□■■■■■■■	776137607760771	211	LAM3	-243---5-3-3	Orphan	243533243624321	339	0		unknown	NA
FXX162006100714	2006	F	27	■■■■■■■■□□□■□■■■■■■■□□□□■■■■■■■■□□□□■■■■■■■	776137607760771	211	LAM3	224326152323	226	243533---------	Orphan	1		France	P
FXX162006100738	2006	F	48	■■■■■■■■□□□■□■■■■■■■□□□□■■■■■■■■□□□□■■■■■■■	776137607760771	211	LAM3	224326152323	226	243533---------	Orphan	1		Morocco	P
FXX162006100740	2006	F	29	■■■■■■■■□□□■□■■■■■■■□□□□■■■■■■■■□□□□■■■■■■■	776137607760771	211	LAM3	224326152323	226	243533---------	Orphan	1		France	P
FXX162006000951	2006	M	28	■■■■■■■■□□□■□■■■■■■■□□□□■■■■■■■■□□□□■■■■■■■	776137607760771	211	LAM3	224326152323	226	243533---------	Orphan	0		France	P
FXX162007101129	2007	M	34	■■■■■■■■□□□■□■■■■■■■□□□□■■■■■■■■□□□□■■■■■■■	776137607760771	211	LAM3	224326152323	226	243533---------	Orphan	1		Algeria	P
FXX162006100917	2006	M	29	■■■■■■■■□□□■□■■■■■■■□□□□■■■■■■■■□□□□■■■■■■■	776137607760771	211	LAM3	224326153313	Orphan	243533---------	Orphan	1		Portugal	P
FXX162008101590	2008	M	34	■■■■■■■■□□□■□■■■■■■■□□□□■■■■■■■■□□□□■■■■■■■	776137607760771	211	LAM3	-243---5-3-3	Orphan	243533243624321	339	1		Morocco	P
FXX162008001314	2008	M	71	■■■■■■■■□□□■□■■■■■■■□□□□■■■■■■■■□□□□■■■■■■■	776137607760771	211	LAM3	-243---5-3-3	Orphan	243533243624321	339	0		unknown	P
FXX162008101393	2008	M	51	■■■■■■■■□□□■□■■■■■■■□□□□■■■■■■■■□□□□■■■■■■■	776137607760771	211	LAM3	-243---5-3-3	Orphan	243533243624321	339	1		unknown	P
FXX162010002092	2010	M	78	■■■■■■■■■■■■□■■■■■■■■■■■■■□□□□□□■□□□□■■■■■■■	777737774020771	218	H1	-243---5-3-3	Orphan	243533333832110	Orphan	0		unknown	NA
FXX162008001404	2008	M	71	■■■■■■■■■■■■□■■■■■■■■■■■■■□□□□□□■□□□□■■■■■■■	777737774020771	218	H1	-253---5-3-4	Orphan	253534334732343	375	0		unknown	NA
FXX162008101373	2008	F	33	■■■■■■■■■■■■□■■■■■■■■■■■■■□□□□□□■□□□□■■■■■■■	777737774020771	218	H1	-253---5-3-3	Orphan	253533335632343	358	1		France	P
FXX162007001187	2007	F	75	■■■■■■■■■■■■■■■■■■■■■■■■■■■■□□□□■□■■□■■■■■■	777777777413371	234	EAI5	254225223533	269	542253---------	Orphan	0		unknown	NA
FXX162004100525	2004	M	73	■■■■■■■■■■■■■■■■■■■■■■■■■■■■□□□□■□■■■■■■■■■	777777777413771	236	EAI5	264225223632	Orphan	642262---------	Orphan	1		Cambodia	EP
FXX162009101822	2009	M	50	■■■■■■■■■■■■■■■■■■■■■■■■■■■■□□□□■□■■■■■■■■■	777777777413771	236	EAI5	-742---1-5-3	Orphan	742153741612523	Orphan	1		unknown	EP
FXX162008001715	2008	F	49	■■■■■■■■■■■■■■■■■■■■■■■■■■■■□□□□■□■■■■■■■■■	777777777413771	236	EAI5	-542---2-5-1	Orphan	542251642611624	Orphan	0		unknown	NA
FXX162006000910	2006	F	46	■■■■■■■■■■■■■■■■■■■■■■■■■■■■□□□□■□■■■■■■■■■	777777777413771	236	EAI5					0		unknown	NA
FXX162007101341	2007	M	63	■■■■■■■■■■■■■■■■■■■■■■■■■■■■□□□□■□■■■■■■■■■	777777777413771	236	EAI5	-A42---2-5-2	Orphan	A42252742612524	Orphan	1		Cambodia	P
FXX162007001155	2007	M	82	■■■■■■■■■■■■■■■■■■■■■■■■■■■■■■□□□□□□□□□□□□□	777777777700000	237	Unknown	225325153323	45	253533---------	Orphan	0		unknown	NA
FXX162008001583	2008	M	75	■■■■■■■■■■■■■■■■■■■■■■■■■■■■■■□□□□□□□□□□□□□	777777777700000	237	Unknown	-253---5-3-4	Orphan	253534332532343	Orphan	0		unknown	NA
FXX162004000512	2004	F	86	■■■■■■■■■■■■■■■■■■■■■■■■■■■■■■□□□□□□□□□□□□□	777777777700000	237	Unknown					0		France	P
FXX162005100571	2005	M	74	■■■■■■■■■■■■■■■■■■■■■■■■■■■■■■□□□□□□□□■■■■■	777777777700171	238	Unknown	224225153323	22	242533---------	Orphan	1		France	EP
FXX162009002035	2009	M	65	■■■■■■■■■■■■■■■■■■■■■■■■■■■■■■□□□□□□□□■■■■■	777777777700171	238	Unknown	-233---5-3-1	Orphan	233531242422228	Orphan	0		unknown	NA
FXX162010002145	2010	F	77	■■■■■■■■■■■■■■■■■■■■■■■■■■■■■■□□□□□□□□■■■■■	777777777700171	238	Unknown	-235---5-3-2	Orphan	235532243322223	Orphan	0		unknown	NA
FXX162007301342	2007	F	30	■■■■■■■■■■■■■■■■■■■■■■■■■■■■■■■■□□□□■□□□□■■	777777777760411	241	T1	-233---4-2-4	Orphan	233424323722221	Orphan	3	SM	Angola	EP
FXX162004100522	2004	F	24	■■■■■■■■■■■■■■■■■■■■■■■■■■■■■■■■□□□□■□□□□■■	777777777760411	241	T1					1		Dem Republic Congo	EP
FXX162007101105	2007	F	21	■■■■■■■■■■■■■■■■■■■■■■■■■■■■■■■■□□□□■□□□□■■	777777777760411	241	T1	223323143324	689	233434---------	Orphan	1		Congo	P
FXX162009101746	2009	M	64	■■■■■■■■■■■■■■■■■■■■■■■■■■■■■■■■□□□□■■□□□□□	777777777760600	243	T1	-234---5-3-2	Orphan	234532221522223	Orphan	1		unknown	P
FXX162005100564	2005	F	53	■■■■■■■■■■■■■■■■■■■■■■■■■■■■■■■■□□□□■■□□□□■	777777777760601	244	T1	224325153323	33	243533---------	Orphan	1		unknown	EP
FXX162007001145	2007	F	81	■■■■■■■■■■■■■■■■■■■■■■■■■■■■■■■■□□□□■■□□□□■	777777777760601	244	T1	224325153324	34	243534---------	Orphan	0		unknown	NA
FXX162007001225	2007	M	88	■■■■■■■■■■■■■■■■■■■■■■■■■■■■■■■■□□□□■■□□□□■	777777777760601	244	T1	224326153323	213	243533---------	Orphan	0		unknown	NA
FXX162010002139	2010	M	45	■■■■■■■■■■■■■■■■■■■■■■■■■■■■■■■■□□□□■■□□□□■	777777777760601	244	T1	-233---5-3-3	Orphan	233533342824443	Orphan	0		unknown	NA
FXX162010002146	2010	M	54	■■■■■■■■■■■■■■■■■■■■■■■■■■■■■■■■□□□□■■□□□□■	777777777760601	244	T1	-243---5-3-3	Orphan	243533343631446	Orphan	0		unknown	NA
FXX162006000893	2006	M	44	■■■■■■■■■■■■■■■■■■■■■■■■■■■■■■■■□□□□■■□□□□■	777777777760601	244	T1					0		unknown	NA
FXX162000100139	2000	M	78	■■■■■■■■■■■■■■■■■■■■■■■■■■■■■■■■□□□□■■□□□□■	777777777760601	244	T1					1		Italy	P
FXX162003100431	2003	M	22	■■■■■■■■■■■■■■■■■■■■■■■■■■■■■■■■□□□□■■□□□□■	777777777760601	244	T1					1		France	P
FXX162008301375	2008	F	27	■■■■■■■■■■■■■■■■■■■■■■■■■■■■■■■■□□□□■■□□□□■	777777777760601	244	T1	-243---5-3-3	Orphan	243533343832445	135	3	SM	unknown	P
FXX162009001891	2009	F	63	■■■■■■■■■■■■■■■■■■■■■■■■■■■■■■■■□□□□■■□■■■■	777777777760671	245	T1	-232---5-3-4	Orphan	232534345522224	318	0		unknown	NA
FXX162009001913	2009	M	14	■■■■■■■■■■■■■■■■■■■■■■■■■■■■■■■■□□□□■■□■■■■	777777777760671	245	T1	-232---5-3-4	Orphan	232534345522224	318	0		unknown	NA
FXX162001200030	2001	M	29	■■■■■■■■■■■■■■■■■■■■□■■□□■■■■■■■□□□□■■■■■■■	777777663760771	253	T1	125326153226	1548	253526---------	Orphan	2	SM, RIF, INH, ETH	France	P
FXX162004100446	2004	M	34	■■■■■■■□□■■■■■■■■■■■■■■■■■■□□□■□□□□■■■■■■■	774777777420771	262	Ural-1	216225113223	Orphan	162123---------	Orphan	1		Armenia	P
FXX162004100519	2004	M	34	■■■■■■■□□■■■■■■■■■■■■■■■■■■□□□■□□□□■■■■■■■	774777777420771	262	Ural-1	226125113223	749	261123---------	Orphan	1		Romania	P

FXX162010102012	2010	M	76	■■■■■■■□□■■■■■■■■■■■■■■■■■■■□□□■□□□□■■■■■■■	774777777420771	262	Ural-1	-271---1-2-3	Orphan	271123452633423	Orphan	1		unknown	P
FXX162008001515	2008	M	32	□□□□□□□□□□□□□□□□□□□□□□□□□□□□□□□□□□■■□■■■■■■	000000000003371	265	Beijing	-233---5-5-3	Orphan	233553446524543	Orphan	0		unknown	NA
FXX162010002016	2010	nsp		□□□□□□□□□□□□□□□□□□□□□□□□□□□□□□□□□□■■□■■■■■■	000000000003371	265	Beijing	-233---5-5-3	Orphan	233553446824543	142	0		unknown	P
FXX162010002017	2010	F		□□□□□□□□□□□□□□□□□□□□□□□□□□□□□□□□□□■■□■■■■■■	000000000003371	265	Beijing	-233---5-5-3	Orphan	233553446824543	142	0		unknown	P
FXX162007201336	2007	M	70	□□□□□□□□□□□□□□□□□□□□□□□□□□□□□□□□□□■■□■■■■■■	000000000003371	265	Beijing	-233---5-5-3	Orphan	233553446824543	142	2	SM, INH, RIF, ETH, PZA	unknown	P
FXX162008001418	2008	F	88	□□□□□□□□□□□□□□□□□□□□□□□□□□□□□□□□□□□□■■■■■■■	000000000000771	269	Beijing	-232---6-5-3	Orphan	23265344A-44443	Orphan	0		unknown	NA
FXX162010302257	2010	M	30	■■■■■■■■■■■□□□■■■■■■□□□□■■■■■■■■□□□□■■■■■■■	777617607760771	273	LAM5	-243---5-3-6	Orphan	24353624273-312	Orphan	3	SM	unknown	P
FXX162010002301	2010	M	77	■■■■■■■■■■■■■■■■■■■■■■■■□□□□□□■■□□□□■■■■■■■	777777770060771	276	T1	-234---5-3-2	Orphan	234532243422221	Orphan	0		unknown	NA
FXX162006000845	2006	M	91	■■■■■■■■■■■■■■■■■■■■■■■■■■■■□■■■□□□□■■■□□□□	777777777560700	277	T1					0		unknown	NA
FXX162006000862	2006	M	54	■■■■■■■■■■■■■■■■■■■■■■■■■■■■□■■■□□□□■■■□□□□	777777777560700	277	T1					0		unknown	NA
FXX162010002218	2010	M	63	■■■■■■■■■■■■■■■■□■■■■■■■■■■■■■■□□□□■■■■■■■	777775777760771	281	T1	-233---4-3-5	Orphan	233435246522223	Orphan	0		unknown	NA
FXX162004100437	2004	F	24	■■■■■■■■■■■■■■■■□■■■■■■■■■■■■■■□□□□■■■■■■■	777775777760771	281	T1					1		Tunisia	P
FXX162006300814	2006	M	32	■■■■■■■■■■■■■■■■■■■■■□□□■□□□□□□■□□□□■■■■■■■	777777704020771	283	H1					3	SM, INH	Senegal	EP
FXX162006000878	2006	F	25	■■■■■■■■■■■■■■■■■■■■■□□□■□□□□□□■□□□□■■■■■■■	777777704020771	283	H1	225325153323	45	253533---------	Orphan	0		unknown	NA
FXX162009102003	2009	M	89	■■■■■■■■■■■■■■■■■■■■■□□□■□□□□□□■□□□□■■■■■■■	777777704020771	283	H1	-243---5-3-3	Orphan	243533335732243	Orphan	1		France	NA
FXX162006301010	2006	M	47	■■■■■■■■■■■■■■■■■■■■■□□□■□□□□□□■□□□□■■■■■■■	777777704020771	283	H1	225325153323	45	253533---------	Orphan	3	SM, INH	unknown	P
FXX162005100637	2005	M	37	□□□□■■■■■■■□□■■■■■■■■■■■■■■■■■■■□□□□■■■■■■■	037637777760771	284	T1	123323153322	Orphan	233532---------	Orphan	1		Gabon	EP
FXX162006100835	2006	M	22	□□□□■■■■■■■□□■■■■■■■■■■■■■■■■■■■□□□□■■■■■■■	037637777760771	284	T1	223313133423	Orphan	233343---------	Orphan	1		Yugoslavia	EP
FXX162007001130	2007	M	80	□□□□■■■■■■■□□■■■■■■■■■■■■■■■■■■■□□□□■■■■■■■	037637777760771	284	T1	323--3153332	Orphan	23-532---------	Orphan	0		unknown	NA
FXX162009002076	2009	M	67	□□□□■■■■■■■□□■■■■■■■■■■■■■■■■■■■□□□□■■■■■■■	037637777760771	284	T1	-232---5-3-2	Orphan	232532442321324	Orphan	0		unknown	NA
FXX162009002064	2009	F	64	□□□□■■■■■■■□□■■■■■■■■■■■■■■■■■■■□□□□■■■■■■■	037637777760771	284	T1	-233---5-3-2	Orphan	233532442321324	Orphan	0		unknown	NA
FXX162009001770	2009	M	48	□□□□■■■■■■■□□■■■■■■■■■■■■■■■■■■■□□□□■■■■■■■	037637777760771	284	T1	-233---5-3-2	Orphan	233532453721324	Orphan	0		unknown	NA
FXX162007001349	2007	M	57	■■■■■■■■■■■■■■■■■■■■□■■■■■■■■■■■□□□□■■■■■■■	777777677760771	291	T1	-271---1-3-2	Orphan	271132322931343	Orphan	0		unknown	NA
FXX162006100945	2006	M	26	■■■■■■■■■■■■■■■■■■■■■■■■■■■■■■□■□□□□■■■■□■■	777777777720751	293	H3					1		unknown	P
FXX162010102115	2010	F	34	■□■■■■■■■■■■■■■■■■■■■■■■■■■■■■□■□□□□■■■■■■■	577777777720771	294	H3	-253---5-3-3	Orphan	253533335732343	363	1		Djibouti	EP
FXX162007101307	2007	F	20	■■■■■■■■■■■■■■■■■■■■□□□□□□■■□■■■□□□□■■■■■■■	777777601560771	306	T1	-243---4-2-4	Orphan	243424262723312	Orphan	1		Congo	P
FXX162010302107	2010	F	37	■■□■■■■□□□□□□□□□□■■■■■■■■■■■■□■■■■■■■■□□□□□	674001777677600	308	BOV	-323---5-3-2	Orphan	323532353412342	382	3	PZA (bovis)	Congo	P
FXX162010102113	2010	M	44	■■■■■■■■■■■■■■■■■■■□■■■■□□□□□□□■□□□□■■■□■■■	777777570020731	314	H3	-243---5-3-3	Orphan	243533352422324	350	1		unknown	P
FXX162010102258	2010	F	40	■■■■■■■■■■■■■■■■■■■□■■■■□□□□□□□■□□□□■■■□■■■	777777570020731	314	H3	-243---5-3-3	Orphan	243533352422324	350	1		unknown	P
FXX162008101372	2008	F	64	■■■■■■■■■■■■■■■■■■■■■■■□■□□□□□□■□□□□■■■□■■■	777777764020731	315	H1	-254---5-4-3	Orphan	254543335732333	Orphan	1		unknown	EP
FXX162010002270	2010	M	55	■■■■■■■■■■■■■■■■■■■■■■■□■□□□□□□■□□□□■■■□■■■	777777764020731	315	H1	-243---5-4-3	Orphan	243543335752333	Orphan	0		unknown	NA
FXX162009101797	2009	M	37	■■■■■■■■■■■■■■■■■■■■■■■■□□□□□□□■□□□□■■■□■■■	777777770020731	316	H3	-243---4-3-4	Orphan	243434351421324	Orphan	1		Central African Republic	EP
FXX162001100091	2001	F	23	■■■■■■■■■■■■■■■■■■■■■■■■□□□□□□□■□□□□■■■□■■■	777777770020731	316	H3					1		France	EP
FXX162007001250	2007	M	40	■■■■■■■■■■■■■■■■■■■■■■■■□□□□□□□■□□□□■■■□■■■	777777770020731	316	H3	223325143323	121	233433---------	Orphan	0		unknown	NA
FXX162010101957	2010	F	20	■■■■■■■■■■■■■■■■■■■■■■■■■■■□■■■■□□□□■■■□■■■	777777777360731	317	T2	-24----5---3	Orphan	24-5-3322421323	Orphan	1		unknown	EP
FXX162001100060	2001	F	33	■■■■■■■■■■■■■■■■■■■■■■■■■■■□■■■■□□□□■■■□■■■	777777777360731	317	T2					1		Dem Republic Congo	P
FXX162003000350	2003	M	25	■■■■■■■■■■■■■■■■■■■■■■■■■■■□■■■■□□□□■■■□■■■	777777777360731	317	T2					0		Congo	P
FXX162004100515	2004	F	35	■□■■■■■□□□□□■■■■■■■■□□□□■■■■■■■■■■■■□□□■■■■	574077607777071	319	AFRI_2					1		unknown	P
FXX162007301268	2007	F	25	■■■■■■□□□□□□□□□□■■■■□□□□■■□□■■■■■■■■□□□■■■■	770003606377071	320	AFRI_2	224424243221	348	244421---------	Orphan	3	SM	unknown	EP
FXX162003300302	2003	F	21	■■■■■■□□□□□□□□□□■■■■□□□□■■□□■■■■■■■■□□□■■■■	770003606377071	320	AFRI_2					3	SM	Nigeria	P
FXX162009301752	2009	M	33	■■■■■■□□□■■■■■■■■■■■■■□□□■■■■■■■■■■■■■■□■■■■	770777707777671	326	AFRI_1	-254---4-5-2	Orphan	254452655631643	Orphan	3	SM	Senegal	P
FXX162010102099	2010	M	36	■■■■■■□□□■■■■■■■■■■■■■□□□■■■■■■■■■■■■■■□■■■■	770777707777671	326	AFRI_1	-254---4-5-2	Orphan	254452655633643	Orphan	1		Senegal	P
FXX162004100465	2004	M	26	■■■■■■□□□■■■■■■■■■■■■■□□□■■■■■■■■■■■■■■□■■■■	770777707777671	326	AFRI_1					1		Ghana	P
FXX162006100773	2006	M	77	■□■■■■■■■■■■■■■■■■■■■■■■■■■■■■■■□□□□■■■■■■■	577777777760771	334	T1					1		France	EP
FXX162009001846	2009	F	53	■□■■■■■■■■■■■■■■■■■■■■■■■■■■■■■■□□□□■■■■■■■	577777777760771	334	T1	-223---4-2-4	Orphan	223424344623223	Orphan	0		unknown	NA
FXX162010102201	2010	M	72	■□■■■■■■■■■■■■■■■■■■■■■■■■■■■■■■□□□□■■■■■■■	577777777760771	334	T1	-234---4-3-2	Orphan	234432245522223	310	1		unknown	EP and P
FXX162002100227	2002	M	78	■■■■■■■■■■■■■■■■■■■■□□□□■■■■■■□■□□□□■■■■■■■	777777607720771	335	LAM9					1		France	EP
FXX162007001184	2007	F	91	■■■■■■■■■■■■■■■■■■■■□□□□■■■■■■□■□□□□■■■■■■■	777777607720771	335	LAM9	225314153323	1406	253533---------	Orphan	0		unknown	NA
FXX162006000844	2006	M	93	■■■■■■■■■■■■■■■■■■■■□□□□■■■■■■□■□□□□■■■■■■■	777777607720771	335	LAM9	225315163323	Orphan	253633---------	Orphan	0		unknown	NA
FXX162003300317	2003	M	77	■■■■■■■■■■■■■■■■■■■■□□□□■■■■■■□■□□□□■■■■■■■	777777607720771	335	LAM9					3	SM, INH	France	P
FXX162006100763	2006	M	82	■■■■■■■■■■■■■■■■■■■■□□□□■■■■■■□■□□□□■■■■■■■	777777607720771	335	LAM9					1		France	P
FXX162007001248	2007	M	55	■□□■■■■□□□■■■■■■■■■■■■■■■■■■■□□□□■□■■■■■■■■■	474377777413771	340	EAI5	2-432A223533	Orphan	-432536335512714	Orphan	0		unknown	NA
FXX162006100812	2006	F	38	■□□■■■■□□□■■■■■■■■■■■■■■■■■■■□□□□■□■■■■■■■■■	474377777413771	340	EAI5	22432A223533	Orphan	243253---------	Orphan	1		Côte d'Ivoire	P
FXX162008001502	2008	M	66	■■■■■■■■■■■■□■■■■■■□□□□□■□□■■■■■□□□□■■■■■■■	777737404760771	367	LAM7-TUR	-151---1-3-2	Orphan	151132342734343	298	0		unknown	P
FXX162005000725	2005	M	30	■■■■■■■■■■■■■■■■■■■■■■□□■■■■■■■■□□□□■□□■■■■	777777747760471	370	T1					0		unknown	NA
FXX162005100586	2005	M	25	■■■■■■■■■■■■■■■■■■■■■■■□■■■■■■■■□□□□■■■■■■■	777777767760771	373	T1	223325153323	116	233533---------	Orphan	1		Congo	EP
FXX162007001137	2007	F	63	■■■■■■■■■■■■■■■■■■■■■■■□■■■■■■■■□□□□■■■■■■■	777777767760771	373	T1	223325143324	112	233434---------	Orphan	0		unknown	NA

FXX162006000970	2006	F	83	■■■■■■■■■■■■■■■■■■■■■■■□■■■■■■■■□□□□■■■■■■■	777777767760771	373	T1	223325153422	Orphan	233542---------	Orphan	0		unknown	NA
FXX162010002159	2010	F	84	■■■■■■■■■■■■■■■■■■■■■■■□■■■■■■■■□□□□■■■■■■■	777777767760771	373	T1	-233---5-3-4	Orphan	233534244522223	Orphan	0		unknown	NA
FXX162008001612	2008	F	61	■■■■■■■■■■■■■■■■■■■■■■■□■■■■■■■■□□□□■■■■■■■	777777767760771	373	T1	-233---5-3-4	Orphan	233534345522223	Orphan	0		unknown	NA
FXX162009101908	2009	F	73	■■■■■■■■■■■■■■■■■■■■■■■□■■■■■■■■□□□□■■■■■■■	777777767760771	373	T1	-233---5-3-4	Orphan	233534344422223	Orphan	1		unknown	P
FXX162002100193	2002	F	14	■■■■■■■■■■■■■□□□□■□□□□□■■■■■■■■■□□□□■■■■■■■	777741017760771	379	Unknown					1		Chad	EP
FXX162009001963	2009	M	88	■■■■■■■■■■■■■□□□□■□□□□□■■■■■■■■■□□□□■■■■■■■	777741017760771	379	Unknown	-233---5-2-3	Orphan	233523222623312	302	0		unknown	NA
FXX162009001964	2009	F	81	■■■■■■■■■■■■■□□□□■□□□□□■■■■■■■■■□□□□■■■■■■■	777741017760771	379	Unknown	-233---5-2-3	Orphan	233523222623312	302	0		unknown	NA
FXX162006000886	2006	F	88	■■■■■■■■■■■■■■■■■■■■■■■■■■■■■□□■□□□□■■■■■■■	777777777620771	390	H3					0		unknown	NA
FXX162002100202	2002	F	81	■■■■■■■■■■■■■■■■■■■■■■■■■■■■■□□■□□□□■■■■■■■	777777777620771	390	H3					1		France	P
FXX162002100223	2002	M	63	■■■■■■■■■■■■■□■■■■■■■■■■■■■■■■■■■■□□□□■■■■■■■	777757777760771	393	T1					1		France	EP
FXX162008001714	2008	F	76	■■■■■■■■■■■■■■■■■■■■■■■■■■■■■■■■□□□□■□□□□□□	777777777760400	395	T1	-243---5-2-4	Orphan	243524343634543	Orphan	0		unknown	NA
FXX162009102006	2009	M	59	■■■■■■■■■■■■■■■■■■■■□□□□■■■■■■■■□□□□■■□□■■■	777777607760631	398	LAM4	-243---5-3-2	Orphan	243532242733312	338	1		France	EP
FXX162006000969	2006	M	25	■■■■■■■■■■■■■■■■■■■■□□□□□□□□□□□□□□□□□□□□□□□	777777600000000	402	Unknown					0		unknown	NA
FXX162005000663	2005	F	18	■■■■■■■■■■■■■■■■■■■■■■□□□■■■■■■■□□□□■■■□■■■	777777743760731	403	LAM10-CAM	223315152323	Orphan	233533---------	Orphan	0		unknown	NA
FXX162000100155	2000	F	22	■□□□□□■■■■■■■■■■■■■■■■■■■■■■■■■■□□□□■■■■■■■	407777777760771	411	T1					1		Africa	P
FXX162001100059	2001	M	63	■■■□□□□■■■■■■■■■■■■■■■□□□□□□□□□□□□■■□■■■■■■	703777740003371	428	CAS1-Delhi					1		Albania	P
FXX162001300112	2001	M	40	■■■■□■■■■■■■■■■■■■■■■■■■■■□■■□■□□□□■■■■■■■	757777777320771	433	H3					3	SM	Africa	P
FXX162008001493	2008	M	53	■■■■■■■■■■■■□■■■■■□■■■■■■■■■■■■■□□□□■■■■■■■	777737377760771	442	T	-133---4-3-2	Orphan	133432344322223	Orphan	0		Bulgaria	P
FXX162006000935	2006	F	78	■■■■■■■■■■■■■■□■■■■■■■□□□□□□□□□□□□□□□□□□□□□	777767700000000	450	Unknown					0		unknown	NA
FXX162010002143	2010	F	23	■■■■■■■■■■■■■■■■■■■■■■■■■■■■□□□□■□□□□■■□■■■	777777777410331	459	EAI1-SOM	-2-2---2-5-3	Orphan	2-2253222612524	Orphan	0		unknown	NA
FXX162008101397	2008	F	30	■■■■■■■■■■■■■■■■■■■■■■■■■■■■□□□□■□□□□■■□■■■	777777777410331	459	EAI1-SOM	-642---2-3-3	Orphan	642233732612526	Orphan	1		unknown	P
FXX162009101722	2009	F	48	■■■■■■■■■■■■■■■■■■■■■■■■■■■■□■■■□□□□■■■■■■■	777777777560771	462	T1	-244---5-3-4	Orphan	244534336616443	Orphan	1		unknown	EP
FXX162006001032	2006	F	97	■■■■■■■■■■■■■■■■■■■■■■■■■■■■□■■■□□□□■■■■■■■	777777777560771	462	T1	223325153322	15	233532---------	Orphan	0		unknown	NA
FXX162005000719	2005	F	79	■■■■■■■■■■■■■■■■■■■■■■■■■■■■□■■■□□□□■■■■■■■	777777777560771	462	T1					0		unknown	NA
FXX162010102251	2010	F	54	■■■■■■■■□□■■■■■■■■□■■■■■■■■■■■■■■□□□□■■■■■■■	776377377760771	466	S	-333---5-3-5	Orphan	333535445723123	Orphan	1		unknown	EP
FXX162001000025	2001	F	47	■■■■■■■■□□■■■■■■■■□■■■■■■■■■■■■■■□□□□■■■■■■■	776377377760771	466	S					0		Algeria	EP
FXX162000100266	2000	M	58	■■■■■■■■□□■■■■■■■■□■■■■■■■■■■■■■■□□□□■■■■■■■	776377377760771	466	S					1		Algeria	P
FXX162001100045	2001	F	54	■■□■■■■■□■■■■■■□■■■■□■■■■■■■■■■■■■■■■■□□□□□	676773677777600	481	BOV_1	232324233322	Orphan	323332---------	Orphan	1		Algeria	EP
FXX162005000555	2005	M	4	■■□■■■■■□■■■■■■□■■■■□■■■■■■■■■■■■■■■■■□□□□□	676773677777600	481	BOV_1	232324253322	49	323532---------	Orphan	0		France	EP
FXX162008001448	2008	M	17	■■□■■■■■□■■■■■■□■■■■□■■■■■■■■■■■■■■■■■□□□□□	676773677777600	481	BOV_1	-323---5-3-2	Orphan	323532632412242	Orphan	0		unknown	EP
FXX162010302190	2010	F	73	■■□■■■■■□■■■■■■□■■■■□■■■■■■■■■■■■■■■■■□□□□□	676773677777600	481	BOV_1	-323---5-3-2	Orphan	323532632512342	387	3	PZA (bovis)	Maghreb	EP
FXX162004000514	2004	F	48	■■□■■■■■□■■■■■■□■■■■□■■■■■■■■■■■■■■■■■□□□□□	676773677777600	481	BOV_1					0		unknown	EP
FXX162006000879	2006	F	81	■■□■■■■■□■■■■■■□■■■■□■■■■■■■■■■■■■■■■■□□□□□	676773677777600	481	BOV_1	232324253322	49	323532---------	Orphan	0		unknown	NA
FXX162010002327	2010	F	23	■■□■■■■■□■■■■■■□■■■■□■■■■■■■■■■■■■■■■■□□□□□	676773677777600	481	BOV_1	-323---5-3-2	Orphan	323532532412342	287	0		unknown	NA
FXX162009001766	2009	F	50	■■□■■■■■□■■■■■■□■■■■□■■■■■■■■■■■■■■■■■□□□□□	676773677777600	481	BOV_1	-323---3-3-2	Orphan	323332632512342	Orphan	0		unknown	NA
FXX162009001750	2009	M	26	■■□■■■■■□■■■■■■□■■■■□■■■■■■■■■■■■■■■■■□□□□□	676773677777600	481	BOV_1	-323---5-3-2	Orphan	323532552512242	386	0		unknown	P
FXX162009301806	2009	M	42	■■□■■■■■□■■■■■■□■■■■□■■■■■■■■■■■■■■■■■□□□□□	676773677777600	481	BOV_1	-323---5-3-2	Orphan	323532552512242	386	3	PZA (bovis)	unknown	P
FXX162007001337	2007	M	15	■■□■■■■■□■■■■■■□■■■■□■■■■■■■■■■■■■■■■■□□□□□	676773677777600	481	BOV_1	-323---5-3-2	Orphan	323532632512342	387	0		unknown	P
FXX162008001396	2008	M	42	■■□■■■■■□■■■■■■□■■■■□■■■■■■■■■■■■■■■■■□□□□□	676773677777600	481	BOV_1	-323---5-3-2	Orphan	323532632512342	387	0		unknown	P
FXX162003000408	2003	M	78	■■□■■■■■□■■■■■■□■■■■■■■■■■■■■■■■■■■■■■□□□□□	676773777777600	482	BOV_1	212324253322	281	123532---------	Orphan	0		France	EP
FXX162007301175	2007	M	4	■■□■■■■■□■■■■■■□■■■■■■■■■■■■■■■■■■■■■■□□□□□	676773777777600	482	BOV_1	222224253322	Orphan	222532553500122	Orphan	3	PZA (bovis)	France	EP
FXX162006000839	2006	M	1	■■□■■■■■□■■■■■■□■■■■■■■■■■■■■■■■■■■■■■□□□□□	676773777777600	482	BOV_1	222324253322	5	223532553500122	219	0		France	EP
FXX162007001060	2007	M	3	■■□■■■■■□■■■■■■□■■■■■■■■■■■■■■■■■■■■■■□□□□□	676773777777600	482	BOV_1	222324253322	5	223532553500122	219	0		unknown	EP
FXX162000000153	2000	F	59	■■□■■■■■□■■■■■■□■■■■■■■■■■■■■■■■■■■■■■□□□□□	676773777777600	482	BOV_1	222324252322	Orphan	223532---------	Orphan	0		France	EP
FXX162003100324	2003	M	18	■■□■■■■■□■■■■■■□■■■■■■■■■■■■■■■■■■■■■■□□□□□	676773777777600	482	BOV_1	222324253322	5	223532---------	Orphan	1		France	EP
FXX162006000746	2006	F	2	■■□■■■■■□■■■■■■□■■■■■■■■■■■■■■■■■■■■■■□□□□□	676773777777600	482	BOV_1	222324253322	5	223532---------	Orphan	0		unknown	EP
FXX162006000882	2006	F	16	■■□■■■■■□■■■■■■□■■■■■■■■■■■■■■■■■■■■■■□□□□□	676773777777600	482	BOV_1	222324253322	5	223532---------	Orphan	0		unknown	EP
FXX162000300156	2000	M	38	■■□■■■■■□■■■■■■□■■■■■■■■■■■■■■■■■■■■■■□□□□□	676773777777600	482	BOV_1	222325252322	1555	223532---------	Orphan	3	PZA (bovis)	Morocco	EP
FXX162004000508	2004	M	53	■■□■■■■■□■■■■■■□■■■■■■■■■■■■■■■■■■■■■■□□□□□	676773777777600	482	BOV_1	232324242322	1586	323432---------	Orphan	0		France	EP
FXX162001000014	2001	F	36	■■□■■■■■□■■■■■■□■■■■■■■■■■■■■■■■■■■■■■□□□□□	676773777777600	482	BOV_1	232324251222	1076	323522---------	Orphan	0		Algeria	EP
FXX162002000206	2002	M	55	■■□■■■■■□■■■■■■□■■■■■■■■■■■■■■■■■■■■■■□□□□□	676773777777600	482	BOV_1	232324252322	1321	323532---------	Orphan	0		France	EP
FXX162007301165	2007	F	57	■■□■■■■■□■■■■■■□■■■■■■■■■■■■■■■■■■■■■■□□□□□	676773777777600	482	BOV_1	232324252322	1321	323532---------	Orphan	3	PZA (bovis)	France	EP
FXX162005300744	2005	F	4	■■□■■■■■□■■■■■■□■■■■■■■■■■■■■■■■■■■■■■□□□□□	676773777777600	482	BOV_1	232324253322	49	323532---------	Orphan	3	PZA (bovis)	unknown	EP
FXX162006300787	2006	F	38	■■□■■■■■□■■■■■■□■■■■■■■■■■■■■■■■■■■■■■□□□□□	676773777777600	482	BOV_1	232324253322	49	323532---------	Orphan	3	PZA (bovis)	France	EP
FXX162006001020	2006	M	2	■■□■■■■■□■■■■■■□■■■■■■■■■■■■■■■■■■■■■■□□□□□	676773777777600	482	BOV_1	232324253322	49	323532---------	Orphan	0		unknown	EP
FXX162006001021	2006	F	9	■■□■■■■■□■■■■■■□■■■■■■■■■■■■■■■■■■■■■■□□□□□	676773777777600	482	BOV_1	232324253322	49	323532---------	Orphan	0		unknown	EP
FXX162004300552	2004	M	72	■■□■■■■■□■■■■■■□■■■■■■■■■■■■■■■■■■■■■■□□□□□	676773777777600	482	BOV_1	232-24252322	Orphan	32-532---------	Orphan	3	R PZA (bovis)	France	EP

FXX162007001300	2007	M	2	■■□■■■■■□■■■■■■□■■■■■■■■■■■■■■■■■■■■■■□□□□□	676773777777600	482	BOV_1	2x232425-3-2	Orphan	x23532553500122	294	0		unknown	EP
FXX162008301669	2008	M	76	■■□■■■■■□■■■■■■□■■■■■■■■■■■■■■■■■■■■■■□□□□□	676773777777600	482	BOV_1	-123---5-3-2	Orphan	123532553500122	297	3	INH, PZA (BCG therapy)	France	EP
FXX162008001495	2008	F	45	■■□■■■■■□■■■■■■□■■■■■■■■■■■■■■■■■■■■■■□□□□□	676773777777600	482	BOV_1	-223---5-3-2	Orphan	223532553500122	219	0		unknown	EP
FXX162009001757	2009	F	20	■■□■■■■■□■■■■■■□■■■■■■■■■■■■■■■■■■■■■■□□□□□	676773777777600	482	BOV_1	-223---5-3-2	Orphan	223532553500122	219	0		unknown	EP
FXX162009302004	2009	F	9	■■□■■■■■□■■■■■■□■■■■■■■■■■■■■■■■■■■■■■□□□□□	676773777777600	482	BOV_1	-223---5-3-2	Orphan	223532553500122	219	3	PZA (bovis)	France	EP
FXX162010002058	2010	M	4	■■□■■■■■□■■■■■■□■■■■■■■■■■■■■■■■■■■■■■□□□□□	676773777777600	482	BOV_1	-223---5-3-2	Orphan	223532553500122	219	0		unknown	EP
FXX162010302116	2010	F	1	■■□■■■■■□■■■■■■□■■■■■■■■■■■■■■■■■■■■■■□□□□□	676773777777600	482	BOV_1	-223---5-3-2	Orphan	223532553500122	219	3	PZA (bovis)	unknown	EP
FXX162010302358	2010	F	1	■■□■■■■■□■■■■■■□■■■■■■■■■■■■■■■■■■■■■■□□□□□	676773777777600	482	BOV_1	-223---5-3-2	Orphan	223532553500122	219	3	PZA (bovis)	Turkey	EP
FXX162009301761	2009	F	70	■■□■■■■■□■■■■■■□■■■■■■■■■■■■■■■■■■■■■■□□□□□	676773777777600	482	BOV_1	-323---5-3-2	Orphan	323532254310142	Orphan	3	PZA (bovis)	unknown	EP
FXX162010302195	2010	M	25	■■□■■■■■□■■■■■■□■■■■■■■■■■■■■■■■■■■■■■□□□□□	676773777777600	482	BOV_1	-423---5-3-2	Orphan	423532554510142	Orphan	3	PZA (bovis)	unknown	EP
FXX162009301799	2009	M	29	■■□■■■■■□■■■■■■□■■■■■■■■■■■■■■■■■■■■■■□□□□□	676773777777600	482	BOV_1	-x23---5-3-2	Orphan	x23532553500122	294	3	PZA (bovis)	unknown	EP
FXX162005000673	2005	M	83	■■□■■■■■□■■■■■■□■■■■■■■■■■■■■■■■■■■■■■□□□□□	676773777777600	482	BOV_1	120324252322	Orphan	203532---------	Orphan	0		unknown	NA
FXX162005000762	2005	F	49	■■□■■■■■□■■■■■■□■■■■■■■■■■■■■■■■■■■■■■□□□□□	676773777777600	482	BOV_1	212324253322	281	123532---------	Orphan	0	BCG therapy	unknown	NA
FXX162000000360	2000	M		■■□■■■■■□■■■■■■□■■■■■■■■■■■■■■■■■■■■■■□□□□□	676773777777600	482	BOV_1	222324153322	Orphan	223532---------	Orphan	0		unknown	NA
FXX162007001216	2007	M		■■□■■■■■□■■■■■■□■■■■■■■■■■■■■■■■■■■■■■□□□□□	676773777777600	482	BOV_1	232224232322	Orphan	322332---------	Orphan	0		unknown	NA
FXX162006000974	2006	F	66	■■□■■■■■□■■■■■■□■■■■■■■■■■■■■■■■■■■■■■□□□□□	676773777777600	482	BOV_1	232324232322	Orphan	323332---------	Orphan	0		unknown	NA
FXX162006000874	2006	F	79	■■□■■■■■□■■■■■■□■■■■■■■■■■■■■■■■■■■■■■□□□□□	676773777777600	482	BOV_1	232324252322	1321	323532---------	Orphan	0		unknown	NA
FXX162006001031	2006	F	82	■■□■■■■■□■■■■■■□■■■■■■■■■■■■■■■■■■■■■■□□□□□	676773777777600	482	BOV_1	232324252422	1585	323542---------	Orphan	0		unknown	NA
FXX162005000731	2005	F	21	■■□■■■■■□■■■■■■□■■■■■■■■■■■■■■■■■■■■■■□□□□□	676773777777600	482	BOV_1	232324253322	49	323532---------	Orphan	0		unknown	NA
FXX162006001034	2006	M	85	■■□■■■■■□■■■■■■□■■■■■■■■■■■■■■■■■■■■■■□□□□□	676773777777600	482	BOV_1	242324242322	Orphan	423432---------	Orphan	0		unknown	NA
FXX162007001313	2007	M	74	■■□■■■■■□■■■■■■□■■■■■■■■■■■■■■■■■■■■■■□□□□□	676773777777600	482	BOV_1	-123---5-3-2	Orphan	123532553500122	297	0	BCG therapy	unknown	NA
FXX162010002090	2010	F	77	■■□■■■■■□■■■■■■□■■■■■■■■■■■■■■■■■■■■■■□□□□□	676773777777600	482	BOV_1	-323---6-3-2	Orphan	323632452510B42	Orphan	0		unknown	NA
FXX162008001446	2008	M	88	■■□■■■■■□■■■■■■□■■■■■■■■■■■■■■■■■■■■■■□□□□□	676773777777600	482	BOV_1	-321---5-3-2	Orphan	321532553400142	Orphan	0		unknown	NA
FXX162008001513	2008	F	61	■■□■■■■■□■■■■■■□■■■■■■■■■■■■■■■■■■■■■■□□□□□	676773777777600	482	BOV_1	-323---5-3-2	Orphan	323532553510142	383	0		unknown	NA
FXX162008001577	2008	F	79	■■□■■■■■□■■■■■■□■■■■■■■■■■■■■■■■■■■■■■□□□□□	676773777777600	482	BOV_1	-323---5-3-2	Orphan	323532552510142	384	0		unknown	NA
FXX162008001738	2008	F	43	■■□■■■■■□■■■■■■□■■■■■■■■■■■■■■■■■■■■■■□□□□□	676773777777600	482	BOV_1	-323---5-3-2	Orphan	323532552510142	384	0		unknown	NA
FXX162009001789	2009	M	89	■■□■■■■■□■■■■■■□■■■■■■■■■■■■■■■■■■■■■■□□□□□	676773777777600	482	BOV_1	-323---5-3-2	Orphan	323532553510142	383	0		unknown	NA
FXX162009001989	2009	F	94	■■□■■■■■□■■■■■■□■■■■■■■■■■■■■■■■■■■■■■□□□□□	676773777777600	482	BOV_1	-323---5-3-2	Orphan	323532554510142	Orphan	0		unknown	NA
FXX162008001703	2008	M	85	■■□■■■■■□■■■■■■□■■■■■■■■■■■■■■■■■■■■■■□□□□□	676773777777600	482	BOV_1	-323---5-4-2	Orphan	323542553410142	Orphan	0		unknown	NA
FXX162008001705	2008	F	29	■■□■■■■■□■■■■■■□■■■■■■■■■■■■■■■■■■■■■■□□□□□	676773777777600	482	BOV_1	-3-----5-3-2	Orphan	3--532254310142	Orphan	0		unknown	NA
FXX162008001518	2008	F	55	■■□■■■■■□■■■■■■□■■■■■■■■■■■■■■■■■■■■■■□□□□□	676773777777600	482	BOV_1	-3-3---5-2-2	Orphan	3-3522-52-10-42	Orphan	0		unknown	NA
FXX162004000489	2004	M	90	■■□■■■■■□■■■■■■□■■■■■■■■■■■■■■■■■■■■■■□□□□□	676773777777600	482	BOV_1	222314353322	Orphan	223532---------	Orphan	0		France	P
FXX162008301422	2008	F	72	■■□■■■■■□■■■■■■□■■■■■■■■■■■■■■■■■■■■■■□□□□□	676773777777600	482	BOV_1	-313---5-3-2	Orphan	313532453610142	Orphan	3	PZA (bovis)	unknown	P
FXX162010302262	2010	M	29	■■□■■■■■□■■■■■■□■■■■■■■■■■■■■■■■■■■■■■□□□□□	676773777777600	482	BOV_1	-323---5-4-2	Orphan	323542752510142	Orphan	3	PZA (bovis)	unknown	P
FXX162007001172	2007	M	1	■■□■■■■■□■■■■■■□■■■■■■■■■■■■■■■■■■■■■■□□□□□	676773777777600	482	BOV_1	222324253322	5	223532---------	Orphan	0		France	P
FXX162010102332	2010	M	48	■■■■■□□□□■■■■■■■■■■■■■■■■□□□□□□■□□□□■■■■■■■	760777774020771	488	H1	-233---4-3-5	Orphan	233435334732543	Orphan	1		unknown	NA
FXX162006100884	2006	M	41	■■■■■□□□□■■■■■■■■■■■■■■■■□□□□□□■□□□□■■■■■■■	760777774020771	488	H1	223325143325	118	233435---------	Orphan	1		France	P
FXX162006100741	2006	M	54	■■■■■□□□□■■■■■■■■■■■■■■■■□□□□□□■□□□□■■■■■■■	760777774020771	488	H1					1		France	P
FXX162007001171	2007	F	44	■■■■■□□□□■■■■■■■■■■■■■■■■□□□□□□■□□□□■■■■■■■	760777774020771	488	H1	223325143323	121	233433---------	Orphan	0		France	P
FXX162010102199	2010	F	68	■■■■■■■■■■■□■■■■■■■■■■■■■■■■■■■■□□□□■■■■■■■	777677777760771	498	T1	-123---5-3-3	Orphan	123533344622322	Orphan	1		unknown	EP
FXX162010002160	2010	F	71	■■■■■■■■■■■□■■■■■■■■■■■■■■■■■■■■□□□□■■■■■■■	777677777760771	498	T1	-233---5-3-3	Orphan	233533243522222	Orphan	0		unknown	NA
FXX162007001356	2007	F	29	■■■■■■■■■■■■□■■■■■■■■□■■■■■■■■■■□□□□■■■■■■■	777737737760771	504	T3	-233---4-3-3	Orphan	233433443322324	Orphan	0		unknown	NA
FXX162002100251	2002	F	65	■■■■■■■■■■■■■■■■■■■■□□□□■□■■■■■■□□□□■■■■■■■	777777605760771	509	LAM9					1		Algeria	EP
FXX162008001685	2008	M	33	■■■■■■■■■■■■■■■■■■■■■□□□□□□□□□□■□□□□■■■■■■■	777777700020771	511	H3	-253---5-3-3	Orphan	253533336732343	Orphan	0		unknown	NA
FXX162004100450	2004	M	50	■■■■■■■■■■■■■■■■■■■■■□□□□□□□□□□■□□□□■■■■■■■	777777700020771	511	H3					1		Poland	P
FXX162002100184	2002	F	75	■■■■■■■■■■■■■■■■■■■■■□□□■■■■■■□■□□□□■■■■■■■	777777707720771	512	H3					1		France	EP
FXX162008001475	2008	F	37	■■■■■■■■■■■■■■■■■■■■■□□□■■■■■■□■□□□□■■■■■■■	777777707720771	512	H3	-253---5-3-2	Orphan	253532338722243	Orphan	0		unknown	NA
FXX162010302060	2010	M	47	■■■■■■■■■■■■■■■■■■■■■□□□■■■■■■□■□□□□■■■■■■■	777777707720771	512	H3	-253---5-3-3	Orphan	253533334732343	369	3	SM	unknown	P
FXX162006300686	2006	M	49	■■■■■■■■■■■■■■■■■■■■■□□□■■■■■■□■□□□□■■■■■■■	777777707720771	512	H3					3	SM	Bosnia and Herzegovina	P
FXX162009101810	2009	M	19	■■■■■■■■■■■■■■■■■■■■■■■■■■■■■■■■□□□□■■□□□■■	777777777760611	521	T1	-254---5-3-2	Orphan	254532221522224	Orphan	1		Angola	P
FXX162006100818	2006	M	73	■■■■■■■■■■■■■■■■■■■■■■■■■■■■■■■■□□□□■■□□□■■	777777777760611	521	T1					1		France	EP and P
FXX162001100068	2001	F	60	■■■■■■■■■■■■■■■■■■■■■■■■■■■■■■■■□□□□■■■■■■□	777777777760770	522	T1					1		France	EP
FXX162007101323	2007	F	72	■■■□■■■■■■■■■■■■■■■■■■■■■□□□□□□■□□□□■■■■■■■	737777774020771	531	H1	-253---5-3-3	Orphan	253533335722-44	Orphan	1		France	EP
FXX162006001049	2006	M	70	■■■■■■■■■■■■■■■■■■■■■□□□■■■■■■■■□□□□■■■■■■■	777777707760771	535	T1					0		unknown	NA
FXX162008101399	2008	M	40	■■■■■■■■■□□□□□□□□□□□□□□□□□□□□□□□□□□□■■■■■■	777000000000371	560	Unknown	-244---5-2-4	Orphan	244524222623112	Orphan	1		unknown	P
FXX162005100628	2005	F	28	■■■■■■■■■■■■■■■■□■■■■■■■■■■■■■■□□□□■■■□■■■	777775777760731	584	T2	221325133322	Orphan	213332---------	Orphan	1		Somalia	P
FXX162003100434	2003	F	65	■■■■■■■■□□□■■■□□□■□□□□□□□□□□□□□□□□□□□□□■■■■	776161000000071	589	Unknown	-243---3-3-4	Orphan	243334243624221	335	1		Maghreb	EP

FXX162010102256	2010	M	22	■■■■■■■■□□□■■■□□□■□□□□□□□□□□□□□□□□□□□□□■■■■	776161000000071	589	Unknown	-243---3-3-4	Orphan	243334243522221	Orphan	1		Cameroon	EP
FXX162010002339	2010	M	30	■■■■■■■■□□□■■■□□□■□□□□□□□□□□□□□□□□□□□□□■■■■	776161000000071	589	Unknown	-243---3-3-3	Orphan	243333244722221	Orphan	0		unknown	NA
FXX162006000960	2006	M	85	■■■■■■■■□□□■■■□□□■□□□□□□□□□□□□□□□□□□□□□■■■■	776161000000071	589	Unknown	-243---3-3-4	Orphan	243334243524221	336	0		unknown	NA
FXX162008001413	2008	M	78	■■■■■■■■□□□■■■□□□■□□□□□□□□□□□□□□□□□□□□□■■■■	776161000000071	589	Unknown	-243---3-3-4	Orphan	243334243624221	335	0		unknown	NA
FXX162008001570	2008	M	76	■■■■■■■■□□□■■■□□□■□□□□□□□□□□□□□□□□□□□□□■■■■	776161000000071	589	Unknown	-243---3-3-4	Orphan	243334243624221	335	0		unknown	NA
FXX162009001788	2009	M	47	■■■■■■■■□□□■■■□□□■□□□□□□□□□□□□□□□□□□□□□■■■■	776161000000071	589	Unknown	-243---3-3-4	Orphan	243334243524221	336	0		unknown	NA
FXX162008101461	2008	F	15	■■■■■■■■□□□■■■□□□■□□□□□□□□□□□□□□□□□□□□□■■■■	776161000000071	589	Unknown	-243---3-3-4	Orphan	243334243624221	335	1		unknown	P
FXX162008101390	2008	M	17	■■■■■■■■□□□■■■□□□■□□□□□□□□□□□□□□□□□□□□□■■■■	776161000000071	589	Unknown	-243---3-3-4	Orphan	243334243624221	335	1		unknown	P
FXX162004100472	2004	M	50	■■■■■■■■■■■■■■■■■■■■■■□■■■■■□□□□■□■■■■■■■■■	777777757413771	591	EAI6-BGD1					1		India	EP
FXX162001100051	2001	M	23	■□■■■□■■■■■■■■■■■■■■■■■■■■■■■■□□□■□□□□■■■■■■■	567777777420771	597	Ural-2					1		Nepal	P
FXX162008001710	2008	F	63	■■■■■■■■■■■■■■■■■■■■■■■■□□□□□□□□□□□□■■■■■■■	777777770000771	602	Unknown	-233---5-3-2	Orphan	233532345522223	321	0		unknown	NA
FXX162006000806	2006	F	48	■■■■■■■■■■■■■■■■■■■■■■■■□□□□□□□□□□□□■■■■■■■	777777770000771	602	Unknown					0		unknown	NA
FXX162009101795	2009	M	84	■■■■■■■■■■■■■■■■■■■■■■■■□□□□□□□□□□□□■■■■■■■	777777770000771	602	Unknown	-232---5-3-2	Orphan	232532235422223	Orphan	1		unknown	P
FXX162006000909	2006	M	26	■■■■■■■■□□□■■■■■■■■■■■■■□□□□□□□□□□□□□□□□□□□	776177770000000	608	Unknown					0		unknown	NA
FXX162006100922	2006	M	1	■■■■■■■■□□□■■■■■■■■■■■■■□□□□□□□□□□□□□□□□□□□	776177770000000	608	Unknown					1		France	P
FXX162007001265	2007	M	83	■■■■■■■■■■■■■■■■■■■■■■■■■■■■■■■■□□□□■■■■□■■	777777777760751	612	T1	223225153324	169	232534---------	Orphan	0		unknown	NA
FXX162005100551	2005	F	75	■■■■■■■■■■■■□■■■■■■■■■■■■■□■□□□□■□■■■■■■■■■	777737776413771	617	EAI5	265225223433	Orphan	652243---------	Orphan	1		Vietnam	EP
FXX162010002055	2010	M	83	■■■■■■■■■■■■□■■■■■■■■■■■■■□■□□□□■□■■■■■■■■■	777737776413771	617	EAI5	-642---2-5-3	Orphan	642253742612524	392	0		unknown	NA
FXX162006000807	2006	F	35	■■■■■■■■■■■■□■■■■■■■■■■■■■■■□□□□■□■■■■■■■■■	777737777413771	618	EAI5	224225223434	Orphan	242244---------	Orphan	0		unknown	NA
FXX162006000803	2006	M	24	■■■■■■■■■■■■□■■■■■■■■■■■■■■■□□□□■□■■■■■■■■■	777737777413771	618	EAI5	264225223433	Orphan	642243---------	Orphan	0		unknown	NA
FXX162009002031	2009	M	56	■■■■■■■■■■■■■■■■■■□□□□□□□■□□□□□□□□□□□□□□□■■	777777002000011	619	Unknown	-642---2-6-3	Orphan	642263642612524	Orphan	0		unknown	NA
FXX162009001833	2009	F	26	□□□□□□□□□□□□□□□□□□□□□□□□□□□□□□□□□□■□■■■■■■■	000000000002771	621	Beijing	-233---7-4-2	Orphan	233742441824643	Orphan	0		unknown	NA
FXX162001100287	2001	M	63	■■■■■■■■■■■■■■■■■■■■■■■■■■■■■■■■□□□□■■■■■□□	777777777760760	628	T1					1		France	P
FXX162006100918	2006	M	73	□□□□□□□□□□□□□□□□□□□□□□□□□□□□□□□□□□■■■□■■■■■	000000000003571	632	Beijing					1		Cambodia	P
FXX162000300279	2000	F	26	□■□□□□□□□□□□□□□□■■■■■■■■■■■□■□□□□■■■■■□□□□□	200003777207600	646	BOV_4-CAPRAE					3	PZA (caprae)	France	P
FXX162010002147	2010	F	66	■■□□□□□■□■■■■■■□■■■■■■■■■■■■■■■■■■■■■■□□□□□	602773777777600	661	BOV_1	-323---5-3-2	Orphan	323532545510142	Orphan	0		unknown	NA
FXX162004300521	2004	M		■■□□□■■■□■■■■■■□■■■■■■■■■■■■■■■■■■■■■■□□□□□	616773777777600	665	BOV_1	232324234322	Orphan	323332---------	Orphan	3	PZA (bovis)	France	EP
FXX162008301424	2008	M	53	■■□□□■■■□■■■■■■□■■■■■■■■■■■■■■■■■■■■■■□□□□□	616773777777600	665	BOV_1	-323---5-3-2	Orphan	323532353412342	382	3	PZA (bovis)	unknown	EP
FXX162009301760	2009	F	23	■■□□□■■■□■■■■■■□■■■■■■■■■■■■■■■■■■■■■■□□□□□	616773777777600	665	BOV_1	-323---5-4-2	Orphan	323542553510142	Orphan	3	PZA (bovis)	unknown	EP
FXX162007001249	2007	F	61	■■□□□■■■□■■■■■■□■■■■■■■■■■■■■■■■■■■■■■□□□□□	616773777777600	665	BOV_1	232324252422	1585	323542453510142	Orphan	0		unknown	NA
FXX162010002213	2010	F	71	■■□□□■■■□■■■■■■□■■■■■■■■■■■■■■■■■■■■■■□□□□□	616773777777600	665	BOV_1	-323---5-4-2	Orphan	323542553500142	Orphan	0		unknown	NA
FXX162008001466	2008	F	29	■■□□□■■■□■■■■■■□■■■■■■■■■■■■■■■■■■■■■■□□□□□	616773777777600	665	BOV_1	-323---5-3-2	Orphan	323532753512342	Orphan	0		unknown	NA
FXX162006000715	2006	F	15	■■□□□■■■□■■■■■■□■■■■■■■■■■■■■■■■■■■■■■□□□□□	616773777777600	665	BOV_1	232324252422	1585	323542554510141	Orphan	0		Tunisia	P
FXX162009301887	2009	M	35	■■□■■□■□□□□□■■■□■■■■■■■■■■■■■■■■■■■■■■□□□□□	664073777777600	683	BOV_2	-322---5-3-2	Orphan	322532354412342	Orphan	3	PZA (bovis)	unknown	EP
FXX162007301126	2007	F	28	■■□■■□■■□■■■■■■□■■■■□■■■■■■■■■■■■■■■■■□□□□□	666773677777600	684	BOV_1	232324253322	49	323532532412342	287	3	PZA (bovis)	Morocco	EP
FXX162008001666	2008	F	56	■■□■■□■■□■■■■■■□■■■■□■■■■■■■■■■■■■■■■■□□□□□	666773677777600	684	BOV_1	-323---5-3-2	Orphan	323532532412342	287	0		unknown	P
FXX162010102015	2010	M	31	■■□■■■■□□□□□■■■□■■■■■■■■■■■■■■■■■■■■■■□□□□□	674073777777600	685	BOV	-322---6-2-2	Orphan	322622632512342	Orphan	1		Turkey	EP
FXX162005000664	2005	F	84	■■□■■■■■□■■■■■■□■■■■□■■■■■□□□■■■■■■■■■□□□□□	676773676177600	697	BOV_1	232223253322	Orphan	322532---------	Orphan	0		unknown	NA
FXX162009101755	2009	M	34	■■■□□□□□□■■■■■■■□■■■■■□□■■■■□□□□■□■■■■■■■■■	700775747413771	702	EAI6-BGD1	-143---2-5-3	Orphan	14325384-712626	Orphan	1		Togo	P
FXX162009001825	2009	M	71	■■■□■■■■■■■■■■■■■□■■■■■■■■■■■■■■□□□□■■■■■■■	737776777760771	708	X1	-353---5-4-3	Orphan	353543332864445	Orphan	0		unknown	NA
FXX162007001080	2007	F	79	■■■■■□□■■■■■■■■■■■■■■■■■■■■■■■■□□□□■■■■■■■	763777777760771	713	T1	223325153322	15	233532---------	Orphan	0		unknown	NA
FXX162009001917	2009	M	28	■■■■■□□■■■■■■■■■■■■■■■■■■■■■■■■□□□□■■■■■■■	763777777760771	713	T1	-234---2-3-2	Orphan	234232245522223	307	0		unknown	NA
FXX162005100561	2005	M	24	■■■■■□□■■■■■■■■■■■■■■■■■■■■■■■■□□□□■■■■■■■	763777777760771	713	T1	223325123322	1054	233232---------	Orphan	1		France	P
FXX162006100713	2006	M	21	■■■■■□□■■■■■■■■■■■■■■■■■■■■■■■■□□□□■■■■■■■	763777777760771	713	T1	223325123322	1054	233232---------	Orphan	1		France	P
FXX162006100691	2006	M	50	■■■■■□□■■■■■■■■■■■■■■■■■■■■■■■■□□□□■■■■■■■	763777777760771	713	T1	223425123322	1568	234232---------	Orphan	1		France	P
FXX162008101591	2008	M	33	■■■■■□□■■■■■■■■■■■■■■■■■■■■■■■■□□□□■■■■■■■	763777777760771	713	T1	-234---2-3-2	Orphan	234232245522223	307	1		unknown	P
FXX162009101804	2009	F	41	■■■■■■■■□□□■■■■■■■■□□□□□■■■■■■■■□□□□■■■■■■■	776177407760771	719	LAM3	-243---5-3-3	Orphan	243533244724421	127	1		France	P
FXX162005000642	2005	F	82	■■■■■■■■■■■■■■□□□■■■□□□□■■■■■■■■□□□□■■■■■■■	777761607760771	731	LAM9	124326153225	427	243525---------	Orphan	0		unknown	NA
FXX162007001279	2007	F	55	■■■■■■■■■■■■■■□□□■■■□□□□■■■■■■■■□□□□■■■■■■■	777761607760771	731	LAM9	224326163321	375	243631---------	Orphan	0		unknown	NA
FXX162005100624	2005	M	80	■■■■■■■■■■■■■■□□□■■■□□□□■■■■■■■■□□□□■■■■■■■	777761607760771	731	LAM9	223226153221	492	232521---------	Orphan	1		Algeria	EP and P
FXX162009001832	2009	M	48	■■■■■■■■■■■■■■□□■■■■■■■■■■■■■■■■□□□□■■■■■■■	777763777760771	732	T1	-234---6-3-2	Orphan	234632243522223	Orphan	0		unknown	NA
FXX162002100207	2002	F	33	■■■■■■■■■■■■■■□□■■■■■■■■■■■■■■■■□□□□■■■■■■■	777763777760771	732	T1					1		France	P
FXX162005000574	2005	M	58	■■■■■■■■■■■■■■■■■■■■□□□□■■■■■■■■□□□□■□■■■■■	777777607760571	737	LAM9	224226143321	738	242431---------	Orphan	0		Morocco	EP
FXX162009001894	2009	M	79	■■■■■■■■■■■■■■■■■■■■□□□□■■■■■■■■□□□□■□■■■■■	777777607760571	737	LAM9	-232---4-3-1	Orphan	232431244823312	Orphan	0		unknown	NA
FXX162009001879	2009	F	36	■■■■■■■■■■■■■■■■■■■■□□□□■■■■■■■■□□□□■□■■■■■	777777607760571	737	LAM9	-233---5-3-1	Orphan	233531242733313	Orphan	0		unknown	NA
FXX162007001328	2007	F	88	■■■■■■■■■■■■■■■■■■■■□□□□■■■■■■■■□□□□■□■■■■■	777777607760571	737	LAM9	-242---4-3-1	Orphan	242431244822312	Orphan	0		unknown	NA
FXX162010002337	2010	F	34	■■■■■■■■■■■■■■■■■■■■□□□□■■■■■■■■□□□□■□■■■■■	777777607760571	737	LAM9	-323---5-3-2	Orphan	323532632512342	387	0		unknown	NA

FXX162007101294	2007	F	73	■■■■■■■■■■■■■■■■■■■■■■□■■■■■■■□■□□□□■■■■■■■	777777757720771	741	H3	-253---5-3-3	Orphan	253533-34732-43	Orphan	1		unknown	EP
FXX162009301943	2009	M	42	■■■■■■■■■■■■■■■■■■■■■■■■■■■■□■□■□□□□■■■■■■■	777777777520771	746	H3	-263---5-3-3	Orphan	263533336332243	377	3	INH	unknown	EP
FXX162010002163	2010	F	16	■■■■■■■■■■■■■■■■■■■■■■■■■■■■□■□■□□□□■■■■■■■	777777777520771	746	H3	-263---5-3-3	Orphan	263533336332243	377	0		unknown	NA
FXX162007001087	2007	M	31	□□□■■■■■■■■■■■■■■■■■■■■■■■■■■■■■□□□□■■■■■■■	077777777760771	751	T	223216153321	332	232531344322225	226	0		unknown	NA
FXX162008001717	2008	M	65	□□□■■■■■■■■■■■■■■■■■■■■■■■■■■■■■□□□□■■■■■■■	077777777760771	751	T	-233---5-3-1	Orphan	233531242322225	Orphan	0		unknown	NA
FXX162008001546	2008	M	31	□□□■■■■■■■■■■■■■■■■■■■■■■■■■■■■■□□□□■■■■■■■	077777777760771	751	T	-232---5-3-1	Orphan	232531344322225	226	0		unknown	NA
FXX162008001605	2008	M	29	□□□■■■■■■■■■■■■■■■■■■■■■■■■■■■■■□□□□■■■■■■■	077777777760771	751	T	-232---5-3-1	Orphan	232531344322225	226	0		unknown	NA
FXX162000000370	2000	F	16	□□□■■■■■■■■■■■■■■■■■■■■■■■■■■■■■□□□□■■■■■■■	077777777760771	751	T					0		Morocco	P
FXX162006001041	2006	M	30	■■■■■■■□□□□□□■■■■■□□□□□□□□□□□□□■□□□□■■■□□□□	774037000020700	760	Unknown	223222123323	Orphan	232233---------	Orphan	0		unknown	NA
FXX162006100790	2006	M	50	■■■■■■■□□□□□□■■■■■□□□□□□□□□□□□□■□□□□■■■□□□□	774037000020700	760	Unknown					1		France	P
FXX162009001855	2009	F	96	■■■■■■■■■■■■■■□□□□□□□■■■■■■■■■■■□□□□■■■■■■■	777760077760771	765	T1	-233---4-3-4	Orphan	233434346522223	Orphan	0		unknown	NA
FXX162007001185	2007	F	30	■■■■■■■■■■■■■■□□□■□□□□□□■■■■■■■■□□□□■■■■■■■	777761007760771	766	LAM9	124326153223	1219	243523---------	Orphan	0		unknown	NA
FXX162007101071	2007	F	84	■■■■■■■■■■■■■■□□□■□□□□□□■■■■■■■■□□□□■■■■■■■	777761007760771	766	LAM9	222215153222	Orphan	222522---------	Orphan	1		unknown	P
FXX162000300330	2000	F	87	■■■■■■■■■■■■■■■■■■■□□■■■■□□□□□□■□□□□■■■■■■■	777777474020771	769	H1					3	SM	France	EP
FXX162010002263	2010	F	88	■■■■■■■■■■■■■■■■■■■□□■■■■□□□□□□■□□□□■■■■■■■	777777474020771	769	H1	-253---5-3-2	Orphan	253532333332347	Orphan	0		unknown	NA
FXX162008001381	2008	F	86	■■■■■■■■■■■■■■■■■■■■■■■■■■■□□□□□□□□□□■■■■■■	777777777000371	775	Unknown	-222---5-3-3	Orphan	222533343322223	301	0		unknown	NA
FXX162008001588	2008	M	38	■■■■■■■■■■■■■■■■■■■■■■■■■■■□□□□□□□□□□■■■■■■	777777777000371	775	Unknown	-222---5-3-3	Orphan	222533343322223	301	0		unknown	NA
FXX162005000697	2005	M	70	■■■■■■■■■■■■■■■■■■■■■■■■■■■□□□□□□□□□□■■■■■■	777777777000371	775	Unknown					0		unknown	NA
FXX162009001896	2009	M	75	□□□□□□□□□□□□□□□□□□□□□□□□■□□□□□□■□□□□■■■□■■■	000000004020731	781	H2	-253---5-3-3	Orphan	253533335732333	370	0		unknown	NA
FXX162006000897	2006	M	36	■□□□□■■■■■■■■■■■■■■■□□□□■■■■■■■■□□□□■■■■■■■	417777607760771	782	LAM1					0		unknown	NA
FXX162006100985	2006	M	1	■■■■■■■■□□■■■■■■■■■■■■■■■■■■■■■■□□□□■■■□■■■	776377777760731	784	S	123326153321	1242	233531345522226	Orphan	1		France	EP
FXX162006000848	2006	M	56	■■■■■■■■□□■■■■■■■■■■■■■■■■■■■■■■□□□□■■■□■■■	776377777760731	784	S	223326143321	402	233431---------	Orphan	0		unknown	NA
FXX162006000933	2006	M	64	■■■■■■■■□□■■■■■■■■■■■■■■■■■■■■■■□□□□■■■□■■■	776377777760731	784	S	223326153321	224	233531---------	Orphan	0		unknown	NA
FXX162006100832	2006	F	35	■■■■■■■■□□■■■■■■■■■■■■■■■■■■■■■■□□□□■■■□■■■	776377777760731	784	S	123326153321	1242	233531---------	Orphan	1		Morocco	P
FXX162009001785	2009	F	52	■■■■■■■■■■■■■■■■■■■■■■■□□□□□□□□■□□□□■■■■■■■	777777760020771	791	H3	-241---5-3-4	Orphan	241534253622225	Orphan	0		unknown	NA
FXX162004100461	2004	F	58	■■■■■■■■■■■■■■■■■■■■■■■□□□□□□□□■□□□□■■■■■■■	777777760020771	791	H3					1		France	P
FXX162010002187	2010	M	32	■■■■■■■□□■■■■■■■■■■■■■■■■■■■■■■■□□□□■■■■■■■	774777777760771	801	T1					0		unknown	NA
FXX162010002051	2010	M	37	■■■■■■■■■■■■■□□□□□□□□□□□■■■■■■■■□□□□■■■■■■■	777740007760771	803	LAM9	-243---5-2-4	Orphan	243524222623312	140	0		unknown	NA
FXX162002100222	2002	M	25	■□□■■■■■■■■■■■■■■■■■■■■■■■■■■■■■□□□□■■■■■■■	477777777760771	804	T1					1		Côte d'Ivoire	EP
FXX162008001687	2008	M	93	■□□■■■■■■■■■■■■■■■■■■■■■■■■■■■■■□□□□■■■■■■■	477777777760771	804	T1	-233---5-3-2	Orphan	233532244522224	Orphan	0		unknown	NA
FXX162000100282	2000	F	36	■■■■□■■■■■■■■■■■■■■■■■■■■■■□□□□■□■■■■■□■■■	757777777413731	806	EAI1-SOM					1		Cambodia	P
FXX162009302021	2009	M	74	■■□■■■■■□■■■■■□□■■■■■■■■■■■■■■■■■■■■■■□□□□□	676763777777600	820	BOV	-323---5-3-2	Orphan	323532454510142	Orphan	3	PZA (bovis)	unknown	EP
FXX162007101108	2007	F	35	■■■■□■■■■■■■■■■■■■■■□□□□■■■■■■■■□□□□■■■■■■■	757777607760771	822	LAM9	222324173424	Orphan	223744---------	Orphan	1		Morocco	EP
FXX162000100164	2000	F	26	■■■■□■■■■■■■■■■■■■■■□□□□■■■■■■■■□□□□■■■■■■■	757777607760771	822	LAM9					1		Morocco	EP
FXX162007001234	2007	M	71	■■■■□■■■■■■■■■■■■■■■□□□□■■■■■■■■□□□□■■■■■■■	757777607760771	822	LAM9	324326153327	Orphan	243537---------	Orphan	0		unknown	NA
FXX162007001360	2007	M	61	■■■■□■■■■■■■■■■■■■■■□□□□■■■■■■■■□□□□■■■■■■■	757777607760771	822	LAM9	-232---4-3-1	Orphan	232431244804312	Orphan	0		unknown	NA
FXX162007001282	2007	M	45	■■■■■■■■□□□□□□□□□□□□□□□□□■■■■■■■□□□□■■■■■■■	776000003760771	823	T1	124326153226	151	243526---------	Orphan	0		unknown	NA
FXX162003000314	2003	M	72	■■■■■■■■□□■■■■□■■■■■■■■■■■■■■■■■□□□□■■■■■■■	776367777760771	831	S					0		Morocco	NA
FXX162007101324	2007	M	41	■■■■■■■■■■■■■■■■■■■■■■□□□■■■■■■■□□□□■■■■□■■	777777743760751	838	LAM10-CAM	-233---5-3-1	Orphan	23353144552232-	Orphan	1		Cameroon	P
FXX162004100477	2004	M	42	■■■■■■■■■■■■■■■■■■■■■■□□□■■■■■□□□□□□□□■■■■■	777777743700171	839	Unknown					1		Cameroon	EP
FXX162004100436	2004	M	27	■■■■■■■■■□□■■■■■■■■■■■■■■■■■■■□■□□□□■■■■■■■	777177777720771	843	H3					1		France	P
FXX162002100180	2002	F	53	■■■■■□□□■■■■■■■■■■■■■■□□□■■■■■■■□□□□■■■□■■■	761777743760731	844	LAM10-CAM					1		Algeria	EP
FXX162008001501	2008	F	19	■□□□□□□□□□□□□□□□■■■■■■□□□■■■■■■■□□□□■■■■■■■	400003743760771	852	LAM10-CAM	-233---5-3-3	Orphan	23353344542232-	Orphan	0		unknown	P
FXX162002100229	2002	M	72	□■■■■■■■■■■■■■■■■■■■■■■■■■■■■■■□□□□■■■□■■■	377777777760731	853	T2					1		France	P
FXX162009101762	2009	M	61	■□■■■■■■■■■■■■■■■■■■□□□□■■■■■■■■□□□□■■■■■■■	577777607760771	866	LAM9	-243---5-3-6	Orphan	243536242833312	Orphan	1		unknown	EP
FXX162006000770	2006	F	28	■□■■■■■■■■■■■■■■■■■■□□□□■■■■■■■■□□□□■■■■■■■	577777607760771	866	LAM9	124326143323	1545	243433---------	Orphan	0		unknown	NA
FXX162006100823	2006	M	36	■□■■■■■■■■■■■■■■■■■■□□□□■■■■■■■■□□□□■■■■■■■	577777607760771	866	LAM9	124326143323	1545	243433---------	Orphan	1		unknown	P
FXX162003300299	2003	M	39	■□■■■■■■■■■■■■■■■■■■□□□□■■■■■■■■□□□□■■■■■■■	577777607760771	866	LAM9					3	SM	France	P
FXX162008301427	2008	M	52	■■■□■■■■■■■■■■■■■■■■■■■■■■■■■■□■□□□□■■■■■■■	737777777720771	871	H3	-353---5-3-3	Orphan	353533336832343	Orphan	3	SM	unknown	EP
FXX162008101439	2008	F	42	■■■□■■■■■■■■■■■■■■■■■■■■■■■■■■□■□□□□■■■■■■■	737777777720771	871	H3	-253---5-3-3	Orphan	253533336722243	359	1		unknown	P
FXX162002000194	2002	F	38	■■■■■■□■■■■■■■■■■■■■■□□■□□■■■■■■■□□□□■■■■■■■	773777623760771	872	T1					0		Côte d'Ivoire	EP
FXX162002100177	2002	M	22	■■■■■■□■■■■■■■■■■■■■■□□■□□■■■■■■■□□□□■■■■■■■	773777623760771	872	T1					1		Guinea	P
FXX162006100689	2006	F	70	■■■■■■□□□□□□□□□□□□■■■■■■□□□□□□□□□□□□□□□□□□□	770000770000000	877	Unknown					1		France	EP
FXX162010002277	2010	F	49	■■■■■■■■■■■■■■■■■■■■□□□□■■■■■□■■□□□□■■■■■■■	777777607660771	891	LAM9	-233---2-3-4	Orphan	233234243722222	Orphan	0		unknown	NA
FXX162003100419	2003	F	78	■■□■■■■■■■■■■■■■■■■■■■■■■□□□□□□■□□□□■■■■■■■	677777774020771	898	H1					1		France	P
FXX162006000911	2006	F	10	■■■□□□□□□□□□□□□□□□□□□□□□□■■■□□■■■■■■■■■■■■■■	700000007177771	910	Unknown	147322253663	Orphan	473563---------	Orphan	0		unknown	NA
FXX162006100953	2006	F	13	■■■□□□□□□□□□□□□□□□□□□□□□□■■■□□■■■■■■■■■■■■■■	700000007177771	910	Unknown					1		France	P

FXX162009101745	2009	M	70	■■■■■■■■■■■■■■■■■■■■□□□□□□□□□□■■□□□□■■■■■■■	777777600060771	911	Unknown	-233---2-3-4	Orphan	233234243622222	Orphan	1		unknown	P
FXX162005100658	2005	M	46	■■□■■■■■■■■■■■■■■■■■■■■■■■■■□□□□■□■■□■■■■■■	677777777413371	912	EAI5	254225223443	Orphan	542243---------	Orphan	1		Mali	P
FXX162009101842	2009	M	39	■■□■■■■■■■■■■■■■■■■■■■■■■■■■□□□□■□■■□■■■■■■	677777777413371	912	EAI5	-542---2-2-3	Orphan	542223642-12324	Orphan	1		Maghreb	P
FXX162006100830	2006	M	42	■■□■■■■■■■■■■■■■■■■■■■■■■■■■□□□□■□■■□■■■■■■	677777777413371	912	EAI5					1		Laos	P
FXX162008101603	2008	M	77	■■□■■■■■■■■■■■■■■■■■■■■■■■■■□□□□■□■■□■■■■■■	677777777413371	912	EAI5	-542---2-5-3	Orphan	542253642112524	Orphan	1		unknown	P
FXX162009101694	2009	M	41	■■■■■■□□□□□□□□□□□□■■□□□□■■■■■■■■□□□□■■■■■■■	770000607760771	918	Unknown	-131---5-3-4	Orphan	131534344522223	Orphan	1		unknown	P
FXX162002100023	2002	M	29	■■■■■■□□□□□□□□□□□□■■□□□□■■■■■■■■□□□□■■■■■■■	770000607760771	918	Unknown					1		Algeria	P
FXX162008101604	2008	F	83	■□□□■■■■■■■■□■■■■■■■■■■■■■■■□□□■□□□□■■■■■■■	437737777420771	921	Ural-2	-2A2---1-3-2	Orphan	2A2132441833344	Orphan	1		unknown	EP
FXX162009001827	2009	F	96	■■■■■■■■■■■■□□■■■■■■■■■■■■■■■■□■□□□□■■■■■■■	777717777720771	925	H3	-242---5-3-1	Orphan	242531335632343	Orphan	0		unknown	NA
FXX162000100147	2000	M	94	■■■■■■□■■■■■■■■■■■■■■■■■■■■■■■■□□□□■■■■■■■	773777777760771	926	T1					1		France	P
FXX162008101492	2008	F	79	■■■■■■■■■■■■■■■■■■■■■■□■□□□□□□■■□□□□■■■■■■■	777777750060771	928	T	-123---5-3-4	Orphan	123534342522223	Orphan	1		unknown	EP
FXX162002100289	2002	M	84	□□□□□□□□□□□□□□□□□□□□□□□□□□□□□□□□□□■■■■■■□■■	000000000003751	941	Beijing					1		France	P
FXX162007101122	2007	M	59	□□□□□□□□□□□□□□□□□□□□□□□□□□□□□□□□□□■■■■■■□■■	000000000003751	941	Beijing	223325173533	17	233753---------	Orphan	1		Réunion	P
FXX162001300120	2001	M	71	■■■■■■■■■■■■■■■■■■■■■■□■■■■■■■■■■□□□□■■■□■■■	777777757760731	943	T					3	SM	France	EP
FXX162000100121	2000	M	72	■□□□■■■■■■■■■■■■■■■■□□□□■■■■■■■■□□□□■■■■■■■	437777607760771	964	LAM1					1		France	EP
FXX162006100791	2006	M	63	■□□□■■■■■■■■■■■■■■■■□□□□■■■■■■■■□□□□■■■■■■■	437777607760771	964	LAM1					1		Morocco	P
FXX162005100523	2005	F	32	■■■■■■■□■■■■■■■■■■■■■■■■■■■■■■■■□□□□■■■■■■■	775777777760771	966	T1	223325153322	15	233532---------	Orphan	1		France	P
FXX162010002224	2010	F	39	■□□□□□□□□□□□□□□□□□□□□■■■■■■■■■■■■■■■■■□□□□□	400000077777600	980	BOV	-323---6-3-2	Orphan	323632652412342	Orphan	0		unknown	NA
FXX162010002336	2010	M	56	■■□■■■■■□■■■■■■□■■■■□■■■■■■■■■■□□□■■■■□□□□□	676773677743600	1022	BOV_1	-243---5-3-5	Orphan	243535242703313	Orphan	0		unknown	NA
FXX162007001220	2007	M	84	■■□■■■■■□■■■■■■□■■■■■■■■■■■■■■■■□■■□■■□□□□□	676773777766600	1043	BOV_1	232324353322	Orphan	323532---------	Orphan	0		unknown	NA
FXX162005300757	2005	M	72	■■□■■■■■□■■■■■■□■■■■■■■■■■■■■■■■□■■■■■□□□□□	676773777767600	1044	BOV_1					3	PZA (bovis)	unknown	EP
FXX162008001608	2008	M	68	■■□■■■■■□■■■■■■□■■■■■■■■■■■■■■■■□■■■■■□□□□□	676773777767600	1044	BOV_1	-323---4-3-2	Orphan	323432452-12342	Orphan	0		unknown	NA
FXX162003100421	2003	M	41	■■■■■■■■■□■■■■■■■■■■■■■■□□□□□□□■□□□□■■■□■■■	777377770020731	1054	H3					1		Central African Republic	P
FXX162010002081	2010	F	65	■■■■■■■■■■□□■■■■■■■■■■■■■■■■■■■■□□□□■■■□■■■	777477777760731	1056	T2	-232---5-3-4	Orphan	232534433602323	Orphan	0		unknown	NA
FXX162005100559	2005	M	21	■■■■■■■■■■□□■■■■■■■■■■■■■■■■■■■■□□□□■■■□■■■	777477777760731	1056	T2	222325153323	7	223533---------	Orphan	1		Cameroon	P
FXX162010102096	2010	M	50	■■■■■■■■■■□□■■■■■■■■■■■■■■■■■■■■□□□□■■■□■■■	777477777760731	1056	T2	-233---5-3-3	Orphan	233533433422323	Orphan	1		unknown	P
FXX162002100204	2002	F	36	■■■■■■□□■■■■■■■■■■■■□□□□■■■■■■■■□□□□■■■■■■■	771777607760771	1064	LAM9					1		France	EP
FXX162010102191	2010	F	72	■■■■■■□□■■■■■■■■■■■■□□□□■■■■■■■■□□□□■■■■■■■	771777607760771	1064	LAM9	-243---8-3-4	Orphan	243834243522226	Orphan	1		Maghreb	P
FXX162005100532	2005	M	71	■■■■■■■□□□□□□■■■■■■■■□□□■■■■■■■■□□□□■■■□□□□	774037707760700	1067	Unknown	223222153323	9	232533---------	Orphan	1		France	EP
FXX162008001683	2008	M	74	■■■■■■■□□□□□□■■■■■■■■□□□■■■■■■■■□□□□■■■□□□□	774037707760700	1067	Unknown	-232---5-3-3	Orphan	232533342522223	317	0		unknown	NA
FXX162010102246	2010	M	27	■■■■■■■□□□□□□■■■■■■■■□□□■■■■■■■■□□□□■■■□□□□	774037707760700	1067	Unknown	-232---5-3-3	Orphan	232533342522223	317	1		Morocco	P
FXX162010102281	2010	M	75	■■■■■■■■■■■■■■■■■■■■□□□□■■■■■■■■□□□□□■■■■■■	777777607760371	1070	LAM9	-243---7-4-4	Orphan	243744243322225	Orphan	1		Morocco	EP
FXX162002100178	2002	F	65	■■■■■■■■■■■■■■■■■■■■□□□□■■■■■■■■□□□□□■■■■■■	777777607760371	1070	LAM9					1		Algeria	EP
FXX162008001476	2008	F	44	■■■■■■■■■■■■■■■■■■■■□□□□■■■■■■■■□□□□□■■■■■■	777777607760371	1070	LAM9	-243---7-4-4	Orphan	243744243322227	343	0		unknown	NA
FXX162010002290	2010	M	49	■■■■■■■■■■■■■■■■■■■■□□□□■■■■■■■■□□□□□■■■■■■	777777607760371	1070	LAM9	-243---7-4-4	Orphan	243744243322227	343	0		unknown	NA
FXX162006001001	2006	M	34	■■■■■■■■■■■■■■■□□■■■□□□□■■■■■■■■□□□□■■■■■■■	777771607760771	1071	LAM9					0		unknown	NA
FXX162003100304	2003	F	23	■■■■■■■■■■■□□■■■■■■■■■■■■■■■■■■■□□□□■■■■■■■	777637777760771	1073	T1					1		Côte d'Ivoire	P
FXX162009101888	2009	M	76	■■■■■■■■■■■■■■■■■■■■□□□□■■■■■■■■□□□□■■■■■□■	777777607760761	1074	LAM9	-243---6-3-4	Orphan	243634244824323	Orphan	1		unknown	EP
FXX162006000869	2006	M	71	■■■■■■■■■■■■■■■■■■■■□□□□■■■■■■■■□□□□■■■■■□■	777777607760761	1074	LAM9	234323163324	1588	343634---------	Orphan	0		unknown	NA
FXX162007001152	2007	M	24	■■■■■■■■■■■■■■■■■■■■□□□□■■■■■■■■□□□□■■■■■□■	777777607760761	1074	LAM9	244323163425	Orphan	443645244423323	293	0		unknown	NA
FXX162009001922	2009	F	65	■■■■■■■■■■■■■■■■■■■■□□□□■■■■■■■■□□□□■■■■■□■	777777607760761	1074	LAM9	-343---6-3-5	Orphan	343635244523322	Orphan	0		unknown	NA
FXX162008001472	2008	M	41	■■■■■■■■■■■■■■■■■■■■□□□□■■■■■■■■□□□□■■■■■□■	777777607760761	1074	LAM9	-443---6-4-5	Orphan	443645244423323	293	0		unknown	NA
FXX162003100301	2003	F	48	■■■■■■■■■■■■■■■■■■■■□□□□■■■■■■■■□□□□■■■■■□■	777777607760761	1074	LAM9					1		Algeria	P
FXX162009101969	2009	F	74	■■■□□□□■■■■■■■■■■■■■□□□□□□□□□□□□□□■■■■■■■■■	703777600003771	1093	CAS1-Delhi	-255---8-2-3	Orphan	255823422845423	Orphan	1		Pakistan	P
FXX162004100500	2004	M	20	■■■■■□■■■■■■■■■■■■■■■■■■■■■■■■■■□□□□■■■■■■■	767777777760771	1122	T1					1		Romania	P
FXX162009101754	2009	M	78	■■■■■■□■■■■■□■■■■■■■■■■■■□□□□□□■□□□□■■■■■■■	773737774020771	1125	H1	-253---5-3-3	Orphan	253533336722243	359	1		unknown	P
FXX162006000927	2006	M	77	■■■■■■■■□□■■■■■■■■■■■□■■□□□□■■■■□□□□■■■■■■■	776377730360771	1128	S					0		unknown	NA
FXX162010002207	2010	F	73	■■■■■■■■□■■■■■■■■■■■■■■■■■■■■■■□□□□■■■■■■■	776777777760771	1129	T1	-233---5-3-5	Orphan	233535344522221	Orphan	0		unknown	NA
FXX162010002264	2010	F	20	■■■■■■■■□■■■■■■■■■■■■■■■■■■■■■■□□□□■■■■■■■	776777777760771	1129	T1	-243---5-3-3	Orphan	243533333712343	263	0		unknown	NA
FXX162005000695	2005	M	36	■■■■■■■■■□■■■■■■■■■■■■■■■■■■■■□■□□□□■■■□■■■	777377777720731	1135	H3					0		unknown	NA
FXX162006000923	2006	F	70	■■■■■■■■■■■■□□□□■■■■■□■■■■■■■■■■□□□□■■■■■■■	777703737760771	1167	T1					0		unknown	NA
FXX162010102110	2010	F	65	■■■■■■■■■■■■■□■■■■■■□□□□■■■■■■■■□□□□■■■■■■■	777757607760771	1176	LAM9	-232---5-3-1	Orphan	232531243604212	Orphan	1		Algeria	EP
FXX162010002216	2010	M	24	■■■□□□□■■■■■□■■■■■■■■■□□□□□□□□□□□□■■□□■■■■■	703737740003171	1198	CAS1-Delhi	-264---1-5-3	Orphan	264153622743423	Orphan	0		unknown	NA
FXX162001100033	2001	F	56	■■■□□□□■■■■■■■■■■■■■■■□□■■■■■■■■■■■■■■■■■■■	703777747777771	1200	Unknown					1		Algeria	EP
FXX162006100836	2006	M	31	■■■□□□□■■■■■■■■■■■■■■■□□■■■■■■■■■■■■■■■■■■■	703777747777771	1200	Unknown	-296---4-5-3	Orphan	296453322844423	Orphan	1		France	P
FXX162010102109	2010	F	17	■■■■■■□■■■■■■■■■■■■■■■■■■■■■■□■□□□□■■■■■■■	773777777720771	1243	H3	-233---5-3-3	Orphan	233533335732344	Orphan	1		France	P
FXX162010002204	2010	F	82	■■■■■■■■■■■■■■■■■■□□■■■■■■■■■■■■□□□□■■■■■■■	777777177760771	1252	T1	-233---5-3-1	Orphan	233531344522223	319	0		unknown	NA

FXX162006100944	2006	M	84	■■■■■■■■■■■■■■■■■■□□■■■■■■■■■■■■□□□□■■■■■■■	777777177760771	1252	T1					1		France	EP and P
FXX162006001040	2006	M	85	■■□■■■■■□□■■■■■■■■■■■□■■■■■■■■■■□□□□■■■■■■■	676377737760771	1253	S					0		unknown	NA
FXX162001100061	2001	M	23	■■■□□□□■■■■■■■■■■■■■■■□□□□□□□□□□□□□□□□□□□□□	703777740000000	1264	CAS1-Delhi					1		Guinea	EP
FXX162007001278	2007	F	82	□□□□■■■■■■■□■■■■■■■■■■■■■■■■■■■■□□□□■■■■■■■	037677777760771	1278	T1	223325153323	116	233533---------	Orphan	0		unknown	NA
FXX162007001354	2007	M	75	□□□□■■■■■■■□■■■■■■■■■■■■■■■■■■■■□□□□■■■■■■■	037677777760771	1278	T1	-233---5-3-2	Orphan	233532453421326	Orphan	0		unknown	NA
FXX162005100655	2005	F	87	□□□□■■■■■■■□■■■■■■■■■■■■■■■■■■■■□□□□■■■■■■■	037677777760771	1278	T1	223325153322	15	233532---------	Orphan	1		France	P
FXX162006101013	2006	M	38	□□□□■■■■■■■□■■■■■■■■■■■■■■■■■■■■□□□□■■■■■■■	037677777760771	1278	T1	223325153323	116	233533---------	Orphan	1		France	P
FXX162010102125	2010	M	80	■■■■■■■■■■■■■■■■■■■□□□□□□□□□□□□■□□□□■■■■■■■	777777400020771	1282	H3	-232---5-3-1	Orphan	232531243635312	Orphan	1		France	P
FXX162001100088	2001	M	78	■■■■■■■■■■■■■■■■■■■□□□□□□□□□□□□■□□□□■■■■■■■	777777400020771	1282	H3					1		France	P
FXX162006100734	2006	F	21	■■■■□□□□■■■■□□□□■■■■■■■■■■■■■■■■□□□□■■■■■■■	741703777760771	1284	T1					1		Angola	P
FXX162000100275	2000	F	31	■■□■■■■■■■■■■■■■■■■□□□□■■■■■■■■□□□□■■■□■■■	677777607760731	1321	LAM					1		France	P
FXX162009001977	2009	M	41	■■□■■■■■■■■■■■■■■■■■■■■■■■■■■■■□□□□■■■□■■■	677777777760731	1332	T2	-234---4-4-6	Orphan	2344463-2522224	Orphan	0		unknown	NA
FXX162003100303	2003	M	7	■■□■■■■■■■■■■■■■■■■■■■■■■■■■■■■□□□□■■■□■■■	677777777760731	1332	T2	223325153225	916	233525---------	Orphan	1		Central African Republic	P
FXX162003000416	2003	M	6	■■□■■■■■■■■■■■■■■■■■■■■■■■■■■■■□□□□■■■□■■■	677777777760731	1332	T2	223325153225	916	233525---------	Orphan	0		Central African Republic	P
FXX162004000510	2004	F	22	■■□■■■■■■■■■■■■■■■■■■■■■■■■■■■■□□□□■■■□■■■	677777777760731	1332	T2	223325153225	916	233525---------	Orphan	0		Central African Republic	P
FXX162009001765	2009	M	63	□■■■■■■■■■■■□■■■■■■■□□□□■■■■■■■■□□□□■■■■■■■	377737607760771	1367	LAM5	-243---5-3-2	Orphan	243532242833312	Orphan	0		unknown	NA
FXX162000000127	2000	F	64	■■■■■■■■■■■■■■■■■■■■■■■■■■■■□□□□■□■■■□□□■■■	777777777413431	1392	EAI5					0		Cambodia	EP
FXX162001300082	2001	M	38	■■■■■■■■■□■■■■■■■□□□□■■□□□□■■□■■□□□□■■■■■■■	777376060660771	1471	LAM11-ZWE					3	SM, INH	Congo	P
FXX162008001532	2008	M	57	■■■□■■■■□□□■■■■■■■■■■□□□□■■■■■■□□□□□□□□■■■■■	736177607700171	1476	AFRI_2	-242---5-3-3	Orphan	242533224922321	Orphan	0		unknown	NA
FXX162002100248	2002	F	36	■■■■□■■■■■■■■■■■■■■■□□■■■■■■■■■■□□□□■■■■■■■	757777637760771	1514	T1					1		France	P
FXX162000100274	2000	F	56	□■■■■■■■□□□■□■■■■■■■□□□□■■■■■■■■□□□□■■■■■■■	376137607760771	1525	LAM3					1		Algeria	EP
FXX162008001380	2008	F	53	■■■■■■■■■■■■■■■■■■■■□□□□■■■■■■■■□□□□■■■□□■■	777777607760711	1530	LAM4	-243---6-3-1	Orphan	243631243624222	Orphan	0		unknown	NA
FXX162008001701	2008	F	62	■■■■■■■■■■■□□■■■■■■■■■■■■■■■■■■□■□□□□■■■■■■■	777637777720771	1533	H3	-253---5-3-3	Orphan	253533334632342	Orphan	0		unknown	NA
FXX162009101813	2009	F	25	■■■■■■■■■■□■■■■■■■■■□□□□■■■■■■■■□□□□■■■■■■■	777577607760771	1535	LAM9	-231---5-3-1	Orphan	231531244823312	115	1		Congo	EP
FXX162008101333	2008	M	65	■■■■■■■■■■□■■■■■■■■■□□□□■■■■■■■■□□□□■■■■■■■	777577607760771	1535	LAM9	-24----5-3-1	Orphan	24-531244825312	Orphan	1		unknown	EP
FXX162003100256	2003	M	43	■■■■■■■■■■□■■■■■■■■■□□□□■■■■■■■■□□□□■■■■■■■	777577607760771	1535	LAM9					1		Dem Republic Congo	EP
FXX162006001009	2006	F	27	■■■■■■■□□□□■□■■■■■■■□□□□□□□□□□□■□□□□■■■■■■■	774137600020771	1538	Unknown					0		unknown	NA
FXX162009101758	2009	M	37	■■■■■■■□□□□■□■■■■■■■□□□□□□□□□□□■□□□□■■■■■■■	774137600020771	1538	Unknown	-243---5-3-2	Orphan	243532242524321	Orphan	1		unknown	P
FXX162008001726	2008	M	83	■■■■■■■■■■■■■■■■■■■■■■■■■■■■■■■■□□□□■□■□■■■	777777777760531	1544	T2	-253---5-3-3	Orphan	253533332334343	367	0		unknown	NA
FXX162002100190	2002	F	34	■■■■■■■■■■■■□□□□■■■□□□□□□□□□□□□□□□□□□■■■■□□	777703400000360	1548	Unknown					1		Congo	P
FXX162003100428	2003	M	16	■■■■■■■■■■■■□□□□■■■□□□□□□□□□□□□□□□□□□■■■■□□	777703400000360	1548	Unknown					1		Dem Republic Congo	P
FXX162005100595	2005	F	10	■■■■■■■□■■■■■■■■■■■■□□□□■■□□□□■■□□□□■■■□■■■	775777606060731	1549	LAM11-ZWE	224126152321	163	241531---------	Orphan	1		Dem Republic Congo	P
FXX162008001644	2008	M	86	■■■■■■■■■■■■■■■■■■■□□□□□□□□□□□□□□□□□□□■■■■■	777777400000171	1559	Unknown	-242---4-3-1	Orphan	242431234824312	Orphan	0		unknown	NA
FXX162005000694	2005	M	80	■■■■■■■■■■■■■■■■■■■□□□□□□□□□□□□□□□□□□□■■■■■	777777400000171	1559	Unknown					0		unknown	NA
FXX162005100676	2005	M	71	■■■■■□□□□□□□□□□□□□□□□□□□■■■■■■■■□□□□■■■■■■■	760000007760771	1574	LAM3	224325153324	34	243534---------	Orphan	1		France	EP and P
FXX162007101149	2007	F	22	■■■■■□■■■■■■■■■■■■■■■■■■■■■■■■■■□□□□■■■□■■■	767777777760731	1578	T2	223325153323	116	233533---------	Orphan	1		unknown	EP
FXX162003100410	2003	M	27	■■■■■□■■■■■■■■■■■■■■■■■■■■■■■■■■□□□□■■■□■■■	767777777760731	1578	T2					1		Algeria	P
FXX162002300293	2002	M	77	■■■■■■■■■■■■■■■■■■■■■■□□■■■■■■■■□□□□■■■■■■■	777777747760771	1580	T					3	ETH	France	P
FXX162007101124	2007	M	22	■■■■■■■■■■■□□■■■■■■■■■■■■■■■□□□□■□■■■■■■■■■	777637777413771	1609	EAI5	254325223433	403	543243---------	Orphan	1		Cambodia	P
FXX162007001161	2007	F	77	■■■■■■■■■■■■■■■■■■■■■■■■■□□□□□□■□□□□■■■■□■■	777777774020751	1629	H1	225325153323	45	253533---------	Orphan	0		unknown	NA
FXX162000300129	2000	F	75	■■■■■■■■■■■■■■■■■■■■■■■■■■■■■■□■□□□□■■■■□□□	777777777720740	1640	H3					3	SM	France	P
FXX162007101320	2007	M	75	■■■■■■■■■■■■■■■■■■□■■■■■■■■■□□□□□□□□□□■■■■■■	777777377000371	1657	Unknown	-223---3-3-3	Orphan	223333333522223	Orphan	1		France	EP
FXX162008001635	2008	F	92	■■■■■■■■□□□□□□□□□□□□□□□□□□□□□□□□□□□□□□□□□□□	776000000000000	1684	Unknown	-243---5-3-4	Orphan	243534244624421	Orphan	0		unknown	NA
FXX162005100572	2005	F	37	■■■■■■■■■■■■■■■■■■■■■□□□□□□□□□□□□□□□□■■■■■■	777777700000371	1736	Unknown	223325143322	120	233432---------	Orphan	1		Madagascar	EP
FXX162007001197	2007	F	51	■■■■■□■■■■■■■■■■■■■■■■■■■■■■■■□■□□□□■■□□■■■	767777777720631	1743	H3	225213153323	350	252533---------	Orphan	0		unknown	NA
FXX162004100476	2004	F	81	■■■□□□□□□□□□□■■■■■■■■■■■■■■■■■■■□□□□■■■■■■■	700037777760771	1753	Unknown					1		France	EP
FXX162005100592	2005	M	24	■■■■■■■■□□■■■■■■■■■■■■■□□□□□□□□□□□□□□□■■■■■	776377760000171	1776	Unknown	333325153221	Orphan	333521---------	Orphan	1		France	P
FXX162009101763	2009	M	49	■■■■■■■■□□■■■■■■■■■■■■■□□□□□□□□□□□□□□□■■■■■	776377760000171	1776	Unknown	-333---5-2-2	Orphan	333522342823123	Orphan	1		unknown	P
FXX162010002223	2010	M	31	■■■□□□□■■■■■■■■■■■■■■■□□□□□□□□□□□□□□□□■■■■■	703777740000171	1789	CAS	-264---7-5-3	Orphan	264753422622523	378	0		unknown	NA
FXX162008101432	2008	M	19	■■■□□□□■■■■■■■■■■■■■■■□□□□□□□□□□□□□□□□■■■■■	703777740000171	1789	CAS	-264---7-5-3	Orphan	264753422622523	378	1		unknown	P
FXX162007101339	2007	M	44	■■■□□□□■■■■■■■■■■■■■■■□□□□□□□□□□□□□□□□■■■■■	703777740000171	1789	CAS	-264---7-5-3	Orphan	264753422622523	378	1		unknown	P
FXX162001300022	2001	M	56	■□■■■■■■■■■■■■■■■■■■■■□□■□□□□□□■□□□□■■■■■■■	577777744020771	1798	H1					3	SM	Spain	P
FXX162010002100	2010	M	17	■■□□□■■■■■■■■■■■■■■■□□□□■■■■■■■■□□□□■■■■■■■	617777607760771	1803	LAM1	-221---5-2-1	Orphan	221521244622212	Orphan	0		unknown	NA
FXX162001300090	2001	M	50	■■■■■■■■■■■■■■■□■■■■□□□□■■■■■■■■□□□□■■■■■■■	777773607760771	1832	LAM9					3	SM	France	EP
FXX162006000885	2006	F	46	■■■■■■■■■■■■■■■□■■■■□□□□■■■■■■■■□□□□■■■■■■■	777773607760771	1832	LAM9	224226163424	1575	242644---------	Orphan	0		unknown	NA
FXX162000100261	2000	F	20	■■■■■■■■■■■■■■□□■■■■□□□□■■■■■■■■□□□□■■■■■■■	777763607760771	1844	LAM9					1		Morocco	P
FXX162000100339	2000	M	40	■■■□■■■■■□■■■■■■■■■■■■■■■■■■■■□□□□■■■■■■■	737377777760771	1877	T1					1		France	P

FXX162000300166	2000	M	72	■■■■■■■■■■■■□□□□□■■■■■■■■■■■■■■■□□□□■■■■■■■	777701777760771	1916	T1					3	INH	France	EP
FXX162003100417	2003	F	89	■■■■■■■■■■■■■■■■■■■■■■■■■■■■■■■■□□□□■■■□□□■	777777777760701	1926	T1					1		France	P
FXX162008101651	2008	M	40	■■■■■■■■■■■■■■■■■■■■□□□□■■■■■■■■□□□□■■■■■■□	777777607760770	1933	LAM9	-243---7-4-2	Orphan	243742243524226	Orphan	1		Morocco	P
FXX162005000648	2005	M	26	■■■□□□□■■■■■■■■■■■□□□□□□□□□□□□□□□□■■■■■■■■■	703777000003771	1951	CAS1-Delhi					0		unknown	NA
FXX162000100344	2000	M	37	■□□■■■■■■■■■■■■■■■■■■■■■■■■■□□□□■□■■□□□□□■■	477777777413011	1966	EAI5					1		Sri lanka	EP
FXX162001200004	2001	M	50	□□■□□□□■■■■■■■■■■■■□□□□□□□□□□□□□□□■■□□■■■■■	103777400003171	1981	CAS1-Delhi					2	INH, RIF, SM	Sudan	P
FXX162002100234	2002	M	31	■■■■■■■■■□■■■■■■■□■■■■■■■■■■■■■■□□□□■■■■■■■	777376777760771	2007	T3					1		France	P
FXX162007101205	2007	M	32	■■■■■■■■■■■■■■■■■■■■■■■■■□□□■■■■□□□□■■■■■■■	777777774360771	2037	T1	223425123322	1568	234232---------	Orphan	1		Tunisia	P
FXX162006101024	2006	M	88	■■■■■■■■□□□□□■■■■■■■□□□□■■■■■■■■□□□□■■■■■■■	776037607760771	2045	LAM3	234316143324	Orphan	343434---------	Orphan	1		Algeria	EP
FXX162007001082	2007	M	23	■■■■■■■■□□□□□■■■■■■■□□□□□■■■■■■■■□□□□■■■■■■■	776037607760771	2045	LAM3	225325153323	45	253533---------	Orphan	0		unknown	NA
FXX162010002046	2010	M	43	■■■■■■■■□□□□□■■■■■■■□□□□■■■■■■■■□□□□■■■■■■■	776037607760771	2045	LAM3	-243---5-3-3	Orphan	243533143724321	Orphan	0		unknown	NA
FXX162005100547	2005	M	79	■■■■■■■■□□□□□■■■■■■■□□□□■■■■■■■■□□□□■■■■■■■	776037607760771	2045	LAM3	224326142223	Orphan	243423---------	Orphan	1		Spain	P
FXX162005100573	2005	M	28	■■■■■■■■□□□□□■■■■■■■□□□□■■■■■■■■□□□□■■■■■■■	776037607760771	2045	LAM3	224326143323	38	243433---------	Orphan	1		France	P
FXX162003100102	2003	M	78	■■■■■■■■□□□■■■□□■■□□□□□□□□□□□□□□□□□□□□□■■■■	776163000000071	2047	Unknown					1		France	P
FXX162007001367	2007	F	14	■■■□■■■■■■■■□■■■■■■■■■■■■■■■■■■■□□□□■■■■■■■	737737777760771	2052	T3	-423---5-3-4	Orphan	423534234522223	Orphan	0		unknown	NA
FXX162001100006	2001	F	24	■■■■■■□□□■■□□■■■■■■■■■■■■■■■■■■■□■■■■■□■■■■	770637777767671	2056	AFRI_1					1		Guinea	EP
FXX162006100733	2006	F	24	■■■■■■■■■■■■■■■■■■■□□□□□□□□□□□■■□□□□■■■■■■■	777777400060771	2066	Unknown					1		Senegal	P
FXX162000000358	2000	M	68	■■■■■■■■■■■■■■■■■■■■■□■■■■■■■■■■□□□□■■■□■■■	777777737760731	2088	T2					0		France	P
FXX162005300745	2005	M	78	■■■■■■■■■■■■■■■■■■■■■□■■■■■■■■■■□□□□■■■□■■■	777777737760731	2088	T2					3	SM	France	EP and P
FXX162007001345	2007	F	87	■■■■■■■■■■■■■■■□■■■■■■■■■■■■■■□■□□□□■■■■■■■	777773777720771	2089	H3	-253---5-3-3	Orphan	253533234532343	Orphan	0		unknown	NA
FXX162006000924	2006	M	84	■■■■■■■■■■■■■■■□■■■■■■■■■■■■■■□■□□□□■■■■■■■	777773777720771	2089	H3					0		unknown	NA
FXX162010102280	2010	M	41	■■■■■■■■□□□■■■■■■■■□□□□□□□□□□□□□□□□□□□■□■■■	776177400000131	2107	Unknown	-243---3-3-5	Orphan	243335253724221	Orphan	1		unknown	EP
FXX162004100488	2004	M	75	■■■■■■■■■■■■■□□□□■□□□□□□■■■■■■■■□□□□■■■■■■■	777741007760771	2191	LAM9					1		France	P
FXX162009002078	2009	M	29	■■■■■■■■■■■■□■■■■■■■■■□□□□□□□□□□□□□□□■■□■■	777737760000151	2234	Unknown	-282---1-4-2	Orphan	282142445624325	Orphan	0		unknown	NA
FXX162002100290	2002	M	30	■■■■■■■■■■■■■■■■■■■■■■□□□■■■□□■□□□□□■■■■■■■	777777743440771	2242	LAM10-CAM					1		Niger	P
FXX162008001505	2008	M	66	■■■■■■■■■■■■■■■■■■■■■■■■■■■■■■□□□□□□□□□□□■■	777777777700011	2258	Unknown	-253---5-3-2	Orphan	253532335622243	355	0		unknown	EP
FXX162010002182	2010	M	62	■■■■□□□□□■■■■■■■■■■■■■■■■■■■■■■■□□□□■■■■■■■	740777777760771	2272	T1	-233---6-3-4	Orphan	233634343723223	Orphan	0		unknown	NA
FXX162007101110	2007	F	74	■■■■□□□□□□□□□□□□□□□□□□□□■■■■■■■■□□□□■■■■■■■	740000007760771	2275	LAM3	224326153323	213	243533---------	Orphan	1		unknown	P
FXX162004100490	2004	M	84	■■■■■■■■■■■■■■■■■■■■■■■■■□□□□□□■□□□□■■■□□■■	777777774020711	2333	H1					1		France	P
FXX162009101691	2009	M	40	■■■■■■■■■□■■■■■■■■■■■■■■■□□□□□□■□□□□■■■■■■■	777377774020771	2337	H1	-253---5-3-3	Orphan	253533335632343	358	1		unknown	P
FXX162005300600	2005	F	63	■■■■■■■■■■■□□□□□□□■■■■■■■■■■■■□■□□□□■■■■■■■	777600777720771	2338	H3	224326153323	213	243533---------	Orphan	3	SM	Algeria	EP
FXX162004100504	2004	M	55	■■■■■■■■■■■□□□□□□□■■■■■■■■■■■■□■□□□□■■■■■■■	777600777720771	2338	H3	225326153323	247	253533---------	Orphan	1		Algeria	P
FXX162010102357	2010	F	23	■■■■■■■■■■■□□□□□□□■■■■■■■■■■■■□■□□□□■■■■■■■	777600777720771	2338	H3	-253---5-3-3	Orphan	253533335532343	Orphan	1		Morocco	P
FXX162006000856	2006	F	88	■■■■■□■■□□■■■■■■■■■■■■■■■■■■■■■■□□□□■■■■■■■	766377777760771	2367	S					0		unknown	NA
FXX162003100336	2003	M	37	■■■■■■■■■■■■□■□□■■■□□□□□□□□□□□□□□□□□□■■■■□□	777723400000360	2382	Unknown	223-26113321	Orphan	23-131---------	Orphan	1		Angola	EP
FXX162004100513	2004	F	54	■■■■■■■■■■■■□■□□■■■□□□□□□□□□□□□□□□□□□■■■■□□	777723400000360	2382	Unknown	223326113322	Orphan	233132---------	Orphan	1		Congo	P
FXX162004100447	2004	M	30	■■■■■■■■■■■■■■■■■■■■□□□□■■□□□□■■□□□□■□□■■■■	777777606060471	2385	LAM11-ZWE	223126152321	237	231531---------	Orphan	1		France	EP
FXX162003100320	2003	F	24	■■■■■■■■■■■■■■■■■■■■□□□□■■□□□□■■□□□□■□□■■■■	777777606060471	2385	LAM11-ZWE	223126152321	237	231531---------	Orphan	1		Congo	P
FXX162006100921	2006	M	63	■■■■■■■■■■■■■■■■■■■■■■■■■□□□□□□□□□□□□■■□■■■	777777774000331	2387	Unknown	225323153322	1582	253532---------	Orphan	1		France	EP
FXX162004100456	2004	M	73	■■■■■■■■■■■■■■■■■■■■■■■■■□□□□□□□□□□□□■■□■■■	777777774000331	2387	Unknown					1		Algeria	EP
FXX162006001053	2006	M	44	■■■■■■■■■■■■■■■■■■■■■■■■■□□□□□□□□□□□□■■□■■■	777777774000331	2387	Unknown	225323153323	43	253533---------	Orphan	0		unknown	NA
FXX162008001731	2008	F	71	■■■■■■■■■■■■■■■■■■■■■■■■■□□□□□□□□□□□□■■□■■■	777777774000331	2387	Unknown	-253---5-3-3	Orphan	253533334732334	Orphan	0		unknown	NA
FXX162009101791	2009	M	38	■■■■■■■■■■■■■■■■■■■■□□□□■■■■■□□□□□□□□□■■■■■■	777777607600371	2401	Unknown	-213---6-2-6	Orphan	213626242733312	Orphan	1		unknown	P
FXX162003100425	2003	M	36	■■■■■■■■■■■■■■■■■■■□□□■■■■■■■■■■□□□□■■■□■■■	777777437760731	2423	T2	223325153322	15	233532---------	Orphan	1		Brazil	P
FXX162005100540	2005	F	68	■■■■■■■■■■■■■■■■□■■■■■■■■■■■■■■■□□□□■■■□□□□	777775777760700	2424	T1	223325143324	112	233434---------	Orphan	1		France	EP
FXX162000000377	2000	M	25	■□□■■■■■■■■■■■■■■■■■■■■■□□□□□□□□□□□□□□□□□□□	477777770000000	2445	Unknown					0		France	P
FXX162005000667	2005	F	55	■■■■■■■■■■■■■■■■■■■■□□□□■■■■■■■■□□□□■■■□□□■	777777607760701	2551	LAM4					0		unknown	NA
FXX162007001242	2007	M	28	■■□■■□■■■■■■■■■■■■■■□□□□■■■■■■■■□□□□■■■■■■■	667777607760771	2572	LAM1	223226153321	128	232531242321312	Orphan	0		unknown	NA
FXX162009101800	2009	F	68	■■■■■■■■■■□□■■■■■■■■■■■■■■■■■■■■□□□□■■■■■■■	777477777760771	2577	T1	-234---3-2-2	Orphan	234322244522223	Orphan	1		unknown	P
FXX162009001861	2009	F	83	■■■■■■■■■■■■■■■■■■■■□□□□■■■■□□□■□□□□■■■■■■■	777777607420771	2632	Ural-1	-243---5-3-6	Orphan	243536242733312	341	0		unknown	NA
FXX162009001871	2009	F	38	■■■■■■■■■■■■■■■■■■■■□□□□■■■■□□□■□□□□■■■■■■■	777777607420771	2632	Ural-1	-243---5-3-6	Orphan	243536242733312	341	0		unknown	NA
FXX162005100534	2005	M	86	□□■■■■■■■■■■■■■■■■■■■■■■■■■■■■□■□□□□■■■■■■■	177777777720771	2644	H3	225325153323	45	253533---------	Orphan	1		Spain	EP
FXX162007101174	2007	F	71	□□■■■■■■■■■■■■■■■■■■■■■■■■■■■■□■□□□□■■■■■■■	177777777720771	2644	H3	225325153323	45	253533---------	Orphan	1		Algeria	P
FXX162008101378	2008	M	65	□□■■■■■■■■■■■■■■■■■■■■■■■■■■■■□■□□□□■■■■■■■	177777777720771	2644	H3	-253---5-3-3	Orphan	253533334632343	199	1		unknown	P
FXX162007001164	2007	F	29	■■■■■■■■■■■■■■■■■■■■■■■■■■■■■□□□□□□□□□■□■■■	777777777600131	2660	Unknown	223325162323	1563	233633---------	Orphan	0		unknown	NA
FXX162007001254	2007	M	32	■■■■■■■■■■■■■■■■■■■■■■■■■■■■■□□□□□□□□□■□■■■	777777777600131	2660	Unknown	223325162323	1563	233633---------	Orphan	0		unknown	NA
FXX162005100609	2005	M	40	■■■■■■■■■■■■■■■■■■■■■■■■■■■■■□□□□□□□□□■□■■■	777777777600131	2660	Unknown	223325162323	1563	233633---------	Orphan	1		Chad	EP and P

FXX162010101998	2010	M	24	■■■■■■■■■■■■■■■■■■■■■□□□□□□□□□□□□□□□□□■■■■■	777777700000171	2721	Unknown	-234---3-3-2	Orphan	234332245522223	308	1		Senegal	P
FXX162009001831	2009	M	59	■■■■■■■■■■■■■■■■■■■■■■■□□□□□□□□□□□□□□■■■■■■	777777760000371	2733	Unknown	-233---5-2-2	Orphan	233522344622223	Orphan	0		unknown	NA
FXX162009301886	2009	F	36	■■■■■■■■■■□■■■■■■■■■■■■■■■■■■■□■□□□□■■■■■■■	777577777720771	2735	H3	-253---5-3-2	Orphan	253532333532343	354	3	SM, INH	unknown	EP
FXX162009001902	2009	F	30	■■■■■■□□□■■■■■■■■■■■□■■■■■■■■■■■■■■■■■□■■■■	770777677777671	2765	AFRI_1	-272---4-5-1	Orphan	272451755632444	Orphan	0		unknown	NA
FXX162007001182	2007	M	48	■■■■■■■■■■■■■■■■■■■■■■■■□□□■■■■■□□□□■■■■■■■	777777770760771	2791	T1	223325143324	112	233434---------	Orphan	0		unknown	NA
FXX162009001769	2009	F	77	■□■■■■■■■■■■■■■■■■■■■■■■□□□□□□□□□□□□□□□□□□□	577777770000000	2797	Unknown	-253---3-3-3	Orphan	253333332534243	Orphan	0		unknown	NA
FXX162005100632	2005	M	39	■■■■■■□□□■■■■■■■■■■■■□□□□■■□□□□□■■■■■■□■■■■	770777703017671	2819	AFRI_1	220324243422	Orphan	203442---------	Orphan	1		Ghana	EP
FXX162007101292	2007	F	84	■■■■■■■□□□□□□□□□□□□□□□□□■■■■■■■■■□□□□■■■■■■■	774000017760771	2883	Unknown	223425173322	1569	234732---------	Orphan	1		unknown	EP
FXX162005100756	2005	F	85	■■■■■■■□□□□□□□□□□□□□□□□□■■■■■■■■■□□□□■■■■■■■	774000017760771	2883	Unknown					1		France	P
FXX162000100165	2000	M	50	■□□■■■■■□■■■■■■■■■■■■■■■■■■■□□□□■□■■■■■■■■■	476777777413771	2889	EAI5					1		France	P
FXX162008101566	2008	M	68	■□□■■■■■■□■■■■■■■■■■■□□□■■■■■■■■□□□□■■■□■■■	477377707760731	2899	T2	-233---5-3-2	Orphan	233532332432243	Orphan	1		unknown	EP
FXX162010102355	2010	M	33	□■■■■■■■■■■■□□■■■■■■■■■■■■■■■■■■□□□□■■■■■■■	377717777760771	2905	T1	-251---1-3-2	Orphan	251132332734343	353	1		Bulgaria	P
FXX162010102361	2010	M	3	□■■■■■■■■■■■□□■■■■■■■■■■■■■■■■■■□□□□■■■■■■■	377717777760771	2905	T1	-251---1-3-2	Orphan	251132332734343	353	1		Bulgaria	P
FXX162010102108	2010	M	77	■■■■■■■■□□□■□■■■■■■□□□□□□□□□□□□□□□□□□□■■■■■	776137400000171	2946	Unknown	-243---3-3-3	Orphan	243333244724221	334	1		Algeria	P
FXX162010102123	2010	F	38	■■■■■■■■□□□■□■■■■■■□□□□□□□□□□□□□□□□□□□■■■■■	776137400000171	2946	Unknown	-243---3-3-3	Orphan	243333244724221	334	1		unknown	P
FXX162009101802	2009	M	48	■■■■■■■□□□□□□■■■■■■■□□□□■■■■■■■■□□□□■■■■■■■	774037607760771	2953	LAM3	-141---5-3-1	Orphan	141531242824312	Orphan	1		unknown	EP
FXX162006300742	2006	M	84	■■■■■■■■■■■■■■■■■■■■□□□□■■■□■■■■□□□□■■■■■■■	777777607360771	3020	LAM9					3	SM	France	P
FXX162010102239	2010	M	39	■■■■■■■■■■■■■■■■■■■■■■□□□■□□□■■■□□□□■■■■■■■	777777742160771	3076	LAM10-CAM	-233---5-3-3	Orphan	233533444522327	Orphan	1		Morocco	EP
FXX162008101558	2008	M	86	□■■□■■■■■■■■■■■■■■■■■■■■■■■■■■■■□□□□■■■■■■■	337777777760771	3089	T1	-234---5-3-2	Orphan	234532244522223	311	1		unknown	P
FXX162000100278	2000	F	74	■■■■■■■■■■■■■■■■■■■■■■■■■■■■■■■□□□□□□□□□■■■	777777777740031	3091	T					1		France	EP
FXX162002100198	2002	F	28	■□■■■■■■■■■■■■■■■■■■□□□□■■■■□■■■□□□□■■■■■■■	577777607560771	3102	LAM9					1		Congo	EP
FXX162008001581	2008	M	24	□□□□□□□□□□□□□□□□□□□□□□□■■■■■■■■■□□□□■■■■■■■	000000017760771	3127	Unknown	-333---5-2-2	Orphan	333522443725123	Orphan	0		unknown	NA
FXX162001100113	2001	M	59	■■■■■■■■■■■■■■■■■■■■□□□□■■■■■■■■□□□□■■□□□□□	777777607760600	3164	LAM					1		Algeria	EP
FXX162009001771	2009	M	44	■■■■□■■■■■■■■■■■■■■■■■■■■■■■■■□■□□□□■■■□■■■	757777777720731	3179	H3	-253---5-3-2	Orphan	253532336722241	Orphan	0		unknown	NA
FXX162009001768	2009	F	78	■■■■□■■■■■■■■■■■■■■■■■■■■■■■■■□■□□□□■■■□■■■	757777777720731	3179	H3	-253---5-3-3	Orphan	253533336722243	359	0		unknown	NA
FXX162008001680	2008	F	37	■■■■■■■■■■■■■■■■■■■■■■■■■■□□□□■■□□□□■■■■■■■	777777776060771	3214	T	-423---5-3-2	Orphan	423532442622121	Orphan	0		unknown	NA
FXX162007001093	2007	M	76	■■■■■■■■■■■■■■■■■■■■■■■■■■■■■□□□□□□□□■■■■■■	777777777600371	3234	Unknown	228325163423	415	283643---------	Orphan	0		unknown	NA
FXX162003100218	2003	F	25	■■■■■■■■□□□□□□■■■■■■■■■■■□□□□□□■□□□□■■■■■■■	776017774020771	3258	H1					1		Romania	P
FXX162002100174	2002	M	29	■■■■■■■■■■■■■■■■■■■■■□□□□□□□□□□□□□□□■■■□■■■	777777700000731	3272	Unknown					1		France	P
FXX162008101598	2008	F	43	■■■■■■■■■■■■■■■■■■■■■□□□□□□□□□□□□□□□■■■□■■■	777777700000731	3272	Unknown	-233---5-2-1	Orphan	23352124262222A	Orphan	1		unknown	P
FXX162008001620	2008	F	33	□□□□□□□□□□□□□□□□□□□□□□□□■□□□□□□■□□□□■■□□■■■	000000004020631	3313	H2	-243---5-3-3	Orphan	243533334622243	348	0		unknown	NA
FXX162008001621	2008	F	92	□□□□□□□□□□□□□□□□□□□□□□□□■□□□□□□■□□□□■■□□■■■	000000004020631	3313	H2	-243---5-3-3	Orphan	243533334622243	348	0		unknown	NA
FXX162008001682	2008	F	4	□□□□□□□□□□□□□□□□□□□□□□□□■□□□□□□■□□□□■■□□■■■	000000004020631	3313	H2	-243---5-3-3	Orphan	243533334622243	348	0		unknown	NA
FXX162008001702	2008	M	60	□□□□□□□□□□□□□□□□□□□□□□□□■□□□□□□■□□□□■■□□■■■	000000004020631	3313	H2	-243---5-3-3	Orphan	243533334622243	348	0		unknown	NA
FXX162005000730	2005	M	72	■■■□□□■■■■■■■■■■■■■■■■■■■■■■■■■■□□□□■■■□□□□	707777777760700	3338	T1					0		unknown	NA
FXX162008101488	2008	M	31	□□□□□□□□□□□□□□□□□□□□□□□□■□■■■■□■□□□□■■■■■■■	000000005720771	3347	H3	-253---5-3-3	Orphan	253533333212343	360	1		unknown	EP
FXX162008001736	2008	M	38	□□□□□□□□□□□□□□□□□□□□□□□□■□■■■■□■□□□□■■■■■■■	000000005720771	3347	H3	-243---5-3-3	Orphan	243533333212343	Orphan	0		unknown	NA
FXX162009001990	2009	M	53	□□□□□□□□□□□□□□□□□□□□□□□□■□■■■■□■□□□□■■■■■■■	000000005720771	3347	H3	-253---5-3-3	Orphan	253533333212343	360	0		unknown	NA
FXX162010002307	2010	M	64	□□□□□□□□□□□□□□□□□□□□□□□□■□■■■■□■□□□□■■■■■■■	000000005720771	3347	H3	-253---5-3-3	Orphan	253533333212343	360	0		unknown	NA
FXX162009102009	2009	F	20	□□□□□□□□□□□□□□□□□□□□□□□□■□■■■■□■□□□□■■■■■■■	000000005720771	3347	H3	-253---5-3-3	Orphan	253533333212343	360	1		France	P
FXX162001100021	2001	M	50	□□□□□□□□□□□□□□□□□□□□□□□□■□■■■■□■□□□□■■■■■■■	000000005720771	3347	H3					1		Europe	P
FXX162007101111	2007	F	61	■■■■□□□□□□■■■■■■■■■■□□□□■■■■■■■■□□□□■■■■■■■	740377607760771	3436*	LAM9	124327143325	1546	243435---------	Orphan	1		unknown	P
FXX162007001117	2007	F	19	■■■■■■■■■■■■■■■■■□■■■■■■■■■■■■■■■□□□□■■■□■■	777776777770351	3437*	Unknown	221325153322	330	213532---------	Orphan	0		unknown	NA
FXX162007001116	2007	F	57	■■■■■■■■■■■■■■■■■□■■■■■■■■■■■■■■■□□□□■■■□■■	777776777770351	3437*	Unknown	22132515332-	Orphan	21353----------	Orphan	0		unknown	NA
FXX162007001097	2007	M	43	■■■■■■■■■■■■■■■■■■■■■■■■■■■■□□□■□□□□■■■■■□□	777777777420760	3438*	Ural-1	222325153222	1553	223522---------	Orphan	0		unknown	NA
FXX162005100591	2005	M	48	■■■■■■■■■■■■■■■■■■■■■■■■■■■■□□□■□□□□■■■■■□□	777777777420760	3438*	Ural-1	222325153222	1553	223522---------	Orphan	1		France	P
FXX162007001100	2007	F	28	■■■■■■■■■■■■□■■■■■■■■■■■■□■■■■■□□□□■■■■■■■	777737776760771	3439*	T3	223125153225	770	231525---------	Orphan	0		unknown	NA
FXX162007001183	2007	M	63	□■□■■■■■■■■■■■■■■■■■□□□□■■■■■■■■□□□□■■■■■■■	277777607760771	3440*	LAM1	223226153321	128	232531---------	Orphan	0		unknown	NA
FXX162010001959	2010	M	20	■■■■■■■■■■■■■■■■■■□□□□■■■■■■■■■■□□□□■■■□■■■	777777037760731	3441*	T2	-234---5-3-2	Orphan	234532242323223	312	0		unknown	NA
FXX162005100626	2005	M	60	■■■■■■■■■■■■■■■■■■□□□□■■■■■■■■■■□□□□■■■□■■■	777777037760731	3441*	T2	223325133322	938	233332---------	Orphan	1		France	P
FXX162002100225	2002	M	46	■■■■■■■■■■■■■■■■■■□□□□■■■■■■■■■■□□□□■■■□■■■	777777037760731	3441*	T2	223-2516--23	Orphan	23-6-3---------	Orphan	1		Maghreb	P
FXX162009101942	2009	M	51	■■■■■■■■■■■■■■■■■■□□□□■■■■■■■■■■□□□□■■■□■■■	777777037760731	3441*	T2	-234---5-3-2	Orphan	234532242323223	312	1		France	P
FXX162010102024	2010	F	24	■■■■■■■■■■■■■■■■■■□□□□■■■■■■■■■■□□□□■■■□■■■	777777037760731	3441*	T2	-234---5-3-2	Orphan	234532242323223	312	1		unknown	P
FXX162008101556	2008	M	65	■■■■■■■■■■■■■■■■■■□□□□■■■■■■■■■■□□□□■■■□■■■	777777037760731	3441*	T2	-234---5-3-2	Orphan	234532242323223	312	1		unknown	P
FXX162010002170	2010	F	87	■■■■■■■■■■■■□■■■■■■■■■□□□□□□□■■□□□□■■■■■■■	777737760060771	3442*	T3	-233---4-3-2	Orphan	233432346622223	Orphan	0		unknown	NA
FXX162005100630	2005	M	85	■■■■■■■■■■■■□■■■■■■■■■□□□□□□□■■□□□□■■■■■■■	777737760060771	3442*	T3	223325143324	112	233434---------	Orphan	1		France	P
FXX162005000737	2005	F	84	■■■■■■■■■■■■□■■■■■■■■■□□□□□□□■■□□□□■■■■■■■	777737760060771	3442*	T3	223326151321	Orphan	233531---------	Orphan	0		France	EP and P

FXX162007001230	2007	M	86	□■□□■■■■■■■■■■■■■■■■■■■■■■■■■■■■□□□□■■■■■■■	237777777760771	3443*	T1	223325153322	15	233532---------	Orphan	0		unknown	NA
FXX162008001386	2008	F	31	□■□□■■■■■■■■■■■■■■■■■■■■■■■■■■■■□□□□■■■■■■■	237777777760771	3443*	T1	-233---5-3-2	Orphan	233532244522223	305	0		unknown	NA
FXX162005100639	2005	M	82	■■■■■■■■■■■■□■■■■■■■■■■■■■■■■■■■□□□□□□□■■■■	777737777760071	3444*	T3	223325153724	Orphan	233574---------	Orphan	1		unknown	NA
FXX162007001159	2007	F	40	□□□■□□■■■■■■■■■■■■■■□□□□■■■■■■■■□□□□■■■■■■■	047777607760771	3445*	LAM1	224116152321	1275	241531---------	Orphan	0		unknown	NA
FXX162007101170	2007	F	34	■■■■■■■□□□□■□■■■■■■■□□□□■■■■■■□■□□□□■■■■■■■	774137607720771	3446*	H3	224326153322	1062	243532---------	Orphan	1		Caucasus	P
FXX162002100170	2002	F	63	■■■■■■■■■■■■■■■■■■□□■■■■■■■■■■□■□□□□■■■■■■■	777777177720771	3447*	H3					1		Algeria	EP
FXX162007001181	2007	F	50	■■■■■■■■■■■■■■■■■■□□■■■■■■■■■■□■□□□□■■■■■■■	777777177720771	3447*	H3	225313153323	42	253533334722243	152	0		unknown	NA
FXX162008001569	2008	M	58	■■■■■■■■■■■■■■■■■■□□■■■■■■■■■■□■□□□□■■■■■■■	777777177720771	3447*	H3	-253---5-3-3	Orphan	253533334722243	152	0		unknown	NA
FXX162009001880	2009	M	49	■■■■■■■■■■■■■■■■■■□□■■■■■■■■■■□■□□□□■■■■■■■	777777177720771	3447*	H3	-253---5-3-3	Orphan	253533334722243	152	0		unknown	NA
FXX162003300414	2003	F	23	■■■■■■■□□□□■■■■■■■■■■■■■■■■■■■□■□□□□■■■■■■■	774177777720771	3448*	H3	225313153321	174	253531---------	Orphan	3	INH	Algeria	EP
FXX162003100406	2003	M	46	■■■■■■■□□□□■■■■■■■■■■■■■■■■■■■□■□□□□■■■■■■■	774177777720771	3448*	H3	225313153323	42	253533---------	Orphan	1		France	P
FXX162006001008	2006	M	45	■■■■■■■□□□□■□■■■■■■■■■■■■■■■■■□■□□□□■■■■■■■	774137777720771	3449*	H3	225313153323	42	253533---------	Orphan	0		unknown	NA
FXX162007001251	2007	F	69	■■■■■■■□□□□■□■■■■■■■■■■■■■■■■■□■□□□□■■■■■■■	774137777720771	3449*	H3	225313153323	42	253533---------	Orphan	0		unknown	NA
FXX162010002310	2010	M	59	■■■■■■■□□□□■□■■■■■■■■■■■■■■■■■□■□□□□■■■■■■■	774137777720771	3449*	H3	-253---5-3-3	Orphan	253533334832343	374	0		unknown	NA
FXX162005100541	2005	M	25	■■■■■■■■■■■■■■■■■■■□□□□□■□■■■■□■□□□□■■■■■■■	777777405720771	3450*	H3	225325153323	45	253533---------	Orphan	1		Congo	P
FXX162009001941	2009	F	30	■□□■■■■■■■■■■■■■□■□■■■■■■□□□□□□■□□□□■■■■■■■	477775374020771	3451*	H1					0		unknown	NA
FXX162007101107	2007	F	25	■□□■■■■■■■■■■■■■□■□■■■■■■□□□□□□■□□□□■■■■■■■	477775374020771	3451*	H1	225325153323	45	253533---------	Orphan	1		Algeria	P
FXX162006100684	2006	F	27	■□□■■■■■■■■■■■■■□■□■■■■■■□□□□□□■□□□□■■■■■■■	477775374020771	3451*	H1					1		Tunisia	P
FXX162004000491	2004	M	72	■■□■■■■■□□□■■■■□■■■■■■■■■■■■■■■■■■■■■□□□□□	676173777777600	3452*	BOV	232324253322	49	323532---------	Orphan	0		France	P
FXX162004000495	2004	F	37	■■□□□□□■□■■■■■■□■■■■■■■□□□■□□□□□□□□□□■□□□□□	602773761000200	3453*	BOV_1	232324253322	49	323532---------	Orphan	0		Djibouti	EP
FXX162009001828	2009	F	88	■■■■■■■■■■■■□■■■■■■■■■■□□□□□□■■□□□□■■■■■■■	777737770060771	3454*	T3	-222---5-3-3	Orphan	222533243222223	Orphan	0		unknown	NA
FXX162005000670	2005	M	75	■■■■■■■■■■■■□■■■■■■■■■■■□□□□□□■■□□□□■■■■■■■	777737770060771	3454*	T3					0		unknown	NA
FXX162009101982	2009	M	37	■■■■■■■■■■□□■■■■■■■■□■■■■■■■■■■■□□□□■■■□■■■	777477677760731	3455*	T2	-223---5-3-3	Orphan	223533433622223	Orphan	1		unknown	P
FXX162010102019	2010	M	71	■■■■■■■■■■■■■■■■■■■□□□□□□□□■■■■■□□□□■■■■■■■	777777400760771	3456*	T1	-232---4-3-2	Orphan	232432244532222	Orphan	1		unknown	P
FXX162010102188	2010	F	24	■■□■■■■□■■■■■■■■■■■■■■□□□□■■■■■■■■□□□□■■■■■■■	675777607760771	3457*	LAM1	-232---5-3-1	Orphan	232531242421312	224	1		unknown	P
FXX162009001867	2009	M	52	■■■■■■■■■■■■■■■□■■■■■■■■■■■■■■■■□□□□■■■□■■■	777773777760731	3458*	T2	-233---4-4-2	Orphan	233442244522221	Orphan	0		unknown	NA
FXX162009001987	2009	M	83	■■■■■■■■■■■■■■■■■■■■■□□□□□□□□□■■□□□□■■■■■■■	777777700060771	3459*	T1	-233---5-3-0	Orphan	233530243623223	Orphan	0		unknown	NA
FXX162008101600	2008	F	83	■■■■■■■■■■■■■■■■■■■■■■■■■■■■□□□□□□□□□■■■■■■	777777777400371	3460*	Unknown	-233---5-3-2	Orphan	233532243422224	Orphan	1		unknown	P
FXX162008101529	2008	M	74	■■■■□■■■■■■■■■■■■■■■■■□□□■■■■■■■□□□□■■■□■■■	757777743760731	3461*	LAM10-CAM	-233---5-3-4	Orphan	233534242422224	Orphan	1		France	P
FXX162001300043	2001	F	29	■■■■□■■■■■■■■■■■■■■■■■□□□■■■■■■■□□□□■■■□■■■	757777743760731	3461*	LAM10-CAM					3	SM	France	P
FXX162009001904	2009	F	39	■■■■■■■■■■■■■□□□□□□■■■■■■■■■■■■■□□□□■■■■■■■	777740377760771	3462*	T1	-231---4-3-4	Orphan	231434343522225	Orphan	0		unknown	NA
FXX162008001414	2008	F	88	■■■■■■■■■■■■■■■■■■□□■■■■■■■■■■■■□□□□■■■□■■■	777777177760731	3463*	T2	-233---5-3-1	Orphan	233531344522223	319	0		unknown	NA
FXX162010002274	2010	M	82	■■■■■■■■■■■■■■■■■■■■■■□■□□□□□□□□□□□□□□□□□□□	777777750000000	3464*	Unknown	-233---5-3-3	Orphan	233533343222223	Orphan	0		unknown	NA
FXX162008001526	2008	F	62	■■■■■■■■■■■■■■■■■■□■■■■□□□□□□□■■□□□□■■■■■■■	777777360060771	3465*	T4	-233---6-3-4	Orphan	233634344621223	326	0		unknown	NA
FXX162009001834	2009	M	53	■■■■■■■■■■■■■■■■■■■■■□□□□□□□□□□□□□□□■■■■■■■	777777700000771	3466*	Unknown	-235---5-3-2	Orphan	235532342512223	Orphan	0		unknown	NA
FXX162009101807	2009	M	52	■■■■■■■■■■■■■■■■■■■■■■■■■■■■□□□□□□□□□□■□■■■	777777777400131	3467*	Unknown	-233---7-1-2	Orphan	233712442342323	Orphan	1		Congo	EP
FXX162009002043	2009	F	79	■■■■■■■■■■■■□■■■■■■■■■■■■■■■■■■■□□□□■■■■■■□	777737777760770	3468*	T3	-233---4-2-3	Orphan	233423453622325	Orphan	0		unknown	NA
FXX162003100310	2003	F	63	■■■■■■■■■■■■□■■■■■■■■■■■■■■■■■■■□□□□■■■■■■□	777737777760770	3468*	T3					1		Algeria	P
FXX162009001975	2009	M	18	■■■■■■■■□□□■■■■■■■■■□□□□□□□□□□□□□□□□□□□□■■■■	776177400000071	3469*	Unknown	-242---3-3-4	Orphan	24233414442-22-	Orphan	0		unknown	NA
FXX162002100241	2002	M	9	■■■■■■■■■■■■■□□□□■□□□□□□□■■■■■■■□□□□■■■□■■■	777741003760731	3470*	T2					1		France	EP
FXX162001100028	2001	M	40	■■■■■■■■■■■■■□□□□■□□□□□□□■■■■■■■□□□□■■■□■■■	777741003760731	3470*	T2					1		Germany	P
FXX162002100183	2002	M	61	■■■■■■■■■■■■■□□□□■□□□□□□□■■■■■■■□□□□■■■□■■■	777741003760731	3470*	T2					1		France	P
FXX162008101618	2008	M	55	■■■■■■■■■■■■■□□□□■□□□□□□□■■■■■■■□□□□■■■□■■■	777741003760731	3470*	T2	-243---5-1-4	Orphan	243514222624312	Orphan	1		unknown	P
FXX162008001527	2008	M	23	□□□□□□□□□□□□□□□□□□□□□□□□□□■■□■■■□□□□■■■□■■■	000000001560731	3471*	T2	-243---5-2-4	Orphan	243524242623312	337	0		unknown	NA
FXX162008301562	2008	F	48	□□□□□□□□□□□□□□□□□□□□□□□□□□■■□■■■□□□□■■■□■■■	000000001560731	3471*	T2	-243---5-2-4	Orphan	243524242623312	337	3	SM	unknown	P
FXX162009301743	2009	M	34	□□□□□□□□□□□□□□□□□□□□□□□□□□■■□■■■□□□□■■■□■■■	000000001560731	3471*	T2	-243---5-2-4	Orphan	243524242623312	337	3	SM	Gabon	P
FXX162008301377	2008	F	29	□□□□□□□□□□□□□□□□□□□□□□□□□□■■□■■■□□□□■■■□■■■	000000001560731	3471*	T2	-243---5-2-4	Orphan	243524242623312	337	3	SM	Gabon	P
FXX162010102189	2010	M	29	■■■■■■■■□□□■■■■■■■■■■□□□□■■■■■■■■□□□□■■■■■■□	776177607760770	3472*	LAM3	-243---5-3-3	Orphan	243533244324321	Orphan	1		Algeria	P
FXX162008001478	2008	F	85	■■■■□□□□□■■■■■■■■■■■□□□□■■□□□□□□□□□□□□□□□■■	740777606000011	3473*	Unknown	-243---8-3-5	Orphan	243835242722423	344	0		unknown	NA
FXX162008001522	2008	M	76	■■■■□□□□□■■■■■■■■■■■□□□□■■□□□□□□□□□□□□□□□■■	740777606000011	3473*	Unknown	-243---8-3-5	Orphan	243835242722423	344	0		unknown	NA
FXX162010002177	2010	F	62	■■■■■■■■■■■■■■□■■■■■■■■■■■■■■■■■□□□□■□□□■■■	777767777760431	3474*	T1	-243---4-3-3	Orphan	243433336732443	Orphan	0		unknown	NA
FXX162008001713	2008	F	89	■■■■■■■■■■■■■■□□□■■■■■■■■■■■■■□■□□□□■■■■■■■	777761777720771	3475*	H3	-253---5-3-3	Orphan	253533335333343	Orphan	0		unknown	NA
FXX162008001443	2008	M	58	■■■■■■■■■■■■■■□□□■■■■■■■■■■■■■□■□□□□■■■■■■■	777761777720771	3475*	H3	-253---5-4-4	Orphan	253544336333343	Orphan	0		unknown	NA
FXX162010002101	2010	M	33	■■■■■■□□□■■■■■■■■■■■■□□□■■■■■■■■■■■■■■□□■■■	770777707777631	3476*	Unknown	-254---4-5-2	Orphan	254452655603644	Orphan	0		unknown	NA
FXX162008301626	2008	M	37	■■□■■■□■□■■■■■■□■■■■■■■■■■■□■■■■■■■■■■□□□□□	672773777377600	3477*	BOV_1	-323---5-3-2	Orphan	323532554410142	385	3	PZA (bovis)	unknown	EP
FXX162009001972	2009	F	1	■■□■■■□■□■■■■■■□■■■■■■■■■■■□■■■■■■■■■■□□□□□	672773777377600	3477*	BOV_1	-323---5-3-2	Orphan	323532554410142	385	0		unknown	NA
FXX162009001933	2009	M	22	■■□■■■■■□■■■■■■□■■■■□■■■■■■■■■■□□■■■■■□□□□□	676773677747600	3478*	BOV_1	-323---5-3-2	Orphan	323532552512342	Orphan	0		unknown	NA

FXX162008101428	2008	M	67	■■■■■■■■□□■■□■□□□■□■■■■■■■■■■■■■□□□□■■■■■■■	776321377760771	3479*	S	-333---5-2-1	Orphan	333521443923123	389	1		unknown	P
FXX162002100038	2002	F	44	■■■■■■■■□□■■□■□□□■□■■■■■■■■■■■■■□□□□■■■■■■■	776321377760771	3479*	S					1		France	P
FXX162008101503	2008	F	65	■■■■■■■■□□■■□■□□□■□■■■■■■■■■■■■■□□□□■■■■■■■	776321377760771	3479*	S	-333---5-2-1	Orphan	333521443923123	389	1		unknown	P
FXX162001000073	2001	F	81	■■■■■■■□□□□□□□□□□□□■■■■■■■■■■■■■□□□□■■■■■■■	774000377760771	3480*	T1					0		France	EP
FXX162010002271	2010	M	75	■■■■■■■□□□□□□□□□□□□■■■■■■■■■■■■■□□□□■■■■■■■	774000377760771	3480*	T1	-333---5-2-2	Orphan	333522444923123	Orphan	0		unknown	NA
FXX162009002028	2009	F	82	□■■■■■■■■■■□□□□■■■■■■■■■■■■■■■■□□□□■■□■■■■	377703777760671	3481*	T1	-343---5-3-3	Orphan	343533443124123	Orphan	0		unknown	NA
FXX162009001897	2009	F	25	■■■■■■■■□□■■■■■■■■■■■■■□■■■■■■■■□□□□■■■■■■■	776377767760771	3482*	S	-433---5-3-4	Orphan	433534335924123	Orphan	0		unknown	NA
FXX162010002232	2010	M	28	■□□■■■■■■■■■■■■■■■■■■■■■■■□□□□□□■□■■□□□■■■■	477777776013071	3483*	EAI3-IND	-543---2-4-3	Orphan	543243642612614	Orphan	0		unknown	NA
FXX162008101554	2008	M	35	■□□■■■■■■■■■■■■■■■■■■■■■■■□□□□□□■□■■□□□■■■■	477777776013071	3483*	EAI3-IND	-543---2-4-3	Orphan	543243642611614	Orphan	1		unknown	P
FXX162008101338	2008	F	40	■■■□■■■■■■■■■■■■■■■■■■□■■■■■□□□□■□■■■■■■■■■	737777757413771	3484*	EAI6-BGD1	-744---2-5-3	Orphan	744253649612624	Orphan	1		Thailand	NA
FXX162001300050	2001	F	67	□□□□□□□□□□□□□□□□□□□□■■■■■■■■■■□■□□□□■■□□■■■	000000177720631	3485*	H3					3	SM	France	EP
FXX162001300085	2001	M	59	■■■■■■■■■■■■■□□□□■□■■■■■■■■■■■■■□□□□■■■■■■■	777741377760771	3486*	T4					3	SM	Congo	P
FXX162000100126	2000	F	88	■■■■■■■■■■■■■■■■■■■■■■■■■■■■■□□□□□□□■■■□■■■	777777777600731	3487*	Unknown					1		France	P
FXX162000100154	2000	F	53	■■■■□■■■■□■■■■■■■■■■■■■■■□□□□□□■□□□□■■■■■■■	757377774020771	3488*	H1					1		Kosovo	P
FXX162003100363	2003	M	52	■■■■■■■■■■■■■■■■■■■■■■□■■■□■■■■■□□□□■■■■■■■	777777756760771	3489*	T5					1		Yugoslavia	P
FXX162000000365	2000	M	40	■■■■■■■■■□■■■■■■■■■■■□□□■■■■■■■■□□□□■■■■■■■	777377707760771	3490*	T1					0		France	EP
FXX162000000366	2000	F	72	■■■■■■■■□□□■■■■■■■■■□□□□■■■■■■■■□□□□■■□■■■■	776177607760671	3491*	LAM3					0		France	EP
FXX162000000371	2000	M	73	■■■■■■■■□□■■□□□□■■■■■■■■■■■■■■■■□□□□■■■■■■■	776303777760771	3492*	S					0		France	EP
FXX162003100407	2003	M	53	■■■■■■■■■■■■■■■■■■■■□■■□□□□□□□□□□□□□□■■■■■■	777777660000371	3493*	Unknown					1		France	P
FXX162004100486	2004	F	28	■□□■■■■■■■■■■■■■■■■■■■■■■■□■□□□□■□■■■■■■■■■	477777776413771	3494*	EAI5					1		Vietnam	P
FXX162005000651	2005	F	86	■■■■■■■■■■■■□■■■■■■■■■■■■■■■■■□□□□□■■■■■■■	777737777740771	3495*	T3					0		unknown	NA
FXX162005000665	2005	M	58	■■■■■■■■■■■■■■■■■■■■■■■■■■■■■■□□□□□□■■■□■■■	777777777700731	3496*	Unknown					0		unknown	NA
FXX162005000709	2005	M	52	■■■■■■■■■■■■■■■□□■■■■■■■■■■■■■□■□□□□■■■■■■■	777771777720771	3497*	H3					0		unknown	NA
FXX162006100758	2006	F	31	■■■■■■□□□■■■■■■■■■■■■□□□■■■■■■■■■■■□■■□■■■■	770777707776671	3498*	AFRI_1					1		Burkina Faso	P
FXX162006101029	2006	M	82	■■■■■■■■■■■■■■■■■■■■■■■■■□■■■■■■□□□□■■■□□□□	777777775760700	3499*	T1					1		Algeria	P
FXX162007001131	2007	F	63	■■□■■■■■□■■■■■■□□■■■□■■■■■■■■■■■■■■■■■□□□□□	676771677777600	3709*	BOV	232324252322	1321	323532---------	Orphan	0		unknown	NA
FXX162005100622	2005	F	82	□□□□□□□□■■■■■■■■■■□■□□□□■■■■□■■■□□□□■■■■■■■	001777207560771	or001	LAM6	124326153225	427	243525---------	Orphan	1		France	EP
FXX162007101128	2007	F	27	■■■■■■■■■■■■■■■■■■■■■■□■■■■■■■■■■□□□□■■■■■□□	777777757760760	or002	T5	213325163322	Orphan	133632---------	Orphan	1		Algeria	EP
FXX162007101203	2007	M	33	■■■■■■■□□□□□□■■■■■■■■□□□□■■■■■■■□□□□■■■□□□□	774037703760700	or003	T1	223222153323	9	232533---------	Orphan	1		unknown	EP
FXX162005100596	2005	F	64	■■■■■■■■■■■■■■■■■■■■■■■□□□■■■■■□□□□□■■■■■■■	777777761740771	or004	T1	223325153322	15	233532---------	Orphan	1		Algeria	EP
FXX162007101127	2007	F	30	■■■■■■■■□□□□■■■■■■■■■□□□□■■■■■■■■□□□□■■■■■■	776077703770371	or005	Unknown	224326153323	213	243533---------	Orphan	1		unknown	EP
FXX162005100575	2005	M	50	■■■■■■■■■■■■□■■■■■□□■■■■■■■■■■□■□□□□■■■■■■■	777737177720771	or006	H3	225313153323	42	253533---------	Orphan	1		Algeria	EP
FXX162005300627	2005	F	27	■■□□□■■■□■■■■■■□■■■■■■■■■■■□□□■■□□■■■■□□□□□	616773777063600	or007	BOV_1	232324253322	49	323532---------	Orphan	3	PZA (bovis)	unknown	EP
FXX162005100569	2005	F	53	■■■■■■■■□□■■■■■■■■■■■■■■■■■■■■□■■□□□□■■■■■■	776377777730371	or008	Unknown	333325153322	361	333532---------	Orphan	1		Algeria	EP
FXX162008101599	2008	F	21	■■■■■■■■■■■■□□□□□■■□□□□□□□□□□□□□□□□□□■■□□□□	777701400000300	or009	Unknown	-233---1-3-1	Orphan	233131246222225	Orphan	1		unknown	EP
FXX162010102260	2010	F	73	■■■■■■■■■■■■■■■■■■□□□□□□■■■■■□■■□□□□■■■■■■■	777777007660771	or010	LAM9	-233---2-2-4	Orphan	233224241622222	Orphan	1		unknown	EP
FXX162008101652	2008	F	56	■■■■□□■■■■■■■■□□■■■■□□□□□□□□□□□□□□□□□□□□□□□	747763600000000	or011	Unknown	-233---5-3-5	Orphan	233535242733312	Orphan	1		unknown	EP
FXX162009101937	2009	F	78	■□■■□■■■■■□□□□□□□□□□□□□□■□□□□□□■□□□□■■■■■■■	557400004020771	or012	H1	-243---5-3-3	Orphan	243533333732343	Orphan	1		unknown	EP
FXX162009101725	2009	F	55	□□□□□□□□□□□□□□□□□□□□□□□□□□□□□□□□□□□■□□□■■■■	000000000001071	or013	Beijing	-246---4-2-1	Orphan	246421482243243	Orphan	1		unknown	EP
FXX162009301721	2009	M	28	■■■■■■■■■□□□□□□□□□□■■□□■■■■■■■■■□□□□■■■■■■■	777000317760771	or014	T3-ETH	-251---1-4-2	Orphan	251142341731343	Orphan	3	SM, INH, ETH	unknown	EP
FXX162008101490	2008	F	32	■■■■■■■■□□■■■■■■■■■■■■■■■□□□□□□□□□□□□■■■■■■	776377774000371	or015	Unknown	-253---5-3-3	Orphan	253533335632343	358	1		unknown	EP
FXX162008301497	2008	M	23	□■■■■■■■■■■■■■■■■■■■■■■■■■■■■■□□□□□□□□□□□□□	377777777700000	or016	Unknown	-253---5-3-3	Orphan	253533333532343	361	3	SM	unknown	EP
FXX162010101999	2010	F	57	■■■■■■■■■■■■■■■■■■□□■■■■■□□□□□□■□□□□■■■□■■■	777777174020731	or017	H1	-253---5-3-3	Orphan	253533334732332	Orphan	1		Tunisia	EP
FXX162003100087	2003	M	80	■■■■■■■■■■□■■□■■■■■■■■■■■■■■■■■■□□□□■□□□■■■	777557777760431	or018	T1					1		France	EP
FXX162001100092	2001	F	66	□■■■■■■■□□■■■■□■■■■■■■■■■■■■■■■■□□□□■■■■■■■	376367777760771	or019	S					1		Algeria	EP
FXX162000300128	2000	F	86	■■■■■■■■■■■■■■■■■■□□■■■■■□□□□□□■□□□□■■■■■■■	777777174020771	or020	H1					3	SM	France	EP
FXX162000100130	2000	F	70	■■■■■■■■□□■■■■■■■■■■■□□□□□□□□□□□□□□□■■□□□■■	776377700000611	or021	Unknown					1		France	EP
FXX162000100145	2000	F	64	□□□□■■■■■■■□■■■■■■■■■■■■■■■■■■□■□□□□■■■□■■■	037677777720731	or022	H3					1		France	EP
FXX162002100246	2002	M	31	■■□■■■■■□□□□□□■□■■■■□□□□■■■■■■■■□□□□■■■■■■■	676013607760771	or023	LAM12-Madrid1					1		Algeria	EP
FXX162002300259	2002	F	95	■■■■■■■■■■■■□□□□□■□□□□□□□□□□□□□□□□□■□□□■■■	777701000000431	or024	Unknown					3	ETH	France	EP
FXX162002100295	2002	F	33	■■■■■■■■■■■■■■■□□■□□□□□□□□□□□□□□□□□□■■■■■■■	777771000000771	or025	Unknown					1		Vietnam	EP
FXX162000000382	2000	F	46	■■■■■■■■■■■■■■■■■■■□□□□□□□□□□□□■□□□□■■■□■■■	777777400020731	or026	H3					0		Algeria	EP
FXX162000000389	2000	F	29	■■■■■■■■■■■■■■■□■■□□□□□□□□□□□□□□□□□□■■■□■■■	777773000000731	or027	Unknown					0		Niger	EP
FXX162003100433	2003	F	18	■■■■■■■■■■□□□□■■■■■■■□□□■■■■■■□□□□□□□□□□□□□	777417707700000	or028	Unknown					1		Niger	EP
FXX162004100492	2004	F	72	■■■■■■■■■□□□□□□□□□□□□■□■■■■■■■■■□□□□■■■■■■■	777000057760771	or029	T5					1		Algeria	EP
FXX162006100813	2006	F	76	■■■■■■■■■■■■□■■■■■■■■□■■□□□□□□■□□□□■■■■■■■	777737754020771	or030	H1					1		France	EP
FXX162006100816	2006	M	56	■■□□□■■□□□□■■■■□■■■■■■■■■■□□□□□□□□■■□■□□□□□	614173776003200	or031	BOV					1		France	EP

FXX162006100883	2006	M	78	■□□□■■■■■■■■■■■■■■■■□■■■■■■■■■■■□□□□■■■■■■■	437777677760771	or032	T1					1	Algeria	EP
FXX162006100919	2006	M	58	■■■■■■■■■■■■□■■■■■■■■■■□□□■■■■■□□□□■■■■■■■	777737770760771	or033	T3					1	France	EP
FXX162006100992	2006	F	82	■■□□■■■■■■■□■■■■■■■■□□□□■■■■■■■■□□□□■■■■■■■	637677607760771	or034	LAM1					1	France	EP
FXX162006101014	2006	M	66	■■■■■■■■■□□□□□□□■■■■■□□□■□□□□□□■□□□□■■■■■■■	777003704020771	or035	H1					1	France	EP
FXX162006101030	2006	M	86	■■□■■■■■■■■■□■■■■■■■■■■■■■■■□□□■□□□□■■■■■■■	677737777420771	or036	Ural-1					1	France	EP
FXX162007001079	2007	M	68	■■■■■■■■■■■■■■■□■■□□□□□□□□□□□□□□□□□□□□□■■■■	777773000000071	or037	Unknown	124326153326	816	243536---------	Orphan	0	unknown	NA
FXX162007001098	2007	M	22	■□□□■■■■■■■■□■■■■■■■■■■■■■■■■■■■□□□□■■■■■■■	437737777760771	or038	T3	213425143323	Orphan	134433---------	Orphan	0	unknown	NA
FXX162007001151	2007	F	83	□■■■□■■■■■■■■■■■■■■■■■■■■■■■■■■□□□□■■■■■■■	357777777760771	or039	T1	223325143324	112	233434---------	Orphan	0	unknown	NA
FXX162005000669	2005	M	83	■■■■■■■■■■■□□□□□□□■■■■■■■■■■■■■■□□□□■■■■■■■	777600777760771	or040	T1	223425153322	157	234532---------	Orphan	0	unknown	NA
FXX162007001138	2007	M	67	□□□■□■■■■■■■■■■■■■■■□□□□■■■■■■■■□□□□■■■■■■■	057777607760771	or041	LAM1	224116152321	1275	241531---------	Orphan	0	unknown	NA
FXX162007001178	2007	M	60	■■□■■■■□■■■■■■■■■■■■□□□□■■■■■■■■□□□□■■■□■■■	675777607760731	or042	LAM	224226163221	1198	242621---------	Orphan	0	unknown	NA
FXX162007001258	2007	M	82	■■□□□□□□□□□□□□□□□□□□□□□□■□□□■■■■□□□□■■□□□■■	600000004360611	or043	LAM3	224325153323	33	243533---------	Orphan	0	unknown	NA
FXX162007001146	2007	M	58	■■■■■■□□□□■■■■■■■■■■■■■■■□□□□□□■□□□□■■■■■■■	770377774020771	or044	H1	225313153323	42	253533---------	Orphan	0	unknown	NA
FXX162007001132	2007	F	74	■■■■■■■■■■■■■■■■■■■■■□□□□■□□□□□□■□□□□■■□■■■	777777702010331	or045	EAI1-SOM	225323153323	43	253533---------	Orphan	0	unknown	NA
FXX162007001139	2007	F	28	■■■■■■■■■■□■■■■■■■■■■■■□■■■■■■■■■■■■■■■■■■■	777577767777771	or046	Unknown	226423163623	Orphan	264663---------	Orphan	0	unknown	NA
FXX162005000723	2005	M	76	■■□■■■■■□■■■■■■□■■■■■■□■■■■■■■■■□■■■■■□□□□□	676773757767600	or047	BOV_1	232224252322	Orphan	322532---------	Orphan	0	unknown	NA
FXX162007001353	2007	F	82	■■■□□□□□□□□□□□□■■■■■■■■■■■■■■■■■■□□□□■■■■■■	700007777770371	or048	Unknown	-223---3-3-2	Orphan	223332342522223	Orphan	0	unknown	NA
FXX162008001442	2008	F	39	■■■■■□□□■■■■□■■■■■■■■■■■■■■■■■■■□□□□■■■□■■■	761737777760731	or049	T	-231---6-3-4	Orphan	231634244542223	Orphan	0	unknown	NA
FXX162007001364	2007	F	81	■■■■□□■■■■■■■■■■■■■■■□■■■■■■■■■■□□□□■■■□■■■	747777737760731	or050	T2	-233---3-3-2	Orphan	233332232522224	Orphan	0	unknown	NA
FXX162007001309	2007	M	41	■■■■■■■■■■■■□■■■■■■■□□□□□□□□□□□□□□□□□■■■■■	777737700000171	or051	Unknown	-233---4-3-4	Orphan	233434243122323	Orphan	0	unknown	NA
FXX162008001587	2008	F	84	■■■■■■□□□□■■■■■■■■■■■■■■■■■■■■■■□□□□■■■□□■■	770377777760711	or052	T1	-233---5-3-1	Orphan	233531243222225	Orphan	0	unknown	NA
FXX162008001741	2008	M	61	□■■■■■■■■■■■■■■■■■■■■□■■■■■■□■■■□□□□■■■■■■■	377777737560771	or053	T1	-234---5-1-3	Orphan	234513234522224	Orphan	0	unknown	NA
FXX162007001303	2007	M	69	■■■■■■■□■■■■■■■■■■■■■■■■□□□□□□□□□□□□□□□□□□□	775777770000000	or054	Unknown	-234---5-3-2	Orphan	234532244522227	Orphan	0	unknown	NA
FXX162009001780	2009	F	74	■■■■■■■■■■■■■■■■■■■■□■■■■■■■■■□■■□□□□■■■■■■■	777777677660771	or055	T1	-234---5-3-2	Orphan	234532244522222	Orphan	0	unknown	NA
FXX162010002203	2010	F	80	■■■■■■□□□□■■■■■■■■□□□□■■■■■■■■■■□□□□■■■■■■■	770377037760771	or056	T1	-234---6-3-2	Orphan	234632244322223	315	0	unknown	NA
FXX162010002054	2010	M	89	■■■■■■■■■■□■■■■■■■■■■■□■■■■■■■■■■□□□□■■■■■■■	777577737760771	or057	T1	-234---5-3-2	Orphan	234532254522223	Orphan	0	unknown	NA
FXX162009001991	2009	F	95	■■■■■■■■■■■■■□■■■■□■■■■■■■■■■■□□□□■■■■■■■■■■	777757377607771	or058	Unknown	-234---5-3-2	Orphan	234532272522223	Orphan	0	unknown	NA
FXX162008001512	2008	M	72	■■■■■□■■■■■■■■■■■■■■■■■■■■■■■■■■□□□□■■■■■□■	767777777760761	or059	T1	-234---5-3-2	Orphan	2345322-3522223	Orphan	0	unknown	NA
FXX162009001918	2009	F	17	■□■■■■■■■■□□□□□□□□□□□□□□□□□□□□□□□□□□□□■■■■■	577400000000171	or060	Unknown	-235---4-3-1	Orphan	235431245522222	Orphan	0	unknown	NA
FXX162010002269	2010	M	80	■■■■■■■■■■■■■□■■■■■□■■■■■■■■□□□□□□□□□□■■■■■■	777757377000371	or061	Unknown	-235---5-3-2	Orphan	235532242522223	Orphan	0	unknown	NA
FXX162010002154	2010	M	40	■■■■□□□□□□□□■■■■■■■■■■■■■■■■■■■■□□□□■■■□■■■	740077777760731	or062	T2	-231---4-3-2	Orphan	231432343422227	Orphan	0	unknown	NA
FXX162010002151	2010	M	74	■■■■■■■■■■■■■■■■■■■■■■■□□□□□□■■■□□□□■■■■■■■	777777760160771	or063	T1	-231---5-3-2	Orphan	231532342722228	Orphan	0	unknown	NA
FXX162009001929	2009	F	23	■■■■■■■■■■■■□□■■■■■■■■■■■■■■■■■■□□□□■□■■■■■	777717777760571	or064	T1	-231---5-3-4	Orphan	231534341221223	Orphan	0	unknown	NA
FXX162009001931	2009	F	82	■■■■■■■■■■■■■■■■□■□■■■■■■■■■■■□■■□□□□■■■■■■■	777775377660771	or065	T4	-233---5-3-3	Orphan	233533333B22224	Orphan	0	unknown	NA
FXX162008001384	2008	M	67	□□□□□□■■■■■■■■■■■■■■■■■■■■■■■■■■□□□□■■■■■■■	007777777760771	or066	T1	-233---5-3-1	Orphan	233531343422223	Orphan	0	unknown	NA
FXX162008001479	2008	F	63	■■■■□■■■■■■□□□□□□□□■■■■■■■■■■■■■□□□□■■■■■■■	757600377760771	or067	T1	-233---5-3-3	Orphan	233533344522223	323	0	unknown	NA
FXX162009001994	2009	M	61	■■■■■■■■■■■■■■■■■■■■■■□□□■■■■■■■□□□□□■■■■■■	777777743760371	or068	LAM10-CAM	-233---5-3-3	Orphan	233533345422324	Orphan	0	unknown	NA
FXX162008001468	2008	M	74	■■■■□□□□□□□□□■■■■■■■■■■■■■■■■■■■□□□□■■■■■■■	740037777760771	or069	T1	-233---5-3-4	Orphan	233534344523223	Orphan	0	unknown	NA
FXX162008001481	2008	M	83	■■■■■■■□□□□□□■■■■■■■■■□■□□□□□□□□□□□□□■■■■■■	774037750000371	or070	Unknown	-233---5-3-4	Orphan	233534342222223	Orphan	0	unknown	NA
FXX162008001521	2008	M	85	■□□□□■■■■■■■■■■■■■■■■■■■■■■■■□■■□□□□■■■■■■■	417777777660771	or071	T1	-233---5-3-4	Orphan	233534344532223	Orphan	0	unknown	NA
FXX162010002265	2010	M	83	□□□□■■■■■■■□■■■■■□□□□□□□□□□□□□□□□□□□□■■□■■■	037676000000331	or072	Unknown	-233---4-3-2	Orphan	233432353421324	Orphan	0	unknown	NA
FXX162009001786	2009	M		□□□□□■■■■■■□■■■■■■■■■■■■■■■■■■■■□□□□■■■■■■■	017677777760771	or073	T1	-232---5-3-2	Orphan	23253245342132-	Orphan	0	unknown	NA
FXX162010002214	2010	F	25	■■■■■□□□□□□□□□□□■■■■■■□□□■■■■■■■□□□□■■■■■■■	760003743760771	or074	LAM10-CAM	-233---5-3-1	Orphan	233531444522320	Orphan	0	unknown	NA
FXX162010002272	2010	M	83	□□□□■■■■■■■□■■■■■■■□■□□□□□□□□□■□□□□■■■□■■■	037677500020731	or075	H3	-233---6-3-3	Orphan	233633453421322	Orphan	0	unknown	NA
FXX162010002333	2010	F	89	■□■■■■■■■■■■■■■■■■■■□□□□□□□□□□□□□□□□□□■■■■■	577777600000171	or076	Unknown	-243---4-3-5	Orphan	243435243922222	Orphan	0	unknown	NA
FXX162008001453	2008	M	23	□□□□□□□□□□■■■■■■■■■■□□□□□□■■□■■■□□□□■■■■■■■	000377601560771	or077	T1	-243---5-2-4	Orphan	243524242723312	Orphan	0	unknown	NA
FXX162008001575	2008	M	70	■■■■■■■■■■■■■■■■■■■■□□□□■■□□□□□□□□□□□□■■■■■	777777606000171	or078	Unknown	-243---5-3-5	Orphan	243535242233312	Orphan	0	unknown	NA
FXX162010002329	2010	F	66	■■■■■■■■■■■■■■■■■■■■□□■■■■■■□□□□□□□□□□□■■■■	777777637400071	or079	Unknown	-243---5-3-6	Orphan	243536253623329	Orphan	0	unknown	NA
FXX162008001477	2008	F	88	■■■■■■■■■■■■■■■■■■■■■■□□□□□□□□□□□□□□□□□■■■■	777777740000071	or080	Unknown	-243---3-3-4	Orphan	243334332022223	Orphan	0	unknown	NA
FXX162009001905	2009	M	2	■■□□□□□□□□□□□□□□□□□□□□□□■□□□■■■■□□□□■■□□□□■	600000004360601	or081	LAM3	-243---5-3-3	Orphan	243533333932442	Orphan	0	unknown	NA
FXX162010002229	2010	M	20	■□□□□■■■■□□■■■■■■■■■■■■■■■■■■■■■□□□□■■■□■■■	417177777760731	or082	T2	-252---4-3-3	Orphan	252433232712254	Orphan	0	unknown	NA
FXX162010002130	2010	F	88	■■■■■■■■■■■■■■■■■■□□□□□■■□□□□□□■□□□□■■■■■■■	777777014020771	or083	H1	-253---5-3-3	Orphan	253533233732343	Orphan	0	unknown	NA
FXX162008001739	2008	M	83	■■■■■■■■■■■■■■■■■■■□□□□□□■□□□□□□□□□□□■■■■■■	777777402000371	or084	Unknown	-253---5-3-2	Orphan	253532335533343	Orphan	0	unknown	NA
FXX162008001524	2008	F	76	■■■■■■■■■■■■■■■■■■■■■■□□■□■■■■□■□□□□■■■■■■■	777777745720771	or085	H3	-253---5-3-3	Orphan	253533334732345	Orphan	0	unknown	NA
FXX162008001634	2008	F	17	□□□□□□□□□□□□■■■■■■■■■■■■■□□□□□□■□□□□■■■■■■■	000077774020771	or086	H1	-253---5-3-3	Orphan	253533334832323	Orphan	0	unknown	NA
FXX162009001776	2009	F	61	■■■■■■■■■■■■■■□□■■□□□□□□■□□□□□□■□□□□■■■■■■■	777763004020771	or087	H1	-253---5-3-3	Orphan	253533334633343	Orphan	0	unknown	NA
FXX162010002266	2010	M	38	■■■□□□□□□□□■■■■■■■■■■■■■■□□□□□□■□□□□■■■□■■■	700177774020731	or088	H1	-253---5-3-3	Orphan	253533336732335	Orphan	0	unknown	NA

FXX162010002295	2010	M	77	■■■■□■■■■■□□□■■□□□□□□□□□□□□□□□□□□□□□□■■■■■■	757430000000371	or089	Unknown	-253---5-3-3	Orphan	253533335522345	Orphan	0		unknown	NA
FXX162008001474	2008	F	19	■■■■■■■□□□□□■■■■□□□□□□□□■■□□□□□■■■■■□□□■■■■	774074006037071	or090	AFRI_3	-254---4-2-1	Orphan	25442144-333243	Orphan	0		unknown	NA
FXX162009001976	2009	F	82	■■■■■■■■■■■■■■□■■■■■■■■■□■□□□□■□■■■■■■■■■■■■	777767776413771	or091	EAI5	-2-----4---3	Orphan	2--4-3-------2-	Orphan	0		unknown	NA
FXX162008001637	2008	F	95	■■■□□□□□□□□□□□□□■□□□□□□□□□□□□□□□□□□□□□□□□□□	700002000000000	or092	CAS	-324---4-y-2	Orphan	3244y2243522223	Orphan	0		unknown	NA
FXX162008001697	2008	F	26	□□□□□■■■□□■■□■■■■■■■■■■■■■■■■■■■■■□□□□■■■□■■■	016337777760731	or093	S	-333---5-2-2	Orphan	333522345723123	Orphan	0		unknown	NA
FXX162008001517	2008	M	55	■■□□□□□□□□□■■■■■■■■■■■■■■■■■■■■■□□□□■■■■□■■	600177777760751	or094	T1	-333---5-2-4	Orphan	333524443623123	Orphan	0		unknown	NA
FXX162008001734	2008	M	39	■□□■■■□■■■■■■■■■■■□■■■■■■■■■□□□□■□■■■■■■■■■	473777377413771	or095	EAI8-MDG	-543---2-5-3	Orphan	543253543312514	Orphan	0		unknown	NA
FXX162010002211	2010	M	65	■□□■■■■■■■■■■■■■■■■■■■■■■■■■□□□□□□□□□□□□□□■	477777777400001	or096	Unknown	-543---2-4-5	Orphan	543245643612614	Orphan	0		unknown	NA
FXX162010002273	2010	F	54	■■■■■■■■□■■■■■■■■■■■■■■■■■■■□□□□■□■■■■■□■■■	776777777413731	or097	EAI1-SOM	-653---2-4-3	Orphan	653243642612923	Orphan	0		unknown	NA
FXX162010002221	2010	F	21	■■■■□■■■■■■■■■■■■■■■■■□■□■■■□□□□■□■■■■■■■■■	757777753413771	or098	EAI6-BGD1	-943---2-5-3	Orphan	943253942612424	Orphan	0		unknown	NA
FXX162005000647	2005	F	41	■■■■■■■■■■■■■■■■■■□□□□□□■■■■□■■■□□□□■■■■■■■	777777007560771	or099	LAM6					0		unknown	NA
FXX162005000701	2005	M	64	□■■■■■■■■■□□■■■■■■■■□□□□■■■■■■■■□□□□□□□□■■■	377477607760031	or100	LAM4					0		unknown	NA
FXX162005000703	2005	M	69	■■■■■■■■■■■■■■■■■■■□■■□■■■■■■■■■■□□□□■■□□■■■	777777557760631	or101	T5-Madrid2					0		unknown	NA
FXX162005000717	2005	M	62	□□□□■■■■■■■□■■■■■□□□□□□□□□□□□□□□□□□□□■■■■□□	037676000000360	or102	Unknown					0		unknown	NA
FXX162005000721	2005	F	33	■■■■■■■■■■■■■■■■■■■■□□□□■■■□□□□■□□□□■■■□■■■	777777607020731	or103	H3					0		unknown	NA
FXX162005000724	2005	M	75	■■■■■■■■■■■■■■■■■■■■■■■■□□□□□□□□□□□□■■□□■■■	777777770000631	or104	Unknown					0		unknown	NA
FXX162005000729	2005	F	86	■■■■■■■□□□□■■■■■■■□■■■■■■□□■■■■■□□□□■■■■■■■	774177374760771	or105	T4					0		unknown	NA
FXX162006000853	2006	F	44	■■■■■■■■■■■■■■■■■■■■■■■■■■■■■■□■□□□□■■□□□□■	777777777720601	or106	H3					0		unknown	NA
FXX162006000859	2006	F	52	□■□□□□□□□□□■■■■■■■■■■■■■■■■■■■■■□□□□■■■■■■■	200177777760771	or107	T1					0		unknown	NA
FXX162006000867	2006	F	83	■□■■■■■■■■■■□■■■■■■■■■■■□□□□□□□□□□□□■■■■■■■	577737770000771	or108	Unknown					0		unknown	NA
FXX162006000873	2006	M	44	■□■■■■□□□□□□□□□□□□■■■■■■■■■■■■■■□□□□■■■■■■■	570000777760771	or109	T1-RUS2					0		unknown	NA
FXX162006000876	2006	F	69	■■■□□□□□□□□□■■■■■□■■□■■■■■■■■■■■□□□□■■■■■■■	700076677760771	or110	X3					0		unknown	NA
FXX162006000889	2006	F		■■■■■■■■■■■■■■■■■■□□□□■■■■■■■■□□□□□□■■■■■■■	777777037700771	or111	Unknown					0		unknown	NA
FXX162006000900	2006	F	15	■■■■■■■■■■■■□■■■■■■■□□□□■■■□□□■□□□□■■■■■■■	777737703420771	or112	Ural-1					0		unknown	NA
FXX162006000913	2006	F	72	■■■■■■■■■□□□□□□□□□□■□□□□□□□□□□□□□□□□□□□□□□□	777000200000000	or113	Unknown					0		unknown	NA
FXX162006000941	2006	M	77	□□■■■□□□□□□□□□□□□■■□□□□□□□□□□□□□□□□□□□■■■■■	160001400000171	or114	Unknown					0		unknown	NA
FXX162006001004	2006	M	42	■■■■■■■■■■■■■■■■■■■■■■□□□■■■■■□■□□□□■■■■■■■	777777743720771	or115	H3					0		unknown	NA
FXX162006001035	2006	F	91	■■■■■■■■■■■■■■■□□■□■■■■■■■■■■■■■□□□□■■■■■■■	777771377760771	or116	T4					0		unknown	NA
FXX162006001036	2006	M	47	■■■■■■■■■■■■□□□□■■■■■■■■■■□■■■■■□□□□■■■■■■■	777703776760771	or117	T1					0		unknown	NA
FXX162006001037	2006	M	83	■■■■□■■■■□□■■■■■■■■■■■■■■■■■■■■■□□□□■■■■■■■	757177777760771	or118	T1					0		unknown	NA
FXX162006001046	2006	M	81	■■■■□■■■■■■■■■■■■■□■■■□□■■■■■■■■□□□□■□□■■■■	757777347760471	or119	T4-CEU1					0		unknown	NA
FXX162006001055	2006	F	78	■■■■■■■■■■■■■■■■■■■■■■■■□□□□□□■■□□□□■□■■■■■	777777770060571	or120	T1					0		unknown	NA
FXX162009002044	2009	M	22	■■■■■■■■■■■■■□□□□■□□□□□□□□□□□■■■□□□□■■■■■■■	777741000160771	or121	T1					0		unknown	NA
FXX162005100585	2005	M	83	■□■■■■■■■■■■■■■■■■□□■■■■■■■■■■■■□□□□■■■■■■■	577777177760771	or122	T1	222325143321	1552	223431---------	Orphan	1		France	P
FXX162005100566	2005	M	71	■□■■■■■■■■■■■■■■■■■■■■■■■■□■□□□■□□□□■■■□■■■	577777776420731	or123	Ural-2	2223251534-3	Orphan	223543---------	Orphan	1		Azerbaijan	P
FXX162005100570	2005	M	36	■■■■■■■■■■□□□□□□□□□■■■■■■■■■■■■■□□□□■■■■■■■	777400377760771	or124	T1	223324153325	1559	233535---------	Orphan	1		France	P
FXX162001100110	2001	M	43	■■□■■■■■■■■■■■■■■■□□□□□□□□□□□□□□□□□□□■■□■■■	677777000000331	or125	Unknown	223325143423	1561	233443---------	Orphan	1		Central African Republic	P
FXX162006100978	2006	M	56	■■□■■■■■■■■■■■■■■■□□□□■■■■■■■■■■□□□□■■■■■■■	677777037760771	or126	T1	22332-163322	Orphan	233632---------	Orphan	1		Spain	P
FXX162005100693	2005	F	40	■■■■■■■□□□□□■■■■■■■■■■■■□■□□□□□□□□□■□□□■■■■	774077772001071	or127	AFRI_3	224123244221	Orphan	241421---------	Orphan	1		Angola	P
FXX162005100529	2005	M	53	■■■■■■□□□□■■□■■■■■■■■■■■■■■■□□□■□□□□■■■■■■■	770337777420771	or128	Ural-1	225225113322	1491	252132---------	Orphan	1		France	P
FXX162005100594	2005	M	23	■■■■■■■■■■■□■■■■■■■■■■■■■■■■□□□□■□■■■□□□■■■	777677777413431	or129	EAI5	265225223632	Orphan	652262---------	Orphan	1		Cambodia	P
FXX162010102250	2010	M	52	■■■■■■□□□□□□□□□□□□■■■■□■■■■■■■■■■□□□□■■■□□■■	770000757760711	or130	T1-RUS2	-221---6-3-3	Orphan	221633344322225	Orphan	1		Romania	P
FXX162009101938	2009	F	97	□■■■■■■■■■■■□□□□■■■■■■■■■■■■■■■■□□□□■■■□■■■	377703777760731	or131	T2	-233---8-3-4	Orphan	233834242522223	Orphan	1		France	P
FXX162009101838	2009	M	45	■■■■■■■■■■■■■■■■■■■■■□■■■■■■■■■■■■□□□□■■■■□■■	777777737760751	or132	T1	-231---5-3-4	Orphan	231534343422223	316	1		Poland	P
FXX162010102119	2010	F	83	■■■■□■■■■■■■■■■■■■□□□□□□□□■■■■■■□□□□■■■■■■■	757777001760771	or133	T1	-232---5-2-2	Orphan	232522342522125	Orphan	1		France	P
FXX162010101997	2010	M	82	□■■■■■■■■■■■■■■■■■■■□□□□■■■■■■■■□□□□■□■■■■■	377777607760571	or134	LAM9	-242---5-3-1	Orphan	242531243724312	330	1		unknown	P
FXX162010102254	2010	M	65	■■■■■■■■■■■■■■■□■■■■■■□□□□□□□□□□□□□□□□□□■■■	777773740000031	or135	Unknown	-243---4-2-4	Orphan	243424342-22223	Orphan	1		France	P
FXX162009101798	2009	M	75	■■■■■■■■■■■■■■■□□■□□□□□□■□□■■■□■□□□□■■■■■■■	777771004720771	or136	H3	-253---5-3-3	Orphan	253533333622243	Orphan	1		Algeria	P
FXX162010102183	2010	M	72	■■■■■■■■■■■■■■■■■■■■■□□■■■■■■□□■□□□□■■■□■■■	777777717620731	or137	H3	-3-3---4-3-2	Orphan	3-34323335-3343	Orphan	1		France	P
FXX162010102020	2010	F	63	□■■■■□□■■■■■■■■■■■■■■■■■■■■■□□□□□□■■■■■■■■■	363777777403771	or138	Unknown	-542---2-6-3	Orphan	542263542712524	Orphan	1		Cambodia	P
FXX162010002172	2010	F	67	■□□■■■■■■■■■■■■■■■□□□■■■■■■■□□□□■□□■■■■■■■■	477777077411771	or139	EAI5	--43---2-5-2	Orphan	-43252644613514	Orphan	0		unknown	P
FXX162001300003	2001	F	9	■■■□□□□□□□□□□□□□■■■■■■■■■□□□□□□■□□□□■■■■■■■	700003774020771	or140	H1					3	SM, INH	France	P
FXX162001100020	2001	F	39	□□□□□□□□□□□□□■□□□□□□□□□□□□□□□□□□□□■■■■■■■■■	000020000003771	or141	Unknown					1		France	P
FXX162001000027	2001	M	88	■■■■■□■■■■■■■■■■■■□■■■■■■■■■■■■■□□□□■■■■■■■	767777377760771	or142	T4					0		Algeria	P
FXX162001100047	2001	M	86	■■■■□□□□□□□□□□□□■■■■■■■■■■■■□□□■□□□□■■■■■■■	740003777420771	or143	Ural-1					1		Poland	P
FXX162001100053	2001	M	50	□□□□■■■■■■■■■□□□□■■□□□□□□■■■■■■■□□□□■■■□■■■	037741403760731	or144	T2					1		Algeria	P
FXX162001300079	2001	F	31	■■■■■■■■□□■■■■■■■■■■■■■■□□■■■■■■□□□□■■■■■■■	776377771760771	or145	S					3	SM	Senegal	P

FXX162001100101	2001	F	77	■■■■■■■■■■■■■■■■■■□□□□□□□□□□□□□□□□□□□□□■■■■	777777000000071	or146	Unknown					1		France	P
FXX162003100103	2003	M	80	■■■■■■■■■■■■■■■■■■□□□□□□■■■■■■■■□□□□■■□■■■■	777777007760671	or147	LAM9					1		Spain	P
FXX162001000107	2001	M	86	■■■□□□□□□□□□□■■■■■■■■■■■■■■■■■■■□□□□■■■□■■■	700037777760731	or148	T2					0		France	P
FXX162002100220	2002	M	83	■□□□□■■■■■■■■■■■■■■■■■■■■■■■■■■■□□□□■■■■■■■	417777777760771	or149	T1					1		Turkey	P
FXX162003100254	2003	M	43	■□□■■■■□□□■■■■■■■■■■■■■■■□□■□□□□■□■■■■■■■■■	474377774413771	or150	EAI4-VNM					1		France	P
FXX162000100265	2000	M	65	■■■■■■■■■■■■■■■□■■□■■■■■■■■■■■□■□□□□■■■■■■■	777773377720771	or151	H3					1		Algeria	P
FXX162000100273	2000	M	76	■■■■■■□■■■■■■■■■■■■■■■■■■□□■□□□□■□■■■■■■■■■	773777774413771	or152	EAI4-VNM					1		Asia	P
FXX162000100283	2000	M	83	■■■■■■■■■■■■■■□□■■□□□□□□■□□□□■□■□□□□■■■■■■■	777763004120771	or153	H3					1		France	P
FXX162000100286	2000	M	27	■■■■■■■■■■■■■■■■■■□□■■■■■■■■■■■■□□□□■□■■■■■	777777177760571	or154	T1					1		France	P
FXX162003000321	2003	M	85	■■■■■■■■■■■■■■■■■■■□■□□□□□□■■■■■□□□□■■■■■■■	777777500760771	or155	T1					0		France	P
FXX162000100327	2000	M	84	■■□■■□□□□□□□□□□□■■■□□□□□□□□□□□□□□□□□□□■■■■■	660003400000171	or156	Unknown					1		France	P
FXX162003100338	2003	M	31	■■■■■■■■■■■■□□□■■■■■■■□□□■■■■■■■□□□□■■■■■■■	777707743760771	or157	LAM10-CAM					1		Congo	P
FXX162003100349	2003	M	55	■■■■□■■□□□□■■■■■■■■■■■■■■■■■□□□□■□■■■■■□■■■	754177777413731	or158	EAI1-SOM					1		Thailand	P
FXX162000000368	2000	M	76	■■■■■■■■■■■■■■■■■■■■■■■■■□□□□□□■□□□□■■■□■□□	777777774020720	or159	H1					0		France	P
FXX162000000369	2000	F	91	■■■■■■■■■■■■■■■□□□■■■■■■■■■■■■□■□□□□■■■■■■■	777770777720771	or160	H3					0		France	P
FXX162000000383	2000	F	74	■■■■■□□□□□□□□□□□■■■□□□□□□□□□□□□□□□□□□□■■■■■	760003400000171	or161	Unknown					0		France	P
FXX162003100411	2003	M	56	■■■■■■■■■■■■□□□□□■□□□□■■■■■■■■■■□□□□■■■■■■■	777701037760771	or162	T1					1		France	P
FXX162003100429	2003	F	36	■■■■■■■■■■■■■□□□□■■■■■■□■■■■■■□■□□□□■■■■■■■	777741767720771	or163	H3					1		Romania	P
FXX162004300457	2004	M	35	■■■■■■■■■■■■■■□□□■□□□□□□□■■■■■■■□□□□■■■■■■■	777761003760771	or164	T1					3	SM, INH	Georgia	P
FXX162004100497	2004	M	81	■■■■■■□□□□□□□□□□□□□□□□□□■■■■■■□■□□□□■■■■■■■	770000007720771	or165	H3					1		France	P
FXX162004000498	2004	F	90	□■□□□□□□□□□□□■■■■■■■■■■■■■■■■■■■□□□□■■■■■■■	200037777760771	or166	T1					0		France	P
FXX162006100760	2006	M	74	■■■■■■■□□□□□□■■■■■■■□□□□□□□□□□□□□□□□□□□□□□□	774037600000000	or167	Unknown					1		Maghreb	P
FXX162006100785	2006	F	75	■■□■■■■■□□□□□■■■■■■■■■■■■■■■■■■□□□□□■■■■■■■	676037777740771	or168	T1					1		France	P
FXX162006100826	2006	M	30	■■■□□□□□□□□□■■■■■■■■■■■■■■■■■■■■□■■■■■□■■■■	700077777767671	or169	AFRI					1		Guinea	P
FXX162006100833	2006	F	32	■■■■■■□□□□□□■■■■■■■■■■■■■■■■■■■■□□□□■■■■■■■	770077777760771	or170	T1					1		Algeria	P
FXX162006100946	2006	F	88	■■■■□□□□■■■■■■□□□■□□□□□□■■■■■■■■□□□□■■■■■■■	741761007760771	or171	LAM9					1		France	P
FXX162006100947	2006	M	82	■■■■■■■■■■■■■■□□□■□□□□□□■□□□□□□■□□□□■■■■■■■	777761004020771	or172	H1					1		Italy	P
FXX162006100987	2006	M	61	■■■■■■■□□□□□□■■■■■■■□□□□□■■■■■■■□□□□■■■□□□□	774037603760700	or173	T1					1		Algeria	P
FXX162006100994	2006	M	24	■■■■■■■■■■■■□■□□□■□□□□□□■■■■■■■■□□□□■■■■■■■	777721007760771	or174	LAM5					1		Azerbaijan	P
FXX162006101026	2006	M	67	■■■■■■■■■■□□□□□□□□□□□□■■■■■■■■■■■□□□□■■■■■■	777400037770371	or175	Unknown					1		France	P
FXX162007101063	2007	M	19	■■■■■■■■■■■□□■□□■■■■■■■■■■■■■■■■□□□□■■■□■■■	777623777760731	or176	T2	223325154423	847	233543---------	Orphan	1		Congo	P
FXX162008001547	2008	M	79	■■■■■■■■■■■■■■■■■■■■■■■■■□□□□□□□□□□□□□□□■■■	777777774000031	or177	Unknown	-233---3-2-1	Orphan	233321244322221	Orphan	0		unknown	P
FXX162008101623	2008	F	81	■■□■■□□□□□□□□□□□□■■□□□□□□□□□□□□□□□□□□□■■■■■	660001400000171	or178	Unknown	-233---3-3-4	Orphan	233334243723221	Orphan	1		unknown	P
FXX162008101459	2008	M	60	■■■■■■■■■■■■□■■□□■■■□□□□□■■■■■■■■□□□□■■■■■■	777731603770371	or179	Unknown	-243---4-3-4	Orphan	243434242722314	Orphan	1		unknown	P
FXX162008101388	2008	M	88	■□■■■■■■■■■■■■■■■■■■□□□□■■■■■■■■□□□□■□■■■■■	577777607760571	or180	LAM9	-243---6-3-5	Orphan	243635242822222	Orphan	1		unknown	P
FXX162008101550	2008	M	61	■■■□□■■■■■■■■■■■■■■■■■■■■■■■■■■□■□□□□■■■■■■■	717777777720771	or181	H3	-253---5-3-1	Orphan	253531336732243	Orphan	1		unknown	P
FXX162008101458	2008	M	54	■■■□□■■■■■■■■■■■■■■■■■■■■■■■□□□□■□■■■■■■■■■	717777777413771	or182	EAI5	-542---1-5-3	Orphan	542153442512626	Orphan	1		unknown	P

* SIT followed by an asterisk indicates a "newly created shared-type" after match with another orphan in the database, or due to 2 or more strains belonging to a new pattern within this study (highlighted in yellow)

** DST Code: Drug susceptibility testing codes “0” not available, “1” pansusceptible, “2” MDRTB (with or without resistance to other drugs), “3” any other resistance(s)

***Type of disease: Pulmonary (P), Extra-pulmonary (EP), or information not available (NA)

True orphans are highlighted in blue; the lineages for orphan patterns are "suggested lineages based on visual interpretation" (not provided by SITVIT2 database)

Note that the 'x' letter in MIRU-VNTRs designates value 2s+3s (highlighted in red)
